# Systematic Review of the Epidemiological Burden of Myalgic Encephalomyelitis/Chronic Fatigue Syndrome Across Europe: Current Evidence and EUROMENE Research Recommendations for Epidemiology

**DOI:** 10.3390/jcm9051557

**Published:** 2020-05-21

**Authors:** Fernando Estévez-López, Kathleen Mudie, Xia Wang-Steverding, Inger Johanne Bakken, Andrejs Ivanovs, Jesús Castro-Marrero, Luis Nacul, Jose Alegre, Paweł Zalewski, Joanna Słomko, Elin Bolle Strand, Derek Pheby, Evelina Shikova, Lorenzo Lorusso, Enrica Capelli, Slobodan Sekulic, Carmen Scheibenbogen, Nuno Sepúlveda, Modra Murovska, Eliana Lacerda

**Affiliations:** 1 Department of Child and Adolescent Psychiatry/Psychology, Erasmus MC University Medical Center, P.O. Box 2040, 3000 CA Rotterdam, The Netherlands; 2 Department of Clinical Research, Faculty of Infectious & Tropical Disease, London School of Hygiene & Tropical Medicine, London WC1E 7HT, UK; Kathleen.Mudie1@lshtm.ac.uk (K.M.); Luis.Nacul@lshtm.ac.uk (L.N.); Nuno.Sepulveda@lshtm.ac.uk (N.S.); Eliana.Lacerda@lshtm.ac.uk (E.L.); 3 Warwick Medical School, University of Warwick, Coventry CV4 7HL, UK; xiasteverding@gmail.com; 4 Centre for Fertility and Health (CeFH), Norwegian Institute of Public Health, 0456 Oslo, Norway; Inger.Johanne.Bakken@helsedir.no; 5 Statistics Unit, Riga Stradins University, LV-1007 Riga, Latvia; Andrejs.Ivanovs@rsu.lv; 6 ME/CFS Unit, Division of Rheumatology, Vall d’Hebron Hospital Research Institute (VHIR), Universitat Autònoma de Barcelona, 08035 Barcelona, Spain; jesus.castro@vhir.org (J.C.-M.); jalegre@vhebron.net (J.A.); 7 Department of Hygiene, Epidemiology, Ergonomics and Postgraduate Education, Collegium Medicum in Bydgoszcz, Nicolaus Copernicus University in Toruń, 85-094 Bydgoszcz, Poland; p.zalewski@cm.umk.pl (P.Z.); jslomko@cm.umk.pl (J.S.); 8 Faculty of Health Studies, VID Specialized University, 0370 Oslo, Norway; Elin.Bolle.Strand@vid.no; 9 Norway & National Advisory Unit on CFS/ME, Oslo University Hospital, 0424 Oslo, Norway; 10 Faculty of Health and Society, Buckinghamshire New University, High Wycombe HP11 3JZ, UK; derekpheby@btinternet.com; 11 Department of Virology, National Center of Infectious and Parasitic Diseases, Sofia, Bulgaria & The National Specialized Hospital for Active Treatment in Haematological Diseases, 1233 Sofia, Bulgaria; evelina_sh@abv.bg; 12 Neurology Department, ASST-Lecco, 23807 Merate, Italy; lorusso.lorenzo@gmail.com; 13 Department of Earth and Environmental Sciences and Centre for Health Technologies, University of Pavia, 27100 Pavia, Italy; enrica.capelli@unipv.it; 14 Department of Neurology, Medical Faculty Novi Sad, University of Novi Sad, 21000 Novi Sad, Serbia; slobodan.sekulic@mf.uns.ac.rs; 15 Institute for Medical Immunology, Charité-Universitätsmedizin Berlin, 13353 Berlin, Germany; Carmen.Scheibenbogen@charite.de; 16 Centre of Statistics and Its Applications, University of Lisbon, 1749-016 Lisbon, Portugal; 17 Institute of Microbiology and Virology, Riga Stradins University, LV-1007 Riga, Latvia; modra@latnet.lv

**Keywords:** central nervous system diseases, infections, muscular diseases, post-exertional malaise, virus diseases

## Abstract

This review aimed at determining the prevalence and incidence of Myalgic Encephalomyelitis/Chronic Fatigue Syndrome (ME/CFS) in Europe. We conducted a primary search in Scopus, PubMed and Web of Science for publications between 1994 and 15 June 2019 (PROSPERO: CRD42017078688). Additionally, we performed a backward-(reference lists) and forward-(citations) search of the works included in this review. Grey literature was addressed by contacting all members of the European Network on ME/CFS (EUROMENE). Independent reviewers searched, screened and selected studies, extracted data and evaluated the methodological and reporting quality. For prevalence, two studies in adults and one study in adolescents were included. Prevalence ranged from 0.1% to 2.2%. Two studies also included incidence estimates. In conclusion, studies on the prevalence and incidence of ME/CFS in Europe were scarce. Our findings point to the pressing need for well-designed and statistically powered epidemiological studies. To overcome the shortcomings of the current state-of-the-art, EUROMENE recommends that future research is better conducted in the community, reviewing the clinical history of potential cases, obtaining additional objective information (when needed) and using adequate ME/CFS case definitions; namely, the Centers for Disease Control & Prevention−1994, Canadian Consensus Criteria, or Institute of Medicine criteria.

## 1. Introduction

Myalgic Encephalomyelitis/Chronic Fatigue Syndrome (ME/CFS) is a disease characterised by post-exertional malaise, including persistent fatigue, and other symptoms aggravated by physical or cognitive efforts, at intensities previously well tolerated by the individual. Post-exertional symptoms may be experienced immediately or, more typically, may be delayed for hours, days, or even longer. They are associated with slow recovery, which may extend to one or more days, together with a heterogeneous array of other symptoms that may include musculoskeletal pain, sleep disturbances, and impaired cognition, among many others [1,2,3,4]. Thus, ME/CFS often decreases health-related quality of life, and affects employment, and the social and familial relationships of patients [5,6,7,8,9]. Although it is unclear whether ME and CFS are two different diseases [10], we will pragmatically use the term ME/CFS.

An additional common burden for patients and their families is lack of recognition of ME/CFS as a serious disease in various countries [6,11]. This lack of official recognition could be explained by poor knowledge of the disease on the parts of different health-related stakeholders. In addition, symptoms often fluctuate over time in the same patient. Also, the use of different definitions for the disease could lead to the production of estimates of ME/CFS prevalence and incidence which are not directly comparable, even in the same population [12]. For example, the prevalence of ME/CFS in Iceland was estimated at between 0 and 5% using two different but widely accepted case definitions [13]. Thus, the real burden of ME/CFS across populations remains elusive, and is a matter of controversial debate.

Previous systematic reviews on the prevalence and incidence of ME/CFS included studies from many parts of the world [14,15,16,17,18]. However, most of these reviews were conducted some years ago [14,15,16,17]. In addition, they neither reported the incidence of ME/CFS nor included children or adolescents [14,15,16,17,18]. Also, the reviews were not easily comparable, due to the use of different case definitions for ME/CFS. The quality of reporting was not adequately evaluated in most of these reviews, even in the most recent study [18]. Most importantly, none of these previous reviews focused on the epidemiology of ME/CFS in Europe. The European Epidemiological Study for ME/CFS (Euro-EpiME), from the European Network on ME/CFS (EUROMENE, EU-funded COST Action; Reference number: 15111) is intended to fill this knowledge gap by performing a systematic review of epidemiological data on ME/CFS in Europe.

Therefore, the aim of the present systematic review was to determine the prevalence and incidence of ME/CFS in Europe and to overcome the shortcomings of the previous reviews.

## 2. Methods

### 2.1. General Information Concerning the Systematic Review

The present literature review is in accordance with the PRISMA framework for systematic reviews and meta-analyses [19]. The design of the present work was fully specified in advance. It was registered in the PROSPERO database with the registration number CRD42017078688. Further details on the protocol can be found elsewhere [20]. This protocol was subject to minor amendments, agreed at a EUROMENE meeting. A description of the amendments can be found in Table A1. This was made publicly available before conducting the primary electronic search at http://www.euromene.eu/workinggroups/20190604protocol-amendments_prevalence-me-cfs.pdf.

#### 2.1.1. Inclusion Criteria

- Studies reporting either prevalence or incidence of ME/CFS, irrespective of age group, utilizing any of the following clinical diagnostic criteria: Centers for Disease Control & Prevention (CDC)−1994 [1], Canadian Consensus Criteria [21], London Criteria [22], International Consensus Criteria [2], or Institute of Medicine criteria [3].
- Studies from European countries; namely (by alphabetical order), Albania, Andorra, Armenia, Austria, Azerbaijan, Belarus, Belgium, Bosnia and Herzegovina, Bulgaria, Croatia, Cyprus, Czechia, Denmark, Estonia, Finland, France, Georgia, Germany, Greece, Hungary, Iceland, Ireland, Italy, Kazakhstan, Kosovo, Latvia, Liechtenstein, Lithuania, Luxembourg, Malta, Moldova, Monaco, Montenegro, the Netherlands, North Macedonia (formerly Macedonia), Norway, Poland, Portugal, Romania, Russia, San Marino, Serbia, Slovakia, Slovenia, Spain, Sweden, Switzerland, Turkey, Ukraine, the United Kingdom and Vatican City.
- Studies conducted in community or primary care settings.

#### 2.1.2. Exclusion Criteria

- Studies without primary data (e.g., reviews).
- Studies conducted in selected samples (e.g., post-infection, following vaccination or in high-risk population sub-groups such as war veterans).
- Studies based on self-reported diagnosis of ME/CFS.
- Studies with definitions inappropriate for the purposes of the present review (e.g., CFS-like illness or other clinical criteria, such as the Oxford criteria, due to lack of specificity [23]).
- Duplicate reports. When populations are overlapping, the study with the largest sample size was included.
- Studies published before 1994, when the first case definition of ME/CFS of those included in the present work was launched; namely, CDC−1994 [1].

No language restriction was applied.

### 2.2. Search Strategy for Identifying Relevant Studies

The search strategy consisted of two stages.

Firstly, a primary systematic literature search in three electronic databases was performed by two independent reviewers (F.E.-L. and K.M.) on 15 June 2019. The combination of search terms in each database was:

- Scopus: ({epidemiology} OR {prevalence} OR {incidence}) AND ({chronic fatigue syndrome} OR {myalgic encephalomyelitis} OR {CFS/ME})
- PubMed: (“Fatigue Syndrome, Chronic”(Mesh) AND ((“Incidence”(Mesh) OR “Epidemiology”(Mesh) OR “epidemiology” (Subheading)) OR “Prevalence”(Mesh) OR “Cross-Sectional Studies”(Mesh)))
- Web of Science: (“epidemiology” OR “prevalence” OR “incidence”) AND (“chronic fatigue syndrome” OR “myalgic encephalomyelitis” OR “CFS/ME” OR “ME/CFS”)

Secondly, a complementary search was conducted as follows (i) a backward (by checking reference lists) and forward (by checking citations) search of the works included in the present review (F.E.-L.) and (ii) grey literature was addressed by contacting – via email - all the members of EUROMENE and asking them to provide, if available, prevalence rates, incidence rates or both of ME/CFS, in their countries, according to national registers, publications in their own languages, or any other publicly accessible source (J.C.-M.).

### 2.3. Selection of Studies for Inclusion to the Review

Two independent researchers (F.E.-L. and K.M.) screened records retrieved by the electronic search by titles/abstracts or by full text of works, to identify potential studies and their suitability. When disagreements emerged, consensus was obtained through discussion.

### 2.4. Assessment of Methodological Quality and Reporting of Data

The methodological quality of the eligible studies was evaluated with the Joanna Briggs Institute-Checklist for Prevalence Studies [24]. Before applying it, six members of the research team (i.e., F.E.-L., L.N., J.A., S.S., M.M., and E.L.) developed an agreed appraisal of the tool. The reporting quality of the eligible studies was evaluated using the observational studies in epidemiology (STROBE) checklist [25]. Two researchers evaluated independently the methodology (i.e., F.E.-L. and I.J.B.) and the quality of reporting (i.e., F.E.-L. and X.W.-S.) of the selected studies. When disagreements emerged between these two researchers, consensus was obtained through discussion.

### 2.5. Data Extraction and Management

To manage the selected studies, we used the Mendeley Desktop. Two researchers (F.E.-L. and A.I.) compiled independently the reference for each study (authors and year of publication), country, total sample size (*n* and % of women), age range, setting (e.g., primary care), case definition (i.e., diagnosis criteria), prevalence and/or incidence rates, overall and stratified by gender and age group (where available). When disagreements emerged between these two researchers, consensus was obtained through discussion.

### 2.6. Data Synthesis and Analysis

We anticipated that studies reporting the prevalence and incidence of ME/CFS in different European countries would be scarce [20]. This was confirmed after data selection. We therefore decided to provide a narrative (descriptive) rather than quantitative synthesis.

## 3. Results

**Figure 1:**
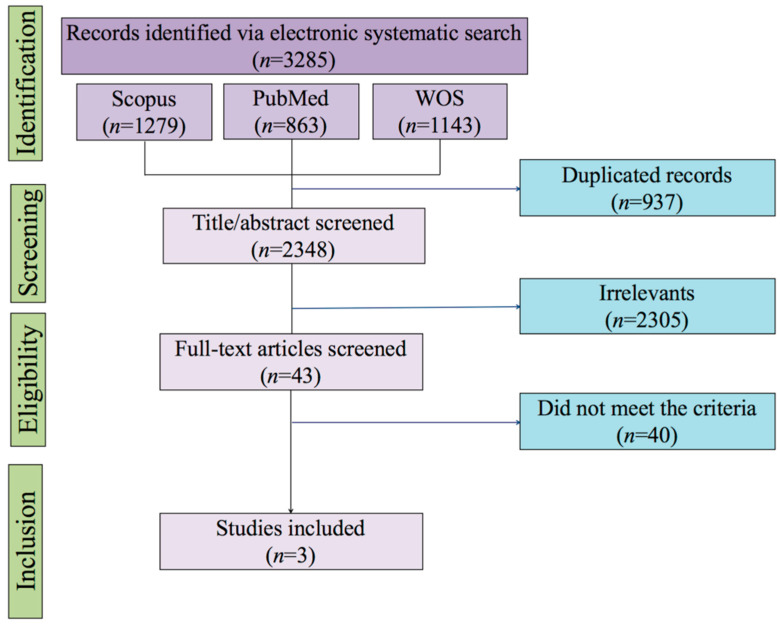
Flow diagram for study selection. Notes. WOS, web of science.

In line with the Open Science framework, and for the sake of transparency and reproducibility, the metadata downloaded (BibTex-Files) from Scopus, PubMed and Web of Science, and which were imported into the Mendeley Desktop, are available in file S1. Figure 1 shows that the (primary) systematic literature search in three electronic databases yielded 2348 studies after automatic identification and deletion of duplicates by Mendeley (for the full list of studies, see supplementary file S2), from which 43 studies were screened in full text; (see supplementary file S3 for the rationale to exclude 40 studies). Three studies were included for prevalence [13,26,27]. Two of these studies also reported incidence estimates of ME/CFS [26,27]. The complementary search did not yield any additional articles.

Table 1 and Table 2 show the characteristics of the included studies for prevalence and for incidence of ME/CFS, respectively, in European countries. Two of the studies were conducted in the United Kingdom [26,27] and the other in Iceland [13]. The sample sizes ranged from 842 [27] to 143,153 participants [26]. All studies used the CDC−1994 case definition [13,26,27] and one also used an additional case definition (namely, CCC−2003) in the same sample [26]. The target population in two studies was adults [13,26] while another study targeted adolescents [27]. The prevalence estimates ranged from 0.1% [27] to 2.2% [13]. In the two studies conducted in the United Kingdom, the estimated incidence rate was 15 cases per 100,000 adults per year [26] and 5 cases per 1000 adolescents per 6 months [27].

The methodology (Table 3) and the quality of reporting (Table A2) of the included studies were judged as good overall according to our appraisal tools. It should be noted that the prevalence estimates from Iceland had typographical errors in the original publication [13], as amended and communicated by the authors [28]. In this review, we used the amended figures [28].

## 4. Discussion

Only three papers on ME/CFS prevalence [13,26,27] in Europe were included in this review. Two of these studies were conducted in the United Kingdom [26,27]. The prevalence estimates from Europe appear to be in the same range as those from other continents [29,30]. The current review shows that in Europe only two studies have aimed at estimating the incidence of ME/CFS, one in adults [26] and the other in adolescents [27]. Previous systematic reviews on the epidemiological burden of ME/CFS did not investigate the incidence of ME/CFS [14,15,16,17]. Overall, as expected [20], studies on prevalence and incidence of ME/CFS in Europe were scarce.

Among the included studies in adults using the CDC−1994 case definition, prevalence estimates ranged from 0.2% [26] to 2.2% [13]. Previous systematic reviews found a similar range of prevalence estimates in other continents. For instance, in the United States [16], estimates of ME/CFS prevalence ranged from 0.2% [29] to 2.5% [30]. As suggested previously [26], these large variations in estimates may be a consequence of differences in methods, in inclusion/exclusion criteria, and in case definitions of ME/CFS. For instance, if in the present review a wider range of case definitions for ME/CFS were included, the maximum estimation among the included studies would have been a prevalence estimate of 7.8% in Iceland, using the Lloyd definition [13]. This reflects the necessity of using standardised case definition in epidemiological studies of ME/CFS. In comparison with prevalence, the study of incidence in adults was even scarcer, as is indicated by the inclusion of only one study in the present systematic review [26].

Only one of the studies included was conducted in young people [27]. The prevalence estimate was found to be 0.1% in adolescents from 11 to 15 years old, and the incidence was estimated at 5 new cases per 1000 adolescents per 6 months [27]. While previous reviews have focused on adults [14,17], it is important to highlight that children and adolescents are also significantly affected by ME/CFS. Indeed, case definitions of ME/CFS were first established for adults, and later extended to younger age groups, reflecting an initial lack of attention to the paediatric population. In our view, ME/CFS in younger individuals requires further investigation.

### 4.1. Implications

One of the studies included in the present review found that initial diagnoses of ME/CFS made by General Practitioners (GPs) were usually inaccurate, which impacts the estimation of the prevalence of ME/CFS [26]. Potential reasons for misdiagnosis include limited knowledge of or inability to recognise ME/CFS, and lack of access for patients with severe ME/CFS symptoms to GPs or other healthcare professionals [31]. In Europe, many primary care professionals rarely or never diagnose ME/CFS, and this could lead to potential disease misclassification. A possible way to overcome this problem is to offer training on ME/CFS diagnosis, and to support healthcare from primary care physicians, which may help to decrease the time to diagnosis, and therefore be beneficial to patients [32]. Cultural reasons may also explain this finding of misclassification [26]. A study of cultural differences that may be involved in the non-recognition of ME/CFS as a debilitating disease with high socio-economic impact in Europe would be highly desirable [9,33].

In the studies included in the present review, those conducted in adults independently used more than one case definition [13,26], while that in the child population did not do so [27]. Until one universal case definition is accepted in Europe, one approach of using several case definitions independently allows the performance of multiple comparisons (e.g., to stratify patients with ME/CFS) [17] and, thereby to provide a more comprehensive picture of the epidemiology of ME/CFS in Europe. Another approach is to use several case definitions sequentially, which has been done in a number of European clinical studies; e.g., [34,35,36]. However, this sequential approach has a number of important limitations for epidemiological purposes, further details of which have been provided elsewhere [17]. 

Currently, there is no accepted objective diagnostic test for ME/CFS (e.g., imaging or blood tests) but many case definitions are available (for a review, [17]). Therefore, the identification of acceptable objective markers of ME/CFS which can be utilised in epidemiological research in Europe is of very high priority. On this question, differences between ME/CFS cases and controls have been observed in the brain (structure, function, and metabolites), cognitive function and sleep function [37,38,39]. Associations between the occurrence of ME/CFS clinical symptoms, HHV−6, HHV−7 and B19 infection/co-infection reactivation, and increased expression levels of TNF-α and IL6 [40] have been observed, as well as alterations in the levels of infection markers of B19V [41] and EBV [42]. In addition, patients’ levels of muscular strength, as measured by the handgrip test, are related to the severity of ME/CFS [43]. Thus, it seems advisable that future epidemiological studies on ME/CFS should include objective clinical measurements in addition to patient-reported outcome measures, which should be summarised in the final reports, to improve the reliability and comparability of studies. In this context, when needed, it is important to adapt and validate questionnaires to languages other than English.

### 4.2. EUROMENE Research Recommendations for Epidemiology

The resources needed for epidemiological studies in ME/CFS tend to be costly, as, in the absence of specific biomarkers. As a result “caseness” must be ascertained by clinical history, symptomology and exclusion of other conditions. This approach requires clinicians and a battery of tests. Therefore, to determine the prevalence and incidence of ME/CFS in Europe, EUROMENE is making four overarching recommendations, as follows:

Firstly, research is best conducted by screening the community, instead of via primary care physicians, because this would help to minimise both selection and referral biases observed in clinical samples [26,27]. This screening may be done by means of the DePaul Symptom Questionnaire (DSQ) [44] or the United Kingdom ME/CFS Biobank Participant Questionnaire (UKMEBPQ) [45]. These two questionnaires were developed to enable comprehensive assessment of the signs of ME/CFS.

Secondly, if the study is conducted in community settings, we recommend that the clinical histories of potential cases identified in the community should be reviewed, in order to search for both key and additional ME/CFS symptoms. Key symptoms are persistent and include debilitation, exhaustion, post-exertional malaise, unrefreshing sleep, widespread musculoskeletal pain and cognitive dysfunction. Additional symptoms may involve a myriad of signs, including, but not limited to, orthostatic intolerance, over-sensitivity to stress or sensory stimuli, food intolerance, infection-immune like symptoms, symptoms of irritable bowel syndrome, weight loss or gain, symptoms of sicca, anxiety and depression. If the study is conducted in primary care settings, participating GPs should identify potential individuals with ME/CFS, using specific disease codes and excluding other diseases that could explain their symptoms. In the United Kingdom, for instance, as the National Health Service (NHS) has universal coverage and unified databases containing a wealth of data on the registered population, research in primary care can be considered population-based. Thus, missing cases and refusals can be accounted, by looking at the characteristics of population covered by participating GP practices. However, the GPs must be willing to participate in the research effort, which – despite being incentivised in the National Institute of Health Research (NIHR), does not always happen. For countries that have health care models based on mixed providers (i.e., private and public health care providers) or without common databases for patient information, this approach would be even more challenging.

Thirdly, when required, there is a need to obtain additional objective information by means of a clinical assessment that may involve a general examination (e.g., signs of anaemia, jaundice, and gross assessment of levels of hydration and nutritional status), a specific examination covering main body systems (e.g., heart and circulation) and a directed examination targeted according to general health history general clinical examination, findings, and specific symptoms which are reported (e.g., blood test for identifying rheumatoid factor).

Fourthly, to confirm ME/CFS cases, the CDC−1994 [1], Canadian Consensus Criteria [21], or Institute of Medicine criteria [3] criteria are found acceptable by EUROMENE. Additionally, the use of several independent case definitions is advisable because this may provide a more comprehensive picture of the epidemiology of ME/CFS in Europe and enhance comparability between studies. People whose symptoms may be mostly or largely explained by other conditions (e.g., cancer, post-traumatic stress disorder or rheumatoid arthritis) should not be identified as ME/CFS cases. When the presence of other conditions does not explain most of the symptoms and signs observed, they may be considered as comorbidities (e.g., fibromyalgia, irritable bowel syndrome, and sicca syndrome) [46].

EUROMENE acknowledges that meeting these four overarching recommendations is not always feasible due to limitations of economic and human resources, particularly in the context of a lack of funding for ME/CFS research. Indeed, it should be noted that, given the current paucity of knowledge of the prevalence and incidence of ME/CFS in Europe, less accurate estimations provided by more feasible research are still of interest. This is so as long as participants are not identified through subject self-diagnosis, or self-report of having been previously diagnosed without further corroboration by the research team. For instance, the alternative of asking potential ME/CFS cases to provide clinical reports of ME/CFS diagnosis could help to exclude alternative pathologies. When possible, and to harmonise research in Europe, ME/CFS cases are best identified by at least one of the following three case definitions: the CDC−1994 [1], Canadian Consensus Criteria [21], or Institute of Medicine criteria [3].

An ambitious approach to be considered is the development of a collaborative effort with concomitant studies using the same protocols, being either performed or developed in different European countries. In this way, the different factors related to the health care settings for each population could be compared and considered in the analyses. These include for example, population coverage, refusals, and missing cases – such as those with severe symptoms - who are less likely to attend health services and are usually underrepresented in research [47].

### 4.3. Limitations and Strengths

Although no language restrictions were applied, most of the retrieved works (93%) were published in English. All the members of EUROMENE were contacted to identify potential grey literature in their countries according to national registers, publications in their own languages, or any other publicly accessible source. Thus, though we are reasonably confident that all the information in other languages was retrieved, studies in languages other than English were not included. The small number of included studies may be considered as a limitation of the present review. It could be argued that including only studies using the CDC−1994 [1], Canadian Consensus Criteria [21], London Criteria [22], International Consensus Criteria [2], or Institute of Medicine criteria [3] case definitions was too restrictive. However, we find that this restrictive approach was a strength of the present study. For instance, including studies based on people’s self-report of having ME/CFS or with an inappropriate case definition (e.g., CFS-like illness or other clinical criteria, such as the Oxford criteria qhich lack of specificity) would have permitted inclusion of more studies, but would also have resulted in unreliable estimates. For instance, in a study conducted in Poland, from 1400 participants who identified themselves as suffering disabling fatigue, only 69 people met the CDC−1994 case definition of ME/CFS [48]. Additional strengths are the carefully designed search strategy, and the timespan coverage. Also, this review was conducted and reviewed by experienced researchers who are members of EUROMENE, which highlights the credibility and reliability of the findings.

## 5. Conclusions

As we anticipated, the present systematic review shows that research on the epidemiological burden of ME/CFS in Europe is scarce. Only three studies have estimated ME/CFS prevalence, while incidence has been estimated in two. Potential causes of this paucity of knowledge may be due to a European lack of (i) official disease recognition, (ii) consensus over case definitions, or (iii) investment by funding agencies, among others. By evidencing the paucity of epidemiological data on ME/CFS in Europe, our findings point to the pressing need for well-designed and statistically powered epidemiological studies. These are paramount requirements for informing and addressing the healthcare needs of people with ME/CFS, as well as for providing reliable information in order to ascertain the burden of disease for the European community, including its socio-economic impact. To overcome the shortcomings of the current state-of-the-art, EUROMENE recommends that future research is better conducted in the community, reviewing the clinical history of potential cases, obtaining additional objective information (when needed) and using adequate ME/CFS case definitions; namely, the CDC−1994 [1], Canadian Consensus Criteria [21], or Institute of Medicine criteria [3].

## Figures and Tables

**Table 1:** Characteristics of the included studies reporting point prevalence of ME/CFS in European countries.

Reference	Procedure	Settings, Country	Sample, Total (% Women)	Age Range	Case Definition	Prevalence Estimate % (95% CI)	Prevalence Estimate by Gender
Nacul et al., [26]	Electronic search (GPs databases), queries to GPs, clinical review of cases	Primary care, The United Kingdom	143,000 (51%)	Adults, 18 to 64 years old	CDC−1994 CCC−2003	0.20 (0.18 to 0.23) 0.10 (0.09 to 0.12)	Women = 0.31 (0.27 to 0.35) Men = 0.08 (0.06 to 0.10) Women = 0.18 (0.15 to 0.21) Men = 0.04 (0.03 to 0.06)
Lindal et al., [13]	Postal delivery to randomly selected people	Community, Iceland	2471 (57%)	Adults, 19 to 75 years old	CDC−1994	2.2 (not reported)	Women = 3.0% (not reported) Men = 1.1% (not reported)
Rimes et al., [27]	Random selection from the Child Benefit Register	Community, The United Kingdom	842 (not reported)	Adolescents, 11 to 15 years old	CDC−1994	0.1 (not reported)	Not reported

Notes. The design of all included studies was cross-sectional. Figures are presented as accurate (i.e., number of decimals) as reported in the original publication. CCC, Canadian Consensus Criteria; CDC, Centers for Disease Control and Prevention; CI, Confidence Interval; GPs, General Practitioners.

**Table 2:** Characteristics of the included studies reporting the incidence of ME/CFS in European countries.

Reference	Follow-up, Procedure	Settings, Country	Sample, Total (Women, %)	Age Range	Case Definition	Incidence Estimate	Incidence Estimate by Gender
Nacul et al., [26]	12 months, Electronic search (GPs databases), queries to GPs, clinical review of cases	Primary care, The United Kingdom	143,153 (51%)	Adults, 18 to 64 years old	CDC−1994 CCC−2003	15 new cases per 100,000 adults per year 5 new cases per 100,000 adults per year	Women = 23 new cases per 100,000 adults per year Men = 7 new cases per 100,000 adults per year Women = 6 new cases per 100,000 adults per year Men = 3 new cases per 100,000 adults per year
Rimes et al., [27]	4 to 6 months, random selection from the Child Benefit Register	Community, The United Kingdom	842 (not reported)	Adolescents, 11 to 15 years old	CDC−1994	5 new cases per 1000 adolescents per 6 months	Not reported

Notes. Figures are presented as accurate (i.e., number of decimals) as reported in the original publication. CCC, Canadian Consensus Criteria; CDC, Centers for Disease Control and Prevention; GP, General Practitioners.

**Table 3:** The methodological quality of the included studies evaluated by the Joanna Briggs Institute-Checklist for Prevalence Studies.

	Nacul et al., [26]	Lindal et al., [13]	Rimes et al., [27]
1. Appropriate sample frame	Yes	Yes	Yes
2. Participants were sampled appropriately	Yes	Yes	Yes
3. Adequate sample size	Yes	Yes	Yes
4. Participants and settings were well described	Yes	Yes	Yes
5. Data analysis with sufficient coverage	Yes	No/Unclear	Yes
6. Valid methods for identifying the condition	Yes	No/Unclear	Yes
7. Standard and reliable measure of the condition	Yes	Yes	Yes
8. Appropriate statistical analyses	Yes	Yes	Yes
9. Adequate response rate	Yes	Yes	No/Unclear

## Supplementary Materials

The following are available online at https://www.mdpi.com/2077-0383/9/5/1557/s1, File S1: Metadata, File S2: List of references after excluding duplicate reports, File S3: List of references excluding after screening the full-text.

## Appendix A

Table A1 shows amendments to the protocol of the present systematic review, and their rationale. These amendments were made publicly available before conducting the primary electronic search at http://www.euromene.eu/workinggroups/20190604protocol-amendments_prevalence-me-cfs.pdf.

**Table A1:** Amendments (and rationale) to the protocol of the present systematic review.

Original Protocol	Amendments (A) and Rationale (R)
Section: The primary systematic literature search on electronic databases. Text: Two independent reviewers (FE-L and JC-M) will perform a primary electronic search in PubMed, Scopus and Web of Science on 9 January 2018	A: The search will be updated to include works published up to 15 June 2019. R: Due to budget restrictions to cover the publication fee, it was decided to postpone the preparation of this work.
Section: Exclusion criteria Text: Studies published more than 10 years ago (i.e., before 2008).	A: Studies published before 1994. R: The decision lacked of a strong rationale and it was too restrictive. Given that studies that used the CDC−1994, Canadian Consensus Criteria, London Criteria, International Consensus Criteria or Institute of Medicine criteria will be considered, we will search for the literature that has been published from 1994, when the CDC−1994 were launched.

*Notes.* The original protocol is accessible in the following link: https://bmjopen.bmj.com/content/8/9/e020817.long. The table shows the amendments to the Protocol manuscript by Estévez-López et al. in BMJ Open.

We would like to apologise for any inconvenience, Fernando Estévez-López, on behalf of the EUROMENE working group on Epidemiology.

**Table A2:** The reporting quality of the included studies evaluated by the observational studies in epidemiology (STROBE) checklist.

	Nacul et al., [26]	Lindal et al., [13]	Rimes et al., [27]
Title and abstract			
1a. Indicate the study design	Yes	Yes	Yes
1b. Informative and balanced abstract	Yes	Yes	Yes
Introduction			
2. Background/rationale	Yes	Yes	Yes
3. Objectives	Yes	Yes	Yes
Methods			
4. Study design	Yes	No/Unclear	No/Unclear
5. Setting	Yes	Yes	No/Unclear
6a. Participants	Yes	Yes	Yes
7. Variables	Yes	Yes	Yes
8. Data sources/measurement	Yes	Yes	Yes
9. Bias	No/Unclear	No/Unclear	No/Unclear
10. Study size	Yes	No/Unclear	No/Unclear
12a. Statistics: description of all methods	Yes	Yes	Yes
12b. Statistics: subgroups and interactions	Yes	Yes	Yes
12c. Statistics: missing data	Yes	Yes	Yes
12d. Statistics: loss to follow-up	Yes	Not applicable	Yes
12e. Statistics: sensitivity analyses	Yes	No/Unclear	No/Unclear
Results			
13a. Participants: individual at each stage	Yes	Yes	Yes
13b. Participants: reasons for non-participation	Yes	No/Unclear	Yes
13c. Participants: flow diagram	Yes	No/Unclear	No/Unclear
14a. Descriptive data: characteristics of participants	Yes	Yes	Yes
14b. Descriptive data: missing data	Yes	Yes	Yes
14c. Descriptive data: follow-up	Yes	Not applicable	Yes
15. Outcome data	Yes	Yes	Yes
16a. Main results	Yes	Yes	Yes
Discussion			
18. Key results	Yes	Yes	Yes
19. Limitations	Yes	Yes	Yes
20. Interpretation	Yes	No/Unclear	No/Unclear
21. Generalisability	Yes	No/Unclear	No/Unclear
Other information			
22. Funding	No/Unclear	No/Unclear	No/Unclear

*Note*. The following items are not displayed in the figure as they were not applicable to the included studies: 6b, 11, 16b, 16c, 17.
