# Supplementary material for: Systematic Review of the Epidemiological Burden of Myalgic Encephalomyelitis/Chronic Fatigue Syndrome Across Europe: Current Evidence and EUROMENE Research Recommendations for Epidemiology
Source: J Clin Med. 2020 May 21;9(5):1557. doi: 10.3390/jcm9051557 (PMC7290765; doi:10.3390/jcm9051557)
Supplement: Supplementary file 1 [file jcm-09-01557-s001.zip › supplementary-files/jcm-784233-supplementary-file2.pdf]

List of references after excluding duplicate reports (n=2348)

1. [Anonymous] XMRV is not pathogenic for human and has no Significance for the safety of blood and blood products Opinions of the Working Group of the Federal Ministry for Blood Health. *BUNDESGESUNDHEITSBLATT-GESUNDHEITSFORSCHUNG-GESUNDHEITSSCHUTZ* **2012**, *55*, 1057–1060. Work in German
2. Aaron, L.A.; Burke, M.M.; Buchwald, D. Overlapping conditions among patients with chronic fatigue syndrome, fibromyalgia, and temporomandibular disorder. *Arch. Intern. Med.* **2000**, *160*, 221–227.
3. Aaron, L.A.; Herrell, R.; Ashton, S.; Belcourt, M.; Schmaling, K.; Goldberg, J.; Buchwald, D. Comorbid clinical conditions in chronic fatigue: a co-twin control study. *J. Gen. Intern. Med.* **2001**, *16*, 24–31.
4. Aaron, L.A.; Buchwald, D. Chronic diffuse musculoskeletal pain, fibromyalgia and co-morbid unexplained clinical conditions. *Best Pract. Res. Clin. Rheumatol.* **2003**, *17*, 563–574.
5. Abbey, S.E.; Garfinkel, P.E. Chronic fatigue syndrome and depression: Cause, effect, or covariate. *Rev. Infect. Dis.* **1991**, *13*, S73–S83.
6. Abbi, B.; Natelson, B.H. Is chronic fatigue syndrome the same illness as fibromyalgia: evaluating the `single syndrome' hypothesis. *QJM-AN Int. J. Med.* **2013**, *106*, 3–9.
7. Abdo, A.A. Health-related quality of life of Saudi hepatitis B and C patients. *Ann. SAUDI Med.* **2012**, *32*, 397–403.
8. Ablashi, D. V; Berneman, Z.N.; Kramarsky, B.; Asano, Y.; Choudhury, S.; Pearson, G.R. Human herpesvirus-7 (HHV-7). *In Vivo (Brooklyn).* **1994**, *8*, 549–554.
9. Ablashi, D. V; Berneman, Z.N.; Kramarsky, B.; Whitman Jr., J.; Asano, Y.; Pearson, G.R. Human herpesvirus-7 (HHV-7): current status. *Clin. Diagn. Virol.* **1995**, *4*, 1–13.
10. Ablashi, D. V; Josephs, S.F.; Buchbinder, A.; Hellman, K.; Nakamura, S.; Llana, T.; Lusso, P.; Kaplan, M.; Dahlberg, J.; Memon, S.; et al. Human B-lymphotropic virus (human herpesvirus-6). *J. Virol. Methods* **1988**, *21*, 29–48.
11. Ablin, J.N.; Zohar, A.H.; Zaraya-Blum, R.; Buskila, D. Distinctive personality profiles of fibromyalgia and chronic fatigue syndrome patients. *PeerJ* **2016**, *2016*.
12. Ablin, J.N.; Buskila, D.; Van Houdenhove, B.; Luyten, P.; Atzeni, F.; Sarzi-Puttini, P. Is fibromyalgia a discrete entity? *Autoimmun. Rev.* **2012**, *11*, 585–588.
13. Abma, F.I.; Amick III, B.C.; van der Klink, J.J.L.; Bultmann, U. Prognostic Factors for Successful Work Functioning in the General Working Population. *J. Occup. Rehabil.* **2013**, *23*, 162–169.
14. Abouassaly, R.; Fossa, S.D.; Giwercman, A.; Kollmannsberger, C.; Motzer, R.J.; Schmoll, H.-J.; Sternberg, C.N. Sequelae of treatment in long-term survivors of testis cancer. *Eur. Urol.* **2011**, *60*, 516–526.
15. Aboud, N.; Depre, F.; Salama, A. Is Autoimmune Thrombocytopenia Itself the Primary Disease in the Presence of Second Diseases? Data from a Long-Term Observation. *Transfus. Med. HEMOTHERAPY* **2017**, *44*, 23–28.
16. Adamczyk-Sowa, M.; Sowa, P.; Adamczyk, J.; Niedziela, N.; Misiolek, H.; Owczarek, M.; Zwirska-Korczala, K. Effect of melatonin supplementation on plasma lipid hydroperoxides, homocysteine concentration and chronic fatigue syndrome in multiple sclerosis patients treated with interferons-beta and mitoxantrone. *J. Physiol. Pharmacol.* **2016**, *67*, 235–242.

17. Adams, D.; Wu, T.; Yang, X.; Tai, S.; Vohra, S. Traditional Chinese medicinal herbs for the treatment of idiopathic chronic fatigue and chronic fatigue syndrome. *COCHRANE DATABASE Syst. Rev.* **2009**.
18. Adams, N.; Sim, J. An overview of fibromyalgia syndrome: Mechanisms, differential diagnosis and treatment approaches. *Physiotherapy* **1998**, *84*, 304–318.
19. Addington, A.M.; Gallo, J.J.; Ford, D.E.; Eaton, W.W. Epidemiology of unexplained fatigue and major depression in the community: the Baltimore ECA follow-up, 1981-1994. *Psychol. Med.* **2001**, *31*, 1037–1044.
20. Addington, J.W. Chronic fatigue syndrome: A dysfunction of the hypothalamic-pituitary-adrenal axis. *J. Chronic Fatigue Syndr.* **2000**, *7*, 63–74.
21. Adler, G.K.; Manfredsdottir, V.F.; Rackow, R.M. Hypothalamic-pituitary-adrenal axis function in fibromyalgia and chronic fatigue syndrome. *Endocrinologist* **2002**, *12*, 513–524.
22. Afari, N.; Ahumada, S.M.; Wright, L.J.; Mostoufi, S.; Golnari, G.; Reis, V.; Cuneo, J.G. Psychological trauma and functional somatic syndromes: A systematic review and meta-analysis. *Psychosom. Med.* **2014**, *76*, 2–11.
23. Afari, N.; Buchwald, D. Chronic fatigue syndrome: A review. *Am. J. Psychiatry* **2003**, *160*, 221–236.
24. Afari, N.; Eisenberg, D.M.; Herrell, R.; Goldberg, J.; Kleyman, E.; Ashton, S.; Buchwald, D. Use of alternative treatments by Chronic Fatigue Syndrome discordant twins. *Integr. Med.* **1999**, *2*, 97–103.
25. Afari, N.; Schmaling, K.B.; Herrell, R.; Hartman, S.; Goldberg, J.; Buchwald, D.S. Coping strategics in twins with chronic fatigue and chronic fatigue syndrome. *J. Psychosom. Res.* **2000**, *48*, 547–554.
26. Aftab, H.; Risstad, H.; Søvik, T.T.; Tomm Bernklev, P.D.; Hewitt, S.; Kristinsson, J.A.; Mala, T. Five-year outcome after gastric bypass for morbid obesity in a Norwegian cohort. *Surg. Obes. Relat. Dis.* **2014**, *10*, 71–78.
27. Aggarwal, V.R.; McBeth, J.; Zakrzewska, J.M.; Lunt, M.; Macfarlane, G.J. The epidemiology of chronic syndromes that are frequently unexplained: do they have common associated factors? *Int. J. Epidemiol.* **2006**, *35*, 468–476.
28. Agmon-Levin, N.; Zafrir, Y.; Kivity, S.; Balofsky, A.; Amital, H.; Shoenfeld, Y. Chronic fatigue syndrome and fibromyalgia following immunization with the hepatitis B vaccine: another angle of the `autoimmune (auto-inflammatory) syndrome induced by adjuvants' (ASIA). *Immunol. Res.* **2014**, *60*, 376–383.
29. Ahmed, S.; Lawrence, A. Anxiety and depression in fibromyalgia: Are we putting the cart before the horse? *Indian J. Rheumatol.* **2018**, *13*, 150–151.
30. Akguel, B.; Pfister, D.; Knuechel, R.; Heidenreich, A.; Wieland, U.; Pfister, H. No evidence for a role of xenotropic murine leukaemia virus-related virus and BK virus in prostate cancer of German patients. *Med. Microbiol. Immunol.* **2012**, *201*, 245–248.
31. Aktan, N.M. Chronic fatigue syndrome. An overview of current concepts. *Adv. Nurse Pract.* **2003**, *11*, 64–66.
32. Alarcia, R.; Ara, J.R.; Martin, J.; Lopez, A.; Bestue, M.; Bertol, V.; Vergara, J.M. Sleep disorders in multiple sclerosis. *NEUROLOGIA* **2004**, *19*, 704–709.
33. Alarcia, R.; Ara, J.R.; Martín, J.; López, A.; Bestué, M.; Bertol, V.; Vergara, J.M. Sleep disorders in multiple sclerosis [Trastornos del sueño en la esclerosis múltiple]. *Neurologia* **2004**, *19*, 704–709. Work in Spanish
34. Albayrak, I.; Yilmaz, H.; Akkurt, H.E.; Salli, A.; Karaca, G. Is pain the only symptom in patients with benign joint hypermobility syndrome? *Clin. Rheumatol.* **2015**, *34*, 1613–1619.

35. Albrecht, F. Chronic fatigue syndrome. *J. Am. Acad. Child Adolesc. Psychiatry* 2000, *39*, 808–809.
36. Albrecht, F.; Garralda, E.; Rangel, L. Chronic fatigue syndrome [2] (multiple letters). *J. Am. Acad. Child Adolesc. Psychiatry* **2000**, *39*, 808–809.
37. Albrecht, F.; Wallace, M. Detecting chronic fatigue syndrome: The role of counselors. *J. Couns. Dev.* **1998**, *76*, 183–188.
38. ALBRECHT, R.M.; OLIVER, V.L.; POSKANZER, D.C. EPIDEMIC NEUROMYASTHENIA. OUTBREAK IN A CONVENT IN NEW YORK STATE. *JAMA* **1964**, *187*, 904–907.
39. Alciati, A.; Sarzi-Puttini, P.; Batticciotto, A.; Torta, R.; Gesuele, F.; Atzeni, F.; Angst, J. Overactive lifestyle in patients with fibromyalgia as a core feature of bipolar spectrum disorder. *Clin. Exp. Rheumatol.* **2012**, *30*, S122–S128.
40. Alexander, M.E. Orthostasis, the autonomic nervous system, and abdominal pain in children: is there a relation between postural orthostatic tachycardia and recurrent abdominal pain? *J. Pediatr. Gastroenterol. Nutr.* **2008**, *47*, 696–700.
41. Alexander, S.; Minton, O.; Andrews, P.; Stone, P. A comparison of the characteristics of disease-free breast cancer survivors with or without cancer-related fatigue syndrome. *Eur. J. Cancer* **2009**, *45*, 384–392.
42. Ali, M.A.; Dale, J.K.; Kozak, C.A.; Goldbach-Mansky, R.; Miller, F.W.; Straus, S.E.; Cohen, J.I. Xenotropic murine leukemia virus-related virus is not associated with chronic fatigue syndrome in patients from different areas of the us in the 1990s. *Virol. J.* **2011**, *8*, 450.
43. Ali, S.; Goldsmith, K.; Burgess, M.; Chalder, T. Guided Self-Help for Patients with Chronic Fatigue Syndrome Prior to Starting Cognitive Behavioural Therapy: a Cohort Study. *Behav. Cogn. Psychother.* **2017**, *45*, 448–466.
44. Ali, S.; Matcham, F.; Irving, K.; Chalder, T. Fatigue and psychosocial variables in autoimmune rheumatic disease and chronic fatigue syndrome: A cross-sectional comparison. *J. Psychosom. Res.* **2017**, *92*, 1–8.
45. Alijotas, J.; Alegre, J.; Fernandez-Sola, J.; Cots, J.M.; Panisello, J.; Peri, J.M.; Pujol, R.; Cronic, G.T.S.F. Consensus report on the diagnosis and treatment of chronique fatigue syndrome in Catalonia, Spain. *Med. Clin. (Barc).* **2002**, *118*, 73–76.
46. Alisky, J.M.; Iczkowski, K.A.; Foti, A.A. Chronic fatigue syndrome. *Am. Fam. Physician* 1991, *44*, 56,61.
47. Allen, L.A.; Gara, M.A.; Escobar, J.I.; Waitzkin, H.; Silver, R.C. Somatization - A debilitating syndrome in primary care. *Psychosomatics* **2001**, *42*, 63–67.
48. Allen, P.R.; Rosati, P. Chronic fatigue syndrome: Implications for women and their health care providers during the childbearing years. *J. MIDWIFERY WOMENS Heal.* **2008**, *53*, 289–301.
49. Almenar-Perez, E.; Sanchez-Fito, T.; Ovejero, T.; Nathanson, L.; Oltra, E. Impact of Polypharmacy on Candidate Biomarker miRNomes for the Diagnosis of Fibromyalgia and Myalgic Encephalomyelitis/Chronic Fatigue Syndrome: Striking Back on Treatments. *Pharmaceutics* **2019**, *11*.
50. Aloisi, A.M.; Bonifazi, M. Sex hormones, central nervous system and pain. *Horm. Behav.* **2006**, *50*, 1–7.
51. Alter, H.J.; Mikovits, J.A.; Switzer, W.M.; Ruscetti, F.W.; Lo, S.-C.; Klimas, N.; Komaroff, A.L.; Montoya, J.G.; Bateman, L.; Levine, S.; et al. A Multicenter Blinded Analysis Indicates No Association between Chronic Fatigue Syndrome/Myalgic Encephalomyelitis and either Xenotropic Murine Leukemia Virus-Related Virus or Polytropic Murine Leukemia Virus. *MBio* **2012**, *3*.

52. Altman, D.; Iliadou, A.N.; Lundholm, C.; Milsom, I.; Pedersen, N.L. Somatic Comorbidity in Women with Overactive Bladder Syndrome. *J. Urol.* **2016**, *196*, 473–477.
53. Amato, M.P.; De Stefano, N. Longitudinal follow-up of "benign" multiple sclerosis at 20 years. *Neurology* 2007, *69*, 938; author reply 938-9.
54. AMBROGETTI, A.; OLSON, L.G. CONSIDERATION OF NARCOLEPSY IN THE DIFFERENTIAL-DIAGNOSIS OF CHRONIC FATIGUE SYNDROME. *Med. J. Aust.* **1994**, *160*, 426–429.
55. Ameratunga, R.; Gillis, D.; Gold, M.; Linneberg, A.; Elwood, J.M. Evidence Refuting the Existence of Autoimmune/Autoinflammatory Syndrome Induced by Adjuvants (ASIA). *J. Allergy Clin. Immunol. Pract.* **2017**, *5*, 1551–1555.e1.
56. Amin, M.M.; Belisova, Z.; Hossain, S.; Gold, M.S.; Broderick, J.E.; Gold, A.R. Inspiratory airflow dynamics during sleep in veterans with Gulf War illness: a controlled study. *SLEEP Breath.* **2011**, *15*, 333–339.
57. Amital, D.; Fostick, L.; Polliack, M.L.; Segev, S.; Zohar, J.; Rubinow, A.; Amital, H. Posttraumatic stress disorder, tenderness, and fibromyalgia syndrome: are they different entities? *J. Psychosom. Res.* **2006**, *61*, 663–669.
58. Amsterdam, J.D.; Shults, J.; Rutherford, N. Open-label study of s-citalopram therapy of chronic fatigue syndrome and co-morbid major depressive disorder. *Prog. Neuropsychopharmacol. Biol. Psychiatry* **2008**, *32*, 100–106.
59. Anand, P.; Sundaram, C.; Jhurani, S.; Kunnumakkara, A.B.; Aggarwal, B.B. Curcumin and cancer: An "old-age" disease with an "age-old" solution. *Cancer Lett.* **2008**, *267*, 133–164.
60. Anbu, A.T.; Cleary, A.G. Chronic fatigue syndrome/myalgic encephalopathy in children. *Paediatr. Child Health (Oxford).* **2009**, *19*, 84–89.
61. Andersen, A.B.; Law, I.; Ostrowski, S.R.; Lebech, A.M.; Hoyer-Hansen, G.; Hojgaard, L.; Gerstoft, J.; Ullum, H.; Kjaer, A. Self-reported fatigue common among optimally treated HIV patients: no correlation with cerebral FDG-PET scanning abnormalities. *Neuroimmunomodulation* **2006**, *13*, 69–75.
62. Andersen, M.M.; Permin, H.; Albrecht, F. Illness and disability in Danish Chronic Fatigue Syndrome patients at diagnosis and 5-year follow-up. *J. Psychosom. Res.* **2004**, *56*, 217–229.
63. Anderson, J.S.; Kayner, D. Milieu Issues in the Treatment of a Person With Chronic Fatigue Syndrome on an Inpatient Psychiatric Unit. *J. Am. Psychiatr. Nurses Assoc.* **1995**, *1*, 12–15.
64. Anderson, J.W.; Lambert, E.A.; Sari, C.I.; Dawood, T.; Esler, M.D.; Vaddadi, G.; Lambert, G.W. Cognitive function, health-related quality of life, and symptoms of depression and anxiety sensitivity are impaired in patients with the postural orthostatic tachycardia syndrome (POTS). *Front. Physiol.* **2014**, *5*.
65. Anderson, L.S.; Beverly, W.T.; Corey, L.A.; Murrelle, L. The Mid-Atlantic Twin Registry. *Twin Res.* **2002**, *5*, 449–455.
66. Anderson, S.J. On the importance of collecting local neuropsychological normative data. *SOUTH AFRICAN J. Psychol.* **2001**, *31*, 29–34.
67. Angst, J.; Gamma, A.; Sellaro, R.; Zhang, H.P.; Merikangas, K. Toward validation of atypical depression in the community: results of the Zurich cohort study. *J. Affect. Disord.* **2002**, *72*, 125–138.
68. Antcliff, D.; Campbell, M.; Woby, S.; Keeley, P. Activity Pacing is Associated With Better and Worse Symptoms for Patients With Long-term Conditions. *Clin. J. Pain* **2017**, *33*, 205–214.

69. Antcliff, D.; Campbell, M.; Woby, S.; Keeley, P. Assessing the Psychometric Properties of an Activity Pacing Questionnaire for Chronic Pain and Fatigue. *Phys. Ther.* **2015**, *95*, 1274–1286.
70. Anthony, K.K.; Schanberg, L.E. Pediatric pain syndromes and management of pain in children and adolescents with rheumatic disease. *Pediatr. Clin. NORTH Am.* **2005**, *52*, 611+.
71. Appel, S.; Chapman, J.; Shoenfeld, Y. Infection and vaccination in chronic fatigue syndrome: Myth or reality? *Autoimmunity* **2007**, *40*, 48–53.
72. Arias, M. Is fibromyalgia a neurological disease? *NEUROLOGIA* **2008**, *23*, 593–601.
73. Armon, C.; Kurland, L.T. Chronic fatigue syndrome: issues in the diagnosis and estimation of incidence. *Rev. Infect. Dis.* **1991**, *13 Suppl 1*, S68-72.
74. Arnett, P.A.; Barwick, F.H.; Beeney, J.E. Depression in multiple sclerosis: Review and theoretical proposal. *J. Int. Neuropsychol. Soc.* **2008**, *14*, 691–724.
75. Arnetz, B.B. Causes of change in the health of populations: A biopsychosocial viewpoint. *Soc. Sci. Med.* **1996**, *43*, 605–608. Proceedings of the XIVth International Conference on the Social Sciences and Medicine in Peebles, Scotland.
76. Arnold, L.D.; Bachmann, G.A.; Rosen, R.; Rhoads, G.G. Assessment of vulvodynia symptoms in a sample of US women: a prevalence survey with a nested case control study. *Am. J. Obstet. Gynecol.* **2007**, *196*, 128.e1-128.e6.
77. Arnold, L.M.; Wang, F.; Ahl, J.; Gaynor, P.J.; Wohlreich, M.M. Improvement in multiple dimensions of fatigue in patients with fibromyalgia treated with duloxetine: Secondary analysis of a randomized, placebo-controlled trial. *Arthritis Res. Ther.* **2011**, *13*.
78. Aronowitz, R.A. The trouble with chronic fatigue. *J. Gen. Intern. Med.* **1991**, *6*, 378–379.
79. Arora, D.; Kumar, M. Food allergies - Leads from Ayurveda. *Indian J. Med. Sci.* **2003**, *57*, 57–63.
80. Arpino, C.; Carrieri, M.P.; Valesini, G.; Pizzigallo, E.; Rovere, P.; Tirelli, U.; Conti, F.; Dialmi, P.; Barberio, A.; Rusconi, N.; et al. Idiopathic chronic fatigue and chronic fatigue syndrome: a comparison of two case-definitions. *Ann. Ist. Super. Sanita* **1999**, *35*, 435–441.
81. Arranz, L.-I.; Canela, M.-A.; Rafecas, M. Fibromyalgia and nutrition, what do we know? *Rheumatol. Int.* **2010**, *30*, 1417–1427.
82. Arredondo, M.; Hackett, J.J.; de Bethencourt, F.R.; Trevino, A.; Escudero, D.; Collado, A.; Qiu, X.; Swanson, P.; Soriano, V.; de Mendoza, C. Prevalence of xenotropic murine leukemia virus-related virus infection in different risk populations in Spain. *AIDS Res. Hum. Retroviruses* **2012**, *28*, 1089–1094.
83. Arzomand, M.L. Chronic fatigue syndrome among school children and their special educational needs. *J. Chronic Fatigue Syndr.* **1998**, *4*, 59–69.
84. Aslangul, E.; Le Jeunne, C. [Diagnosing asthenia and chronic fatigue syndrome]. *Rev. Prat.* **2005**, *55*, 1029–1033. Work in French
85. Assil, S.; Bolze, P.-A.; Boukhali, M.; Cariou, C.; Chauveau, L.; Chuvin, N.; Dhondt, K.; Ducuing, A.; Dupont, J.-B.; Grandin, C.; et al. XMRV human retrovirus: the end of an appealing story? *Virologie* **2011**, *15*, 222–234.
86. Assumpcao, A.; Cavalcante, A.B.; Capela, C.E.; Sauer, J.F.; Chalot, S.D.; Pereira, C.A.B.; Marques, A.P.; Assumpção, A.; Cavalcante, A.B.; Capela, C.E.; et al. Prevalence of fibromyalgia in a low socioeconomic status population. *BMC Musculoskelet. Disord.* **2009**, *10*, 64.

87. Astudillo, L.; Laure, A.; Fabry, V.; Pugnet, G.; Maury, P.; Labrunee, M.; Sailler, L.; Traon, A.P.-L. Postural tachycardia syndrome (PoTS): An up-to-date. *Rev. Med. INTERNE* **2018**, *39*, 627–634.
88. Atasever, M.; Kalem, M.N.; Sonmez, C.; Seval, M.M.; Yuce, T.; Aker, S.S.; Koc, A.; Genc, H. Lower serotonin level and higher rate of fibromyalgia syndrome with advancing pregnancy. *J. Matern. NEONATAL Med.* **2017**, *30*, 2204–2211.
89. Auquier, L.; Bontoux, D.; Löo, H.; Godeau, P.; Menkès, C.-J.; Paolaggi, J.-B.; Perrot, S. Fibromyalgia [La fibromyalgie]. *Rev. Med. Interne* **2008**, *29*, 161–168. Work in French
90. Authier, F.J.; Gherardi, R.K. Muscular complications of human immunodeficiency virus (HIV) infection in the era of effective anti-retroviral therapy [Complications musculaires de l'infection par le virus de l'immunodéficience humaine (VIH) à l'ère des trithérapies]. *Rev. Neurol. (Paris).* **2006**, *162*, 71–81. Work in French
91. Authier, F.-J.; Chariot, P.; Gherardi, R.K. Skeletal muscle involvement in human immunodeficiency virus (HIV)-infected patients in the era of highly active antiretroviral therapy (HAART). *Muscle and Nerve* **2005**, *32*, 247–260.
92. Authier, F.-J.; Sauvat, S.; Champey, J.; Drogou, I.; Coquet, M.; Gherardi, R.K. Chronic fatigue syndrome in patients with macrophagic myofasciitis. *Arthritis Rheum.* **2003**, *48*, 569–570.
93. Auwaerter, P.G. Point: Antibiotic therapy is not the answer for patients with persisting symptoms attributable to Lyme disease. *Clin. Infect. Dis.* **2007**, *45*, 143–148. Proceedings of the 44th Annual Meeting of the Infectious-Diseases-Society-of-America inToronto, Canada.
94. Avellaneda Fernández, A.; Pérez Martín, Á.; Izquierdo Martínez, M. Chronic fatigue syndrome. Consensus document [Síndrome de fatiga crónica. Documento de consenso]. *Aten. Primaria* **2009**, *41*, 529–531. Work in Spanish
95. Avellaneda Fernández, A.; Pérez Martín, Á.; Izquierdo Martínez, M.; Arruti Bustillo, M.; Barbado Hernández, F.J.; De La Cruz Labrado, J.; Díaz-Delgado Pẽas, R.; Gutiérrez Rivas, E.; Palacín Delgado, C.; Ramón Giménez, J.R.; et al. Chronic fatigue syndrome. Summary of the consensus document [Síndrome de fatiga crónica. Resumen del documento de consenso]. *Aten. Primaria* **2009**, *41*, e1–e5. Work in Spanish
96. Avellaneda Fernandez, A.; Perez Martin, A.; Izquierdo Martinez, M.; Arruti Bustillo, M.; Barbado Hernandez, F.J.; de la Cruz Labrado, J.; Diaz-Delgado Penas, R.; Gutierrez Rivas, E.; Palacin Delgado, C.; Rivera Redondo, J.; et al. Chronic fatigue syndrome: aetiology, diagnosis and treatment. *BMC Psychiatry* **2009**, *9*.
97. Ayres, J.G.; Flint, N.; Smith, E.G.; Tunnicliffe, W.S.; Fletcher, T.J.; Hammond, K.; Ward, D.; Marmion, B.P. Post-infection fatigue syndrome following Q fever. *QJM* **1998**, *91*, 105–123.
98. Ayres, J.G.; Smith, E.G.; Flint, N. Protracted fatigue and debility after acute Q fever. *Lancet (London, England)* 1996, *347*, 978–979.
99. Ayres, J.G.; Wildman, M.; Groves, J.; Ment, J.; Smith, E.G.; Beattie, J.M. Long-term follow-up of patients from the 1989 Q fever outbreak: no evidence of excess cardiac disease in those with fatigue. *QJM* **2002**, *95*, 539–546.
100. Azpiroz, F.; Dapoigny, M.; Pace, F.; Muller-Lissner, S.; Coremans, G.; Whorwell, P.; Stockbrugger, R.W.; Smout, A. Nongastrointestinal disorders in the irritable bowel syndrome. *Digestion* **2000**, *62*, 66–72.

101. Bae, J.; Lin, J.-M.S. Healthcare Utilization in Myalgic Encephalomyelitis/Chronic Fatigue Syndrome (ME/CFS): Analysis of US Ambulatory Healthcare Data, 2000-2009. *Front. Pediatr.* **2019**, *7*.
102. Baert, I.A.C.; Lluch, E.; Van Glabbeek, F.; Nuyts, R.; Rufai, S.; Tuynman, J.; Struyf, F.; Meeus, M. Short stem total hip arthroplasty: Potential explanations for persistent post-surgical thigh pain. *Med. Hypotheses* **2017**, *107*, 45–50.
103. Baetz, M.; Bowen, R. Chronic pain and fatigue: Associations with religion and spirituality. *Pain Res. Manag.* **2008**, *13*, 383–388.
104. Bai, A.; Guo, Y.; Shen, Y.; Xie, Y.; Lu, N. Gender-Related and City- and Countryside-Related Differences in Patients with Ulcerative Colitis in a Chinese Population. *Intern. Med.* **2008**, *47*, 2103–2107.
105. Baidina, T. V; Akintseva, Y. V; Trushnikova, T.N. A chronic fatigue syndrome and blood platelet serotonin levels in patients with multiple sclerosis. *Zhurnal Nevrol. i Psihiatr. Im. S.S. Korsakova* **2014**, *2014*, 25–28.
106. Bains, W. Treating Chronic Fatigue states as a disease of the regulation of energy metabolism. *Med. Hypotheses* **2008**, *71*, 481–488.
107. Baird, W.L.M.; Morgan, M. Substance misuse amongst anaesthetists. *Anaesthesia* **2000**, *55*, 943–945.
108. Baker, A.J.L.; Maiorino, E. Assessments of emotional abuse and neglect with the CTQ: Issues and estimates. *Child. Youth Serv. Rev.* **2010**, *32*, 740–748.
109. Bakken, I.J.; Tveito, K.; Aaberg, K.M.; Ghaderi, S.; Gunnes, N.; Trogstad, L.; Magnus, P.; Stoltenberg, C.; Haberg, S.E. Comorbidities treated in primary care in children with chronic fatigue syndrome / myalgic encephalomyelitis: A nationwide registry linkage study from Norway. *BMC Fam. Pract.* **2016**, *17*, 128.
110. Bakken, I.J.; Tveito, K.; Gunnes, N.; Ghaderi, S.; Stoltenberg, C.; Trogstad, L.; Haberg, S.E.; Magnus, P. Two age peaks in the incidence of chronic fatigue syndrome/myalgic encephalomyelitis: a population-based registry study from Norway 2008-2012. *BMC Med.* **2014**, *12*, 167.
111. Bakker, R.J. Unexplained chronic fatigue [Onverklaarde aanhoudende moeheidsklachten]. *Tijdschr. Kindergeneeskd.* **2005**, *73*, 104–108. Work in Dutch
112. Bakker, R.J.; Van De Putte, E.M.; Kuis, W.; Sinnema, G. Effects of an educational video film in fatigued children and adolescents: A randomised controlled trial. *Arch. Dis. Child.* **2011**, *96*, 457–460.
113. BALACHANDRAN, N.; TIRAWATNAPONG, S.; PFEIFFER, B.; ABLASHI, D. V; SALAHUDDIN, S.Z. ELECTROPHORETIC ANALYSIS OF HUMAN HERPESVIRUS-6 POLYPEPTIDES IMMUNOPRECIPITATED FROM INFECTED-CELLS WITH HUMAN SERA. *J. Infect. Dis.* **1991**, *163*, 29–34.
114. Balada, E.; Castro-Marrero, J.; Felip, L.; Vilardell-Tarres, M.; Ordi-Ros, J. Xenotropic Murine Leukemia Virus-Related Virus (XMRV) in Patients with Systemic Lupus Erythematosus. *J. Clin. Immunol.* **2011**, *31*, 584–587.
115. Balasubramaniam, R.; de Leeuw, R.; Zhu, H.; Nickerson, R.B.; Okeson, J.P.; Carlson, C.R. Prevalence of temporomandibular disorders in fibromyalgia and failed back syndrome patients: A blinded prospective comparison study. *ORAL Surg. ORAL Med. ORAL Pathol. ORAL Radiol. ENDODONTOLOGY* **2007**, *104*, 204–216.
116. Balasubramaniam, R.; Laudenbach, J.M.; Stoopler, E.T. Fibromyalgia: an update for oral health care providers. *ORAL Surg. ORAL Med. ORAL Pathol. ORAL Radiol. ENDODONTOLOGY* **2007**, *104*, 589–602.
117. Baldwin, C.M.; Bell, I.R. Increased cardiopulmonary disease risk in a community-based sample with chemical odor intolerance: Implications for women's health and

health-care utilization. *Arch. Environ. Health* **1998**, *53*, 347–353. Proceedings of the 6th Annual Meeting of the Society-of-Behavioral-Medicine in San Diego, USA.
118. Baldwin, C.M.; Bell, I.R.; Guerra, S.; Quan, S.F. Associations between chemical odor intolerance and sleep disturbances in community-living adults. *Sleep Med.* **2004**, *5*, 53–59. Proceedings of the 12th Annual Scientific Meeting of the Association-of-Professional-Sleep-Societies in Chicago, USA.
119. Balkarli, A.; Sengul, C.; Tepeli, E.; Balkarli, H.; Cobankara, V. Synaptosomal-associated protein 25 (Snap-25) gene Polymorphism frequency in fibromyalgia syndrome and relationship with clinical symptoms. *BMC Musculoskelet. Disord.* **2014**, *15*.
120. Ballantyne, A.J.; Rogers, W.A. Sex bias in studies selected for clinical guidelines. *J. Women's Heal.* **2011**, *20*, 1297–1306.
121. Band, R.; Barrowclough, C.; Caldwell, K.; Emsley, R.; Wearden, A. Activity patterns in response to symptoms in patients being treated for chronic fatigue syndrome: An experience sampling methodology study. *Health Psychol.* **2017**, *36*, 264–269.
122. Bandelow, B. Generalized anxiety disorder and pain. *Mod. Trends Pharmacopsychiatry* **2015**, *30*, 153–165.
123. Bankier, B.; Aigner, M.; Bach, M. Clinical validity of ICD-10 neurasthenia. *Psychopathology* **2001**, *34*, 134–139.
124. Banks, J.; Prior, L. Doing things with illness - The micro politics of the CFS clinic. *Soc. Sci. Med.* **2001**, *52*, 11–23.
125. Bannert, N. Is a novel human retrovirus associated with prostate cancer and chronic fatigue syndrome? *Future Microbiol.* **2010**, *5*, 689–691.
126. Bansal, A.S.; Bradley, A.S.; Bishop, K.N.; Kiani-Alikhan, S.; Ford, B. Chronic fatigue syndrome, the immune system and viral infection. *Brain. Behav. Immun.* **2012**, *26*, 24–31.
127. Barah, F.; Whiteside, S.; Batista, S.; Morris, J. Neurological aspects of human parvovirus B19 infection: A systematic review. *Rev. Med. Virol.* **2014**, *24*, 154–168.
128. Baraniuk, J.N. Xenotropic murine leukemia virus-related virus in chronic fatigue syndrome and prostate cancer. *Curr. Allergy Asthma Rep.* **2010**, *10*, 210–214.
129. Baraniuk, J.N.; Clauw, D.J.; Gaumond, E. Rhinitis symptoms in chronic fatigue syndrome. *Ann. Allergy, Asthma Immunol.* **1998**, *81*, 359–365.
130. Baraniuk, J.N.; Maibach, H. Pathophysiological classification of chronic rhinosinusitis. *Respir. Res.* **2005**, *6*.
131. Baraniuk, J.N.; Naranch, K.; Maibach, H.; Clauw, D.J. Tobacco sensitivity in Chronic Fatigue Syndrome (CFS). *J. Chronic Fatigue Syndr.* **2000**, *7*, 33–52.
132. Baraniuk, J.N.; Zheng, Y. Relationships among rhinitis, fibromyalgia, and chronic fatigue. *Allergy Asthma Proc.* **2010**, *31*, 169–178.
133. Baraniuk, J.N. Chronic fatigue syndrome prevalence is grossly overestimated using Oxford criteria compared to Centers for Disease Control (Fukuda) criteria in a U.S. population study. *FATIGUE-BIOMEDICINE Heal. Behav.* **2017**, *5*, 215–230.
134. Baraniuk, J.N.; Adewuyi, O.; Merck, S.J.; Ali, M.; Ravindran, M.K.; Timbol, C.R.; Ray-Han, R.; Zheng, Y.; Le, U.; Esteitie, R.; et al. A Chronic Fatigue Syndrome (CFS) severity score based on case designation criteria. *Am. J. Transl. Res.* **2013**, *5*, 53–68.

135. Barbour, A.G. “Lyme”: Chronic Fatigue Syndrome by Another Name? *Clin. Infect. Dis.* 2016, *62*, 134–135.
136. Barnden, L.R.; Kwiatek, R.; Crouch, B.; Burnet, R.; Del Fante, P. Autonomic correlations with MRI are abnormal in the brainstem vasomotor centre in Chronic Fatigue Syndrome. *NeuroImage. Clin.* **2016**, *11*, 530–537.
137. Barnes, E.; Flanagan, P.; Brown, A.; Robinson, N.; Brown, H.; McClure, M.; Oxenius, A.; Collier, J.; Weber, J.; Günthard, H.F.; et al. Failure to detect xenotropic murine leukemia virus-related virus in blood of individuals at high risk of blood-borne viral infections. *J. Infect. Dis.* **2010**, *202*, 1482–1485.
138. Barrett, D.H.; Gray, G.C.; Doebbeling, B.N.; Clauw, D.J.; Reeves, W.C. Prevalence of symptoms and symptom-based conditions among Gulf War veterans: Current status of research findings. *Epidemiol. Rev.* **2002**, *24*, 218–227.
139. Barron, D.F.; Cohen, B.A.; Geraghty, M.T.; Violand, R.; Rowe, P.C. Joint hypermobility is more common in children with chronic fatigue syndrome than in healthy controls. *J. Pediatr.* **2002**, *141*, 421–425.
140. Barrows, D.M. Functional capacity evaluations of persons with chronic fatigue immune dysfunction syndrome. *Am. J. Occup. Ther. Off. Publ. Am. Occup. Ther. Assoc.* **1995**, *49*, 327–337.
141. Barton, J.C.; Bertoli, L.F.; Barton, J.C.; Acton, R.T. Fibromyalgia in 300 adult index patients with primary immunodeficiency. *Clin. Exp. Rheumatol.* **2017**, *35 Suppl 105*, 68–73.
142. Barun, B. Pathophysiological background and clinical characteristics of sleep disorders in multiple sclerosis. *Clin. Neurol. Neurosurg.* **2013**, *115*, S82–S85.
143. Basch, M.C.; Chow, E.T.; Logan, D.E.; Schechter, N.L.; Simons, L.E. Perspectives on the clinical significance of functional pain syndromes in children. *J. Pain Res.* **2015**, *8*, 675–686.
144. Bass, C.; Peveler, R.; House, A. Somatoform disorders: severe psychiatric illnesses neglected by psychiatrists. *Br. J. PSYCHIATRY* **2001**, *179*, 11–14.
145. Basseri, B.; Yamini, D.; Chee, G.; Enayati, P.D.P.; Tran, T.; Poordad, F. Comorbidities associated with the increasing burden of hepatitis C infection. *Liver Int.* **2010**, *30*, 1012–1018.
146. Bateman, L.; Darakjy, S.; Klimas, N.; Peterson, D.; Levine, S.M.; Allen, A.; Carlson, S.A.; Balbin, E.; Gottschalk, G.; March, D. Chronic fatigue syndrome and co-morbid and consequent conditions: evidence from a multi-site clinical epidemiology study. *FATIGUE-BIOMEDICINE Heal. Behav.* **2015**, *3*, 1–15.
147. Bates, D.W.; Buchwald, D.; Lee, J.; Kith, P.; Doolittle, T.H.; Umali, P.; Komaroff, A.L. A comparison of case definitions of chronic fatigue syndrome. *Clin. Infect. Dis.* **1994**, *18 Suppl 1*, S11-5.
148. Bates, D.W.; Schmitt, W.; Buchwald, D.; Ware, N.C.; Lee, J.; Thoyer, E.; Kornish, R.J.; Komaroff, A.L. Prevalence of fatigue and chronic fatigue syndrome in a primary care practice. *Arch. Intern. Med.* **1993**, *153*, 2759–2765.
149. BATES, D.W.; SCHMITT, W.; LEE, J.; KORNISH, R.J.; KOMAROFF, A.L. PREVALENCE OF FATIGUE AND CHRONIC FATIGUE SYNDROME IN A PRIMARY CARE PRACTICE. *Clin. Res.* **1991**, *39*, A571.
150. Bates, M.N. Mercury amalgam dental fillings: An epidemiologic assessment. *Int. J. Hyg. Environ. Health* **2006**, *209*, 309–316.
151. Bates, M.N.; Fawcett, J.; Garrett, N.; Cutress, T.; Kjellstrom, T. Health effects of dental amalgam exposure: a retrospective cohort study. *Int. J. Epidemiol.* **2004**, *33*, 894–902.

152. Bauer, A.E.; Olivas, S.; Cooper, M.; Hornstra, H.; Keim, P.; Pearson, T.; Johnson, A.J. Estimated herd prevalence and sequence types of Coxiella burnetii in bulk tank milk samples from commercial dairies in Indiana. *BMC Vet. Res.* **2015**, *11*.
153. Baumer, J.H. Management of chronic fatigue syndrome/myalgic encephalopathy (CFS/ME). *Arch. Dis. Child. Educ. Pract. Ed.* **2005**, *90*, ep46-ep50.
154. Bayliss, K.; Riste, L.; Band, R.; Peters, S.; Wearden, A.; Lovell, K.; Fisher, L.; Chew-Graham, C.A. Implementing resources to support the diagnosis and management of Chronic Fatigue Syndrome/Myalgic Encephalomyelitis (CFS/ME) in primary care: A qualitative study. *BMC Fam. Pract.* **2016**, *17*.
155. Bayliss, K.; Riste, L.; Fisher, L.; Wearden, A.; Peters, S.; Lovell, K.; Chew-Graham, C. Diagnosis and management of chronic fatigue syndrome/myalgic encephalitis in black and minority ethnic people: a qualitative study. *Prim. Health Care Res. Dev.* **2014**, *15*, 143–155.
156. Bazelmans, E.; Vercoulen, J.H.; Galama, J.M.; van Weel, C.; van der Meer, J.W.; Bleijenberg, G. [Prevalence of chronic fatigue syndrome and primary fibromyalgia syndrome in The Netherlands]. *Ned. Tijdschr. Geneeskd.* **1997**, *141*, 1520–1523. Work in Dutch
157. Bazelmans, E.; Vercoulen, J.H.; Swanink, C.M.; Fennis, J.F.; Galama, J.M.; van Weel, C.; van der Meer, J.W.; Bleijenberg, G. Chronic Fatigue Syndrome and Primary Fibromyalgia Syndrome as recognized by GPs. *Fam. Pract.* **1999**, *16*, 602–604.
158. Bazelmans, E.; Vercoulen, J.H.M.M.; Galama, J.M.D.; Van Weel, C.; Van Der Meer, J.W.M.; Bleijenberg, G. Prevalence of chronic fatigue syndrome and primary fibromyalgia syndrome (PFS) in the Netherlands [Prevalentie van het chronische-vermoeidheidsyndroom en het primaire-fibromyalgiesyndroom in Nederland]. *Ned. Tijdschr. Geneeskd.* **1997**, *141*, 1520–1523. Work in Dutch
159. Beadle, A. Ciguatera fish poisoning. *Mil. Med.* **1997**, *162*, 319–322.
160. Beauchesne, P.; Blonski, M.; Brissart, H. Response to Intrathecal Infusions of Depocyt (R) in Secondary Diffuse Leptomeningeal Gliomatosis. A Case Report. *In Vivo (Brooklyn).* **2011**, *25*, 991–1025.
161. Bechter, K. Psychiatric differential aspects of pain. *Acta Clin. Croat.* **2010**, *49*, 82–84.
162. Begg, C.B.; Pike, M.C. Comment on "The predictive capacity of personal genome sequencing." *Sci. Transl. Med.* **2012**, *4*.
163. Behan, P.O. Postviral neurological syndromes. *Br. Med. J.* **1983**, *287*, 853–854.
164. Behan, P.O. Epidemic myalgic encephalomyelitis. *Practitioner* **1980**, *224*, 805–807.
165. Beiske, A.G.; Svensson, E. Fatigue in Parkinson's disease: a short update. *Acta Neurol. Scand. Suppl.* **2010**, 78–81.
166. Belgamwar, R.B.; Jorsh, M.S.; Knisely-Marpole, A.; Snowden, H.; Mayall, E.; Singhal, A.; Jones, J.M. Multidisciplinary group treatment for chronic fatigue syndrome. *Prog. Neurol. Psychiatry* **2009**, *13*, 27–29.
167. Bell, E.J.; McCartney, R.A. A study of Coxsackie B virus infections, 1972-1983. *J. Hyg. (Lond).* **1984**, *93*, 197–203.
168. Bell, I.R.; Walsh, M.E.; Goss, A.; Gersmeyer, J.; Schwartz, G.E.; Kanof, P. Cognitive dysfunction and disability in geriatric veterans with self- reported intolerance to environmental chemicals. *J. Chronic Fatigue Syndr.* **1997**, *3*, 15–42.

169. Bell, I.R.; Warg-Damiani, L.; Baldwin, C.M.; Walsh, M.E.; Schwartz, G.E.R. Self-reported chemical sensitivity and wartime chemical exposures in gulf war veterans with and without decreased global health ratings. *Mil. Med.* **1998**, *163*, 725–732.
170. Bell, K.M.; Cookfair, D.; Bell, D.S.; Reese, P.; Cooper, L. Risk factors associated with chronic fatigue syndrome in a cluster of pediatric cases. *Rev. Infect. Dis.* **1991**, *13 Suppl 1*, S32-8.
171. Bellanti, J.A. [Chronic fatigue syndrome]. *Rev. Alerg. Mex.* 1994, *41*, 65–68. Work in Spanish
172. Benca, R.M.; Ancoli-Israel, S.; Moldofsky, H. Special considerations in insomnia diagnosis and management: depressed, elderly, and chronic pain populations. *J. Clin. Psychiatry* **2004**, *65 Suppl 8*, 26–35.
173. Bennett, B.K.; Goldstein, D.; Chen, M.; Davenport, T.A.; Vollmer-Conna, U.; Scott, E.M.; Hickie, I.B.; Lloyd, A.R. Characterization of fatigue states in medicine and psychiatry by structured interview. *Psychosom. Med.* **2014**, *76*, 379–388.
174. Bennett, R. Fibromyalgia, chronic fatigue syndrome, and myofascial pain. *Curr. Opin. Rheumatol.* **1998**, *10*, 95–103.
175. Bennett, R.M.; Russell, J.; Cappelleri, J.C.; Bushmakin, A.G.; Zlateva, G.; Sadosky, A. Identification of symptom and functional domains that fibromyalgia patients would like to see improved: a cluster analysis. *BMC Musculoskelet. Disord.* **2010**, *11*, 134.
176. Berende, A.; ter Hofstede, H.J.M.; Donders, A.R.T.; van Middendorp, H.; Kessels, R.P.C.; Adang, E.M.M.; Vos, F.J.; Evers, A.W.M.; Kullberg, B.J. Persistent Lyme Empiric Antibiotic Study Europe (PLEASE) - design of a randomized controlled trial of prolonged antibiotic treatment in patients with persistent symptoms attributed to Lyme borreliosis. *BMC Infect. Dis.* **2014**, *14*.
177. Berg, A.M.; Naides, S.J.; Simms, R.W. Established fibromyalgia syndrome and parvovirus B19 infection. *J. Rheumatol.* **1993**, *20*, 1941–1943.
178. Berger, J.R.; Pocoski, J.; Preblick, R.; Boklage, S. Fatigue heralding multiple sclerosis. *Mult. Scler. J.* **2013**, *19*, 1526–1532.
179. Berkovitz, S.; Ambler, G.; Jenkins, M.; Thurgood, S. Serum 25-hydroxy vitamin D levels in chronic fatigue syndrome: a retrospective survey. *Int. J. Vitam. Nutr. Res.* **2009**, *79*, 250–254.
180. Berlin, B. Confronting AIDS in older adults. *N. J. Med.* **1997**, *94*, 39–41.
181. Bernabeu-Mestre, J.; Santos, A.P.C.; Pellicer, J.X.E.; Galiana-Sanchez, M.E. [Diagnostic categories and gender: two examples in contemporary Spanish medicine, chlorosis and neurasthenia (1877-1936)]. *Asclepio.* **2008**, *60*, 83–102. Work in Spanish
182. Bernatsky, S.; Dobkin, P.L.; De Civita, M.; Penrod, J.R. Comorbidity and physician use in fibromyalgia. *Swiss Med. Wkly.* **2005**, *135*, 76–81.
183. Bertoli, E.; de Leeuw, R.; Schmidt, J.E.; Okeson, J.P.; Carlson, C.R. Prevalence and impact of post-traumatic stress disorder symptoms in patients with masticatory muscle or temporomandibular joint pain: Differences and similarities. *J. Orofac. Pain* **2007**, *21*, 107–119.
184. Bertolin, J.M.; Calvo, J. [Chronic fatigue syndrome. To be or not to be?]. *Med. Clin. (Barc).* 1997, *108*, 577–579. Work in Spanish
185. Bested, A.C.; Marshall, L.M. Review of Myalgic Encephalomyelitis/Chronic Fatigue Syndrome: an evidence-based approach to diagnosis and management by clinicians. *Rev. Environ. Health* **2015**, *30*, 223–249.

186. Bhui, K.S.; Dinos, S.; Ashby, D.; Nazroo, J.; Wessely, S.; White, P.D. Chronic fatigue syndrome in an ethnically diverse population: the influence of psychosocial adversity and physical inactivity. *BMC Med.* **2011**, *9*, 26.
187. Bierl, C.; Nisenbaum, R.; Haoglin, D.C.; Randall, B.; Jones, A.-B.; Unger, E.R.; Reeves, W.C. Regional distribution of fatiguing illnesses in the United States: A pilot study. *Popul. Health Metr.* **2004**, *2*.
188. Bijlsma, N.; Cohen, M.M. Expert clinician's perspectives on environmental medicine and toxicant assessment in clinical practice. *Environ. Health Prev. Med.* **2018**, *23*.
189. Bileviciute-Ljungar, I.; Pajediene, E.; Friberg, D. The prevalence of restless legs syndrome (RLS) and sleep medication use in patients with myalgic encephalomyelitis/chronic fatigue syndrome (ME/CFS). *J. Sleep Res.* **2014**, *23*, 313.
190. Bileviciute-Ljungar, I.; Maroti, D.; Bejerot, S. Patients with chronic fatigue syndrome do not score higher on the autism-spectrum quotient than healthy controls: Comparison with autism spectrum disorder. *Scand. J. Psychol.* **2018**, *59*, 428–432.
191. Billiard, M.; Sonka, K. Idiopathic hypersomnia. *Sleep Med. Rev.* **2016**, *29*, 23–33.
192. Bischoff, E.; Soetekouw, P.; De Vries, M.; Scheepers, P.T.J.; Bleijenberg, G.; van der Meer, J.W.M. Chemical sensitivity in symptomatic Cambodia veterans. *Arch. Environ. Health* **2003**, *58*, 740–745.
193. Bishop, J. Epidemic myalgic encephalomyelitis. *Med. J. Aust.* **1980**, *1*, 585–586.
194. Bizzaro, N.; Tonutti, E.; Tampoia, M.; Infantino, M.; Cucchiaro, F.; Pesente, F.; Morozzi, G.; Fabris, M.; Villalta, D. Specific chemoluminescence and immunoasdorption tests for anti-DFS70 antibodies avoid false positive results by indirect immunofluorescence. *Clin. Chim. Acta* **2015**, *451*, 271–277.
195. Bjørklund, G.; Dadar, M.; Aaseth, J. Delayed-type hypersensitivity to metals in connective tissue diseases and fibromyalgia. *Environ. Res.* **2018**, *161*, 573–579.
196. Blank, P.R.; Tomonaga, Y.; Szucs, T.D.; Schwenkglenks, M. Economic burden of symptomatic iron deficiency- A survey among Swiss women 11 Medical and Health Sciences 1103 Clinical Sciences 11 Medical and Health Sciences 1117 Public Health and Health Services. *BMC Womens. Health* **2019**, *19*.
197. Blazquez, A.; Ruiz, E.; Vazquez, A.; De Sevilla, T.F.; Garcia-Quintana, A.; Garcia-Quintana, J.; Alegre, J. Sexual dysfunction as related to severity of fatigue in women with CFS. *J. Sex Marital Ther.* **2008**, *34*, 240–247.
198. Blazquez, A.; Alegre, J.; Ruiz, E. Women with chronic fatigue syndrome and sexual dysfunction: past, present, and future. *J. Sex Marital Ther.* **2009**, *35*, 347–359.
199. Blazquez, A.; Ruiz, E.; Aliste, L.; Garcia-Quintana, A.; Alegre, J. The Effect of Fatigue and Fibromyalgia on Sexual Dysfunction in Women With Chronic Fatigue Syndrome. *J. Sex Marital Ther.* **2015**, *41*, 1–10.
200. Blenkiron, P.; Edwards, R.; Lynch, S. Associations between perfectionism, mood, and fatigue in chronic fatigue syndrome: a pilot study. *J. Nerv. Ment. Dis.* **1999**, *187*, 566–570.
201. Blitshteyn, S.; Brinth, L.; Hendrickson, J.E.; Martinez-Lavin, M. Autonomic dysfunction and HPV immunization: an overview. *Immunol. Res.* **2018**, *66*, 744–754. Proceedings of the 11th International Congress on Autoimmunity in Lisbon, Portugal.
202. Blockmans, D.; Persoons, P. Long-term methylphenidate intake in chronic fatigue syndrome. *Acta Clin. Belg.* **2016**, *71*, 407–414.

203. Blondel-Hill, E.; Shafran, S.D. Treatment of the chronic fatigue syndrome. A review and practical guide. *Drugs* **1993**, *46*, 639–651.
204. Blotman, F.; Thomas, E.; Myon, E.; Andre, E.; Caubere, J.P.; Taïeb, C. Awareness and knowledge of fibromyalgia among French rheumatologists and general practitioners. *Clin. Exp. Rheumatol.* **2005**, *23*, 697–700.
205. Bode, L.; Ludwig, H. Borna disease virus infection, a human mental-health risk. *Clin. Microbiol. Rev.* **2003**, *16*, 534+.
206. Boeckle, M.; Schrimpf, M.; Liegl, G.; Pieh, C. Neural correlates of somatoform disorders from a meta-analytic perspective on neuroimaging studies. *NEUROIMAGE-CLINICAL* **2016**, *11*, 606–613.
207. Boele, F.W.; Verdonck-de Leeuw, I.M.; Cuijpers, P.; Reijneveld, J.C.; Heimans, J.J.; Klein, M. Internet-based guided self-help for glioma patients with depressive symptoms: Design of a randomized controlled trial. *BMC Neurol.* **2014**, *14*.
208. Boles, R.G.; Zaki, E.A.; Kerr, J.R.; Das, K.; Biswas, S.; Gardner, A. Increased prevalence of two mitochondrial DNA polymorphisms in functional disease: Are we describing different parts of an energy-depleted elephant? *Mitochondrion* **2015**, *23*, 1–6.
209. Bolt, E.E.; Snijdewind, M.C.; Willems, D.L.; van der Heide, A.; Onwuteaka-Philipsen, B.D. Can physicians conceive of performing euthanasia in case of psychiatric disease, dementia or being tired of living? *J. Med. Ethics* **2015**, *41*, 592–598.
210. Bolt, H.M.; Kiesswetter, E. Is multiple chemical sensitivity a clinically defined entity? *Toxicol. Lett.* **2002**, *128*, 99–106.
211. BOMBARDIER, C.H.; BUCHWALD, D. OUTCOME AND PROGNOSIS OF PATIENTS WITH CHRONIC FATIGUE VS CHRONIC FATIGUE SYNDROME. *Arch. Intern. Med.* **1995**, *155*, 2105–2110.
212. Boneva, R.S.; Decker, M.J.; Maloney, E.M.; Lin, J.-M.; Jones, J.F.; Helgason, H.G.; Heim, C.M.; Rye, D.B.; Reeves, W.C. Higher heart rate and reduced heart rate variability persist during sleep in chronic fatigue syndrome: a population-based study. *Auton. Neurosci.* **2007**, *137*, 94–101.
213. Boneva, R.S.; Lin, J.-M.S.; Wieser, F.; Nater, U.M.; Ditzen, B.; Taylor, R.N.; Unger, E.R. Endometriosis as a Comorbid Condition in Chronic Fatigue Syndrome (CFS): Secondary Analysis of Data From a CFS Case-Control Study. *Front. Pediatr.* **2019**, *7*.
214. Boneva, R.S.; Maloney, E.M.; Lin, J.-M.; Jones, J.F.; Wieser, F.; Nater, U.M.; Heim, C.M.; Reeves, W.C. Gynecological history in chronic fatigue syndrome: a population-based case-control study. *J. Womens. Health (Larchmt).* **2011**, *20*, 21–28.
215. Boot, C.R.L.; Vercoulen, J.; van der Gulden, J.W.J.; Orbon, K.H.; van den Hoogen, H.; Folgering, H.T.M. Sick leave in patients with obstructive lung disease is related to psychosocial and work variables rather than to FEV1. *Respir. Med.* **2005**, *99*, 1022–1031.
216. Bornhauser N., N.; Csef, H. New maladies - Of the soul? Reflections concerning some structural analogies among chronic fatigue syndrome, fibromyalgie and multiple chemical sensitivities [Nuevas enfermedades ¿del alma? Reflexiones psicosomáticas a propósito de algunas analogías estructurales entre síndrome de fatiga crónica, fibromialgia y sensitividad química múltiple]. *Rev. Chil. Neuropsiquiatr.* **2005**, *43*, 41–50. Work in Spanish

217. Bortolato, B.; Berk, M.; Maes, M.; McIntyre, R.S.; Carvalho, A.F. Fibromyalgia and Bipolar Disorder: Emerging Epidemiological Associations and Shared Pathophysiology. *Curr. Mol. Med.* **2016**, *16*, 119–136.
218. Boscarino, J.A. Posttraumatic stress disorder and physical illness: Results from clinical and epidemiologic studies. *Ann. N. Y. Acad. Sci.* **2004**, *1032*, 141–153. Proceedings of the Conference on Protective and Damaging Effects of the Biobehavioral Stress Response in New York, USA.
219. Bou-Holaigah, I.; Calkins, H.; Flynn, J.A.; Tunin, C.; Chang, H.-C.C.; Kan, J.S.; Rowe, P.C.; BouHolaigah, I.; Calkins, H.; Flynn, J.A.; et al. Provocation of hypotension and pain during upright tilt table testing in adults with fibromyalgia. *Clin. Exp. Rheumatol.* **1997**, *15*, 239–246.
220. BOUHOLAIGAH, I.; ROWE, P.C.; KAN, J.; CALKINS, H. THE RELATIONSHIP BETWEEN NEURALLY-MEDIATED HYPOTENSION AND THE CHRONIC FATIGUE SYNDROME. *JAMA-JOURNAL Am. Med. Assoc.* **1995**, *274*, 961–967.
221. Bould, H.; Collin, S.M.; Lewis, G.; Rimes, K.; Crawley, E. Depression in paediatric chronic fatigue syndrome. *Arch. Dis. Child.* **2013**, *98*, 425–428.
222. Bourdette, D.N.; McCauley, L.A.; Barkhuizen, A.; Johnston, W.; Wynn, M.; Joos, S.K.; Storzbach, D.; Shuell, T.; Sticker, D. Symptom factor analysis, clinical findings, and functional status in a population-based case control study of Gulf War unexplained illness. *J. Occup. Environ. Med.* **2001**, *43*, 1026–1040.
223. Bourke, J.; Soldan, J.; Silk, D.B.A.; Aziz, Q.; Libby, G.W. "Idiopathic" intestinal failure - the importance of identifying and treating primary psychopathology. *Neurogastroenterol. Motil.* **2012**, *24*, 242–251.
224. Bower, J.E. Fatigue, brain, behavior, and immunity: Summary of the 2012 Named Series on fatigue. *BRAIN Behav. Immun.* **2012**, *26*, 1220–1223.
225. Bozzini, S.; Albergati, A.; Capelli, E.; Lorusso, L.; Gazzaruso, C.; Pelissero, G.; Falcone, C. Cardiovascular characteristics of chronic fatigue syndrome. *Biomed. Reports* **2018**, *8*, 26–30.
226. Branco, J.C.; Tavares, V.; Abreu, I.; Humbel, R.L. Viral infection and fibromyalgia [Infecção viral e fibromialgia.]. *Acta Med. Port.* **1994**, *7*, 337–341. Work in Portuguese
227. Braun, D.K.; Dominguez, G.; Pellett, P.E. Human herpesvirus 6. *Clin. Microbiol. Rev.* **1997**, *10*, 521–567.
228. Braun, M.; Vierling, J.M. The clinical and immunologic impact of using interferon and ribavirin in the immunosuppressed host. *Liver Transplant.* **2003**, *9*, S79–S89.
229. Breau, L.M.; McGrath, P.J.; Ju, L.H. Review of juvenile primary fibromyalgia and chronic fatigue syndrome. *J. Dev. Behav. Pediatr.* **1999**, *20*, 278–288.
230. Breuer, K.; Diehl, V.; Rüffer, U. Long-term toxicities following treatment for Hodgkin's disease [Langzeittoxizitaten in der behandlung des morbus Hodgkin]. *Med. Klin.* **2000**, *95*, 378–384. Work in German
231. Brewer, J.H.; Thrasher, J.D.; Straus, D.C.; Madison, R.A.; Hooper, D. Detection of mycotoxins in patients with chronic fatigue syndrome. *Toxins (Basel).* **2013**, *5*, 605–617.
232. Brewer, J.M.; Berg, D. Hypercoaguable state associated with active human herpesvirus-6 (HHV-6) viremia in patients with chronic fatigue syndrome. *J. Chronic Fatigue Syndr.* **2001**, *8*, 111–116.
233. Brigden, A.; Beasant, L.; Hollingworth, W.; Metcalfe, C.; Gaunt, D.; Mills, N.; Jago, R.; Crawley, E. Managed Activity Graded Exercise iN Teenagers and pre-

Adolescents (MAGENTA) feasibility randomised controlled trial: study protocol. *BMJ Open* **2016**, *6*.
234. Brigden, A.; Loades, M.; Abbott, A.; Bond-Kendall, J.; Crawley, E. Practical management of chronic fatigue syndrome or myalgic encephalomyelitis in childhood. *Arch. Dis. Child.* **2017**, *102*, 981–986.
235. Briggs, N.C.; Levine, P.H. A comparative review of systemic and neurological symptomatology in 12 outbreaks collectively described as chronic fatigue syndrome, epidemic neuromyasthenia, and myalgic encephalomyelitis. *Clin. Infect. Dis.* **1994**, *18 Suppl 1*, S32-42.
236. Brimacombe, M.; Helmer, D.A.; Natelson, B.H. Birth order and its association with the onset of chronic fatigue syndrome. *Hum. Biol.* **2002**, *74*, 615–620.
237. Brinth, L.; Pors, K.; Spahic, J.M.; Sutton, R.; Fedorowski, A.; Mehlsen, J. Postural Orthostatic Tachycardia Syndrome (POTS) in Denmark: Increasingly recognized or new epidemic? *Auton. Neurosci. Clin.* **2018**, *213*, 92–95.
238. Brinton, L.A.; Buckley, L.M.; Dvorkina, O.; Lubin, J.H.; Colton, T.; Murray, M.C.; Hoover, R.N. Risk of connective tissue disorders among breast implant patients. *Am. J. Epidemiol.* **2004**, *160*, 619–627.
239. Brito-Zerón, P.; Nicolás-Ocejo, D.; Jordán, A.; Retamozo, S.; López-Soto, A.; Bosch, X. Diagnosing unexplained fever: Can quick diagnosis units replace inpatient hospitalization? *Eur. J. Clin. Invest.* **2014**, *44*, 707–718.
240. Brkić, S.; Tomić, S.; Ružić, M.; Marić, D.; Brkic, S.; Tomic, S.; Ruzic, M.; Maric, D. Chronic Fatigue Syndrome. *Srp. Arh. Celok. Lek.* **2011**, *139*, 256–261.
241. Broadbent, S.; Coutts, R. The protocol for a randomised controlled trial comparing intermittent and graded exercise to usual care for chronic fatigue syndrome patients. *BMC Sports Sci. Med. Rehabil.* **2013**, *5*.
242. Broderick, G.; Katz, B.Z.; Fernandes, H.; Fletcher, M.A.; Klimas, N.; Smith, F.A.; O'Gorman, M.R.G.; Vernon, S.D.; Taylor, R. Cytokine expression profiles of immune imbalance in post-mononucleosis chronic fatigue. *J. Transl. Med.* **2012**, *10*.
243. Brooks, L.; Hadi, J.; Amber, K.T.; Weiner, M.; La Riche, C.L.; Ference, T. Assessing the prevalence of autoimmune, endocrine, gynecologic, and psychiatric comorbidities in an ethnically diverse cohort of female fibromyalgia patients: does the time from hysterectomy provide a clue? *J. Pain Res.* **2015**, *8*, 561–569.
244. Brooks, S.K.; Chalder, T.; Rimes, K.A. Chronic Fatigue Syndrome: Cognitive, Behavioural and Emotional Processing Vulnerability Factors. *Behav. Cogn. Psychother.* **2017**, *45*, 156–169.
245. Brown, H.; Cartwright, R. Re: The Relationship between Irritable Bowel Syndrome, Functional Dyspepsia, Chronic Fatigue and Overactive Bladder Syndrome: A Controlled Study 6 Years after Acute Gastrointestinal Infection. *Eur. Urol.* **2015**, *68*, 1099–1100.
246. Brown, J.D. A description of "Australian Lyme disease" epidemiology and impact: an analysis of submissions to an Australian senate inquiry. *Intern. Med. J.* **2018**, *48*, 422–426.
247. Brown, R.J. Introduction to the special issue on medically unexplained symptoms: background and future directions. *Clin. Psychol. Rev.* 2007, *27*, 769–780.
248. Browne, T.; Chalder, T. Chronic fatigue syndrome. *Psychiatry* **2009**, *8*, 153–157.
249. Browne, T.; Chalder, T. Chronic fatigue syndrome. *Psychiatry* **2006**, *5*, 48–51.
250. Bruno, R.L. Paralytic vs. "nonparalytic" polio: Distinction without a difference? *Am. J. Phys. Med. Rehabil.* **2000**, *79*, 4–12.

251. Brurberg, K.G.; Fønhus, M.S.; Larun, L.; Flottorp, S.; Malterud, K. Case definitions for chronic fatigue syndrome/myalgic encephalomyelitis (CFS/ME): A systematic review. *BMJ Open* **2014**, *4*.
252. Buchwald, D.; Herrell, R.; Ashton, S.; Belcourt, M.; Schmaling, K.; Sullivan, P.; Neale, M.; Goldberg, J. A twin study of chronic fatigue. *Psychosom. Med.* **2001**, *63*, 936–943.
253. Buchwald, D.; Pascualy, R.; Bombardier, C.; Kith, P. Sleep disorders in patients with chronic fatigue. *Clin. Infect. Dis.* **1994**, *18*, S68–S72.
254. Buchwald, D.; Pearlman, T.; Kith, P.; Katon, W.; Schmaling, K. Screening for psychiatric disorders in chronic fatigue and chronic fatigue syndrome. *J. Psychosom. Res.* **1997**, *42*, 87–94.
255. Buchwald, D.; Pearlman, T.; Kith, P.; Schmaling, K. Gender differences in patients with chronic fatigue syndrome. *J. Gen. Intern. Med.* **1994**, *9*, 397–401.
256. Buchwald, D.; Umali, P.; Umali, J.; Kith, P.; Pearlman, T.; Komaroff, A.L. Chronic fatigue and the chronic fatigue syndrome: prevalence in a Pacific Northwest health care system. *Ann. Intern. Med.* **1995**, *123*, 81–88.
257. Buck Louis, G.M.; Bell, E.; Xie, Y.; Sundaram, R.; Yeung, E. Parental health status and infant outcomes: Upstate KIDS Study. *Fertil. Steril.* **2018**, *109*, 315–323.
258. Buck, R.; Barnes, M.C.; Cohen, D.; Aylward, M. Common Health Problems, Yellow Flags and Functioning in a Community Setting. *J. Occup. Rehabil.* **2010**, *20*, 235–246.
259. Buckingham, E.T.; Daniolos, P. Longitudinal outcomes for victims of child abuse. *Curr. Psychiatry Rep.* **2013**, *15*.
260. Buechel, H.M.; Popovic, J.; Staggs, K.; Anderson, K.L.; Thibault, O.; Blalock, E.M. Aged rats are hypo-responsive to acute restraint: implications for psychosocial stress in aging. *Front. Aging Neurosci.* **2014**, *6*.
261. Buffington, C.A.T. Developmental influences on medically unexplained symptoms. *Psychother. Psychosom.* **2009**, *78*, 139–144.
262. Bulbena, A.; Sperry, L.; Pailhez, G.; Gago, J. Anxiety, temporomandibular disorders, and joint hypermobility syndrome. Response to "Psychological assessment of patients with temporomandibular disorders: confirmatory analysis of the dimensional structure of the Brief Symptoms Inventory 18." *J. Psychosom. Res.* **2006**, *61*, 851.
263. Bullinger, M. Environmental health conditions [Umweltbezogene gesundheitsstörungen]. *PPmP Psychother. Psychosom. Medizinische Psychol.* **2008**, *58*, 430–442. Work in German
264. Bullinger, M. [Environmental health conditions]. *Psychother. Psychosom. Med. Psychol.* **2008**, *58*, 430–440. Work in German
265. Bürgel, B.; Friesland, M.; Koch, A.; Manns, M.P.; Wedemeyer, H.; Weissenborn, K.; Schulz-Schaeffer, W.J.; Pietschmann, T.; Steinmann, E.; Ciesek, S.; et al. Hepatitis C virus enters human peripheral neuroblastoma cells - Evidence for extra-hepatic cells sustaining hepatitis C virus penetration. *J. Viral Hepat.* **2011**, *18*, 562–570.
266. Burke, P.; Elliott, M.; Fleissner, R. Irritable bowel syndrome and recurrent abdominal pain: A comparative review. *Psychosomatics* **1999**, *40*, 277–285.
267. Burton, C. Beyond somatisation: A review of the understanding and treatment of medically unexplained physical symptoms (MUPS). *Br. J. Gen. Pract.* **2003**, *53*, 231–239.
268. Buskila, D. Fibromyalgia, chronic fatigue syndrome, and myofascial pain syndrome. *Curr. Opin. Rheumatol.* **2001**, *13*, 117–127.

269. Buskila, D. Fibromyalgia, chronic fatigue syndrome, and myofascial pain syndrome. *Curr. Opin. Orthop.* **2000**, *11*, 215–224.
270. Buskila, D.; Atzeni, F.; Sarzi-Puttini, P. Etiology of fibromyalgia: The possible role of infection and vaccination. *Autoimmun. Rev.* **2008**, *8*, 41–43.
271. Buskila, D.; Sarzi-Puttini, P. Biology and therapy of fibromyalgia: Genetic aspects of fibromyalgia syndrome. *Arthritis Res. Ther.* **2006**, *8*.
272. Butler, J.A.; Chalder, T.; Wessely, S. Causal attributions for somatic sensations in patients with chronic fatigue syndrome and their partners. *Psychol. Med.* **2001**, *31*, 97–105.
273. Butts, B.N.; Fischer, P.R.; MacK, K.J. Human Papillomavirus Vaccine and Postural Orthostatic Tachycardia Syndrome: A Review of Current Literature. *J. Child Neurol.* **2017**, *32*, 956–965.
274. Buyukkose, M.; Kozanoglu, E.; Basaran, S.; Bayramoglu, O.; Yarkin, F. Seroprevalence of parvovirus B19 in fibromyalgia syndrome. *Clin. Rheumatol.* **2009**, *28*, 305–309.
275. Cairns, R.; Hotopf, M. A systematic review describing the prognosis of chronic fatigue syndrome. *Occup. Med.* **2005**, *55*, 20–31.
276. Cairns, V.; Godwin, J. Post-Lyme borreliosis syndrome: A meta-analysis of reported symptoms. *Int. J. Epidemiol.* **2005**, *34*, 1340–1345.
277. Cajanding, R.J.M. Causes, assessment and management of fatigue in critically ill patients. *Br. J. Nurs.* **2017**, *26*, 1176–1181.
278. CALABRESE, L.H.; DAVIS, M.E.; WILKE, W.S. CHRONIC FATIGUE SYNDROME AND A DISORDER RESEMBLING SJOGRENS-SYNDROME - PRELIMINARY-REPORT. *Clin. Infect. Dis.* **1994**, *18*, S28–S31.
279. Calabrese, L.H.; Davis, M.E.; Wilke, W.S. Chronic fatigue syndrome and a disorder resembling Sjogren’s syndrome: preliminary report. *Clin. Infect. Dis.* **1994**, *18 Suppl 1*, S28-31.
280. Calandre, E.P.; Rico-Villademoros, F. The role of antipsychotics in the management of fibromyalgia. *CNS Drugs* **2012**, *26*, 135–153.
281. Callaway, E. Virology: Fighting for a cause. *Nature* **2011**, *471*, 282–285.
282. Calvo, N.; Pueyo, N.; Gutierrez, F.; Ferrer, M.; Castro-Marrero, J.; Alegre, J.; Casas, M.; Ramos Quiroga, J.-A.; Saez-Francas, N. Dimensional Personality Assessment among a Chronic Fatigue Syndrome (CFS) sample with Personality Inventory for DSM-5 (PID-5). *Actas Esp. Psiquiatr.* **2018**, *46*, 125–132.
283. Calvo, N.; Saez-Francas, N.; Valero, S.; Alegre, J.; Casas, M. Comorbid personality disorders in chronic fatigue syndrome patients: a marker of psychopathological severity. *Actas Esp. Psiquiatr.* **2015**, *43*, 58–65.
284. Cambras, T.; Castro-Marrero, J.; Zaragoza, M.C.; Diez-Noguera, A.; Alegre, J. Circadian rhythm abnormalities and autonomic dysfunction in patients with Chronic Fatigue Syndrome/Myalgic Encephalomyelitis. *PLoS One* **2018**, *13*, e0198106.
285. Cameron, B.; Flamand, L.; Juwana, H.; Middeldorp, J.; Naing, Z.; Rawlinson, W.; Ablashi, D.; Lloyd, A. Serological and Virological Investigation of the Role of the Herpesviruses EBV, CMV and HHV-6 in Post-Infective Fatigue Syndrome. *J. Med. Virol.* **2010**, *82*, 1684–1688.
286. Cameron, K.; Williamson, P.; Short, M.A.; Gradisar, M. Validation of the Flinders Fatigue Scale as a measure of daytime fatigue. *SLEEP Med.* **2017**, *30*, 105–112.
287. Cameron, R.L.; Ahmed, S.; Pollock, K.G.J. Adverse event monitoring of the human papillomavirus vaccines in Scotland. *Intern. Med. J.* **2016**, *46*, 452–457.

288. Caminero, A.; Bartolomé, M. Sleep disturbances in multiple sclerosis. *J. Neurol. Sci.* **2011**, *309*, 86–91.
289. Campbell-Tofte, J.; Vrahatis, A.; Josefsen, K.; Mehlsen, J.; Winther, K. Investigating the aetiology of adverse events following HPV vaccination with systems vaccinology. *Cell. Mol. Life Sci.* **2019**, *76*, 67–87.
290. Campistol, J. Chronic fatigue syndrome in adolescents [Sindrome de fatiga cronica a l'adolescencia]. *Pediatr. Catalana* **1997**, *57*, 294–302. Work in Catala
291. Cao, Y.; Zhang, Y.; Chang, D.F.; Wang, G.; Zhang, X. Psychosocial and Immunological Factors in Neurasthenia. *Psychosomatics* **2009**, *50*, 24–29.
292. Capelli, E.; Lorusso, L.; Ghitti, M.; Venturini, L.; Cusa, C.; Ricevuti, G. Chronic fatigue syndrome: Features of a population of patients from northern Italy. *Int. J. Immunopathol. Pharmacol.* **2015**, *28*, 53–59.
293. Capelli, E.; Zola, R.; Lorusso, L.; Venturini, L.; Sardi, F.; Ricevuti, G. Chronic fatigue syndrome/myalgic encephalomyelitis: an update. *Int. J. Immunopathol. Pharmacol.* **2010**, *23*, 981–989.
294. Carlo-Stella, N.; Bozzini, S.; De Silvestri, A.; Sbarsi, I.; Pizzochero, C.; Lorusso, L.; Martinetti, M.; Cuccia, M. Molecular study of receptor for advanced glycation endproduct gene promoter and identification of specific HLA haplotypes possibly involved in chronic fatigue syndrome. *Int. J. Immunopathol. Pharmacol.* **2009**, *22*, 745–754.
295. Carlo-Stella, N.; Cuccia, M. Demographic and clinical aspects of an Italian patient population with chronic fatigue syndrome. *Reumatismo* **2009**, *61*, 285–289.
296. Carlo-Stella, N.; Lorusso, L.; Candura, S.M.; Cuccia, M. Chronic fatigue syndrome: A review [La sindrome da stanchezza cronica]. *Recenti Prog. Med.* **2004**, *95*, 546–552+560.
297. Carlo-Stella, N.; Lorusso, L.; Candura, S.M.; Cuccia, M. [Chronic fatigue syndrome: a review]. *Recenti Prog. Med.* **2004**, *95*, 546–52; quiz 560. Work in Italian
298. Carlsson, F.; Persson, R.; Karlson, B.; Osterberg, K.; Marie, A.; Garde, A.H.; Orbaek, P. Salivary cortisol and self-reported stress among persons with environmental annoyance. *Scand. J. Work Environ. Heal.* **2006**, *32*, 109–120.
299. Carter, B.D.; Edwards, J.F.; Kronenberger, W.G.; Michalczyk, L.; Marshall, G.S. Case control study of chronic fatigue in pediatric patients. *Pediatrics* **1995**, *95*, 179–186.
300. Carter, C.J. Susceptibility genes are enriched in those of the herpes simplex virus 1/host interactome in psychiatric and neurological disorders. *Pathog. Dis.* **2013**, *69*, 240–261.
301. Casale, R.; Sarzi-Puttini, P.; Atzeni, F.; Gazzoni, M.; Buskila, D.; Rainoldi, A. Central motor control failure in fibromyalgia: a surface electromyography study. *BMC Musculoskelet. Disord.* **2009**, *10*.
302. Caserta, M.T.; Mock, D.J.; Dewhurst, S. Human herpesvirus 6. *Clin. Infect. Dis.* **2001**, *33*, 829–833.
303. Cassina, G.; Russo, D.; De Battista, D.; Broccolo, F.; Lusso, P.; Malnati, M.S. Calibrated real-time polymerase chain reaction for specific quantitation of HHV-6A and HHV-6B in clinical samples. *J. Virol. Methods* **2013**, *189*, 172–179.
304. Cassisi, G.; Sarzi-Puttini, P.; Cazzola, M. Chronic widespread pain and fibromyalgia: Could there be some relationship with infections and vaccinations? *Clin. Exp. Rheumatol.* **2011**, *29*, S118–S126.
305. Castell, L.M. *Physical or mental fatigue and immunodepression*; 2008;

306. Castilla-Puentes, R.C.; Secin, R.; Grau, A.; Galeno, R.; De Mello, M.F.; Peňa, N.; Sanchez-Russi, C.A. A multicenter study of major depressive disorder among emergency department patients in Latin-American countries. *Depress. Anxiety* **2008**, *25*, E199–E204.
307. Castori, M.; Celletti, C.; Camerota, F.; Grammatico, P. Chronic fatigue syndrome is commonly diagnosed in patients with Ehlers-Danlos syndrome hypermobility type/joint hypermobility syndrome. *Clin. Exp. Rheumatol.* 2011, *29*, 597–598.
308. Castro-Marrero, J.; Sáez-Francàs, N.; Santillo, D.; Alegre, J. Treatment and management of chronic fatigue syndrome/myalgic encephalomyelitis: all roads lead to Rome. *Br. J. Pharmacol.* **2017**, *174*, 345–369.
309. Castro-Marrero, J.; Faro, M.; Aliste, L.; Saez-Francas, N.; Calvo, N.; Martinez-Martinez, A.; de Sevilla, T.F.; Alegre, J. Comorbidity in Chronic Fatigue Syndrome/Myalgic Encephalomyelitis: A Nationwide Population-Based Cohort Study. *Psychosomatics* **2017**, *58*, 533–543.
310. Catassi, C.; Kryszak, D.; Louis-Jacques, O.; Duerksen, D.R.; Hill, I.; Crowe, S.E.; Brown, A.R.; Procaccini, N.J.; Wonderly, B.A.; Hartley, P.; et al. Detection of celiac disease in primary care: A multicenter case-finding study in North America. *Am. J. Gastroenterol.* **2007**, *102*, 1454–1460.
311. Cathébras, P. Fibromyalgia. A critical review [La fibromyalgie. Une revue critique]. *Ann. Med. Interne (Paris).* **1998**, *149*, 406–414. Work in French
312. CATHEBRAS, P.; BOUCHOU, K.; CARTRY, O.; ROUSSET, H. THE EPIDEMIOLOGY OF FATIGUE - IMPLICATIONS FOR THE DEFINITION OF CHRONIC FATIGUE SYNDROME. *Sem. DES Hop.* **1995**, *71*, 111–118.
313. Celej-Szuster, J.; Muzyczka, K.; Anasiewicz, M.; Kociuba-Adamczuk, K.; Turowski, K.; Stanisławek, A.; Kachaniuk, H.; Szadowska-Szlachetka, Z. Multiple sclerosis - Epidemiology, diagnosis, treatment and rehabilitation - A literature review. *Curr. Issues Pharm. Med. Sci.* **2012**, *25*, 61–63.
314. Cella, M.; White, P.D.; Sharpe, M.; Chalder, T. Cognitions, behaviours and co-morbid psychiatric diagnoses in patients with chronic fatigue syndrome. *Psychol. Med.* **2013**, *43*, 375–380.
315. Cella, M.; Chalder, T. Measuring fatigue in clinical and community settings. *J. Psychosom. Res.* **2010**, *69*, 17–22.
316. Cervigni, M.; Natale, F. Gynecological disorders in bladder pain syndrome/interstitial cystitis patients. *Int. J. Urol.* **2014**, *21*, 85–88.
317. Chalder, T.; Godfrey, E.; Ridsdale, L.; King, M.; Wessely, S. Predictors of outcome in a fatigued population in primary care following a randomized controlled trial. *Psychol. Med.* **2003**, *33*, 283–287.
318. Chalder, T.; Goodman, R.; Wessely, S.; Hotopf, M.; Meltzer, H. Epidemiology of chronic fatigue syndrome and self reported myalgic encephalomyelitis in 5-15 year olds: cross sectional study. *BMJ* **2003**, *327*, 654–655.
319. Chalmers, K.J.; Catley, M.J.; Evans, S.F.; Moseley, G.L. Clinical assessment of the impact of pelvic pain on women. *Pain* **2017**, *158*, 498–504.
320. Chalmers, R.M.; Thomas, D.R.; Salmon, R.L. Borna disease virus and the evidence for human pathogenicity: A systematic review. *QJM - Mon. J. Assoc. Physicians* **2005**, *98*, 255–274.
321. Chan, J.S.M.; Ho, R.T.H.; Wang, C.; Yuen, L.P.; Sham, J.S.T.; Chan, C.L.W. Effects of Qigong Exercise on Fatigue, Anxiety, and Depressive Symptoms of Patients with Chronic Fatigue Syndrome-Like Illness: A Randomized Controlled Trial. *EVIDENCE-BASED Complement. Altern. Med.* **2013**.

322. Chan, W.; Bosch, J.A.; Jones, D.; Kaur, O.; Inston, N.; Moore, S.; McClean, A.; McTernan, P.G.; Harper, L.; Phillips, A.C.; et al. Predictors and consequences of fatigue in prevalent kidney transplant recipients. *Transplantation* **2013**, *96*, 987–994.
323. Chandler, H.K.; Ciccone, D.; MacBride, R.J.; Natelson, B. Medically unexplained illness in short- and long-term disability applicants: Prevalence and cost of salary reimbursement. *Disabil. Rehabil.* **2008**, *30*, 1185–1191.
324. Chandran, V.; Pal, P.K.; Reddy, J.Y.C.; Thennarasu, K.; Yadav, R.; Shivashankar, N. Non-motor features in essential tremor. *Acta Neurol. Scand.* **2012**, *125*, 332–337.
325. Chandrashekar, C.R.; Math, S.B. Psychosomatic disorders in developing countries: current issues and future challenges. *Curr. Opin. Psychiatry* **2006**, *19*, 201–206.
326. Chang, C.M.; Warren, J.L.; Engels, E.A. Chronic fatigue syndrome and subsequent risk of cancer among elderly US adults. *Cancer* **2012**, *118*, 5929–5936.
327. Chang, F.-L.; Sun, Y.-M.; Chuang, K.-H.; Hsu, D.-J. Work fatigue and physiological symptoms in different occupations of high-elevation construction workers. *Appl. Ergon.* **2009**, *40*, 591–596.
328. Chang, F.-Y.; Lu, C.-L. Irritable bowel syndrome and migraine: Bystanders or partners? *J. Neurogastroenterol. Motil.* **2013**, *19*, 301–311.
329. Chanson, J.-B.; Zephir, H.; Collongues, N.; Outteryck, O.; Blanc, F.; Fleury, M.; Vermersch, P.; de Seze, J. Evaluation of health-related quality of life, fatigue and depression in neuromyelitis optica. *Eur. J. Neurol.* **2011**, *18*, 836–841.
330. Chao, C.-H.; Chen, H.-J.; Wang, H.-Y.; Li, T.-C.; Kao, C.-H. Increased risk of organic erectile dysfunction in patients with chronic fatigue syndrome: a nationwide population-based cohort study. *Andrology* **2015**, *3*, 666–671.
331. Chapenko, S.; Krumina, A.; Kozireva, S.; Nora, Z.; Sultanova, A.; Viksna, L.; Murovska, M. Activation of human herpesviruses 6 and 7 in patients with chronic fatigue syndrome. *J. Clin. Virol.* **2006**, *37 Suppl 1*, S47-51. Proceedings of the 5th International Conference on Human Herpesvirus 6 and 7 (HHV-6&7) in Barcelona, Spain.
332. Chastin, S.F.M.; Granat, M.H. Methods for objective measure, quantification and analysis of sedentary behaviour and inactivity. *Gait Posture* **2010**, *31*, 82–86.
333. Chaudhuri, A.; Robson, J. Q fever queries and answers. *Med. Today* **2013**, *14*, 54–59.
334. Chen, C.-S.; Lin, W.-M.; Yang, T.-Y.; Chen, H.-J.; Kuo, C.-N.; Kao, C.-H. Chronic fatigue syndrome is associated with the risk of fracture: a nationwide cohort study. *QJM* **2014**, *107*, 635–641.
335. Chen, C.-H.; Yang, T.-Y.; Lin, C.-L.; Chen, C.-S.; Lin, W.-M.; Kuo, C.-N.; Lin, M.-C.; Kao, C.-H. Dry eye syndrome risks in patients with Fibromyalgia: A national retrospective cohort study. *Med. (United States)* **2016**, *95*.
336. Chen, C.-S.; Cheng, H.-M.; Chen, H.-J.; Tsai, S.-Y.; Kao, C.-H.; Lin, H.-J.; Wan, L.; Yang, T.-Y. Dry eye syndrome and the subsequent risk of chronic fatigue syndrome-a prospective population-based study in Taiwan. *Oncotarget* **2018**, *9*, 30694–30703.
337. Chen, I.-C.; Lee, M.; Wu, S.-L.; Lin, H.-H.; Chang, K.-M.; Lin, H. Somatic symptoms are sensitive in predicting interstitial cystitis/bladder pain syndrome: A nationwide cohort study. *Int. J. Psychiatry Med.* **2017**, *52*, 48–61.
338. Chen, Y.-W.; Camp, P.G.; Coxson, H.O.; Road, J.D.; Guenette, J.A.; Hunt, M.A.; Reid, W.D. Comorbidities That Cause Pain and the Contributors to Pain in

Individuals With Chronic Obstructive Pulmonary Disease. *Arch. Phys. Med. Rehabil.* **2017**, *98*, 1535–1543.
339. Chen, Y.; Wei, J. Identification of Pathogen Signatures in Prostate Cancer Using RNA-seq. *PLoS One* **2015**, *10*.
340. Cheng, H.; Gurland, B.J.; Maurer, M.S. Self-reported lack of energy (anergia) among elders in a multiethnic community. *JOURNALS Gerontol. Ser. A-BIOLOGICAL Sci. Med. Sci.* **2008**, *63*, 707–714.
341. Chester, A.C. Symptoms of rhinosinusitis in patients with unexplained chronic fatigue or bodily pain: A pilot study. *Arch. Intern. Med.* **2003**, *163*, 1832–1836.
342. Chester, A.C. Yeast and chronic fatigue syndrome. *J. Chronic Fatigue Syndr.* **2000**, *7*, 87–88.
343. Chester, A.C. Chronic fatigue syndrome criteria in patients with other forms of unexplained chronic fatigue. *J. Psychiatr. Res.* **1997**, *31*, 45–50. Proceedings of the 1st Research and Clinical Conference of the American-Association-for-Chronic-Fatigue-Syndrome in FT Lauderdale, USA.
344. Chester, A.C.; Levine, P.H. The natural history of concurrent sick building syndrome and chronic fatigue syndrome. *J. Psychiatr. Res.* **1997**, *31*, 51–57. Proceedings of the 1st Research and Clinical Conference of the American-Association-for-Chronic-Fatigue-Syndrome in FT Lauderdale, USA.
345. Chester, A.C.; Levine, P.H. Concurrent sick building syndrome and chronic fatigue syndrome: epidemic neuromyasthenia revisited. *Clin. Infect. Dis.* **1994**, *18 Suppl 1*, S43-8.
346. Chester, A.C. Chronic rhinosinusitis-related fatigue: does the Internet provide a clue? *Ear. Nose. Throat J.* 2008, *87*, 310; author reply 310.
347. Cheung, F.; Lin, K.M. Neurasthenia, depression and somatoform disorder in a Chinese-Vietnamese woman migrant. *Cult. Med. Psychiatry* **1997**, *21*, 247–258.
348. Cho, H.J.; Bhugra, D.; Wessely, S. `Physical or psychological?' - a comparative study of causal attribution for chronic fatigue in Brazilian and British primary care patients. *ACTA Psychiatr. Scand.* **2008**, *118*, 34–41.
349. Cho, H.J.; Kivimaki, M.; Bower, J.E.; Irwin, M.R. Association of C-reactive protein and interleukin-6 with new-onset fatigue in the Whitehall II prospective cohort study. *Psychol. Med.* **2013**, *43*, 1773–1783.
350. Cho, H.J.; Wessely, S. The prevalence and associations of unexplained chronic fatigue in Brazilian primary care. *Prim. Care Community Psychiatry* **2007**, *12*, 81–87.
351. Cho, H.J.; Menezes, P.R.; Bhugra, D.; Wessely, S. The awareness of chronic fatigue syndrome: a comparative study in Brazil and the United Kingdom. *J. Psychosom. Res.* **2008**, *64*, 351–355.
352. Cho, H.J.; Menezes, P.R.; Hotopf, M.; Bhugra, D.; Wessely, S. Comparative epidemiology of chronic fatigue syndrome in Brazilian and British primary care: prevalence and recognition. *Br. J. Psychiatry* **2009**, *194*, 117–122.
353. Choi, C.-J.; Knutsen, R.; Oda, K.; Fraser, G.E.; Knutsen, S.F. The Association Between Incident Self-reported Fibromyalgia and Nonpsychiatric Factors: 25-years Follow-up of the Adventist Health Study. *J. PAIN* **2010**, *11*, 994–1003.
354. Chou, K.-L. Chronic fatigue and affective disorders in older adults: Evidence from the 2007 British National Psychiatric Morbidity Survey. *J. Affect. Disord.* **2013**, *145*, 331–335.
355. Choung, R.S.; Locke, G.R. Epidemiology of IBS. *Gastroenterol. Clin. North Am.* **2011**, *40*, 1–10.

356. Christley, Y.; Hollins Martin, C.J.; Martin, C.R. Perinatal perspectives on chronic fatigue syndrome. *Br. J. Midwifery* **2012**, *20*, 389–393.
357. Christley, Y.; Duffy, T.; Martin, C.R. A review of the definitional criteria for chronic fatigue syndrome. *J. Eval. Clin. Pract.* **2012**, *18*, 25–31.
358. Chu, L. Cytokine inhibition in patients with chronic fatigue syndrome. *Ann. Intern. Med.* **2017**, *167*, 447–448.
359. Chu, L.; Valencia, I.J.; Garvert, D.W.; Montoya, J.G. Onset patterns and course of myalgic encephalomyelitis/chronic fatigue syndrome. *Front. Pediatr.* **2019**, *7*.
360. Chu, L.; Norris, J.L.; Valencia, I.J.; Montoya, J.G. Patients diagnosed with Myalgic encephalomyelitis/chronic fatigue syndrome also fit systemic exertion intolerance disease criteria. *FATIGUE-BIOMEDICINE Heal. Behav.* **2017**, *5*, 114–128.
361. Chu, L.; Valencia, I.J.; Garvert, D.W.; Montoya, J.G. Deconstructing post-exertional malaise in myalgic encephalomyelitis/ chronic fatigue syndrome: A patient-centered, cross-sectional survey. *PLoS One* **2018**, *13*, e0197811.
362. Chung, S.-D.; Lin, C.-C.; Liu, S.-P.; Lin, H.-C. Obstructive sleep apnea increases the risk of bladder pain syndrome/interstitial cystitis: A population-based matched-cohort study. *Neurourol. Urodyn.* **2014**, *33*, 278–282.
363. Chung, S.-D.; Liu, S.-P.; Lin, C.-C.; Li, H.-C.; Lin, H.-C. Bladder Pain Syndrome/Interstitial Cystitis Is Associated with Hyperthyroidism. *PLoS One* **2013**, *8*.
364. Church, A.J. Myalgic encephalomyelitis: “an obscene cosmic joke”? *Med. J. Aust.* **1980**, *1*, 307–308.
365. Ciccone, D.S.; Busichio, K.; Vickroy, M.; Natelson, B.H. Psychiatric morbidity in the chronic fatigue syndrome: are patients with personality disorder more physically impaired? *J. Psychosom. Res.* **2003**, *54*, 445–452.
366. Ciccone, D.S.; Natelson, B.H. Comorbid illness in women with chronic fatigue syndrome: a test of the single syndrome hypothesis. *Psychosom. Med.* **2003**, *65*, 268–275. Proceedings of the 59th Annual Meeting of the American-Psychosomatic-Society in Monterey, USA.
367. Ciccone, D.S.; Weissman, L.; Natelson, B.H. Chronic fatigue syndrome in male Gulf war veterans and civilians: a further test of the single syndrome hypothesis. *J. Health Psychol.* **2008**, *13*, 529–536.
368. Cioni, F. Experience in an oncological environment on Chronic Fatigue Syndrome as a reference model in the treatment of general fatigue. *Prog. Nutr.* **2013**, *15*, 126–132.
369. Claliw, D.J. The pathogenesis of chronic pain and fatigue syndromes, with special reference to fibromyalgia. *Med. Hypotheses* **1995**, *44*, 369–378.
370. Clark, C.J.; Khattab, A.D.; Carr, E.C.J. Chronic widespread pain and neurophysiological symptoms in joint hypermobility syndrome (JHS). *Int. J. Ther. Rehabil.* **2014**, *21*, 60–67.
371. Clark, C.; Goodwin, L.; Stansfeld, S.A.; Hotopf, M.; White, P.D. Premorbid risk markers for chronic fatigue syndrome in the 1958 British birth cohort. *Br. J. Psychiatry* **2011**, *199*, 323–329.
372. Clark, J.E.; Davidson, S.L.; Maclachlan, L.; Newton, J.L.; Watson, S. Rethinking childhood adversity in chronic fatigue syndrome. *FATIGUE-BIOMEDICINE Heal. Behav.* **2018**, *6*, 20–29.
373. Clark, J.E.; Ng, W.F.; Watson, S.; Newton, J.L. The aetiopathogenesis of fatigue: unpredictable, complex and persistent. *Br. Med. Bull.* **2016**, *117*, 139–148.

374. Clarke, J.N.; James, S. The radicalized self: The impact on the self of the contested nature of the diagnosis of chronic fatigue syndrome. *Soc. Sci. Med.* **2003**, *57*, 1387–1395.
375. Clauson, K.A.; Zeng-Treitler, Q.; Kandula, S. Readability of Patient and Health Care Professional Targeted Dietary Supplement Leaflets Used for Diabetes and Chronic Fatigue Syndrome. *J. Altern. Complement. Med.* **2010**, *16*, 119–124.
376. Clauw, D. Fibromyalgia associated syndromes. *J. Musculoskelet. Pain* **2002**, *10*, 201–214. Proceedings of the 5th World Congress on Myofascial Pain and Fibromyalgia (MYOPAIN 2001) in Portland, USA.
377. CLAUW, D.J. THE PATHOGENESIS OF CHRONIC PAIN AND FATIGUE SYNDROMES, WITH SPECIAL REFERENCE TO FIBROMYALGIA. *Med. Hypotheses* **1995**, *44*, 369–378.
378. Clauw, D.J.; Williams, D.A. Physical trauma and fibromyalgia. *Trauma* **2002**, *44*, 5–25.
379. Cleare, A.J.; Blair, D.; Chambers, S.; Wessely, S. Urinary free cortisol in chronic fatigue syndrome. *Am. J. Psychiatry* **2001**, *158*, 641–643.
380. Clemens, J.Q.; Elliott, M.N.; Suttorp, M.; Berry, S.H. Temporal ordering of interstitial cystitis/bladder pain syndrome and non-bladder conditions. *Urology* **2012**, *80*, 1227–1231.
381. Coetzee, N.; Maree, D.J.F.; Smit, B.N. The relationship between chronic fatigue syndrome, burnout, job satisfaction, social support and age among academics at a tertiary institution. *Int. J. Occup. Med. Environ. Health* **2019**, *32*, 75–85.
382. Coffin, J.M.; Stoye, J.P. A new virus for old diseases? *Science (80-.).* **2009**, *326*, 530–531.
383. Cohen, H.; Neumann, L.; Glazer, Y.; Ebstein, R.P.; Buskila, D. The relationship between a common catechol-O-methyltransferase (COMT) polymorphism val(158)met and fibromyalgia. *Clin. Exp. Rheumatol.* **2009**, *27*, S51–S56.
384. Cohen, P. Tired of waiting. *Nurs. Times* **1997**, *93*, 29–31.
385. Colby, J. Special problems of children with myalgic encephalomyelitis/chronic fatigue syndrome and the enteroviral link. *J. Clin. Pathol.* **2007**, *60*, 125–128.
386. Collin, S.M.; Bakken, I.J.; Nazareth, I.; Crawley, E.; White, P.D. Trends in the incidence of chronic fatigue syndrome and fibromyalgia in the UK, 2001-2013: a Clinical Practice Research Datalink study. *J. R. Soc. Med.* **2017**, *110*, 231–244.
387. Collin, S.M.; Crawley, E.; May, M.T.; Sterne, J.A.C.; Hollingworth, W. The impact of CFS/ME on employment and productivity in the UK: a cross-sectional study based on the CFS/ME national outcomes database. *BMC Health Serv. Res.* **2011**, *11*, 217.
388. Collin, S.M.; Nijs, J.; Meeus, M.; Polli, A.; Willekens, B.; Ickmans, K. Endogenous Pain Facilitation Rather Than Inhibition Differs Between People with Chronic Fatigue Syndrome, Multiple Sclerosis, and Controls: An Observational Study. *Pain Physician* **2017**, *20*, E489–E497.
389. Collin, S.M.; Norris, T.; Deere, K.C.; Jago, R.; Ness, A.R.; Crawley, E. Physical activity at age 11 years and chronic disabling fatigue at ages 13 and 16 years in a UK birth cohort. *Arch. Dis. Child.* **2018**, *103*, 586–591.
390. Collin, S.M.; Norris, T.; Nuevo, R.; Tilling, K.; Joinson, C.; Sterne, J.A.C.; Crawley, E. Chronic Fatigue Syndrome at Age 16 Years. *Pediatrics* **2016**, *137*, e20153434.
391. Collin, S.M.; Nuevo, R.; van de Putte, E.M.; Nijhof, S.L.; Crawley, E. Chronic fatigue syndrome (CFS) or myalgic encephalomyelitis (ME) is different in children

compared to in adults: a study of UK and Dutch clinical cohorts. *BMJ Open* **2015**, *5*.
392. Collin, S.M.; Sterne, J.A.C.; Hollingworth, W.; May, M.T.; Crawley, E. Equity of access to specialist chronic fatigue syndrome (CFS/ME) services in England (2008-2010): a national survey and cross-sectional study. *BMJ Open* **2012**, *2*.
393. Collin, S.M.; Tilling, K.; Joinson, C.; Rimes, K.A.; Pearson, R.M.; Hughes, R.A.; Sterne, J.A.C.; Crawley, E. Maternal and childhood psychological factors predict chronic disabling fatigue at age 13 years. *J. Adolesc. Health* **2015**, *56*, 181–187.
394. Colloca, G.; Corsonello, A.; Marzetti, E.; Balducci, L.; Landi, F.; Extermann, M.; Scambia, G.; Cesari, M.; Carreca, I.; Monfardini, S.; et al. Treating Cancer in Older and Oldest Old Patients. *Curr. Pharm. Des.* **2015**, *21*, 1699–1705.
395. Collop, N.A. Can't sleep? You may have sleep apnea! *Chest* **2001**, *120*, 1768–1769.
396. Combs, S.E.; Wagner, J.; Bischof, M.; Welzel, T.; Edler, L.; Rausch, R.; Wagner, F.; Zabel-du Bois, A.; Debus, J.; Schulz-Ertner, D. Radiochemotherapy in Patients With Primary Glioblastoma Comparing Two Temozolomide Dose Regimens. *Int. J. Radiat. Oncol. Biol. Phys.* **2008**, *71*, 999–1005.
397. Comiskey, C.; Larkan, F. A national cross-sectional survey of diagnosed sufferers of myalgic encephalomyelitis/chronic fatigue syndrome: pathways to diagnosis, changes in quality of life and service priorities. *Ir. J. Med. Sci.* **2010**, *179*, 501–505.
398. Conti, F.; Magrini, L.; Priori, R.; Valesini, G.; Bonini, S. Eosinophil cationic protein serum levels and allergy in chronic fatigue syndrome. *Allergy Eur. J. Allergy Clin. Immunol.* **1996**, *51*, 124–127.
399. Conti, F.; Priori, R.; De Petrillo, G.; Rusconi, A.C.; Arpino, C.; Valesini, G. Prevalence of chronic fatigue syndrome in Italian patients with persistent fatigue. *Ann. Ital. di Med. interna organo Uff. della Soc. Ital. di Med. interna* **1994**, *9*, 219–222.
400. Cook, D.B.; O'Connor, P.J.; Lange, G.; Steffener, J. Functional neuroimaging correlates of mental fatigue induced by cognition among chronic fatigue syndrome patients and controls. *Neuroimage* **2007**, *36*, 108–122.
401. Cope, H.; Mann, A.; Pelosi, A.; David, A. Psychosocial risk factors for chronic fatigue and chronic fatigue syndrome following presumed viral illness: A case-control study. *Psychol. Med.* **1996**, *26*, 1197–1209.
402. Copeland, S.M. Unexpected findings and promoting monocausal claims, a cautionary tale. *J. Eval. Clin. Pract.* **2017**, *23*, 1055–1061.
403. Coplan, J.; Singh, D.; Gopinath, S.; Mathew, S.J.; Bulbena, A. A Novel Anxiety and Affective Spectrum Disorder of Mind and Body-The ALPIM (Anxiety-Laxity-Pain-Immune-Mood) Syndrome: A Preliminary Report. *J. Neuropsychiatry Clin. Neurosci.* **2015**, *27*, 93–103.
404. Coppens, E.; Van Wambeke, P.; Morlion, B.; Weltens, N.; Ly, H.G.; Tack, J.; Luyten, P.; Van Oudenhove, L. Prevalence and impact of childhood adversities and post-traumatic stress disorder in women with fibromyalgia andchronic widespread pain. *Eur. J. PAIN* **2017**, *21*, 1582–1590.
405. Corfield, E.C.; Martin, N.G.; Nyholt, D.R. Familiality and Heritability of Fatigue in an Australian Twin Sample. *TWIN Res. Hum. Genet.* **2017**, *20*, 208–215.
406. Cornelissen, M.; Zorgdrager, F.; Blom, P.; Jurriaans, S.; Repping, S.; van Leeuwen, E.; Bakker, M.; Berkhout, B.; van der Kuyl, A.C. Lack of Detection of XMRV in Seminal Plasma from HIV-1 Infected Men in The Netherlands. *PLoS One* **2010**, *5*.

407. Costigan, A.; Elliott, C.; McDonald, C.; Newton, J.L. Orthostatic symptoms predict functional capacity in chronic fatigue syndrome: implications for management. *QJM* **2010**, *103*, 589–595.
408. Coughlin, S.S.; McNeil, R.B.; Provenzale, D.T.; Dursa, E.K.; Thomas, C.M. Method issues in epidemiological studies of medically unexplained Symptom-based conditions in veterans. *J. Mil. Veterans. Health* **2013**, *21*, 4–10.
409. Coughlin, S.S.; Kang, H.K.; Mahan, C.M. Alcohol use and selected health conditions of 1991 Gulf War veterans: survey results, 2003-2005. *Prev. Chronic Dis.* **2011**, *8*, A52.
410. Coulter, P. Chronic Fatigue Syndrome: An Old Virus With a New Diagnosis. *J. Community Health Nurs.* **1988**, *5*, 87–95.
411. Courjaret, J.; Schotte, C.K.W.; Wijnants, H.; Moorkens, G.; Cosyns, P. Chronic fatigue syndrome and DSM-IV personality disorders. *J. Psychosom. Res.* **2009**, *66*, 13–20.
412. Coutts, R.; Weatherby, R.; Davie, A. The use of a symptom “self-report” inventory to evaluate the acceptability and efficacy of a walking program for patients suffering with chronic fatigue syndrome. *J. Psychosom. Res.* **2001**, *51*, 425–429.
413. Craddock, R.C.; Taylor, R.; Broderick, G.; Whistler, T.; Klimas, N.; Unger, E.R. Exploration of statistical dependence between illness parameters using the entropy correlation coefficient. *Pharmacogenomics* **2006**, *7*, 421–428.
414. Craig, A.; Tran, Y.; Siddall, P.; Wijesuriya, N.; Lovas, J.; Bartrop, R.; Middleton, J. Developing a model of associations between chronic pain, depressive mood, chronic fatigue, and self-efficacy in people with spinal cord injury. *J. Pain* **2013**, *14*, 911–920.
415. Cramer, C.R. Fibromyalgia and chronic fatigue syndrome: An update for athletic trainers. *J. Athl. Train.* **1998**, *33*, 359–361.
416. Crawley, E.; Collin, S.M.; White, P.D.; Rimes, K.; Sterne, J.A.C.; May, M.T.; Database, C.F.S.M.E.N.O. Treatment outcome in adults with chronic fatigue syndrome: a prospective study in England based on the CFS/ME National Outcomes Database. *QJM-AN Int. J. Med.* **2013**, *106*, 555–565.
417. Crawley, E.; Hughes, R.; Northstone, K.; Tilling, K.; Emond, A.; Sterne, J.A.C. Chronic disabling fatigue at age 13 and association with family adversity. *Pediatrics* **2012**, *130*, e71–e79.
418. Crawley, E.; Smith, G.D. Is chronic fatigue syndrome (CFS/ME) heritable in children, and if so, why does it matter? *Arch. Dis. Child.* **2007**, *92*, 1058–1061.
419. Crawley, E. Pediatric chronic fatigue syndrome: current perspectives. *Pediatr. Heal. Med. Ther.* **2018**, *9*, 27–33.
420. Crawley, E. The epidemiology of chronic fatigue syndrome/myalgic encephalitis in children. *Arch. Dis. Child.* **2014**, *99*, 171–174.
421. Crawley, E.; Hunt, L.; Stallard, P. Anxiety in children with CFS/ME. *Eur. Child Adolesc. Psychiatry* **2009**, *18*, 683–689.
422. Creavin, S.T.; Dunn, K.M.; Mallen, C.D.; Nijrolder, I.; van der Windt, D.A.W.M. Co-occurrence and associations of pain and fatigue in a community sample of Dutch adults. *Eur. J. Pain* **2010**, *14*, 327–334.
423. Creed, F.H.; Tomenson, B.; Chew-Graham, C.; Macfarlane, G.J.; Davies, I.; Jackson, J.; Littlewood, A.; McBeth, J. Multiple somatic symptoms predict impaired health status in functional somatic syndromes. *Int. J. Behav. Med.* **2013**, *20*, 194–205.

424. Creed, F.; Tomenson, B.; Chew-Graham, C.; Macfarlane, G.; McBeth, J. The associated features of multiple somatic symptom complexes. *J. Psychosom. Res.* **2018**, *112*, 1–8.
425. Crépeaux, G.; Gherardi, R.K.; Authier, F.-J. ASIA, chronic fatigue syndrome, and selective low dose neurotoxicity of aluminum adjuvants. *J. Allergy Clin. Immunol. Pract.* **2018**, *6*, 707–456.e1.
426. Creswell, C.; Chalder, T. Defensive coping styles in chronic fatigue syndrome. *J. Psychosom. Res.* **2001**, *51*, 607–610.
427. Creti, L.; Libman, E.; Baltzan, M.; Rizzo, D.; Bailes, S.; Fichten, C.S. Impaired sleep in chronic fatigue syndrome: how is it best measured? *J. Health Psychol.* **2010**, *15*, 596–607.
428. Crowhurst, G. XMRV: does this virus hold the key to myalgic encephalomyelitis/CFS? *Br. J. Nurs.* **2010**, *19*, 919–922.
429. Crum-Cianflone, N.F. Nonalcoholic fatty liver disease: An increasingly common cause of liver disease among HIV-infected persons? *AIDS Read.* **2007**, *17*, 513–518.
430. Csef, H. Communities of chronic fatigue syndrome, fibromyalgia and multiple chemical sensitivity [Gemeinsamkeiten von chronic fatigue syndrom, fibromyalgie und multipler chemischer sensitivitat]. *Dtsch. Medizinische Wochenschrift* **1999**, *124*, 163–169. Work in German
431. Csef, H. [Similarities of chronic fatigue syndrome, fibromyalgia and multiple chemical sensitivity]. *Dtsch. Med. Wochenschr.* **1999**, *124*, 163–169. Work in German
432. Cuende, J.I.; Civeira, P.; Diez, N.; Prieto, J. High prevalence without reactivation of herpes virus 6 in subjects with chronic fatigue syndrome [Alta prevalencia sin reactivación del herpesvirus humano-6 en sujetos con síndrome de fatiga crónica.]. *An. Med. Interna* **1997**, *14*, 441–444. Work in Spanish
433. Cuende, J.I.; Civeira, P.; Diez, N.; Prieto, J. [High prevalence without reactivation of herpes virus 6 in subjects with chronic fatigue syndrome]. *An. Med. Interna* **1997**, *14*, 441–444. Work in Spanish
434. Cui, X.; Lu, X.; Hisada, A.; Fujiwara, Y.; Katoh, T. The correlation between mental health and multiple chemical sensitivity: a survey study in Japanese workers. *Environ. Health Prev. Med.* **2015**, *20*, 123–129.
435. Cullen, W.; Kearney, Y.; Bury, G. Prevalence of fatigue in general practice. *Ir. J. Med. Sci.* **2002**, *171*, 10–12.
436. Currow, D.C.; Clark, K.; Kamal, A.; Collier, A.; Agar, M.R.; Lovell, M.R.; Phillips, J.L.; Ritchie, C. The Population Burden of Chronic Symptoms that Substantially Predate the Diagnosis of a Life-Limiting Illness. *J. Palliat. Med.* **2015**, *18*, 480–485.
437. Curt, G.A. Cancer related fatigue, An evolving priority in patient care [La sindrome da affaticamento da turnore maligno. Una crescente priorità nell’assistenza ai pazienti]. *Recenti Prog. Med.* **2001**, *92*, 408–412. Work in Italian
438. Curt, G.A. [Fatigue syndrome caused by malignant tumor. An increasing priority in patient care]. *Recenti Prog. Med.* **2001**, *92*, 408–412. Work in Italian
439. Cutler, S.J.; Bouzid, M.; Cutler, R.R. Q fever. *J. Infect.* **2007**, *54*, 313–318.
440. Cutolo, M.; Nobili, F.; Sulli, A.; Pizzorni, C.; Briata, M.; Faelli, F.; Vitali, P.; Mariani, G.; Copello, F.; Seriolo, B.; et al. Evidence of cerebral hypoperfusion in scleroderma patients. *Rheumatology* **2000**, *39*, 1366–1373.
441. Cvejic, E.; Lloyd, A.R.; Vollmer-Conna, U. Neurocognitive improvements after best-practice intervention for chronic fatigue syndrome: Preliminary evidence of

divergence between objective indices and subjective perceptions. *Compr. Psychiatry* **2016**, *66*, 166–175.
442. Dahan, H.; Shir, Y.; Nicolau, B.; Keith, D.; Allison, P. Self-Reported Migraine and Chronic Fatigue Syndrome Are More Prevalent in People with Myofascial vs Nonmyofascial Temporomandibular Disorders. *J. oral facial pain headache* **2016**, *30*, 7–13.
443. DAIKOS, G.K.; GARZONIS, S.; PALEOLOGUE, A.; BOUSVAROS, G.A.; PAPADOYANNAKIS, N. Benign myalgic encephalomyelitis: an outbreak in a nurses' school in Athens. *Lancet (London, England)* **1959**, *1*, 693–696.
444. Daly, E.; Komaroff, A.L.; Bloomingdale, K.; Wilson, S.; Albert, M.S. Neuropsychological function in patients with chronic fatigue syndrome, multiple sclerosis, and depression. *Appl. Neuropsychol.* **2001**, *8*, 12–22.
445. Daniels, J.; Brigden, A.; Kacorova, A. Anxiety and depression in chronic fatigue syndrome/myalgic encephalomyelitis (CFS/ME): Examining the incidence of health anxiety in CFS/ME. *Psychol. Psychother.* **2017**, *90*, 502–509.
446. Danielson, B.P.; Ayala, G.E.; Kimata, J.T. Detection of Xenotropic Murine Leukemia Virus-Related Virus in Normal and Tumor Tissue of Patients from the Southern United States with Prostate Cancer Is Dependent on Specific Polymerase Chain Reaction Conditions. *J. Infect. Dis.* **2010**, *202*, 1470–1477.
447. Dansie, E.J.; Furberg, H.; Afari, N.; Buchwald, D.; Edwards, K.; Goldberg, J.; Schur, E.; Sullivan, P.F. Conditions comorbid with chronic fatigue in a population-based sample. *Psychosomatics* **2012**, *53*, 44–50.
448. Dansie, E.J.; Heppner, P.; Furberg, H.; Goldberg, J.; Buchwald, D.; Afari, N. The Comorbidity of Self-Reported Chronic Fatigue Syndrome, Post-Traumatic Stress Disorder, and Traumatic Symptoms. *Psychosomatics* **1970**, *53*, 250–257.
449. Dantoft, T.M.; Ebstrup, J.F.; Linneberg, A.; Skovbjerg, S.; Madsen, A.L.; Mehlsen, J.; Brinth, L.; Eplov, L.F.; Carstensen, T.W.; Schroder, A.; et al. Cohort description: The Danish study of functional disorders. *Clin. Epidemiol.* **2017**, *9*, 127–139.
450. Dantoft, T.M.; Skovbjerg, S.; Andersson, L.; Claeson, A.-S.; Engkilde, K.; Lind, N.; Nordin, S.; Hellgren, L.I. Gene expression profiling in persons with multiple chemical sensitivity before and after a controlled n-butanol exposure session. *BMJ Open* **2017**, *7*.
451. Darbishire, L.; Ridsdale, L.; Seed, P.T. Distinguishing patients with chronic fatigue from those with chronic fatigue syndrome: a diagnostic study in UK primary care. *Br. J. Gen. Pract.* **2003**, *53*, 441–445.
452. Daugherty, S.A.; Henry, B.E.; Peterson, D.L.; Swarts, R.L.; Bastien, S.; Thomas, R.S. Chronic fatigue syndrome in northern Nevada. *Rev. Infect. Dis.* **1991**, *13 Suppl 1*, S39-44.
453. David, A.S. Postviral fatigue syndrome and psychiatry. *Br. Med. Bull.* **1991**, *47*, 966–988.
454. DAVID, A.S.; WESSELY, S.; PELOSI, A.J. CHRONIC FATIGUE SYNDROME - SIGNS OF A NEW APPROACH. *Br. J. Hosp. Med.* **1991**, *45*, 158–163.
455. David, A.S.; Wessely, S.; Pelosi, A.J. Postviral fatigue syndrome: Time for a new approach. *Br. Med. J. (Clin. Res. Ed).* **1988**, *296*, 696–699.
456. David, A.; Pelosi, A.; McDonald, E.; Stephens, D.; Ledger, D.; Rathbone, R.; Mann, A. Tired, weak, or in need of rest: fatigue among general practice attenders. *BMJ* **1990**, *301*, 1199–1202.
457. Davies, M.; Wilton, L.; Shakir, S. Safety profile of modafinil across a range of prescribing indications, including off-label use, in a primary care setting in

England: Results of a modified prescription-event monitoring study. *Drug Saf.* **2013**, *36*, 237–246.
458. Davies, S.; Crawley, E. Chronic fatigue syndrome in children aged 11 years old and younger. *Arch. Dis. Child.* **2008**, *93*, 419–422.
459. Davis, A.K.; Barsuglia, J.P.; Lancelotta, R.; Grant, R.M.; Renn, E. The epidemiology of 5-methoxy-N, N-dimethyltryptamine (5-MeO-DMT) use: Benefits, consequences, patterns of use, subjective effects, and reasons for consumption. *J. Psychopharmacol.* **2018**, *32*, 779–792.
460. De Becker, P.; McGregor, N.; De Meirleir, K. A definition-based analysis of symptoms in a large cohort of patients with chronic fatigue syndrome. *J. Intern. Med.* **2001**, *250*, 234–240.
461. De Gucht, W.; Maes, S. Explaining medically unexplained symptoms: Toward a multidimensional, theory-based approach to somatization. *J. Psychosom. Res.* **2006**, *60*, 349–352.
462. de Jong, L.W.; Prins, J.B.; Fiselier, T.J.; Weemaes, C.M.; Meijer-van den Bergh, E.M.; Bleijenberg, G. [Chronic fatigue syndrome in young persons]. *Ned. Tijdschr. Geneeskd.* **1997**, *141*, 1513–1516. Work in Dutch
463. De Jong, L.W.A.M.; Prins, J.B.; Fiselier, T.J.W.; Weemaes, C.M.R.; Meijer-Van Den Bergh, E.M.M.; Bleijenberg, G. Chronic fatigue syndrome in adolescence [Het chronische-vermoeidheidsyndroom bij jongeren]. *Ned. Tijdschr. Geneeskd.* **1997**, *141*, 1513–1516. Work in Dutch
464. de Korwin, J.-D.; Chiche, L.; Banovic, I.; Ghali, A.; Delliaux, S.; Authier, F.-J.; Cozon, G.; Hatron, P.-Y.; Fornasieri, I.; Morinet, F. Chronic fatigue syndrome: A new disorder? *Rev. Med. INTERNE* **2016**, *37*, 811–819.
465. de León, F.; Gutiérrez Fernández, J.; Martín Mazuelos, E.; García-Bragado, F. Infection by human herpesvirus type 6: epidemiology, immunopathology and clinical implications [Infección por virus herpes humano 6: epidemiología, inmunopatología e implicaciones clínicas.]. *Rev. clínica española* **1992**, *190*, 37–42. Work in Spanish
466. De Lorenzo, F. Phosphate diabetes in patients with chronic fatigue syndrome. *Postgrad. Med. J.* **1998**, *74*, 229–232.
467. de Luca, C.; Raskovic, D.; Pacifico, V.; Thai, J.C.S.; Korkina, L. The search for reliable biomarkers of disease in multiple chemical sensitivity and other environmental intolerances. *Int. J. Environ. Res. Public Health* **2011**, *8*, 2770–2797.
468. de Luca, C.; Scordo, G.; Cesareo, E.; Raskovic, D.; Genovesi, G.; Korkina, L. Idiopathic environmental intolerances (IEI): From molecular epidemiology to molecular medicine. *Indian J. Exp. Biol.* **2010**, *48*, 625–635.
469. De Meirleir, K.; Bisbal, C.; Campine, I.; De Becker, P.; Salehzada, T.; Demettre, E.; Lebleu, B. A 37 kDa 2-5A binding protein as a potential biochemical marker for chronic fatigue syndrome. *Am. J. Med.* **2000**, *108*, 99–105.
470. De Meirleir, K.; McGregor, N. Journal of Chronic Fatique Syndrome: Editorial. *J. Chronic Fatigue Syndr.* **2003**, *11*, 1–5.
471. De Sanctis, V.; Mangiagli, A.; Campisi, S.; Raiola, G. [Asthenia in adolescents]. *Minerva Pediatr.* **2002**, *54*, 631–637. Work in Italian
472. de Serres, F.; Blanco, I. Role of alpha-1 antitrypsin in human health and disease. *J. Intern. Med.* **2014**, *276*, 311–335.
473. De Simone, R.; Ranieri, A.; Sansone, M.; Marano, E.; Russo, C.V.; Sacca, F.; Bonavita, V. Dural sinus collapsibility, idiopathic intracranial hypertension, and

the pathogenesis of chronic migraine. *Neurol. Sci.* **2019**, *40*, S59–S70. Proceedings of the International Multidisciplinary Seminar (Stresa Headache) in Stresa, Italy.
474. De Souza, M.D.F.M.; Messing, K.; Menezes, P.R.; Cho, H.J. Chronic fatigue among bank workers in Brazil. *Occup. Med. (Chic. Ill).* **2002**, *52*, 187–194.
475. De Tommaso, M. Prevalence, clinical features and potential therapies for fibromyalgia in primary headaches. *Expert Rev. Neurother.* **2012**, *12*, 287–296.
476. De Toni, T.; Calvillo, M. [Adolescents and the problem of a chronic disease]. *Minerva Pediatr.* **2001**, *53*, 383–389. Work in Spanish
477. De Venter, M.; Van Den Eede, F. Childhood trauma as a predisposing factor in fibromyalgia and chronic fatigue syndrome: Literature review [Jeugdtrauma als voorbeschikkende factor voor fibromyalgie en het chronischevermoeidheidssyndroom: Een literatuuroverzicht]. *Tijdschr. Geneeskd.* **2013**, *69*, 912–921. Work in Dutch
478. De Vries, E.; Fransen, L.; Van Den Aker, M.; Meijboom, B.R. Preventing gatekeeping delays in the diagnosis of rare diseases. *Br. J. Gen. Pract.* **2018**, *68*, 145–146.
479. De Vries, M.; Soetekouw, P.M.; Van Der Meer, J.W.; Bleijenberg, G. Natural course of symptoms in Cambodia veterans: a follow-up study. *Psychol. Med.* **2001**, *31*, 331–338.
480. Deák, M.; Szvetnik, A.; Balog, A.; Sohár, N.; Varga, R.; Pokorny, G.; Tóth, G.; Kiss, M.; Kovács, L. Neuroimmune interactions in Sjögren's syndrome: Relationship of exocrine gland dysfunction with autoantibodies to muscarinic acetylcholine receptor-3 and mental health status parameters. *Neuroimmunomodulation* **2013**, *20*, 79–86.
481. Deandres-Galiana, E.J.; Fernandez-Martinez, J.L.; Saligan, L.N.; Sonis, S.T. Impact of Microarray Preprocessing Techniques in Unraveling Biological Pathways. *J. Comput. Biol.* **2016**, *23*, 957–968.
482. Deary, V.; Chalder, T.; Sharpe, M. The cognitive behavioural model of medically unexplained symptoms: a theoretical and empirical review. *Clin. Psychol. Rev.* **2007**, *27*, 781–797.
483. Delahanty, D.L. Toward the predeployment detection of risk for PTSD. *Am. J. Psychiatry* **2011**, *168*, 9–11.
484. DELEON, F.L.; FERNANDEZ, J.G.; MAZUELOS, E.M.; GARCIABRAGADO, F. INFECTION WITH HUMAN HERPESVIRUS TYPE-6 - EPIDEMIOLOGY, IMMUNOPATHOLOGY AND CLINICAL IMPLICATIONS. *Rev. Clin. Esp.* **1992**, *190*, 37–42.
485. Dell'Osso, L.; Carmassi, C.; Consoli, G.; Conversano, C.; Ramacciotti, C.E.; Musetti, L.; Massimetti, E.; Pergentini, I.; Corsi, M.; Ciapparelli, A.; et al. Lifetime post-traumatic stress symptoms are related to the health-related quality of life and severity of pain/fatigue in patients with fibromyalgia. *Clin. Exp. Rheumatol.* **2011**, *29*, S73–S78.
486. Delsing, C.E.; Kullberg, B.J. Q fever in the Netherlands: A concise overview and implications of the largest ongoing outbreak. *Neth. J. Med.* **2008**, *66*, 365–367.
487. Deluca, J.; Johnson, S.K.; Natelson, B.H. Neuropsychiatric status of patients with chronic fatigue syndrome: an overview. *Toxicol. Ind. Health* **1994**, *10*, 513–522.
488. Deluca, J.; Christodoulou, C.; Diamond, B.J.; Rosenstein, E.D.; Kramer, N.; Natelson, B.H. Working memory deficits in chronic fatigue syndrome: differentiating between speed and accuracy of information processing. *J. Int. Neuropsychol. Soc.* **2004**, *10*, 101–109.

489. Demitrack, M.A. Clinical methodology and its implications for the study of therapeutic interventions for chronic fatigue syndrome: a commentary. *Pharmacogenomics* **2006**, *7*, 521–528.
490. den Boeft, M.; Huisman, D.; van der Wouden, J.C.; Numans, M.E.; van der Horst, H.E.; Lucassen, P.L.; Hartman, T.C.O. Recognition of patients with medically unexplained physical symptoms by family physicians: results of a focus group study. *BMC Fam. Pract.* **2016**, *17*.
491. Denz-Penhey, H.; Murdoch, J.C.; DENZPENHEY, H.; Murdoch, J.C. General practitioners acceptance of the validity of chronic fatigue syndrome as a diagnosis. *N. Z. Med. J.* **1993**, *106*, 122–124.
492. Devanur, L.D.; Kerr, J.R. Chronic fatigue syndrome. *J. Clin. Virol.* **2006**, *37*, 139–150.
493. Di Luca, D.; Zorzenon, M.; Mirandola, P.; Colle, R.; Botta, G.A.; Cassai, E. Human herpesvirus 6 and human herpesvirus 7 in chronic fatigue syndrome. *J. Clin. Microbiol.* **1995**, *33*, 1660–1661.
494. Dick, H.; Vanderfaeillie, J.; Marchand, J.; Lampo, A. Psychiatric disorders in youngsters with chronic fatigue syndrome and implications towards treatment [Psychiatrische stoornissen bij jongeren met het chronischevermoeidheidssyndroom en implicaties inzake behandeling]. *Tijdschr. Geneeskd.* **2008**, *64*, 118–125. Work in Dutch
495. Dickey, R.W.; Plakas, S.M. Ciguatera: A public health perspective. *TOXICON* **2010**, *56*, 123–136.
496. DiClementi, J.D.; Schmaling, K.B.; Jones, J.F. Information processing in chronic fatigue syndrome: A preliminary investigation of suggestibility. *J. Psychosom. Res.* **2001**, *51*, 679–686.
497. DIEHL, D.L.; FULLERTON, S.; MAYER, E.A. FUNCTIONAL BOWEL-DISEASE SYMPTOMS IN PATIENTS WITH CHRONIC FATIGUE SYNDROME (CFS) - PREVALENCE AND QUALITY-OF-LIFE. *Gastroenterology* **1995**, *108*, A592.
498. Dietel, A.; Jordan, L.; Muhlinghaus, T.; Eikmann, T.F.; Herr, C.E.W.; Nowak, D.; Gil, F.P.; Podoll, K.; Wiesmuller, G.A.; Eis, D. Psychiatric disorders of environmental outpatients results of the standardized psychiatric interview (CIDI) from the German multi-center study on multiple chemical sensitivity (MCS). *Psychother. Psychosom. MEDIZINISCHE Psychol.* **2006**, *56*, 162–171.
499. Dietert, R.R.; Zelikoff, J.T. Pediatric immune dysfunction and health risks following early-life immune insult. *Curr. Pediatr. Rev.* **2009**, *5*, 36–51.
500. DiLuca, D.; Mirandola, P.; Ravaioli, T.; Bigoni, B.; Cassai, E. Distribution of HHV-6 variants in human tissues. *Infect. AGENTS Dis. ISSUES Comment.* **1996**, *5*, 203–214.
501. DILUCA, D.; ZORZENON, M.; MIRANDOLA, P.; COLLE, R.; BOTTA, G.A.; CASSAI, E. HUMAN HERPESVIRUS-6 AND HUMAN HERPESVIRUS-7 IN CHRONIC FATIGUE SYNDROME. *J. Clin. Microbiol.* **1995**, *33*, 1660–1661.
502. Dinos, S.; Khoshaba, B.; Ashby, D.; White, P.D.; Nazroo, J.; Wessely, S.; Bhui, K.S. A systematic review of chronic fatigue, its syndromes and ethnicity: prevalence, severity, co-morbidity and coping. *Int. J. Epidemiol.* **2009**, *38*, 1554–1570.
503. Dirkzwager, A.J.E.; Verhaak, P.F.M. Patients with persistent medically unexplained symptoms in general practice: characteristics and quality of care. *BMC Fam. Pract.* **2007**, *8*.

504. Dittner, A.J.; Rimes, K.; Thorpe, S. Negative perfectionism increases the risk of fatigue following a period of stress. *Psychol. Health* **2011**, *26*, 253–268.
505. Dixit, R.; Popescu, A.; Bagic, A.; Ghearing, G.; Hendrickson, R. Medical comorbidities in patients with psychogenic nonepileptic spells (PNES) referred for video-EEG monitoring. *EPILEPSY Behav.* **2013**, *28*, 137–140.
506. Dobbins, J.G.; Randall, B.; Reyes, M.; Steele, L.; Livens, E.A.; Reeves, W.C. The prevalence of chronic fatiguing illnesses among adolescents in the United States. *J. Chronic Fatigue Syndr.* **1997**, *3*, 15–28.
507. Dobke, M.K.; Svahn, J.K.; Vastine, V.L.; Landon, B.N.; Stein, P.C.; Parsons, C.L.; Burkhardt, B.R. Characterization of microbial presence at the surface of silicone mammary implants. *Ann. Plast. Surg.* **1995**, *34*, 563–571.
508. Dodd, R.Y. Chronic fatigue syndrome, XMRV and blood safety. *Future Microbiol.* **2011**, *6*, 385–389.
509. Dodd, R.Y.; Hackett, J.J.; Linnen, J.M.; Dorsey, K.; Wu, Y.; Zou, S.; Qiu, X.; Swanson, P.; Schochetman, G.; Gao, K.; et al. Xenotropic murine leukemia virus-related virus does not pose a risk to blood recipient safety. *Transfusion* **2012**, *52*, 298–306.
510. Doebbeling, B.N.; Clarke, W.R.; Watson, D.; Torner, J.C.; Woolson, R.F.; Voelker, M.D.; Barrett, D.H.; Schwartz, D.A. Will we solve the Gulf War syndrome puzzle by population surveys or clinical research? Reply. *Am. J. Med.* **2000**, *109*, 745–748.
511. Doebbeling, B.N.; Jones, M.F.; Hall, D.B.; Clarke, W.R.; Woolson, R.F.; Torner, J.C.; Burmeister, L.F.; Snyders-Crumley, T.; Barrett, D.H.; Falter, K.H.; et al. Methodologic issues in a population-based health survey of Gulf War veterans. *J. Clin. Epidemiol.* **2002**, *55*, 477–487.
512. Dogru, M.T.; Aydin, G.; Tosun, A.; Keles, I.; Gueneri, M.; Arslan, A.; Ebinc, H.; Orkun, S. Correlations between autonomic dysfunction and circadian changes and arrhythmia prevalence in women with fibromyalgia syndrome. *Anatol. J. Cardiol.* **2009**, *9*, 110–117.
513. Domenech-Llaberia, E.; Jane, M.C.; Canals, J.; Ballespi, S.; Esparo, G.; Garralda, E. Parental reports of somatic symptoms in preschool children: Prevalence and associations in a Spanish sample. *J. Am. Acad. CHILD Adolesc. PSYCHIATRY* **2004**, *43*, 598–604.
514. Donegan, K.; Beau-Lejdstrom, R.; King, B.; Seabroke, S.; Thomson, A.; Bryan, P. Bivalent human papillomavirus vaccine and the risk of fatigue syndromes in girls in the UK. *Vaccine* **2013**, *31*, 4961–4967.
515. Donnachie, E.; Schneider, A.; Mehring, M.; Enck, P. Incidence of irritable bowel syndrome and chronic fatigue following GI infection: a population-level study using routinely collected claims data. *Gut* **2018**, *67*, 1078–1086.
516. Donnay, A.H. On the recognition of multiple chemical sensitivity in medical literature and government policy. *Int. J. Toxicol.* **1999**, *18*, 383–392.
517. Donnay, A.; Ziem, G. Prevalence and overlap of chronic fatigue syndrome and fibromyalgia syndrome among 100 new patients with multiple chemical sensitivity syndrome. *J. Chronic Fatigue Syndr.* **1999**, *5*, 71–80.
518. Donner-Banzhoff, N.; Kunz, R.; Rosser, W. Studies of symptoms in primary care. *Fam. Pract.* **2001**, *18*, 33–38.
519. Donta, S.T.; Engel, C.C.; Collins, J.F.; Baseman, J.B.; Dever, L.L.; Taylor, T.; Boardman, K.D.; Kazis, L.E.; Martin, S.E.; Horney, R.A.; et al. Benefits and harms of doxycycline treatment for Gulf War veterans' illnesses. *Ann. Intern. Med.* **2004**, *141*, 85–94.

520. Dougall, D.; Johnson, A.; Goldsmith, K.; Sharpe, M.; Angus, B.; Chalder, T.; White, P. Adverse events and deterioration reported by participants in the PACE trial of therapies for chronic fatigue syndrome. *J. Psychosom. Res.* **2014**, *77*, 20–26.
521. Douzenis, A.; Seretis, D. Descriptive and predictive validity of somatic attributions in patients with somatoform disorders: A systematic review of quantitative research. *J. Psychosom. Res.* **2013**, *75*, 199–210.
522. Downey, D.C. Fatigue syndromes: new thoughts and reinterpretation of previous data. *Med. Hypotheses* **1992**, *39*, 185–190.
523. Dowsett, E.G.; Colby, J. Long-term sickness absence due to ME/CFS in UK schools: An epidemiological study with medical and educational implications. *J. Chronic Fatigue Syndr.* **1997**, *3*, 29–42.
524. Dowsett, E.G.; Ramsay, A.M.; McCartney, R.A.; Bell, E.J. Myalgic encephalomyelitis--a persistent enteroviral infection? *Postgrad. Med. J.* **1990**, *66*, 526–530.
525. Doyle, J.P.; Frank, E.; Saltzman, L.E.; McMahon, P.M.; Fielding, B.D. Domestic violence and sexual abuse in women physicians: Associated medical, psychiatric and professional difficulties. *J. Women's Heal. Gender-Based Med.* **1999**, *8*, 955–965.
526. DuBuske, L.; Markov, A.; Sheffer, A.; Schacterle, R.; Komaroff, A.; Schur, P. Prevalence of atopy among patients with chronic fatigue syndrome (CFS). *J. Allergy Clin. Immunol.* **1998**, *101*, S170.
527. Duclos, P. Safety of immunization and adverse events following vaccination against hepatitis B. *J. Hepatol.* **2003**, *39*, S83–S88.
528. Duncan, R.B. Latency immunity and therapy: A clinical study of latent Epstein Barr Virus Incidence in 297 idiopathic chronic fatigue patients with plausible hypotheses. *J. Chronic Fatigue Syndr.* **1999**, *5*, 77–95.
529. Dunstan, R.H.; Donohoe, M.; Taylor, W.; Roberts, T.K.; Murdoch, R.N.; Watkins, J.A.; McGregor, N.R. Chlorinated hydrocarbons and chronic fatigue syndrome. *Med. J. Aust.* **1996**, *164*, 251.
530. Dunstan, R.H.; Donohoe, M.; Taylor, W.; Roberts, T.K.; Murdoch, R.N.; Watkins, J.A.; McGregor, N.R. A preliminary investigation of chlorinated hydrocarbons and chronic fatigue syndrome. *Med. J. Aust.* **1995**, *163*, 294–297.
531. Dunstan, R.H.; McGregor, N.R.; Butt, H.L.; Roberts, T.K. Biochemical and microbiological anomalies in chronic fatigue syndrome: The development of laboratory based tests and the possible role of toxic chemicals. *J. Nutr. Environ. Med.* **1999**, *9*, 97–108.
532. Durand, D. V; François, S.; Nové-Josserand, R.; Durupt, S.; Durieu, I.; Morel, Y.; Rousset, H. Haemochromatosis screening in 120 patients complaining with persistant fatigue [Dépistage de l'hémochromatose chez 120 sujets consultant pour une asthénie chronique]. *Rev. Med. Interne* **2004**, *25*, 623–628. Work in French
533. Durham, J.; McDonald, C.; Hutchinson, L.; Newton, J.L. Painful temporomandibular disorders are common in patients with postural orthostatic tachycardia syndrome and impact significantly upon quality of life. *J. oral facial pain headache* **2015**, *29*, 152–157.
534. Dürrwald, R.; Kolodziejek, J.; Herzog, S.; Nowotny, N. Meta-analysis of putative human bornavirus sequences fails to provide evidence implicating Borna disease virus in mental illness. *Rev. Med. Virol.* **2007**, *17*, 181–203.
535. Duvignaud, A.; Fianu, A.; Bertolotti, A.; Jaubert, J.; Michault, A.; Poubeau, P.; Fred, A.; Mechain, M.; Gauzere, B.-A.; Favier, F.; et al. Rheumatism and chronic

fatigue, the two facets of post-chikungunya disease: the TELECHIK cohort study on Reunion island. *Epidemiol. Infect.* **2018**, *146*, 633–641.
536. Dyck, D.; Allen, S.; Barron, J.; Marchi, J.; Price, B.-A.; Spavor, L.; Tateishi, S. Management of chronic fatigue syndrome: Case study. *AAOHN J.* **1996**, *44*, 85–92.
537. Earl, K.E.; Sakellariou, G.K.; Sinclair, M.; Fenech, M.; Croden, F.; Owens, D.J.; Tang, J.; Miller, A.; Lawton, C.; Dye, L.; et al. Vitamin D status in chronic fatigue syndrome/myalgic encephalomyelitis: a cohort study from the North-West of England. *BMJ Open* **2017**, *7*.
538. Edgcomb, J.B.; Tseng, C.-H.; Kerner, B. Medically unexplained somatic symptoms and bipolar spectrum disorders: A systematic review and meta-analysis. *J. Affect. Disord.* **2016**, *204*, 205–213.
539. Edmonds, M.; Mcguire, H.; Price, J.R. Exercise therapy for chronic fatigue syndrome. *Cochrane Database Syst. Rev.* **2004**, *2004*.
540. Edwards, R.H.; Gibson, H.; Clague, J.E.; Helliwell, T. Muscle histopathology and physiology in chronic fatigue syndrome. *Ciba Found. Symp.* **1993**, *173*, 102–131.
541. Efficace, F.; Baccarani, M.; Breccia, M.; Cottone, F.; Alimena, G.; Deliliers, G.L.; Barate, C.; Specchia, G.; Di Lorenzo, R.; Luciano, L.; et al. Chronic fatigue is the most important factor limiting health-related quality of life of chronic myeloid leukemia patients treated with imatinib. *Leukemia* **2013**, *27*, 1511–1519.
542. Egerman, R.S. Mononucleosis. *Prim. Care Update Ob. Gyns.* **1995**, *2*, 152–156.
543. Eglinton, R.; Chung, M.C. The relationship between posttraumatic stress disorder, illness cognitions, defence styles, fatigue severity and psychological well-being in chronic fatigue syndrome. *Psychiatry Res.* **2011**, *188*, 245–252.
544. Einecke, U. Practical problem of somatoform disorders: How to help problem patients [Praxisproblem somatoforme störungen: So helfen sie "lästigen" patienten]. *MMW-Fortschritte der Medizin* **2009**, *151*, 20–21. Work in German
545. Eis, D. ``Multiple Chemical Sensitivity{''} (MCS) epidemiology. *ALLERGOLOGIE* **1999**, *22*, 538–554.
546. Eisen, S.A.; Karlinsky, J.; Jackson, L.W.; Blanchard, M.; Kang, H.K.; Murphy, F.M.; Alpern, R.; Reda, D.J.; Toomey, R.; Battistone, M.J.; et al. Spouses of Persian Gulf War I veterans: Medical evaluation of a U.S. cohort. *Mil. Med.* **2006**, *171*, 613–618.
547. Eisen, S.A.; Kang, H.K.; Murphy, F.M.; Blanchard, M.S.; Reda, D.J.; Henderson, W.G.; Toomey, R.; Jackson, L.W.; Alpern, R.; Parks, B.J.; et al. Gulf War veterans' health: medical evaluation of a U.S. cohort. *Ann. Intern. Med.* **2005**, *142*, 881–890.
548. Eldin, C.; Melenotte, C.; Mediannikov, O.; Ghigo, E.; Million, M.; Edouard, S.; Mege, J.-L.; Maurin, M.; Raoult, D. From Q Fever to Coxiella burnetii Infection: a Paradigm Change. *Clin. Microbiol. Rev.* **2017**, *30*, 115–190.
549. Elkins, L.E.; Pollina, D.A.; Scheffer, S.R.; Krupp, L.B. Psychological states and neuropsychological performances in chronic Lyme disease. *Appl. Neuropsychol.* **1999**, *6*, 19–26.
550. Elliott, H. Use of formal and informal care among people with prolonged fatigue: a review of the literature. *Br. J. Gen. Pract.* **1999**, *49*, 131–134.
551. Elwood, J.M.; Ameratunga, R. Autoimmune diseases after hepatitis B immunization in adults: Literature review and meta-analysis, with reference to `autoimmune/autoinflammatory syndrome induced by adjuvants' (ASIA). *Vaccine* **2018**, *36*, 5796–5802.

552. Eminson, D.M. Medically unexplained symptoms in children and adolescents. *Clin. Psychol. Rev.* **2007**, *27*, 855–871.
553. Enbom, M.; Linde, A.; Evengard, B. No evidence of active infection with human herpesvirus 6 (HHV-6) or HHV-8 in chronic fatigue syndrome. *J. Clin. Microbiol.* **2000**, *38*, 2457.
554. Endicott, N.A. Chronic fatigue syndrome in private practice psychiatry: Family history of physical and mental health. *J. Psychosom. Res.* **1999**, *47*, 343–354.
555. Endicott, N.A. Chronic fatigue syndrome in psychiatric patients: lifetime and premorbid personal history of physical health. *Psychosom. Med.* **1998**, *60*, 744–751.
556. Engel, C.C. Unexplained physical symptoms: Medicine's ``dirty little secret{''} and the need for prospective studies that start in childhood. *PSYCHIATRY-INTERPERSONAL Biol. Process.* **2000**, *63*, 153–159.
557. Engel, C.C.; Liu, M.; Hoge, C.; Smith, S. Multiple idiopathic physical symptoms in the ECA study: Competing-risks analysis of 1-year incidence, mortality, and resolution. *Am. J. Psychiatry* **2002**, *159*, 998–1004.
558. Enserink, M. Chronic fatigue syndrome. CDC struggles to recover from debacle over earmark. *Science* 2000, *287*, 22–23.
559. Enserink, M. Chronic fatigue syndrome. New XMRV paper looks good, skeptics admit--yet doubts linger. *Science* 2010, *329*, 1000.
560. Epstein, S.A.; Kay, G.; Clauw, D.; Heaton, R.; Klein, D.; Krupp, L.; Kuck, J.; Leslie, V.; Masur, D.; Wagner, M.; et al. Psychiatric disorders in patients with fibromyalgia - A multicenter investigation. *Psychosomatics* **1999**, *40*, 57–63. Proceedings of the 44th Annual Meeting of the Academy-of-Psychosomatic-Medicine in San Diego, USA.
561. Eriksen, H.R.; Ursin, H. Subjective health complaints, sensitization, and sustained cognitive activation (stress). *J. Psychosom. Res.* **2004**, *56*, 445–448.
562. Eriksen, T.E.; Kirkengen, A.L.; Vetlesen, A.J. The medically unexplained revisited. *Med. Heal. CARE Philos.* **2013**, *16*, 587–600.
563. Erlwein, O.; Kaye, S.; McClure, M.O.; Weber, J.; Wills, G.; Collier, D.; Wessely, S.; Cleare, A. Failure to detect the novel retrovirus XMRV in chronic fatigue syndrome. *PLoS One* **2010**, *5*.
564. Erlwein, O.; Robinson, M.J.; Dustan, S.; Weber, J.; Kaye, S.; McClure, M.O. DNA extraction columns contaminated with murine sequences. *PLoS One* **2011**, *6*.
565. Erlwein, O.; Robinson, M.J.; Kaye, S.; Wills, G.; Izui, S.; Wessely, S.; Weber, J.; Cleare, A.; Collier, D.; McClure, M.O. Investigation into the Presence of and Serological Response to XMRV in CFS Patients. *PLoS One* **2011**, *6*.
566. Escobar, J.I. Pharmacological treatment of somatization/hypochondriasis overview of somatization: Diagnosis, epidemiology, and management. *Psychopharmacol. Bull.* **1996**, *32*, 589–596. Proceedings of the 36th Annual Meeting of the New-Clinical-Drug-Evaluation-Unit in Boca Raton, USA.
567. Esposito, S.; Principi, N.; Cornaglia, G. Barriers to the vaccination of children and adolescents and possible solutions. *Clin. Microbiol. Infect.* **2014**, *20*, 25–31.
568. Essau, C.A. Comorbidity of depressive disorders among adolescents in community and clinical settings. *PSYCHIATRY Res.* **2008**, *158*, 35–42.
569. Estévez-López, F.; Castro-Marrero, J.; Wang, X.; Bakken, I.J.; Ivanovs, A.; Nacul, L.; Sepúlveda, N.; Strand, E.B.; Pheby, D.; Alegre, J.; et al. Prevalence and incidence of myalgic encephalomyelitis/chronic fatigue syndrome in Europe - the Euro-epiME study from the European network EUROMENE: A protocol for a systematic review. *BMJ Open* **2018**, *8*.

570. Estevez-Lopez, F.; Castro-Marrero, J.; Wang, X.; Bakken, I.J.; Ivanovs, A.; Nacul, L.; Sepulveda, N.; Strand, E.B.; Pheby, D.; Alegre, J.; et al. Prevalence and incidence of myalgic encephalomyelitis/chronic fatigue syndrome in Europethe Euro-epiME study from the European network EUROMENE: a protocol for a systematic review. *BMJ Open* **2018**, *8*.
571. Evengård, B.; Jacks, A.; Pedersen, N.L.; Sullivan, P.F.; Evengard, B.; Jacks, A.; Pedersen, N.L.; Sullivan, P.F. The epidemiology of chronic fatigue in the Swedish Twin Registry. *Psychol. Med.* **2005**, *35*, 1317–1326.
572. Evengard, B.; Jonzon, E.; Sandberg, A.; Theorell, T.; Lindh, G. Differences between patients with chronic fatigue syndrome and with chronic fatigue at an infectious disease clinic in Stockholm, Sweden. *PSYCHIATRY Clin. Neurosci.* **2003**, *57*, 361–368.
573. Evengård, B.; Lipkin, W. A known viral disease in animals is suspected in humans; BDV has been detected in cases of human neuropathy [Känt virus hos djur misstänks hos människa: Borna disease-virus har spårats vid human neurosjukdom]. *Lakartidningen* **1997**, 4753–4756. Work in Swedish
574. Evengard, B.; Nilsson, C.G.; Lindh, G.; Lindquist, L.; Eneroth, P.; Fredrikson, S.; Terenius, L.; Henriksson, K.G. Chronic fatigue syndrome differs from fibromyalgia. No evidence for elevated substance P levels in cerebrospinal fluid of patients with chronic fatigue syndrome. *Pain* **1998**, *78*, 153–155.
575. Evengard, B.; Sachs, L. [CFS, the condition with the thousands different names. "The tired nomad"--a modern epidemic?]. *Lakartidningen* **1994**, *91*, 4001–4005. Work in Swedish
576. Evengård, B.; Schacterle, R.S.; Komaroff, A.L. Chronic fatigue syndrome: New insights and old ignorance. *J. Intern. Med.* **1999**, *246*, 455–469.
577. Evengard, B.; Klimas, N.; Evengård, B.; Klimas, N. Chronic fatigue syndrome: Probable pathogenesis and possible treatments. *Drugs* **2002**, *62*, 2433–2446.
578. Evering, R.M.H.; Tonis, T.M.; Vollenbroek-Hutten, M.M.R. Deviations in daily physical activity patterns in patients with the chronic fatigue syndrome: a case control study. *J. Psychosom. Res.* **2011**, *71*, 129–135.
579. Evering, R.M.H.; van Weering, M.G.H.; Groothuis-Oudshoorn, K.C.G.M.; Vollenbroek-Hutten, M.M.R. Daily physical activity of patients with the chronic fatigue syndrome: a systematic review. *Clin. Rehabil.* **2011**, *25*, 112–133.
580. Eyigor, S.; Ozdedeli, S.; Durmaz, B. The prevalence of generalized soft tissue rheumatic conditions in Turkish medical students. *J. Clin. Rheumatol.* **2008**, *14*, 65–68.
581. Faller, H.; Lippelt, A.; Nagele, S.; Klein, C.E. Emotional wellbeing, physical pain and doctor-patient relationship in patients hypersensitive to local anesthesia. Signs of somatization? A controlled cross-sectional study. *ZEITSCHRIFT FUR Klin. Psychol. Psychiatr. UND Psychother.* **1999**, *47*, 316–332.
582. Fang, H.; Xie, Q.; Boneva, R.; Fostel, J.; Perkins, R.; Tong, W. Gene expression profile exploration of a large dataset on chronic fatigue syndrome. *Pharmacogenomics* **2006**, *7*, 429–440.
583. Farmer, A.; Fowler, T.; Scourfield, J.; Thapar, A. Prevalence of chronic disabling fatigue in children and adolescents. *Br. J. Psychiatry* **2004**, *184*, 477–481.
584. Farney, R.J.; Lugo, A.; Jensen, R.L.; Walker, J.M.; Cloward, T. V Simultaneous use of antidepressant and antihypertensive medications increases likelihood of diagnosis of obstructive sleep apnea syndrome. *Chest* **2004**, *125*, 1279–1285.
585. Faro, M.; Sáez-Francàs, N.; Castro-Marrero, J.; Aliste, L.; Collado, A.; Alegre, J. Impact of the fibromyalgia in the chronic fatigue syndrome [Impacto de la

fibromialgia en el síndrome de fatiga crónica]. *Med. Clin. (Barc).* **2014**, *142*, 519–525. Work in Spanish
586. Faro, M.; Saez-Francas, N.; Castro-Marrero, J.; Aliste, L.; Collado, A.; Alegre, J. [Impact of the fibromyalgia in the chronic fatigue syndrome]. *Med. Clin. (Barc).* **2014**, *142*, 519–525. Work in Spanish
587. Faro, M.; Saez-Francas, N.; Castro-Marrero, J.; Aliste, L.; Fernandez de Sevilla, T.; Alegre, J. Gender differences in chronic fatigue syndrome. *Reumatol. Clin.* **2016**, *12*, 72–77.
588. Farrar, D.J.; Locke, S.E.; Kantrowitz, F.G. Chronic fatigue syndrome. 1: Etiology and pathogenesis. *Behav. Med.* **1995**, *21*, 5–16.
589. Fasano, A. Celiac disease - How to handle a clinical chameleon. *N. Engl. J. Med.* **2003**, *348*, 2568–2570.
590. Fatt, S.J.; Cvejic, E.; Lloyd, A.R.; Vollmer-Conna, U.; Beilharz, J.E. The Invisible Burden of Chronic Fatigue in the Community: a Narrative Review. *Curr. Rheumatol. Rep.* **2019**, *21*.
591. Faulkner, S.; Smith, A. A longitudinal study of the relationship between psychological distress and recurrence of upper respiratory tract infections in chronic fatigue syndrome. *Br. J. Health Psychol.* **2008**, *13*, 177–186.
592. Fava, M. Prevalence and burden in the medically ill. *Prim. Care Companion J. Clin. Psychiatry* **2006**, *7*, 282–285.
593. Fawkes-Kirby, T.M.; Wheeler, M.A.; Anton, H.A.; Miller, W.C.; Townson, A.F.; Weeks, C.A.O. Clinical correlates of fatigue in spinal cord injury. *Spinal Cord* **2008**, *46*, 21–25.
594. Fedorowski, A. Postural orthostatic tachycardia syndrome: clinical presentation, aetiology and management. *J. Intern. Med.* **2019**, *285*, 352–366.
595. Feiring, B.; Laake, I.; Trogstad, L. No conflicting results in the article "HPV vaccination and risk of chronic fatigue syndrome/myalgic encephalomyelitis: A nationwide register-based study from Norway." *Vaccine* **2017**, *35*, 7082–7083.
596. Feiring, B.; Laake, I.; Bakken, I.J.; Greve-Isdahl, M.; Wyller, V.B.; Haberg, S.E.; Magnus, P.; Trogstad, L. HPV vaccination and risk of chronic fatigue syndrome/myalgic encephalomyelitis: A nationwide register-based study from Norway. *Vaccine* **2017**, *35*, 4203–4212.
597. Felger, J.C.; Cole, S.W.; Pace, T.W.W.; Hu, F.; Woolwine, B.J.; Doho, G.H.; Raison, C.L.; Miller, A.H. Molecular signatures of peripheral blood mononuclear cells during chronic interferon-α treatment: Relationship with depression and fatigue. *Psychol. Med.* **2012**, *42*, 1591–1603.
598. Fenske, M. Comment on "Diurnal excretion of urinary cortisol, cortisone, and cortisol metabolites in chronic fatigue syndrome." *J. Psychosom. Res.* **2006**, *60*, 627–628.
599. Ferguson, E.; Rabbitt Roff, S.; Unwin, C.; David, A.; Everitt, B.; Ismail, K.; Wessely, S.; Hunt, S.C.; Richardson, R.D.; McFall, M.; et al. Is there a Gulf War syndrome? (multiple letters) [1]. *Lancet* **1999**, *353*, 1182–1183.
600. Ferrada-Noli, M. [Occupational stress, suicide and fatigue depression]. *Lakartidningen* **2001**, *98*, 3158–3160. Work in Swedish
601. Ferre Ybarz, L.; Cardona Dahl, V.; Cadahia Garcia, A.; Ruiz, E.; Vazquez, A.; Fernandez de Sevilla, T.; Alegre Martin, J. [Prevalence of atopy in chronic fatigue syndrome]. *Allergol. Immunopathol. (Madr).* **2005**, *33*, 42–47. Work in Spanish
602. Ferré Ybarz, L.; Cardona Dahl, V.; Cadahía García, A.; Ruiz, E.; Vázquez, A.; Fernández De Sevilla, T.; Alegre Martín, J. Prevalence of atopy in chronic fatigue

syndrome [Prevalencia de atopia en el síndrome de fatiga crónica]. *Allergol. Immunopathol. (Madr).* **2005**, *33*, 42–47. Work in Spanish
603. Ferre, A. Chronic fatigue syndrome and sleep disorders: clinical associations and diagnostic difficulties. *NEUROLOGIA* **2018**, *33*, 385–394.
604. Ferré, A. Chronic fatigue syndrome and sleep disorders: clinical associations and diagnostic difficulties [Síndrome de fatiga crónica y los trastornos del sueño: relaciones clínicas y dificultades diagnósticas]. *Neurologia* **2018**, *33*, 385–394. Work in Spanish
605. Fiedler, N.; Kipen, H.M. Controlled exposures to volatile organic compounds in sensitive groups. *Ann. N. Y. Acad. Sci.* **2001**, *933*, 24–37.
606. Fiedler, N.; Kipen, H.M.; DeLuca, J.; Kelly-McNeil, K.; Natelson, B. A controlled comparison of multiple chemical sensitivities and chronic fatigue syndrome. *Psychosom. Med.* **1996**, *58*, 38–49.
607. Fiedler, N.; Kipen, H.; Natelson, B.; Ottenweller, J. Chemical sensitivities and the Gulf War: Department of Veterans Affairs Research Center in basic and clinical science studies of environmental hazards. *Regul. Toxicol. Pharmacol.* **1996**, *24*, S129-38.
608. Fiedler, N.; Lange, G.; Tiersky, L.; DeLuca, J.; Policastro, T.; Kelly-McNeil, K.; McWilliams, R.; Korn, L.; Natelson, B. Stressors, personality traits, and coping of Gulf War veterans with chronic fatigue. *J. Psychosom. Res.* **2000**, *48*, 525–535.
609. Field, T.; Hernandez-Reif, M.; Diego, M.; Schanberg, S.; Kuhn, C. Cortisol decreases and serotonin and dopamine increase following massage therapy. *Int. J. Neurosci.* **2005**, *115*, 1397–1413.
610. Fiest, K.M.; Currie, S.R.; Williams, J.V.A.; Wang, J. Chronic conditions and major depression in community-dwelling older adults. *J. Affect. Disord.* **2011**, *131*, 172–178.
611. Findlay, S.M. The tired teen: A review of the assessment and management of the adolescent with sleepiness and fatigue. *Paediatr. Child Health* **2008**, *13*, 37–42.
612. Fink, P.; Schroder, A. One single diagnosis, bodily distress syndrome, succeeded to capture 10 diagnostic categories of functional somatic syndromes and somatoform disorders. *J. Psychosom. Res.* **2010**, *68*, 415–426.
613. Firoz, E.F.; Kamino, H.; Lehman, T.J.A.; Orlow, S.J. Morphea, diabetes mellitus type I, and celiac disease: Case report and review of the literature. *Pediatr. Dermatol.* **2010**, *27*, 48–52.
614. Fischer, S.; Gaab, J.; Ehlert, U.; Nater, U.M. Prevalence, Overlap, and Predictors of Functional Somatic Syndromes in a Student Sample. *Int. J. Behav. Med.* **2013**, *20*, 184–193.
615. Fischer, S.; Lemmer, G.; Gollwitzer, M.; Nater, U.M. Stress and Resilience in Functional Somatic Syndromes - A Structural Equation Modeling Approach. *PLoS One* **2014**, *9*.
616. Fischer, S.; Nater, U.M. Functional somatic syndromes: asking about exclusionary medical conditions results in decreased prevalence and overlap rates. *BMC Public Health* **2014**, *14*, 1034.
617. Fischler, B.; Cluydts, R.; De Gucht, Y.; Kaufman, L.; De Meirleir, K. Generalized anxiety disorder in chronic fatigue syndrome. *Acta Psychiatr. Scand.* **1997**, *95*, 405–413.
618. Fisher, L. Chronic fatigue syndrome. *Prof. Nurse* **1997**, *12*, 578–581.
619. Fisher, M.M.; Doig, G.S. Prevention of anaphylactic reactions to anaesthetic drugs. *Drug Saf.* **2004**, *27*, 393–410.

620. Fisher, M.M.; Rose, M. Anaesthesia for patients with idiopathic environmental intolerance and chronic fatigue syndrome. *Br. J. Anaesth.* **2008**, *101*, 486–491.
621. Fitzgibbon, E.J.; Murphy, D.; O'Shea, K.; Kelleher, C. Chronic debilitating fatigue in Irish general practice: a survey of general practitioners' experience. *Br. J. Gen. Pract.* **1997**, *47*, 618–622.
622. Fjällskog, M.-L.; Janson, E.T. Treatment of endocrine pancreatic tumors. *Acta Oncol. (Madr).* **2005**, *44*, 329–338.
623. Fletcher, C.; Bradnam, L.; Barr, C. The relationship between knowledge of pain neurophysiology and fear avoidance in people with chronic pain: A point in time, observational study. *Physiother. Theory Pract.* **2016**, *32*, 271–276.
624. Flo, E.; Chalder, T. Prevalence and predictors of recovery from chronic fatigue syndrome in a routine clinical practice. *Behav. Res. Ther.* **2014**, *63*, 1–8.
625. Florenzano, R. Neurasthenia and chronic fatigue syndrome: The rise, fall and revival of a disease entity [La neurastenia y el sindrome de fatiga cronica: Auge, caida y renacimiento de un concepto morbido]. *Rev. Chil. Neuropsiquiatr.* **1997**, *35*, 175–185. Work in Spanish
626. Flores, S.; Brown, A.; Adeoye, S.; Jason, L.A.; Evans, M. Examining the impact of obesity on individuals with chronic fatigue syndrome. *Workplace Health Saf.* **2013**, *61*, 299–307.
627. Fluge, Ø.; Risa, K.; Lunde, S.; Alme, K.; Rekeland, I.G.; Sapkota, D.; Kristoffersen, E.K.; Sørland, K.; Bruland, O.; Dahl, O.; et al. B-lymphocyte depletion in myalgic encephalopathy/chronic fatigue syndrome. An open-label phase II study with rituximab maintenance treatment. *PLoS One* **2015**, *10*.
628. Flynn, J.T.; Urbina, E.M. Pediatric Ambulatory Blood Pressure Monitoring: Indications and Interpretations. *J. Clin. Hypertens.* **2012**, *14*, 372–382.
629. Fohlman, J.; Friman, G. Is juvenile diabetes a viral disease? *Ann. Med.* **1993**, *25*, 569–574.
630. Fontana, J.; Wenz, R.; Groden, C.; Schmieder, K.; Wenz, H. The Preinterventional Psychiatric History as a Major Predictor for a Reduced Quality of Life After Treatment of Unruptured Intracranial Aneurysms. *World Neurosurg.* **2015**, *84*, 1215–1222.
631. Fontenele, J.B.; Félix, F.H.C. Fibromyalgia and related medically unexplained symptoms: A lost link between cardiovascular and nociception modulation. *J. Musculoskelet. Pain* **2009**, *17*, 67–79.
632. Forsyth, L.M.; Preuss, H.G.; MacDowell, A.L.; Chiazze, L.; Birkmayer, G.D.; Bellanti, J.A. Therapeutic effects of oral NADH on the symptoms of patients with chronic fatigue syndrome. *Ann. ALLERGY ASTHMA Immunol.* **1999**, *82*, 185–191.
633. Fossey, M.; Libman, E.; Bailes, S.; Baltzan, M.; Schondorf, R.; Amsel, R.; Fichten, C.S. Sleep quality and psychological adjustment in chronic fatigue syndrome. *J. Behav. Med.* **2004**, *27*, 581–605.
634. Fostel, J.; Boneva, R.; Lloyd, A. Exploration of the gene expression correlates of chronic unexplained fatigue using factor analysis. *Pharmacogenomics* **2006**, *7*, 441–454.
635. Fourquet, J.; Sinaii, N.; Stratton, P.; Khayel, F.; Alvarez-Garriga, C.; Bayona, M.; Ballweg, M.L.; Flores, I. Characteristics of women with endometriosis from the USA and Puerto Rico. *J. Endometr.* **2015**, *7*, 129–135.
636. Franc, M.; Michalski, B.; Kuczerawy, I.; Szuta, J.; Skrzypulec-Plinta, V. Cancer related fatigue syndrome in neoplastic diseases. *Prz. Menopauzalny* **2014**, *13*, 352–355.
637. Francis, C. Take ME seriously. *Nurs. Stand.* **2001**, *15*, 22.

638. Francis, S.S.; Selvin, S.; Yang, W.; Buffler, P.A.; Wiemels, J.L. Unusual space-time patterning of the Fallon, Nevada leukemia cluster: Evidence of an infectious etiology. *Chem. Biol. Interact.* **2012**, *196*, 102–109.
639. Frank, E.; Dingle, A.D. Self-reported depression and suicide attempts among U.S. women physicians. *Am. J. Psychiatry* **1999**, *156*, 1887–1894.
640. Frean, J.; Blumberg, L. Tick bite fever and Q fever - A South African perspective. *South African Med. J.* **2007**, *97*, 1198–1202.
641. Freeman, R. Objective evidence of autonomic dysfunction and the role of stress in the gulf war syndrome. *JAMA Neurol.* **2013**, *70*, 158–159.
642. Freeman, R. *Chronic Fatigue and the Autonomic Nervous System*; 2004;
643. Freeman, R. The chronic fatigue syndrome is a disease of the autonomic nervous system. Sometimes. *Clin. Auton. Res.* **2002**, *12*, 231–233.
644. Friedberg, F. Chronic fatigue syndrome: A new clinical application. *Prof. Psychol. Pract.* **1996**, *27*, 487–494.
645. Friedberg, F.; Dechene, L.; McKenzie, M.J. 2nd; Fontanetta, R. Symptom patterns in long-duration chronic fatigue syndrome. *J. Psychosom. Res.* **2000**, *48*, 59–68.
646. Friedberg, F.; Leung, D.W.; Quick, J. Do support groups help people with chronic fatigue syndrome and fibromyalgia? A comparison of active and inactive members. *J. Rheumatol.* **2005**, *32*, 2416–2420.
647. Friedberg, F.; Quick, J. Alexithymia in chronic fatigue syndrome: associations with momentary, recall, and retrospective measures of somatic complaints and emotions. *Psychosom. Med.* **2007**, *69*, 54–60.
648. Friedberg, F.; Sohl, S.; Schmeizer, B. Publication trends in chronic fatigue syndrome: comparisons with fibromyalgia and fatigue: 1995-2004. *J. Psychosom. Res.* **2007**, *63*, 143–146.
649. Friedberg, F.; Tintle, N.; Clark, J.; Bromet, E. Prolonged fatigue in Ukraine and the United States: prevalence and risk factors. *FATIGUE-BIOMEDICINE Heal. Behav.* **2015**, *3*, 33–46.
650. Friganović, A.; Kovačević, I.; Ilić, B.; Žulec, M.; Krikšić, V.; Bile, C.G. Healthy settings in hospital - How to prevent burnout syndrome in nurses: Literature review. *Acta Clin. Croat.* **2017**, *56*, 292–298.
651. Frith, J.; Ng, W.-F.; Day, C.P.; Payne, B.; Sheerin, N.; Gorman, G.; Jones, D.; Newton, J.L. Orthostatic intolerance is common in chronic disease - A clinical cohort study. *Int. J. Cardiol.* **2014**, *174*, 861–863.
652. Fritschi, C.; Quinn, L. Fatigue in patients with diabetes: A review. *J. Psychosom. Res.* **2010**, *69*, 33–41.
653. Frost, J.; Okun, S.; Vaughan, T.; Heywood, J.; Wicks, P. Patient-reported outcomes as a source of evidence in off-label prescribing: Analysis of data from PatientsLikeMe. *J. Med. Internet Res.* **2011**, *13*.
654. Frostholm, L.; Ornbol, E.; Hansen, H.S.; Olesen, F.; Weinman, J.; Fink, P. Which is more important for outcome: the physician's or the patient's understanding of a health problem? A 2-year follow-up study in primary care. *Gen. Hosp. Psychiatry* **2010**, *32*, 1–8.
655. Fukuda, K.; Dobbins, J.G.; Wilson, L.J.; Dunn, R.A.; Wilcox, K.; Smallwood, D. An epidemiologic study of fatigue with relevance for the chronic fatigue syndrome. *J. Psychiatr. Res.* **1997**, *31*, 19–29. Proceedings of the 1st Research and Clinical Conference of the American-Association-for-Chronic-Fatigue-Syndrome in FT Lauderdale, USA.

656. FUKUDA, K.; STRAUS, S.E.; HICKIE, I.; SHARPE, M.C.; DOBBINS, J.G.; KOMAROFF, A.; SCHLUEDERBERG, A.; JONES, J.F.; LLOYD, A.R.; WESSELY, S.; et al. THE CHRONIC FATIGUE SYNDROME - A COMPREHENSIVE APPROACH TO ITS DEFINITION AND STUDY. *Ann. Intern. Med.* **1994**, *121*, 953–959.
657. Fukudo, S.; Kaneko, H.; Akiho, H.; Inamori, M.; Endo, Y.; Okumura, T.; Kanazawa, M.; Kamiya, T.; Sato, K.; Chiba, T.; et al. Evidence-based clinical practice guidelines for irritable bowel syndrome. *J. Gastroenterol.* **2014**, *50*, 11–30.
658. Fulle, S.; Belia, S.; Vecchiet, J.; Morabito, C.; Vecchiet, L.; Fano, G. Modification of the functional capacity of sarcoplasmic reticulum membranes in patients suffering from chronic fatigue syndrome. *Neuromuscul. Disord.* **2003**, *13*, 479–484.
659. Fuller-Thomson, E.; Nimigon-Young, J.; Brennenstuhl, S. Individuals with fibromyalgia and depression: Findings from a nationally representative Canadian survey. *Rheumatol. Int.* **2012**, *32*, 853–862.
660. Fuller-Thomson, E.; Nimigon, J. Factors associated with depression among individuals with chronic fatigue syndrome: findings from a nationally representative survey. *Fam. Pract.* **2008**, *25*, 414–422.
661. Furberg, H.; Olarte, M.; Afari, N.; Goldberg, J.; Buchwald, D.; Sullivan, P.F. The prevalence of self-reported chronic fatigue in a U.S. twin registry. *J. Psychosom. Res.* **2005**, *59*, 283–290.
662. Furczyk, K.; Thome, J. Adult ADHD and suicide. *ADHD Atten. Deficit Hyperact. Disord.* **2014**, *6*, 153–158.
663. Furst, G. Measuring fatigue in chronic fatigue syndrome: Why and how. *J. Chronic Fatigue Syndr.* **1999**, *5*, 55–59.
664. Furuta, R.A.; Miyazawa, T.; Sugiyama, T.; Kuratsune, H.; Ikeda, Y.; Sato, E.; Misawa, N.; Nakatomi, Y.; Sakuma, R.; Yasui, K.; et al. No association of xenotropic murine leukemia virus-related virus with prostate cancer or chronic fatigue syndrome in Japan. *Retrovirology* **2011**, *8*.
665. Gaab, J. Psychotherapy of chronic fatigue states [Psychotherapie chronischer erschöpfungszustände]. *Psychotherapeut* **2004**, *49*, 431–445. Work in German
666. Gaab, J. Search for the somatic cause of the chronic fatigue syndrome. *PSYCHOTHERAPEUT* **2011**, *56*, 211–215.
667. Gaab, J.; Engert, V.; Heitz, V.; Schad, T.; Schurmeyer, T.H.; Ehlert, U. Associations between neuroendocrine responses to the Insulin Tolerance Test and patient characteristics in chronic fatigue syndrome. *J. Psychosom. Res.* **2004**, *56*, 419–424.
668. Gaber, T.; Oo, W.W. Prevalence of hypothyroidism in chronic fatigue syndrome patients (CFS/ME). *J. Neurol.* **2013**, *260*, S98–S99.
669. Gaber, T.A.-Z.K.; Oo, W.W.; Ringrose, H. Multiple Sclerosis/Chronic Fatigue Syndrome overlap: When two common disorders collide. *NeuroRehabilitation* **2014**, *35*, 529–534.
670. Gadalla, T. Association of comorbid mood disorders and chronic illness with disability and quality of life in Ontario, Canada. *Chronic Dis. Can.* **2008**, *28*, 148–154.
671. Gadalla, T.M. Disability associated with comorbid anxiety disorders in women with chronic physical illness in Ontario, Canada. *Women Heal.* **2008**, *48*, 1–20.

672. Galbraith, D.N.; Nairn, C.; Clements, G.B. Phylogenetic analysis of short enteroviral sequences from patients with chronic fatigue syndrome. *J. Gen. Virol.* **1995**, *76 (Pt 7)*, 1701–1707.
673. Gallagher, A.M.; Thomas, J.M.; Hamilton, W.T.; White, P.D. Incidence of fatigue symptoms and diagnoses presenting in UK primary care from 1990 to 2001. *J. R. Soc. Med.* **2004**, *97*, 571–575.
674. Galvez-Sánchez, C.M.; Duschek, S.; Del Paso, G.A.R. Psychological impact of fibromyalgia: Current perspectives. *Psychol. Res. Behav. Manag.* **2019**, *12*, 117–127.
675. Galvez-Sanchez, C.M.; Duschek, S.; del Paso, G.A. Psychological impact of fibromyalgia: current perspectives. *Psychol. Res. Behav. Manag.* **2019**, *12*, 117–127.
676. Gamma, A.; Angst, J.; Ajdacic, V.; Eich, D.; Roessler, W. The spectra of neurasthenia and depression: course, stability and transitions. *Eur. Arch. Psychiatry Clin. Neurosci.* **2007**, *257*, 120–127.
677. Ganelin-Cohen, E.; Ashkenasi, A. Disordered sleep in pediatric patients with attention deficit hyperactivity disorder: An overview. *Isr. Med. Assoc. J.* **2013**, *15*, 705–709.
678. Gantz, N.M. 10 key questions answered on chronic fatigue syndrome. *Contemp. Intern. Med.* **1995**, *7*, 15-16,21-24,27-28.
679. Garcia-Alvarez, L.; Perez-Matute, P.; Blanco, J.R.; Ibarra, V.; Oteo, J.A.; García-Álvarez, L.; Pérez-Matute, P.; Blanco, J.R.; Ibarra, V.; Oteo, J.A. High prevalence of asymptomatic carriers of Tropheryma whipplei in different populations from the North of Spain. *Enferm. Infecc. Microbiol. Clin.* **2016**, *34*, 340–345.
680. Garg, R.K.; Malhotra, H.S.; Jain, A.; Malhotra, K.P. Dengue-associated neuromuscular complications. *Neurol. India* **2015**, *63*, 497–516.
681. Garralda, M.E.; Rangel, L. Annotation: Chronic fatigue syndrome in children and adolescents. *J. Child Psychol. Psychiatry Allied Discip.* **2002**, *43*, 169–176.
682. Garralda, M.E.; Rangel, L. Chronic fatigue syndrome of childhood. Comparative study with emotional disorders. *Eur. Child Adolesc. Psychiatry* **2005**, *14*, 424–430.
683. Garralda, M.E.; Rangel, L. Impairment and coping in children and adolescents with chronic fatigue syndrome: a comparative study with other paediatric disorders. *J. Child Psychol. Psychiatry.* **2004**, *45*, 543–552.
684. Gärtner, B.C.; Fischinger, J.M.; Roemer, K.; Mak, M.; Fleurent, B.; Mueller-Lantzsch, N.; Gartner, B.C.; Fischinger, J.M.; Roemer, K.; Mak, M.; et al. Evaluation of a recombinant line blot for diagnosis of Epstein-Barr Virus compared with ELISA, using immunofluorescence as reference method. *J. Virol. Methods* **2001**, *93*, 89–96.
685. Garus-Pakowska, A.; Leśniewska, A.; Gaszyńska, E.; Szatko, F. Occupational exposure and health problems among Polish denturists: a population-based study in Łódź province. *Int. Dent. J.* **2016**, *66*, 237–246.
686. Gasperi, M.; Krieger, J.N.; Forsberg, C.; Goldberg, J.; Buchwald, D.; Afari, N. Chronic prostatitis and comorbid non-urological overlapping pain conditions: A co-twin control study. *J. Psychosom. Res.* **2017**, *102*, 29–33.
687. Geinitz, H.; Zimmermann, F.B.; Thamm, R.; Keller, M.; Busch, R.; Molls, M. Fatigue in patients with adjuvant radiation therapy for breast cancer: Long-term follow-up. *J. Cancer Res. Clin. Oncol.* **2004**, *130*, 327–333.
688. Geisser, M.E.; Strader Donnell, C.; Petzke, F.; Gracely, R.H.; Clauw, D.J.; Williams, D.A. Comorbid somatic symptoms and functional status in patients with

fibromyalgia and chronic fatigue syndrome: sensory amplification as a common mechanism. *Psychosomatics* **2008**, *49*, 235–242.
689. Geist, R.; Weinstein, M.; Walker, L.; Campo, J. V Medically unexplained symptoms in young people: The doctor's dilemma. *Paediatr. Child Health* **2008**, *13*, 487–491.
690. Gendelman, O.; Amital, H. Is it tiring to deal with fatigue? *Isr. Med. Assoc. J.* **2012**, *14*, 566–567.
691. Genuis, S.J.; Lipp, C.T. Electromagnetic hypersensitivity: Fact or fiction? *Sci. Total Environ.* **2012**, *414*, 103–112.
692. Geoffroy, P.A.; Amad, A.; Gangloff, C.; Thomas, P. Fibromyalgia and psychiatry: 35 years later ... What's new? *Press. MEDICALE* **2012**, *41*, 455–465.
693. Geraghty, K.J.; Adeniji, C. The importance of accurate diagnosis of ME/CFS in children and adolescents: A commentary. *Front. Pediatr.* **2019**, *6*.
694. Gerstenblith, T.A.; Stern, T.A. Lyme Disease: A Review of Its Epidemiology, Evaluation, and Treatment. *Psychosomatics* **2014**, *55*, 421–429.
695. Gewin, V. Medical research: Subject to reflection. *Nature* **2015**, *521*, 551–553.
696. Ghosh, A.K.; Ghosh, K. The head-up tilt test for diagnosing chronic fatigue syndrome [2]. *QJM - Mon. J. Assoc. Physicians* **2003**, *96*, 379–380.
697. Gibbons, R.; Pheby, D.F.H.; Richards, C.; Bray, F.I. Severe CFS/ME of juvenile onset - A report from the CHROME database. *J. Chronic Fatigue Syndr.* **1998**, *4*, 67–80.
698. Gibson, P.R.; Vogel, V.M. Sickness-related dysfunction in persons with self-reported multiple chemical sensitivity at four levels of severity. *J. Clin. Nurs.* **2009**, *18*, 72–81.
699. Gielissen, M.F.; Knoop, H.; Servaes, P.; Kalkman, J.S.; Huibers, M.J.; Verhagen, S.; Bleijenberg, G. Differences in the experience of fatigue in patients and healthy controls: patients' descriptions. *Health Qual. Life Outcomes* **2007**, *5*, 36.
700. Gikas, A.; Kokkini, S.; Tsioutis, C. Q fever: Clinical manifestations and treatment. *Expert Rev. Anti. Infect. Ther.* **2010**, *8*, 529–539.
701. Gilhooly, P.E.; Ottenweller, J.E.; Lange, G.; Tiersky, L.; Natelson, B.H. Chronic fatigue and sexual dysfunction in female Gulf War veterans. *J. Sex Marital Ther.* **2001**, *27*, 483–487.
702. Gillespie, N.A.; Zhu, G.; Heath, A.C.; Hickie, I.B.; Martin, N.G. The genetic aetiology of somatic distress. *Psychol. Med.* **2000**, *30*, 1051–1061.
703. Gillespie, N.; Kirk, K.M.; Heath, A.C.; Martin, N.G.; Hickie, I. Somatic distress as a distinct psychological dimension. *Soc. Psychiatry Psychiatr. Epidemiol.* **1999**, *34*, 451–458.
704. Giloteaux, L.; Goodrich, J.K.; Walters, W.A.; Levine, S.M.; Ley, R.E.; Hanson, M.R. Reduced diversity and altered composition of the gut microbiome in individuals with myalgic encephalomyelitis/chronic fatigue syndrome. *Microbiome* **2016**, *4*.
705. Ginsburg, K.S.; Kundsin, R.B.; Walter, C.W.; Schur, P.H. Ureaplasma urealyticum and mycoplasma hominis in women with systemic lupus erythematosus. *Arthritis Rheum.* **1992**, *35*, 429–433.
706. Gladwell, P.W.; Pheby, D.; Rodriguez, T.; Poland, F. Use of an online survey to explore positive and negative outcomes of rehabilitation for people with CFS/ME. *Disabil. Rehabil.* **2014**, *36*, 387–394.
707. Glattacker, M.; Opitz, U.; Jaeckel, W.H. Illness representations in women with fibromyalgia. *Br. J. Health Psychol.* **2010**, *15*, 367–387.

708. Godás Sieso, T.; Gómez Gil, E.; Salamero Baró, M.; Fernandez-Huerta, J.M.; Fernandez-Solá, J. Relationship between chronic fatigue syndrome and type A behaviour [Relación entre el síndrome de fatiga crónica y el patrón de conducta tipo A]. *Med. Clin. (Barc).* **2009**, *133*, 539–541. Work in Spanish
709. Godas Sieso, T.; Gomez Gil, E.; Salamero Baro, M.; Fernandez-Huerta, J.M.; Fernandez-Sola, J. [Relationship between chronic fatigue syndrome and type A behaviour]. *Med. Clin. (Barc).* **2009**, *133*, 539–541. Work in Spanish
710. Godas Sieso, T.; Nogue Xarau, S.; Salamero Baro, M.; Fernandez Sola, J. [Psychopathologic status in patients with chronic fatigue syndrome, associated or not with multiple chemical sensitivity]. *Med. Clin. (Barc).* 2014, *143*, 467–468. Work in Spanish
711. Godfrey, E.; Cleare, A.; Coddington, A.; Roberts, A.; Weinman, J.; Chalder, T. Chronic fatigue syndrome in adolescents: do parental expectations of their child's intellectual ability match the child's ability? *J. Psychosom. Res.* **2009**, *67*, 165–168.
712. Godwin, M.; Delva, D.; Miller, K.; Molson, J.; Hobbs, N.; MacDonald, S.; MacLeod, C. Investigating fatigue of less than 6 months' duration: Guidelines for family physicians. *Can. Fam. Physician* **1999**, *45*, 373–379.
713. Goedendorp, M.M.; Knoop, H.; Schippers, G.M.; Bleijenberg, G. The lifestyle of patients with chronic fatigue syndrome and the effect on fatigue and functional impairments. *J. Hum. Nutr. Diet.* **2009**, *22*, 226–231.
714. Goertzel, B.N.; Pennachin, C.; de Souza Coelho, L.; Gurbaxani, B.; Maloney, E.M.; Jones, J.F. Combinations of single nucleotide polymorphisms in neuroendocrine effector and receptor genes predict chronic fatigue syndrome. *Pharmacogenomics* **2006**, *7*, 475–483.
715. Goertzel, B.N.; Pennachin, C.; de Souza Coelho, L.; Maloney, E.M.; Jones, J.F.; Gurbaxani, B. Allostatic load is associated with symptoms in chronic fatigue syndrome patients. *Pharmacogenomics* **2006**, *7*, 485–494.
716. Gołab-Janowska, M.; Kotlega, D.; Safranow, K.; Meller, A.; Budzianowska, A.; Honczarenko, K. Risk Factors of Fatigue in Idiopathic Parkinson's Disease in a Polish Population. *Parkinsons. Dis.* **2016**, *2016*.
717. Gold, A.R.; Dipalo, F.; Gold, M.S.; O'Hearn, D. The symptoms and signs of upper airway resistance syndrome: A link to the functional somatic syndromes. *Chest* **2003**, *123*, 87–95.
718. Goldenberg, D.L. Diagnosis and Differential Diagnosis of Fibromyalgia. *Am. J. Med.* **2009**, *122*, S14–S21.
719. Goldenberg, D.L. Fibromyalgia, chronic fatigue syndrome, and myofascial pain syndrome. *Curr. Opin. Rheumatol.* **1993**, *5*, 199–208.
720. Goldenberg, D.L.; Simms, R.W.; Geiger, A.; Komaroff, A.L. High frequency of fibromyalgia in patients with chronic fatigue seen in a primary care practice. *Arthritis Rheum.* **1990**, *33*, 381–387.
721. Gomborone, J.E.; Gorard, D.A.; Dewsnap, P.A.; Libby, G.W.; Farthing, M.J. Prevalence of irritable bowel syndrome in chronic fatigue. *J. R. Coll. Physicians Lond.* **1996**, *30*, 512–513.
722. Gonthier, A.; Favrat, B. Chronic fatigue syndrome [Syndrome de fatigue chronique]. *Rev. Med. Suisse* **2015**, *11*, 2236–2242. Work in French
723. Goodnick, P.J.; Sandoval, R. Psychotropic treatment of chronic fatigue syndrome and related disorders. *J. Clin. Psychiatry* **1993**, *54*, 13–20.

724. Goodwin, L.; White, P.D.; Hotopf, M.; Stansfeld, S.A.; Clark, C. Life course study of the etiology of self-reported irritable bowel syndrome in the 1958 British birth cohort. *Psychosom. Med.* **2013**, *75*, 202–210.
725. Goodwin, L.; White, P.D.; Hotopf, M.; Stansfeld, S.A.; Clark, C. Psychopathology and physical activity as predictors of chronic fatigue syndrome in the 1958 british birth cohort: a replication study of the 1946 and 1970 birth cohorts. *Ann. Epidemiol.* **2011**, *21*, 343–350.
726. Goodwin, S.D.; Sproat, T.T.; Russell, W.L. Management of Lyme disease. *Clin. Pharm.* **1990**, *9*, 192–205.
727. Gordon, N.F.; Gulanick, M.; Costa, F.; Fletcher, G.; Franklin, B.A.; Roth, E.J.; Shephard, T. Physical activity and exercise recommendations for stroke survivors: An American Heart Association scientific statement from the Council on Clinical Cardiology, Subcommittee on Exercise, Cardiac Rehabilitation, and Prevention; the Council on Cardiovascular Nursing; the Council on Nutrition, Physical Activity, and Metabolism; and the Stroke Council. *Stroke* **2004**, *35*, 1230–1240.
728. Gorman, D.; Monigatti, J.; Glass, B.; Gronwall, D.; Beasley, M. Assessment of pentachlorophenolexposed timber workers using a test-of-poisoning model. *Int. J. Occup. Environ. Health* **2001**, *7*, 189–194.
729. Gorman, G.S.; Elson, J.L.; Newman, J.; Payne, B.; McFarland, R.; Newton, J.L.; Turnbull, D.M. Perceived fatigue is highly prevalent and debilitating in patients with mitochondrial disease. *Neuromuscul. Disord.* **2015**, *25*, 563–566.
730. Gottfries, C.G.; Regland, B.; Zachrisson, O.; Walinder, J. [Sick listing--"disaster" or incompetence in statistics and diagnostics?]. *Lakartidningen* **2003**, *100*, 1912–1914. Work in Swedish
731. Gotts, Z.M.; Newton, J.L.; Ellis, J.G.; Deary, V. The experience of sleep in chronic fatigue syndrome: A qualitative interview study with patients. *Br. J. Health Psychol.* **2016**, *21*, 71–92.
732. Goudsmit, E.M. Chronic fatigue syndrome. Distinguish between syndromes... *BMJ* 1994, *308*, 1297–1298.
733. Govender, C.; Cassimjee, N.; Schoeman, J.; Meyer, H. Psychological characteristics of FMS patients. *Scand. J. Caring Sci.* **2009**, *23*, 76–83.
734. Graff, L.A.; Walker, J.R.; Russell, A.S.; Bissonnette, R.; Bernstein, C.N. Fatigue and quality of sleep in patients with immune-mediated inflammatory disease. *J. Rheumatol.* **2011**, *38*, 36–42.
735. Grafman, J.; Johnson R., J.; Scheffers, M. Cognitive and mood-state changes in patients with chronic fatigue syndrome. *Rev. Infect. Dis.* **1991**, *13*, S45–S52.
736. Graham, C.D.; Rose, M.R.; Grunfeld, E.A.; Kyle, S.D.; Weinman, J. A systematic review of quality of life in adults with muscle disease. *J. Neurol.* **2011**, *258*, 1581–1592.
737. Gräns, H.; Evengård, B.; Nilsson, P. Transcriptome analysis of peripheral blood mononuclear cells from patients with chronic fatigue syndrome. *J. Chronic Fatigue Syndr.* **2008**, *14*, 7–25.
738. Gräns, H.; Nilsson, M.; Dahlman-Wright, K.; Evengård, B. Reduced levels of oestrogen receptor β mRNA in Swedish patients with chronic fatigue syndrome. *J. Clin. Pathol.* **2007**, *60*, 195–198.
739. Grans, H.; Nilsson, M.; Dahlman-Wright, K.; Evengard, B. Reduced levels of oestrogen receptor beta mRNA in Swedish patients with chronic fatigue syndrome. *J. Clin. Pathol.* **2007**, *60*, 195–198.

740. Grassini, S.; Nordin, S. Comorbidity in Migraine with Functional Somatic Syndromes, Psychiatric Disorders and Inflammatory Diseases: A Matter of Central Sensitization? *Behav. Med.* **2017**, *43*, 91–99.
741. Gray, D.; Parker-Cohen, N.Y.; White, T.; Clark, S.T.; Seiner, S.H.; Achilles, J.; McMahon, W.M. A comparison of individual and family psychology of adolescents with chronic fatigue syndrome, rheumatoid arthritis, and mood disorders. *J. Dev. Behav. Pediatr.* **2001**, *22*, 234–242.
742. Gray, G.C.; Kaiser, K.S.; Hawksworth, A.W.; Hall, F.W.; Barrett-Connor, E. Increased postwar symptoms and psychological morbidity among U.S. Navy Gulf War veterans. *Am. J. Trop. Med. Hyg.* **1999**, *60*, 758–766.
743. Gray, G.C.; Reed, R.J.; Kaiser, K.S.; Smith, T.C.; Gastañaga, V.M.; Gastanaga, V.M. Self-reported symptoms and medical conditions among 11,868 Gulf War-era veterans: The Seabee health study. *Am. J. Epidemiol.* **2002**, *155*, 1033–1044.
744. Greco, A.; Tannock, C.; Brostoff, J.; Costa, D.C. Brain MR in chronic fatigue syndrome. *Am. J. Neuroradiol.* **1997**, *18*, 1265–1269.
745. Green, C.R.; Cowan, P.; Elk, R.; O'Neil, K.M.; Rasmussen, A.L. National Institutes of Health Pathways to Prevention Workshop: Advancing the Research on Myalgic Encephalomyelitis/Chronic Fatigue Syndrome. *Ann. Intern. Med.* **2015**, *162*, 860–865.
746. Greenbaum, H.; Weil, C.; Chodick, G.; Shalev, V.; Eisenberg, V.H. Evidence for an association between endometriosis, fibromyalgia, and autoimmune diseases. *Am. J. Reprod. Immunol.* **2019**, *81*.
747. Grinde, B. Is chronic fatigue syndrome caused by a rare brain infection of a common, normally benign virus? *Med. Hypotheses* **2008**, *71*, 270–274.
748. Groom, H.C.T.; Boucherit, V.C.; Makinson, K.; Randal, E.; Baptista, S.; Hagan, S.; Gow, J.W.; Mattes, F.M.; Breuer, J.; Kerr, J.R.; et al. Absence of xenotropic murine leukaemia virus-related virus in UK patients with chronic fatigue syndrome. *Retrovirology* **2010**, *7*, 10.
749. Groom, H.C.T.; Warren, A.Y.; Neal, D.E.; Bishop, K.N. No Evidence for Infection of UK Prostate Cancer Patients with XMRV, BK Virus, Trichomonas vaginalis or Human Papilloma Viruses. *PLoS One* **2012**, *7*.
750. Grossman, E.R.; Speight, N.; Franklin, A. Does myalgic encephalomyelitis exist? *Lancet (London, England)* 2001, *357*, 1889–1890.
751. Groven, N.; Fors, E.A.; Iversen, V.C.; White, L.R.; Reitan, S.K. Association between cytokines and psychiatric symptoms in chronic fatigue syndrome and healthy controls. *Nord. J. Psychiatry* **2018**, *72*, 556–560.
752. Grufferman, S. Issues and problems in the conduct of epidemiologic research on chronic fatigue syndrome. *Rev. Infect. Dis.* **1991**, *13 Suppl 1*, S60-7.
753. Guilleminault, C.; Poyares, D.; Rosa, A. da; Kirisoglu, C.; Almeida, T.; Lopes, M.C. Chronic fatigue, unrefreshing sleep and nocturnal polysomnography. *Sleep Med.* **2006**, *7*, 513–520.
754. Gulas, E.; Wysiadecki, G.; Strzelecki, D.; Gawlik-Kotelnicka, O.; Polguj, M. Can microbiology affect psychiatry? A link between gut microbiota and psychiatric disorders [Jak mikrobiologia może wpływać na psychiatrię? Powiązania między florą bakteryjną jelit a zaburzeniami psychicznymi]. *Psychiatr. Pol.* **2018**, *52*, 1023–1039. Work in two languages: English and Polish
755. GUNN, W.J. EPIDEMIOLOGY OF CHRONIC FATIGUE SYNDROME .1. INTRODUCTION. *Clin. Infect. Dis.* **1994**, *18*, S10. Proceedings of the Symposium on chronic fatigue syndrome in London, UK.

756. Gunn, W.J. Part 1: Epidemiology of chronic fatigue syndrome. *Clin. Infect. Dis.* **1994**, *18*, S10.
757. Gunn, W.J.; Connell, D.B.; Randall, B. Epidemiology of chronic fatigue syndrome: the Centers for Disease Control Study. *Ciba Found. Symp.* **1993**, *173*, 83–101.
758. Gupta, A. Unconscious amygdalar fear conditioning in a subset of chronic fatigue syndrome patients. *Med. Hypotheses* **2002**, *59*, 727–735.
759. Gurbaxani, B.M.; Jones, J.F.; Goertzel, B.N.; Maloney, E.M. Linear data mining the Wichita clinical matrix suggests sleep and allostatic load involvement in chronic fatigue syndrome. *Pharmacogenomics* **2006**, *7*, 455–465.
760. Guseo, A. [Changes in the concept of multiple sclerosis]. *Orv. Hetil.* **1998**, *139*, 2875–2881. Work in Hungarian
761. Guven, B.; Oner, T.; Tavli, V.; Yilmazer, M.M.; Demirpence, S.; Mese, T. Low iron storage in children with tilt positive neurally mediated syncope. *WORLD J. Pediatr.* **2013**, *9*, 146–151.
762. Hackett, K.L.; Lambson, R.L.; Strassheim, V.; Gotts, Z.; Deary, V.; Newton, J.L. A concept mapping study evaluating the UK's first NHS generic fatigue clinic. *Heal. Expect.* **2016**, *19*, 1138–1149.
763. Hadlandsmyth, K.; Vowles, K.E. Does depression mediate the relation between fatigue severity and disability in chronic fatigue syndrome sufferers? *J. Psychosom. Res.* **2009**, *66*, 31–35.
764. Hadler, N.M.; Ehrlich, G.E. Fibromyalgia and the conundrum of disability determination. *J. Occup. Environ. Med.* **2003**, *45*, 1030–1033.
765. Hadzi-Pavlovic, D.; Hickie, I.B.; Wilson, A.J.; Davenport, T.A.; Lloyd, A.R.; Wakefield, D. Screening for prolonged fatigue syndromes: Validation of the SOFA scale. *Soc. Psychiatry Psychiatr. Epidemiol.* **2000**, *35*, 471–479.
766. Haeuser, W.; Ablin, J.; Fitzcharles, M.-A.; Littlejohn, G.; Luciano, J. V; Usui, C.; Walitt, B. Fibromyalgia. *Nat. Rev. Dis. Prim.* **2015**, *1*.
767. HAGNELL, O.; GRASBECK, A.; OJESJO, L.; OTTERBECK, L. MENTAL TIREDNESS IN THE LUNDBY STUDY - INCIDENCE AND COURSE OVER 25 YEARS. *ACTA Psychiatr. Scand.* **1993**, *88*, 316–321.
768. Haines, L.C.; Saidi, G.; Cooke, R.W.I. Prevalence of severe fatigue in primary care. *Arch. Dis. Child.* **2005**, *90*, 367–368.
769. Hairon, N. NICE guidance on managing chronic fatigue syndrome/ME. *Nurs. Times* **2007**, *103*, 21–22.
770. Hakim, A.; Grahame, R. Joint hypermobility. *BEST Pract. Res. Clin. Rheumatol.* **2003**, *17*, 989–1004.
771. Hakimi, R. [Comment on W. Hausotter: Modern illness from the critical viewpoint]. *Versicherungsmedizin* 2002, *54*, 149–50; author reply 150. Work in German
772. Hall, B.J.; Chang, K.; Chen, W.; Sou, K.L.; Latkin, C.; Yeung, A. Exploring the association between depression and shenjing shuairuo in a population representative epidemiological study of Chinese adults in Guangzhou, China. *Transcult. Psychiatry* **2018**, *55*, 733–753.
773. Hall, D.L.; Lattie, E.G.; Antoni, M.H.; Fletcher, M.A.; Czaja, S.; Perdomo, D.; Klimas, N.G. Stress management skills, cortisol awakening response, and post-exertional malaise in Chronic Fatigue Syndrome. *Psychoneuroendocrinology* **2014**, *49*, 26–31.
774. Halliez, M.C.M.; Buret, A.G. Extra-intestinal and long term consequences of Giardia duodenalis infections. *World J. Gastroenterol.* **2013**, *19*, 8974–8985.

775. Halpin, P.; Williams, M. V; Klimas, N.G.; Fletcher, M.A.; Barnes, Z.; Ariza, M.E. Myalgic encephalomyelitis/chronic fatigue syndrome and gulf war illness patients exhibit increased humoral responses to the herpesviruses-encoded dUTPase: Implications in disease pathophysiology. *J. Med. Virol.* **2017**, *89*, 1636–1645.
776. Hamaguchi, M.; Kawahito, Y.; Takeda, N.; Kato, T.; Kojima, T. Characteristics of chronic fatigue syndrome in a Japanese community population : chronic fatigue syndrome in Japan. *Clin. Rheumatol.* **2011**, *30*, 895–906.
777. Hambrook, D.; Oldershaw, A.; Rimes, K.; Schmidt, U.; Tchanturia, K.; Treasure, J.; Richards, S.; Chalder, T. Emotional expression, self-silencing, and distress tolerance in anorexia nervosa and chronic fatigue syndrome. *Br. J. Clin. Psychol.* **2011**, *50*, 310–325.
778. Hamilton, W.T.; Gallagher, A.M.; Thomas, J.M.; White, P.D. Risk markers for both chronic fatigue and irritable bowel syndromes: a prospective case-control study in primary care. *Psychol. Med.* **2009**, *39*, 1913–1921.
779. Hamilton, W.T.; Hall, G.H.; Round, A.P. Frequency of attendance in general practice and symptoms before development of chronic fatigue syndrome: a case-control study. *Br. J. Gen. Pract.* **2001**, *51*, 553–558.
780. Hamlen, R. Lyme borreliosis: perspective of a scientist-patient. *Lancet. Infect. Dis.* **2004**, *4*, 603–604.
781. Hanevik, K.; Wensaas, K.-A.; Rortveit, G.; Eide, G.E.; Morch, K.; Langeland, N. Irritable bowel syndrome and chronic fatigue 6 years after giardia infection: a controlled prospective cohort study. *Clin. Infect. Dis.* **2014**, *59*, 1394–1400.
782. Hanlon, P.; Nicholl, B.I.; Jani, B.D.; Lee, D.; McQueenie, R.; Mair, F.S. Frailty and pre-frailty in middle-aged and older adults and its association with multimorbidity and mortality: a prospective analysis of 493 737 UK Biobank participants. *Lancet Public Heal.* **2018**, *3*, e323–e332.
783. Hanno, P.; Nordling, J.; Fall, M. Bladder pain syndrome. *Med. Clin. North Am.* **2011**, *95*, 55–73.
784. Hansen, A.H.; Lian, O.S. Experiences of general practitioner continuity among women with chronic fatigue syndrome/myalgic encephalomyelitis: a cross-sectional study. *BMC Health Serv. Res.* **2016**, *16*, 650.
785. Hansen, A.H.; Lian, O.S. How do women with chronic fatigue syndrome/myalgic encephalomyelitis rate quality and coordination of healthcare services? A cross-sectional study. *BMJ Open* **2016**, *6*, e010277.
786. Hara, K.W.; Bjorngaard, J.H.; Jacobsen, H.B.; Borchgrevink, P.C.; Johnsen, R.; Stiles, T.C.; Brage, S.; Woodhouse, A. Biopsychosocial predictors and trajectories of work participation after transdiagnostic occupational rehabilitation of participants with mental and somatic disorders: a cohort study. *BMC Public Health* **2018**, *18*, 1014.
787. Harbeck, B.; Suefke, S.; Harten, P.; Haas, C.S.; Lehnert, H.; Moenig, H. High prevalence of fibromyalgia-associated symptoms in patients with hypothalamic-pituitary disorders. *Clin. Exp. Rheumatol.* **2013**, *31*, S16–S21.
788. Hardt, J.; Buchwald, D.; Wilks, D.; Sharpe, M.; Nix, W.A.; Egle, U.T. Health-related quality of life in patients with chronic fatigue syndrome: an international study. *J. Psychosom. Res.* **2001**, *51*, 431–434.
789. Harlow, B.L.; Signorello, L.B.; Hall, J.E.; Dailey, C.; Komaroff, A.L. Reproductive correlates of chronic fatigue syndrome. *Am. J. Med.* **1998**, *105*, 94S–99S.
790. Harrison, H.H.; Berg, D.E.; Berg, L.H.; Brewer, J. Increased prevalence of thrombophilia and hypofibrinolysis abnormalities in patients with Chronic Fatigue

Syndrome: Identification of probable predisposition factors. *Am. J. Hum. Genet.* **2001**, *69*, 564.
791. Hartman, J.M.; Berger, A.; Baker, K.; Bolle, J.; Handel, D.; Mannes, A.; Pereira, D.; St Germain, D.; Ronsaville, D.; Sonbolian, N.; et al. Quality of life and pain in premenopausal women with major depressive disorder: The POWER Study. *Health Qual. Life Outcomes* **2006**, *4*.
792. Hartz, A.J.; Bentler, S.; Noyes, R.; Hoehns, J.; Logemann, C.; Sinift, S.; Butani, Y.; Wang, W.; Brake, K.; Ernst, M.; et al. Randomized controlled trial of Siberian ginseng for chronic fatigue. *Psychol. Med.* **2004**, *34*, 51–61.
793. Hartz, A.J.; Kuhn, E.M.; Bentler, S.E.; Levine, P.H.; London, R. Prognostic factors for persons with idiopathic chronic fatigue. *Arch. Fam. Med.* **1999**, *8*, 495–501.
794. Hartz, A.J.; Kuhn, E.M.; Levine, P.H. Characteristics of fatigued persons associated with features of chronic fatigue syndrome. *J. Chronic Fatigue Syndr.* **1998**, *4*, 71–97.
795. Harvey, S.B.; Wadsworth, M.; Wessely, S.; Hotopf, M. The relationship between prior psychiatric disorder and chronic fatigue: evidence from a national birth cohort study. *Psychol. Med.* **2008**, *38*, 933–940.
796. Harvey, S.B.; Wadsworth, M.; Wessely, S.; Hotopf, M. Etiology of chronic fatigue syndrome: testing popular hypotheses using a national birth cohort study. *Psychosom. Med.* **2008**, *70*, 488–495.
797. Harvey, S.B.; Wessely, S. How Should Functional Somatic Syndromes Be Diagnosed? *Int. J. Behav. Med.* **2013**, *20*, 239–241.
798. Harvey, W.T.; Salvato, P. `Lyme disease': ancient engine of an unrecognized borreliosis pandemic? *Med. Hypotheses* **2003**, *60*, 742–759.
799. Hashimoto, N. History of chronic fatigue syndrome. *Nippon rinsho. Japanese J. Clin. Med.* **2007**, *65*, 975–982.
800. Hashimoto, N. [Chronic fatigue syndrome]. *Nihon Rinsho.* **1993**, *51 Suppl*, 1107–1114. <u>Work in Japanese</u>
801. Hasson, D.; Theorell, T.; Bergquist, J.; Canlon, B. Acute Stress Induces Hyperacusis in Women with High Levels of Emotional Exhaustion. *PLoS One* **2013**, *8*.
802. Hatchette, T.F.; Hayes, M.; Merry, H.; Schlech, W.F.; Marrie, T.J. The effect of C. burnetii infection on the quality of life of patients following an outbreak of Q fever. *Epidemiol. Infect.* **2003**, *130*, 491–495.
803. Hauge, C.R.; Rasmussen, A.; Piet, J.; Bonde, J.P.; Jensen, C.; Sumbundu, A.; Skovbjerg, S. Mindfulness-based cognitive therapy (MBCT) for multiple chemical sensitivity (MCS): Results from a randomized controlled trial with 1 year follow-up. *J. Psychosom. Res.* **2015**, *79*, 628–634.
804. Haukenes, G.; Aarli, J.A. [Postviral fatigue syndrome]. *Tidsskr. Nor. Laegeforen.* **1995**, *115*, 3017–3022. <u>Work in Norwegian</u>
805. Hauser, W.; Wilhelm, R.; Klein, W.; Zimmer, C. Causal illness attributions and healthcare utilization in fibromyalgia syndrome. *SCHMERZ* **2006**, *20*, 119+.
806. Hausteiner-Wiehle, C.; Henningsen, P. Irritable bowel syndrome: Relations with functional, mental, and somatoform disorders. *World J. Gastroenterol.* **2014**, *20*, 6024–6030.
807. Hausteiner, C.; Bornschein, S.; Hansen, J.; Zilker, T.; Forstl, H. Self-reported chemical sensitivity in Germany: A population-based survey. *Int. J. Hyg. Environ. Health* **2005**, *208*, 271–278.

808. Hausteiner, C.; Mergeay, A.; Bornschein, S.; Zilker, T.; Forstl, H. New aspects of psychiatric morbidity in idiopathic environmental intolerances. *J. Occup. Environ. Med.* **2006**, *48*, 76–82.
809. Havdahl, A.; Mitchell, R.; Paternoster, L.; Smith, G.D. Investigating causality in the association between vitamin D status and self-reported tiredness. *Sci. Rep.* **2019**, *9*.
810. Hawkes, D.; Benhamu, J.; Sidwell, T.; Miles, R.; Dunlop, R.A. Revisiting adverse reactions to vaccines: A critical appraisal of Autoimmune Syndrome Induced by Adjuvants (ASIA). *J. Autoimmun.* **2015**, *59*, 77–84.
811. Hayaki, C.; Anno, K.; Shibata, M.; Iwaki, R.; Kawata, H.; Sudo, N.; Hosoi, M. Family dysfunction A comparison of chronic widespread pain and chronic localized pain. *Medicine (Baltimore).* **2016**, *95*.
812. Haywood, K.L.; Collin, S.M.; Crawley, E. Assessing severity of illness and outcomes of treatment in children with Chronic Fatigue Syndrome/Myalgic Encephalomyelitis (CFS/ME): a systematic review of patient-reported outcome measures (PROMs). *Child. Care. Health Dev.* **2014**, *40*, 806–824.
813. Hazendonk, K.M.; Crowe, S.F. A neuropsychological study of the postpolio syndrome: support for depression without neuropsychological impairment. *Neuropsychiatry. Neuropsychol. Behav. Neurol.* **2000**, *13*, 112–118.
814. Heim, C.; Bierl, C.; Nisenbaum, R.; Wagner, D.; Reeves, W.C. Regional prevalence of fatiguing illnesses in the United States before and after the terrorist attacks of September 11, 2001. *Psychosom. Med.* **2004**, *66*, 672–678.
815. Heim, C.; Nater, U.M.; Maloney, E.; Boneva, R.; Jones, J.F.; Reeves, W.C. Childhood Trauma and Risk for Chronic Fatigue Syndrome Association With Neuroendocrine Dysfunction. *Arch. Gen. Psychiatry* **2009**, *66*, 72–80.
816. Heim, C.; Wagner, D.; Maloney, E.; Papanicolaou, D.A.; Solomon, L.; Jones, J.F.; Unger, E.R.; Reeves, W.C. Early adverse experience and risk for chronic fatigue syndrome: results from a population-based study. *Arch. Gen. Psychiatry* **2006**, *63*, 1258–1266.
817. Helfenstein Junior, M.; Goldenfum, M.A.; Favaro Siena, C.A. Fibromyalgia: clinical and occupational aspects. *Rev. Assoc. Med. Bras.* **2012**, *58*, 358–365.
818. Helg, C. Incapacitating late effects after chemo-and radiotherapy [Séquelles incapacitantes après chimio-et radiothérapie]. *Douleur Analg.* **2011**, *24*, S29–S33. Work in French
819. Heller, U.; Becker, E.W.; Zenner, H.P.; Berg, P.A. Incidence and clinical relevance of antibodies against phospholipids, serotonin and gangliosides in patients with sudden deafness and progressive hearing loss. *HNO* **1998**, *46*, 583–586.
820. Heller, U.; Becker, E.W.; Zenner, H.P.; Berg, P.A. Incidence and clinical relevance of antibodies against phospholipids, serotonin and gangliosides in patients with sudden deafness and progressive hearing loss [Haufigkeit und klinische relevanz von antikorpern gegen phospholipide, serotonin und ganglioside bei patienten mit horsturz und progredienter innenohrschwerhorigkeit]. *HNO* **1998**, *46*, 583–586. Work in German
821. Hempel, S.; Chambers, D.; Bagnall, A.-M.; Forbes, C. Risk factors for chronic fatigue syndrome/myalgic encephalomyelitis: a systematic scoping review of multiple predictor studies. *Psychol. Med.* **2008**, *38*, 915–926.
822. Henderson, D.A. Reflections on epidemic neuromyasthenia (chronic fatigue syndrome). *Clin. Infect. Dis.* **1994**, *18 Suppl 1*, S3-6; discussion S7-9.
823. Henderson, M.; Tannock, C. Objective assessment of personality disorder in chronic fatigue syndrome. *J. Psychosom. Res.* **2004**, *56*, 251–254.

824. Henderson, M.; Tannock, C. Use of depression rating scales in chronic fatigue syndrome. *J. Psychosom. Res.* **2005**, *59*, 181–184.
825. Henningsen, P.; Zimmermann, T.; Sattel, H. Medically unexplained physical symptoms, anxiety, and depression: a meta-analytic review. *Psychosom. Med.* **2003**, *65*, 528–533.
826. Henrich, T.J.; Li, J.Z.; Felsenstein, D.; Kotton, C.N.; Plenge, R.M.; Pereyra, F.; Marty, F.M.; Lin, N.H.; Grazioso, P.; Crochiere, D.M.; et al. Xenotropic murine leukemia virus-related virus prevalence in patients with chronic fatigue syndrome or chronic immunomodulatory conditions. *J. Infect. Dis.* **2010**, *202*, 1478–1481.
827. Herr, J.R. Is sleep disorder treatment appropriate for premenstrual syndrome? *Acta Obstet. Gynecol. Scand.* **2003**, *82*, 99.
828. Herrera, S.; de Vega, W.C.; Ashbrook, D.; Vernon, S.D.; McGowan, P.O. Genome-epigenome interactions associated with Myalgic Encephalomyelitis/Chronic Fatigue Syndrome. *EPIGENETICS* **2018**, *13*, 1174–1190.
829. Heyll, U.; Wachauf, P.; Senger, V.; Diewitz, M. Definitions of ``'Chronic Fatigue Syndrome'' (CFS). *Med. Klin.* **1997**, *92*, 221–227.
830. Hickie, I.B.; Davenport, T.A.; Hadzi-Pavlovic, D.; Koschera, A.; Naismith, S.L.; Scott, E.M.; Wilhelm, K.A. Development of a simple screening tool for common mental disorders in general practice. *Med. J. Aust.* **2001**, *175 Suppl*, S10-7.
831. Hickie, I.B.; Hooker, A.W.; Hadzi-Pavlovic, D.; Bennett, B.K.; Wilson, A.J.; Lloyd, A.R. Fatigue in selected primary care settings: Sociodemographic and psychiatric correlates. *Med. J. Aust.* **1996**, *164*, 585–588.
832. Hickie, I.B.; Lloyd, A.R.; Wakefield, D. Chronic fatigue syndrome: Current perspectives on evaluation and management. *Med. J. Aust.* **1995**, *163*, 314–315+317.
833. Hickie, I.B.; Scott, E.M.; Davenport, T.A. Somatic distress: Developing more integrated concepts. *Curr. Opin. Psychiatry* **1998**, *11*, 153–158.
834. Hickie, I.B.; Wilson, A.J.; Wright, J.M.; Bennett, B.K.; Wakefield, D.; Lloyd, A.R. A randomized, double-blind placebo-controlled trial of moclobemide in patients with chronic fatigue syndrome. *J. Clin. Psychiatry* **2000**, *61*, 643–648.
835. Hickie, I.; Davenport, T.; Issakidis, C.; Andrews, G. Neurasthenia: prevalence, disability and health care characteristics in the Australian community. *Br. J. PSYCHIATRY* **2002**, *181*, 56–61.
836. Hickie, I.; Hadzi-Pavlovic, D.; Ricci, C. Reviving the diagnosis of neurasthenia. *Psychol. Med.* 1997, *27*, 989–994.
837. Hickie, I.; Koschera, A.; Hadzi-Pavlovic, D.; Bennett, B.; Lloyd, A. The temporal stability and co-morbidity of prolonged fatigue: a longitudinal study in primary care. *Psychol. Med.* **1999**, *29*, 855–861.
838. Hickie, I.; Lloyd, A.; Wakefield, D. Chronic fatigue syndrome and depression. *Lancet (London, England)* 1991, *337*, 922–923.
839. Hickie, I.; Lloyd, A.; Wakefield, D.; Parker, G. The psychiatric status of patients with the chronic fatigue syndrome. *Br. J. Psychiatry* **1990**, *156*, 534–540.
840. Hickie, I.; Davenport, T.; Vernon, S.D.; Nisenbaum, R.; Reeves, W.C.; Hadzi-Pavlovic, D.; Lloyd, A. Are chronic fatigue and chronic fatigue syndrome valid clinical entities across countries and health-care settings? *Aust. N. Z. J. Psychiatry* **2009**, *43*, 25–35.
841. Hickie, I.; Davenport, T.; Wakefield, D.; Vollmer-Conna, U.; Cameron, B.; Vernon, S.D.; Reeves, W.C.; Lloyd, A. Post-infective and chronic fatigue

syndromes precipitated by viral and non-viral pathogens: prospective cohort study. *BMJ* **2006**, *333*, 575.
842. Higgins, J.N.P.; Pickard, J.D.; Lever, A.M.L. Chronic fatigue syndrome and idiopathic intracranial hypertension: Different manifestations of the same disorder of intracranial pressure? *Med. Hypotheses* **2017**, *105*, 6–9.
843. Hilgers, A.; Frank, J. Chronic fatigue syndrome: Evaluation of a 30-criteria-score and correlation with immune activation. *J. Chronic Fatigue Syndr.* **1996**, *2*, 35–47.
844. Hilgers, A.; Schoenherr, G. Chronic fatigue syndrome - Differential diagnosis at the laboratory of immunology [Das chronische mudigkeitssyndrom differentialdiagnostik im immunlabor]. *Arztezeitschrift fur Naturheilverfahren* **1999**, *40*, 208–212. Work in German
845. Hill, W.M. Are echoviruses still orphans? *Br. J. Biomed. Sci.* **1996**, *53*, 221–226.
846. Hiller, W.; Cuntz, U.; Rief, W.; Fichter, M.M. Searching for a gastrointestinal subgroup within the somatoform disorders. *Psychosomatics* **2001**, *42*, 14–20.
847. Hirschhorn, K.; Hirschhorn, R.; Hirschhorn, J.N. A Conversation with Kurt and Rochelle Hirschhorn. *Annu. Rev. Genomics Hum. Genet.* **2017**, *18*, 31–44.
848. Hjermstad, M.J.; Oldervoll, L.; Fosså, S.D.; Holte, H.; Jacobsen, A.B.; Loge, J.H. Quality of life in long-term Hodgkin's disease survivors with chronic fatigue. *Eur. J. Cancer* **2006**, *42*, 327–333.
849. Hjermstad, M.J.; Fossa, S.D.; Oldervoll, L.; Holte, H.; Jacobsen, A.B.; Loge, J.H. Fatigue in long-term Hodgkin's Disease survivors: a follow-up study. *J. Clin. Oncol.* **2005**, *23*, 6587–6595.
850. Ho-Yen, D.O.; McNamara, I. General practitioners' experience of the chronic fatigue syndrome. *Br. J. Gen. Pract.* **1991**, *41*, 324–326.
851. Ho, R.T.H.; Chan, J.S.M.; Wang, C.-W.; Lau, B.W.M.; So, K.F.; Yuen, L.P.; Sham, J.S.T.; Chan, C.L.W. A Randomized Controlled Trial of Qigong Exercise on Fatigue Symptoms, Functioning, and Telomerase Activity in Persons with Chronic Fatigue or Chronic Fatigue Syndrome. *Ann. Behav. Med.* **2012**, *44*, 160–170.
852. Hoad, A.; Spickett, G.; Elliott, J.; Newton, J. Postural orthostatic tachycardia syndrome is an under-recognized condition in chronic fatigue syndrome. *QJM* **2008**, *101*, 961–965.
853. Hobday, R.A.; Thomas, S.; O'Donovan, A.; Murphy, M.; Pinching, A.J. Dietary intervention in chronic fatigue syndrome. *J. Hum. Nutr. Diet.* **2008**, *21*, 141–149.
854. Hod, K.; Ringel-Kulka, T.; Martin, C.F.; Maharshak, N.; Ringel, Y. High-sensitive C-Reactive Protein as a Marker for Inflammation in Irritable Bowel Syndrome. *J. Clin. Gastroenterol.* **2016**, *50*, 227–232.
855. Hodgson, M.J.; Kipen, H.M. Gulf War illnesses: Causation and treatment. *J. Occup. Environ. Med.* **1999**, *41*, 443–452.
856. Hoey, M. Chronic fatigue syndrome. What is happening to M.E.? *Aust. Nurs. J.* **1994**, *2*, 18–20.
857. Hoffman, R.; Bibby, H.; Bennett, D.; Klineberg, E.; Rushworth, A.; Towns, S. Family functioning as a protective factor in treating adolescents with complex medico-psychosocial presentations. *Int. J. Adolesc. Med. Health* **2015**, *2015*.
858. Hohn, O.; Bannert, N. Origin of XMRV and its demise as a human pathogen associated with chronic fatigue syndrome. *Viruses* **2011**, *3*, 1312–1319.
859. Holecek, V.; Rokyta, R. Chronic fatifue syndrome [Chronický Únavový syndrome]. *Ceskoslov. Fysiol.* **2016**, *65*, 69–74. Work in Czech

860. Holecek, V.; Rokyta, R. [Chronic fatifue syndrome.]. *Ceskoslov. Fysiol.* **2016**, *65*, 69–74. Work in Czech
861. Holman, A.J. Positional cervical spinal cord compression and fibromyalgia: A novel comorbidity with important diagnostic and treatment implications. *J. PAIN* **2008**, *9*, 613–622.
862. Holtorf, K. Diagnosis and treatment of hypothalamic-pituitary-adrenal (HPA) axis dysfunction in patients with chronic fatigue syndrome (CFS) and fibromyalgia (FM). *J. Chronic Fatigue Syndr.* **2008**, *14*, 59–88.
863. Honda, M.; Kitamura, K.; Nakasone, T.; Fukushima, Y.; Matsuda, S.; Nishioka, K.; Matsuda, J.; Hashimoto, N.; Yamazaki, S. Japanese patients with chronic fatigue syndrome are negative for known retrovirus infections. *Microbiol. Immunol.* **1993**, *37*, 779–784.
864. Hood, E. Abdiction/addiction connection. *Environ. Health Perspect.* **2005**, *113*, A812–A814.
865. Hornig, M.; Briese, T.; Licinio, J.; Khabbaz, R.F.; Altshuler, L.L.; Potkin, S.G.; Schwemmle, M.; Siemetzki, U.; Mintz, J.; Honkavuori, K.; et al. Absence of evidence for bornavirus infection in schizophrenia, bipolar disorder and major depressive disorder. *Mol. Psychiatry* **2012**, *17*, 486–493.
866. Hosier, G.W.; Doiron, R.C.; Tolls, V.; Nickel, J.C. The X-Y factor: Females and males with urological chronic pelvic pain syndrome present distinct clinical phenotypes. *Can. Urol. Assoc. J.* **2018**, *12*, E270–E275.
867. Hossain, J.L.; Reinish, L.W.; Kayumov, L.; Bhuiya, P.; Shapiro, C.M. Underlying sleep pathology may cause chronic high fatigue in shift-workers. *J. Sleep Res.* **2003**, *12*, 223–230.
868. Hotopf, M.; Noah, N.; Wessely, S. Chronic fatigue and minor psychiatric morbidity after viral meningitis: A controlled study. *J. Neurol. Neurosurg. Psychiatry* **1996**, *60*, 504–509.
869. Hotopf, M.; Wessely, S. Can epidemiology clear the fog of war? Lessons from the 1990-91 Gulf War. *Int. J. Epidemiol.* **2005**, *34*, 791–800.
870. Houdenhove, B. V; Pae, C.-U.C.-U.; Luyten, P.; Van Houdenhove, B.; Pae, C.-U.C.-U.; Luyten, P. Chronic fatigue syndrome: Is there a role for non-antidepressant pharmacotherapy? *Expert Opin. Pharmacother.* **2010**, *11*, 215–223.
871. Hourfar, M.K.; Mayr-Wohlfart, U.; Themann, A.; Sireis, W.; Seifried, E.; Schrezenmeier, H.; Schmidt, M. Recipients potentially infected with parvovirus B19 by red blood cell products. *Transfusion* **2011**, *51*, 129–136.
872. Howard, K.J.; Mayer, T.G.; Neblett, R.; Perez, Y.; Cohen, H.; Gatchel, R.J. Fibromyalgia Syndrome in Chronic Disabling Occupational Musculoskeletal Disorders Prevalence, Risk Factors, and Posttreatment Outcomes. *J. Occup. Environ. Med.* **2010**, *52*, 1186–1191.
873. Howland, R.H. General health, health care utilization, and medical comorbidity in dysthymia. *Int. J. Psychiatry Med.* **1993**, *23*, 211–238.
874. HOYEN, D.O.; MCNAMARA, I. GENERAL-PRACTITIONERS EXPERIENCE OF THE CHRONIC FATIGUE SYNDROME. *Br. J. Gen. Pract.* **1991**, *41*, 324–326.
875. HOYEN, D.O.; Shanks, M.; Ho-Yen, D.O.; Shanks, M. CHRONIC FATIGUE SYNDROME - PREVALENCE STUDY OVERLOOKED. *Br. Med. J.* **1994**, *308*, 1299.
876. Hsu, P.-F.; Chuang, S.-Y.; Yu, W.-C.; Leu, H.-B.; Chan, W.-L.; Chen, C.-H. The impacts of serum uric acid on arterial hemodynamics and cardiovascular risks. *Acta Cardiol. Sin.* **2013**, *29*, 142–150.

877. Hu, H.; Baines, C. Recent insights into 3 underrecognized conditions: Myalgic encephalomyelitis–chronic fatigue syndrome, fibromyalgia, and environmental sensitivities–multiple chemical sensitivity [Observations récentes concernant 3 troubles de santé mal reconnus: Encéphalomyélite myalgique/syndrome de fatigue chronique, fibromyalgie et manifestations d'intolérance au milieu/de sensibilité chimique multiple]. *Can. Fam. Physician* **2018**, *64*, 413–421. Work in French
878. Huang, C.Y.; Chung, S.D.; Kao, L.T.; Lin, H.C.; Wang, L.H. Statin Use Is Associated with Bladder Pain Syndrome/Interstitial Cystitis: A Population-Based Case-Control Study. *Urol. Int.* **2015**, *95*, 227–232.
879. Huang, W.; See, D.; Tilles, J. The prevalence of Mycoplasma Incognitus in normal controls or patients with AIDS or the chronic fatigue syndrome. *Clin. Infect. Dis.* **1997**, *25*, 484.
880. Hughes, A.-M.; Lucas, R.M.; Mcmichael, A.J.; Dwyer, T.; Pender, M.P.; van der Mei, I.; Taylor, B. V; Valery, P.; Chapman, C.; Coulthard, A.; et al. Early-life hygiene-related factors affect risk of central nervous system demyelination and asthma differentially. *Clin. Exp. Immunol.* **2013**, *172*, 466–474.
881. Hughes, G.; Martinez, C.; Myon, E.; Taieb, C.; Wessely, S. The impact of a diagnosis of fibromyalgia on health care resource use by primary care patients in the UK - An observational study based on clinical practice. *ARTHRITIS Rheum.* **2006**, *54*, 177–183.
882. Hughes, J.L. Illness narrative and chronic fatigue syndrome/myalgic encephalomyelitis: A review. *Br. J. Occup. Ther.* **2002**, *65*, 9–14.
883. Huibers, M.J.H.; Beurskens, A.J.H.M.; Prins, J.B.; Kant, I.J.; Bazelmans, E.; Van Schayck, C.P.; Knottnerus, J.A.; Bleijenberg, G. Fatigue, burnout, and chronic fatigue syndrome among employees on sick leave: do attributions make the difference? *Occup. Environ. Med.* **2003**, *60 Suppl 1*, i26-31.
884. Huibers, M.J.H.; Kant, I.J.; Swaen, G.M.H.; Kasl, S. V Prevalence of chronic fatigue syndrome-like caseness in the working population: results from the Maastricht cohort study. *Occup. Environ. Med.* **2004**, *61*, 464–466.
885. Huibers, M.J.H.; Bultmann, U.; Kasl, S. V; Kant, I.; van Amelsvoort, L.G.P.M.; van Schayck, C.P.; Swaen, G.M.H. Predicting the two-year course of unexplained fatigue and the onset of long-term sickness absence in fatigued employees: results from the Maastricht Cohort Study. *J. Occup. Environ. Med.* **2004**, *46*, 1041–1047.
886. Huibers, M.J.H.; Kant, I.J.; Knottnerus, J.A.; Bleijenberg, G.; Swaen, G.M.H.; Kasl, S. V Development of the chronic fatigue syndrome in severely fatigued employees: predictors of outcome in the Maastricht cohort study. *J. Epidemiol. Community Health* **2004**, *58*, 877–882.
887. Huisman-de Waal, G.; Versleijen, M.; van Achterberg, T.; Jansen, J.B.; Sauerwein, H.; Schoonhoven, L.; Wanten, G. Psychosocial Complaints Are Associated With Venous Access-Device Related Complications in Patients on Home Parenteral Nutrition. *J. Parenter. Enter. Nutr.* **2011**, *35*, 588–595.
888. Humphrey, L.; Arbuckle, R.; Mease, P.; Williams, D.A.; Samsoe, B.D.; Gilbert, C. Fatigue in fibromyalgia: a conceptual model informed by patient interviews. *BMC Musculoskelet. Disord.* **2010**, *11*, 216.
889. Hunsaker, D.H.; Riffenburgh, R.H. Snoring significance in patients undergoing home sleep studies. *Otolaryngol. Head. Neck Surg.* **2006**, *134*, 756–760.
890. Hunskar, G.S.; Langeland, N.; Wensaas, K.-A.; Hanevik, K.; Eide, G.E.; Morch, K.; Rortveit, G. The impact of atopic disease on the risk of post-infectious fatigue and irritable bowel syndrome 3 years after Giardia infection. A historic cohort study. *Scand. J. Gastroenterol.* **2012**, *47*, 956–961.

891. Hunt, S.C.; Richardson, R.D.; Engel, C.C.; Atkins, D.C.; McFall, M. Gulf war veterans' illnesses: A pilot study of the relationship of illness beliefs to symptom severity and functional health status. *J. Occup. Environ. Med.* **2004**, *46*, 818–827.
892. Hunter, M. Chronic fatigue syndrome could be costing UK economy pound 100m a year, study says. *BMJ* 2011, *343*, d5905.
893. Hutchinson, C. V; Maltby, J.; Badham, S.P.; Jason, L.A. Vision-related symptoms as a clinical feature of chronic fatigue syndrome/myalgic encephalomyelitis? Evidence from the DePaul Symptom Questionnaire. *Br. J. Ophthalmol.* **2014**, *98*, 144–145.
894. Hvidberg, M.F.; Brinth, L.S.; Olesen, A. V; Petersen, K.D.; Ehlers, L. The Health-Related Quality of Life for Patients with Myalgic Encephalomyelitis/Chronic Fatigue Syndrome (ME/CFS). *PLoS One* **2015**, *10*.
895. Hyams, K.C. Lessons derived from evaluating Gulf War syndrome: suggested guidelines for investigating possible outbreaks of new diseases. *Psychosom. Med.* 1998, *60*, 137–139.
896. HYAMS, K.C.; HANSON, K.; WIGNALL, F.S.; ESCAMILLA, J.; OLDFIELD, E.C. THE IMPACT OF INFECTIOUS-DISEASES ON THE HEALTH OF US TROOPS DEPLOYED TO THE PERSIAN-GULF DURING OPERATION-DESERT-SHIELD AND OPERATION-DESERT-STORM. *Clin. Infect. Dis.* **1995**, *20*, 1497–1504.
897. Hyams, K.C. The investigation of chronic fatigue syndrome: a case-study of the limitations of inductive inferences and non-falsifiable hypotheses in medical research. *Med. Hypotheses* **2003**, *60*, 760–766.
898. Hyyppa, M.T.; Lindholm, T.; Lehtinen, V.; Puukka, P. Self-perceived fatigue and cortisol secretion in a community sample. *J. Psychosom. Res.* **1993**, *37*, 589–594.
899. Iacob, E.; Light, A.R.; Donaldson, G.W.; Okifuji, A.; Hughen, R.W.; White, A.T.; Light, K.C. Gene Expression Factor Analysis to Differentiate Pathways Linked to Fibromyalgia, Chronic Fatigue Syndrome, and Depression in a Diverse Patient Sample. *Arthritis Care Res. (Hoboken).* **2016**, *68*, 132–140.
900. Ibeziako, P.; Bujoreanu, S. Approach to psychosomatic illness in adolescents. *Curr. Opin. Pediatr.* **2011**, *23*, 384–389.
901. Ickmans, K.; Meeus, M.; De Kooning, M.; Lambrecht, L.; Pattyn, N.; Nijs, J. Associations Between Cognitive Performance and Pain in Chronic Fatigue Syndrome: Comorbidity with Fibromyalgia Does Matter. *Pain Physician* **2015**, *18*, E841-52.
902. Ickmans, K.; Meeus, M.; De Kooning, M.; Lambrecht, L.; Pattyn, N.; Nijs, J. Can recovery of peripheral muscle function predict cognitive task performance in chronic fatigue syndrome with and without fibromyalgia? *Phys. Ther.* **2014**, *94*, 511–522.
903. Ickmans, K.; Meeus, M.; Kos, D.; Clarys, P.; Meersdom, G.; Lambrecht, L.; Pattyn, N.; Nijs, J. Cognitive performance is of clinical importance, but is unrelated to pain severity in women with chronic fatigue syndrome. *Clin. Rheumatol.* **2013**, *32*, 1475–1485.
904. Ihlebaek, C.; Brage, S.; Natvig, B.; Bruusgaard, D. [Occurrence of musculoskeletal disorders in Norway]. *Tidsskr. Nor. Laegeforen.* **2010**, *130*, 2365–2368. Work in Norwegian
905. Ikuta, K.; Ibrahim, M.S.; Kobayashi, T.; Tomonaga, K. Borna disease virus and infection in humans. *Front. Biosci.* **2002**, *7*, d470-95.
906. Irlbeck, D.M.; Vernon, S.D.; McCleary, K.K.; Bateman, L.; Klimas, N.G.; Lapp, C.W.; Peterson, D.L.; Brown, J.R.; Remlinger, K.S.; Wilfret, D.A.; et al. No

association found between the detection of either xenotropic murine leukemia virus-related virus or polytropic murine leukemia virus and chronic fatigue syndrome in a blinded, multi-site, prospective study by the establishment and use of the SolveCFS BioBank. *BMC Res. Notes* **2014**, *7*.
907. Ishihara, S.; Okada, S.; Wakiguchi, H.; Kurashige, T.; Morishima, T.; Kawa-Ha, K. Chronic active Epstein-Barr virus infection in children in Japan. *Acta Paediatr.* **1995**, *84*, 1271–1275.
908. Ismail, K.; Kent, K.; Sherwood, R.; Hull, L.; Seed, P.; David, A.S.; Wessely, S. Chronic fatigue syndrome and related disorders in UK veterans of the Gulf War 1990-1991: results from a two-phase cohort study. *Psychol. Med.* **2008**, *38*, 953–961.
909. Ismail, K.; Lewis, G. Multi-symptom illnesses, unexplained illness and Gulf War Syndrome. *Philos. Trans. R. Soc. Lond. B. Biol. Sci.* **2006**, *361*, 543–551.
910. Israeli, E.; Pardo, A. The sick building syndrome as a part of the autoimmune (auto-inflammatory) syndrome induced by adjuvants. *Mod. Rheumatol.* **2011**, *21*, 235–239.
911. Itoh, Y.; Hamada, H.; Imai, T.; Seki, T.; Igarashi, T.; Yuge, K.; Fukunaga, Y.; Yamamoto, M. Antinuclear antibodies in children with chronic nonspecific complaints. *Autoimmunity* **1997**, *25*, 243–250.
912. Itoh, Y.; Shigemori, T.; Igarashi, T.; Fukunaga, Y. Fibromyalgia and chronic fatigue syndrome in children. *Pediatr. Int.* **2012**, *54*, 266–271.
913. Iyengar, S.; Levine, P.H.; Ablashi, D.; Neequaye, J.; Pearson, G.R. Sero-epidemiological investigations on human herpesvirus 6 (HHV-6) infections using a newly developed early antigen assay. *Int. J. cancer* **1991**, *49*, 551–557.
914. Jacob, G.; Biaggioni, I. Idiopathic orthostatic intolerance and postural tachycardia syndromes. *Am. J. Med. Sci.* **1999**, *317*, 88–101.
915. Jacobsen, H.B.; Bjorngaard, J.H.; Hara, K.W.; Borchgrevink, P.C.; Woodhouse, A.; Landro, N.I.; Harris, A.; Stiles, T.C. The Role of Stress in Absenteeism: Cortisol Responsiveness among Patients on Long-Term Sick Leave. *PLoS One* **2014**, *9*.
916. JAGER, L. CHRONIC FATIGUE SYNDROME - IMMUNOLOGICAL AND ALLERGIC ASPECTS. *ALLERGOLOGIE* **1995**, *18*, 228–233.
917. Jager, L. Chronic fatigue syndrome - Immunological and allergic aspects [CHRONIC FATIGUE SYNDROME - IMMUNOLOGISCHE UND ALLERGOLOGISCHE ASPEKTE]. *Allergologie* **1995**, *18*, 228–233. Work in German
918. Jain, S.S.; DeLisa, J.A. Chronic fatigue syndrome: a literature review from a physiatric perspective. *Am. J. Phys. Med. Rehabil.* **1998**, *77*, 160–167.
919. Jain, V.; Arunkumar, A.; Kingdon, C.; Lacerda, E.; Nacul, L. Prevalence of and risk factors for severe cognitive and sleep symptoms in ME/CFS and MS. *BMC Neurol.* **2017**, *17*, 117.
920. Jalnapurkar, I.; Rafika, N.; Tassone, F.; Hagerman, R. Immune mediated disorders in women with a fragile X expansion and FXTAS. *Am. J. Med. Genet. Part A* **2015**, *167*, 190–197.
921. James, M.F.M. Magnesium in obstetrics. *Best Pract. Res. Clin. Obstet. Gynaecol.* **2010**, *24*, 327–337.
922. Jammes, Y.; Steinberg, J.G.; Delliaux, S. Chronic fatigue syndrome: Acute infection and history of physical activity affect resting levels and response to exercise of plasma oxidant/antioxidant status and heat shock proteins. *J. Intern. Med.* **2012**, *272*, 74–84.

923. Janal, M.N.; Ciccone, D.S.; Natelson, B.H. Sub-typing CFS patients on the basis of "minor" symptoms. *Biol. Psychol.* **2006**, *73*, 124–131.
924. Janal, M.N.; Raphael, K.G.; Cook, D.B.; Sirois, D.A.; Nemelivsky, L.; Staud, R. Thermal temporal summation and decay of after-sensations in temporomandibular myofascial pain patients with and without comorbid fibromyalgia. *J. Pain Res.* **2016**, *9*, 641–652.
925. Janca, A.; Isaac, M.; Ventouras, J. Towards better understanding and management of somatoform disorders. *Int. Rev. Psychiatry* **2006**, *18*, 5–12.
926. Janse, A.; Wiborg, J.F.; Bleijenberg, G.; Tummers, M.; Knoop, H. The Efficacy of Guided Self-instruction for Patients With Idiopathic Chronic Fatigue: A Randomized Controlled Trial. *J. Consult. Clin. Psychol.* **2016**, *84*, 377–388.
927. Janssens, K.A.M.; Zijlema, W.L.; Joustra, M.L.; Rosmalen, J.G.M. Mood and Anxiety Disorders in Chronic Fatigue Syndrome, Fibromyalgia, and Irritable Bowel Syndrome: Results From the LifeLines Cohort Study. *Psychosom. Med.* **2015**, *77*, 449–457.
928. Jarjour, I.T.; Jarjour, L.K. Low iron storage in children and adolescents with neurally mediated syncope. *J. Pediatr.* **2008**, *153*, 40–44.
929. Jason, L.A.; Bell, D.S.; Rowe, K.; Van Hoof, E.L.S.; Jordan, K.; Lapp, C.; Gurwitt, A.; Miike, T.; Torres-Harding, S.; De Meirleir, K. A pediatric case definition for myalgic encephalomyelitis and chronic fatigue syndrome. *J. Chronic Fatigue Syndr.* **2006**, *13*, 1–44.
930. Jason, L.A.; Benton, M.C.; Valentine, L.; Johnson, A.; Torres-Harding, S. The Economic impact of ME/CFS: Individual and societal costs. *Dyn. Med.* **2008**, *7*.
931. Jason, L.A.; Evans, M.; Brown, M.; Porter, N. What is Fatigue? Pathological and Nonpathological Fatigue. *PM R* **2010**, *2*, 327–331.
932. Jason, L.A.; Fitzgibbon, G.; Taylor, S.L.; Johnson, S.; Salina, D. Strategies in identifying people with chronic fatigue syndrome. *J. Community Psychol.* **1993**, *21*, 339–344.
933. Jason, L.A.; Jordan, K.M.; Richman, J.A.; Rademaker, A.W.; Huang, C.-F.; Mccready, W.; Shlaes, J.; King, C.P.; Landis, D.; Torres, S.; et al. A community-based study of prolonged fatigue and chronic fatigue. *J. Health Psychol.* **1999**, *4*, 9–26.
934. Jason, L.A.; King, C.P.; Richman, J.A.; Taylor, R.R.; Torres, S.R.; Song, S. U.S. Case definition of chronic fatigue syndrome: Diagnostic and theoretical issues. *J. Chronic Fatigue Syndr.* **1999**, *5*, 3–33.
935. Jason, L.A.; Lapp, C.; Kimberly Kenney, K.; Lupton, T. *An innovative approach in training health care workers to diagnose and manage patients with CFS*; 2010;
936. Jason, L.A.; Porter, N.; Brown, M.; Brown, A.; Evans, M. A constructive debate with the cdc on the empirical case definition of chronic fatigue syndrome. *J. Disabil. Policy Stud.* **2010**, *20*, 251–256.
937. Jason, L.A.; Porter, N.; Hunnell, J.; Brown, A.; Rademaker, A.; Richman, J.A. A Natural History Study of Chronic Fatigue Syndrome. *Rehabil. Psychol.* **2011**, *56*, 32–42.
938. Jason, L.A.; Richman, J.A. How science can stigmatize: The case of chronic fatigue syndrome. *J. Chronic Fatigue Syndr.* **2008**, *14*, 85–103.
939. Jason, L.A.; Richman, J.A.; Friedberg, F.; Wagner, L.; Taylor, R.; Jordan, K.M. Politics, science, and the emergence of a new disease. The case of chronic fatigue syndrome. *Am. Psychol.* **1997**, *52*, 973–983.

940. Jason, L.A.; Richman, J.A.; Rademaker, A.W.; Jordan, K.M.; Plioplys, A. V; Taylor, R.R.; McCready, W.; Huang, C.F.; Plioplys, S. A community-based study of chronic fatigue syndrome. *Arch. Intern. Med.* **1999**, *159*, 2129–2137.
941. Jason, L.A.; Ropacki, M.T.; Santoro, N.B.; Richman, J.A.; Heatherly, W.; Taylor, R.; Ferrari, J.R.; Haney-Davis, T.M.; Rademaker, A.; Dupuis, J.; et al. A screening instrument for chronic fatigue syndrome: Reliability and validity. *J. Chronic Fatigue Syndr.* **1997**, *3*, 39–59.
942. Jason, L.A.; Sunnquist, M.; Brown, A.; McManimen, S.; Furst, J. Reflections on the Institute of Medicine's systemic exertion intolerance disease. *Pol. Arch. Med. Wewn.* **2015**, *125*, 576–581.
943. Jason, L.A.; Taylor, R.R. Applying cluster analysis to define a typology of chronic fatigue syndrome in a medically-evaluated, random community sample. *Psychol. Health* **2002**, *17*, 323–337.
944. Jason, L.A.; Taylor, R.R.; Carrico, A.W. A community-based study of seasonal variation in the onset of chronic fatigue syndrome and idiopathic chronic fatigue. *Chronobiol. Int.* **2001**, *18*, 315–319.
945. Jason, L.A.; Taylor, R.R.; Kennedy, C.L. Chronic fatigue syndrome, fibromyalgia, and multiple chemical sensitivities in a community-based sample of persons with chronic fatigue syndrome-like symptoms. *Psychosom. Med.* **2000**, *62*, 655–663.
946. Jason, L.A.; Taylor, R.R.; Kennedy, C.L.; Jordan, K.M.; Song, S.; Johnson, D.; Torres-Harding, S. Chronic Fatigue Syndrome: Symptom subtypes in a community based sample. *Women Heal.* **2003**, *37*, 1–13.
947. Jason, L.A.; Taylor, R.R.; Kennedy, C.L.; Jordan, K.; Song, S.; Johnson, D.E.; Torres, S.R. Chronic fatigue syndrome: sociodemographic subtypes in a community-based sample. *Eval. Health Prof.* **2000**, *23*, 243–263.
948. Jason, L.A.; Taylor, R.R.; Kennedy, C.L.; Song, S.; Johnson, D.; Torres, S. Chronic fatigue syndrome: occupation, medical utilization, and subtypes in a community-based sample. *J. Nerv. Ment. Dis.* **2000**, *188*, 568–576.
949. Jason, L.A.; Taylor, R.R.; Plioplys, S.; Stepanek, Z.; Shlaes, J. Evaluating Attributions for an Illness Based Upon the Name: Chronic Fatigue Syndrome, Myalgic Encephalopathy and Florence Nightingale Disease. *Am. J. Community Psychol.* **2002**, *30*, 133–148.
950. Jason, L.A.; Taylor, R.; Wagner, L.; Holden, J.; Ferrari, J.R.; Plioplys, A. V; Plioplys, S.; Lipkin, D.; Papernik, M. Estimating rates of chronic fatigue syndrome from a community-based sample: a pilot study. *Am. J. Community Psychol.* **1995**, *23*, 557–568.
951. Jason, L.A.; Taylor, S.L.; Johnson, S.; Goldston, S.E.; Salina, D.; Bishop, P.; Wagner, L. Prevalence of chronic fatigue syndrome-related symptoms among nurses. *Eval. Health Prof.* **1993**, *16*, 385–399. Proceedigns of the Annual Meeting of the American-Educational-Research-Association in Chicago, USA.
952. Jason, L.A.; Wagner, L.; Rosenthal, S.; Goodlatte, J.; Lipkin, D.; Papernik, M.; Plioplys, S.; Plioplys, A. V Estimating the prevalence of chronic fatigue syndrome among nurses. *Am. J. Med.* **1998**, *105*, 91S–93S.
953. Jason, L.A.; Wagner, L.; Taylor, R.; Ropacki, M.T.; Shlaes, J.; Ferrari, J.R.; Slavich, S.P.; Stenzel, C. Chronic fatigue syndrome: A new challenge for health care professionals. *J. Community Psychol.* **1995**, *23*, 143–164.
954. Jason, L.A. Small wins matter in advocacy movements: giving voice to patients. *Am. J. Community Psychol.* **2012**, *49*, 307–316.

955. Jason, L.A.; Corradi, K.; Gress, S.; Williams, S.; Torres-Harding, S. Causes of death among patients with chronic fatigue syndrome. *Health Care Women Int.* **2006**, *27*, 615–626.
956. Jason, L.A.; Fox, P.A.; Gleason, K.D. The importance of a research case definition. *FATIGUE-BIOMEDICINE Heal. Behav.* **2018**, *6*, 52–58.
957. Jason, L.A.; McManimen, S.; Sunnquist, M.; Brown, A.; Furst, J.; Newton, J.L.; Strand, E.B. Case definitions integrating empiric and consensus perspectives. *FATIGUE-BIOMEDICINE Heal. Behav.* **2016**, *4*, 1–23.
958. Jason, L.A.; McManimen, S.; Sunnquist, M.; Newton, J.L.; Strand, E.B. Clinical criteria versus a possible research case definition in chronic fatigue syndrome/myalgic encephalomyelitis. *FATIGUE-BIOMEDICINE Heal. Behav.* **2017**, *5*, 89–102.
959. Jason, L.A.; Plioplys, A. V; Torres-Harding, S.; Corradi, K. Comparing symptoms of chronic fatigue syndrome in a community-based versus tertiary care sample. *J. Health Psychol.* **2003**, *8*, 459–464.
960. Jason, L.A.; Porter, N.; Hunnell, J.; Rademaker, A.; Richman, J.A. CFS prevalence and risk factors over time. *J. Health Psychol.* **2011**, *16*, 445–456.
961. Jason, L.A.; Reed, J. The use of mixed methods in studying a chronic illness. *Heal. Psychol. Behav. Med.* **2015**, *3*, 40–51.
962. Jason, L.A.; Skendrovic, B.; Furst, J.; Brown, A.; Weng, A.; Bronikowski, C. Data mining: comparing the empiric CFS to the Canadian ME/CFS case definition. *J. Clin. Psychol.* **2012**, *68*, 41–49.
963. Jason, L.A.; Sunnquist, M.; Brown, A.; Reed, J. Defining Essential Features of Myalgic Encephalomyelitis and Chronic Fatigue Syndrome. *J. Hum. Behav. Soc. Environ.* **2015**, *25*, 657–674.
964. Jason, L.A.; Sunnquist, M.; Kot, B.; Brown, A. Unintended Consequences of not Specifying Exclusionary Illnesses for Systemic Exertion Intolerance Disease. *DIAGNOSTICS* **2015**, *5*, 272–286.
965. Jason, L.A.; Taylor, R.R.; Kennedy, C.L.; Jordan, K.; Huang, C.-F.; Torres-Harding, S.; Song, S.; Johnson, D. A factor analysis of chronic fatigue symptoms in a community-based sample. *Soc. Psychiatry Psychiatr. Epidemiol.* **2002**, *37*, 183–189.
966. Jason, L.A.; Unger, E.R.; Dimitrakoff, J.D.; Fagin, A.P.; Houghton, M.; Cook, D.B.; Marshall, G.D.J.; Klimas, N.; Snell, C. Minimum data elements for research reports on CFS. *Brain. Behav. Immun.* **2012**, *26*, 401–406.
967. Javaras, K.N.; Pope, H.G.; Lalonde, J.K.; Roberts, J.L.; Nillni, Y.I.; Laird, N.M.; Bulik, C.M.; Crow, S.J.; McElroy, S.L.; Walsh, B.T.; et al. Co-occurrence of binge eating disorder with psychiatric and medical disorders. *J. Clin. Psychiatry* **2008**, *69*, 266–273.
968. Jay, K.; Brandt, M.; Sundstrup, E.; Schraefel, M.C.; Jakobsen, M.D.; Sjogaard, G.; Andersen, L.L. Effect of individually tailored biopsychosocial workplace interventions on chronic musculoskeletal pain, stress and work ability among laboratory technicians: randomized controlled trial protocol. *BMC Musculoskelet. Disord.* **2014**, *15*.
969. Jeffery, D.D.; Bulathsinhala, L.; Kroc, M.; Dorris, J. Prevalence, health care utilization, and costs of fibromyalgia, irritable bowel, and chronic fatigue syndromes in the military health system, 2006-2010. *Mil. Med.* **2014**, *179*, 1021–1029.
970. Jelenova, D.; Prasko, J.; Ociskova, M.; Hruby, R.; Marackova, M.; Slepecky, M.; Vyskocilova, J.; Holubova, M.; Hunkova, M.; Kamaradova, D.; et al. Psychosocial

and psychiatric aspects of children suffered from chronic physical illness. *Act. Nerv. Super. REDIVIVA* **2016**, *58*, 123–131.
971. Jelsness-Jørgensen, L.-P.; Bernklev, T.; Henriksen, M.; Torp, R.; Moum, B.A. Chronic fatigue is more prevalent in patients with inflammatory bowel disease than in healthy controls. *Inflamm. Bowel Dis.* **2011**, *17*, 1564–1572.
972. Jelsness-Jørgensen, L.-P.; Bernklev, T.; Lundin, K.E.A. Fatigue as an extra-intestinal manifestation of celiac disease: A systematic review. *Nutrients* **2018**, *10*.
973. Jenkins, R. Epidemiology: Lessons from the past. *Br. Med. Bull.* **1991**, *47*, 952–965.
974. Jenkins, R. Post-viral fatigue syndrome. Epidemiology: lessons from the past. *Br. Med. Bull.* **1991**, *47*, 952–965.
975. Jensen, Ø.; Bernklev, T.; Gibbs, C.; Moe, R.B.; Hofsø, D.; Jelsness-Jørgensen, L.-P. Fatigue in type 1 diabetes, prevalence, predictors and comparison with the background population. *Diabetes Res. Clin. Pract.* **2018**, *143*, 71–78.
976. Jerome, K.R.; Diem, K.; Huang, M.-L.; Selke, S.; Corey, L.; Buchwald, D. Xenotropic murine leukemia virus-related virus in monozygotic twins discordant for chronic fatigue syndrome. *Diagn. Microbiol. Infect. Dis.* **2011**, *71*, 66–71.
977. Jewell, D.; Logan, A. July focus. *Br. J. Gen. Pract.* **2001**, *51*, 521.
978. Jeziorski, E.; Foulongne, V.; Ludwig, C.; Louhaem, D.; Chiocchia, G.; Segondy, M.; Rodiere, M.; Sitbon, M.; Courgnaud, V. No evidence for XMRV association in pediatric idiopathic diseases in France. *Retrovirology* **2010**, *7*.
979. Ji-hong, W.; Tie-qu, C.; Guo-hua, L.; Lin, L. Effects of the Intelligent-Turtle Massage on the Physical Symptoms and Immune Functions in Patients with Chronic Fatigue Syndrome. *J. Tradit. CHINESE Med.* **2009**, *29*, 24–28.
980. Johanning, E.; Biagini, R.; Hull, D.; Morey, P.; Jarvis, B.; Landsbergis, P. Health and immunology study following exposure to toxigenic fungi (Stachybotrys chartarum) in a water-damaged office environment. *Int. Arch. Occup. Environ. Health* **1996**, *68*, 207–218.
981. Jóhannsdóttir, I.M.; Hjermstad, M.J.; Moum, T.; Wesenberg, F.; Hjorth, L.; Schrøder, H.; Mört, S.; Jónmundsson, G.; Loge, J.H. Increased prevalence of chronic fatigue among survivors of childhood cancers: A population-based study. *Pediatr. Blood Cancer* **2012**, *58*, 415–420.
982. Johansson, S.; Ytterberg, C.; Claesson, I.M.; Lindberg, J.; Hillert, J.; Andersson, M.; Widen Holmqvist, L.; von Koch, L. High concurrent presence of disability in multiple sclerosis. Associations with perceived health. *J. Neurol.* **2007**, *254*, 767–773.
983. Johnson, A.D.; Cohn, C.S. Xenotropic Murine Leukemia Virus-Related Virus (XMRV) and the Safety of the Blood Supply. *Clin. Microbiol. Rev.* **2016**, *29*, 749–757.
984. Johnson, M.; Hammad, A.; Nriagu, J.O.; Savoie, K.; Jamil, H. E. Environmental disease burden in Arab-American communities in the Detroit area: Prevalence and severity. *Ethn. Dis.* **2005**, *15*, S1-43-S1-46.
985. Johnson, R.W.; McElhaney, J. Postherpetic neuralgia in the elderly. *Int. J. Clin. Pract.* **2009**, *63*, 1386–1391.
986. Johnson, S.K. *Chronic Fatigue Syndrome: A Biopsychosocial Perspective*; 2015;
987. Johnson, S.K.; DeLuca, J.; Natelson, B.H. Chronic fatigue syndrome: reviewing the research findings. *Ann. Behav. Med.* **1999**, *21*, 258–271.
988. Johnston, S.C.; Staines, D.R.; Marshall-Gradisnik, S.M. Epidemiological characteristics of chronic fatigue syndrome/myalgic encephalomyelitis in Australian patients. *Clin. Epidemiol.* **2016**, *8*, 97–107.

989. Johnston, S.; Brenu, E.W.; Staines, D.; Marshall-Gradisnik, S. The prevalence of chronic fatigue syndrome/ myalgic encephalomyelitis: A meta-analysis. *Clin. Epidemiol.* **2013**, *5*, 105–110.
990. Johnston, S.C.; Brenu, E.W.; Hardcastle, S.L.; Huth, T.K.; Staines, D.R.; Marshall-Gradisnik, S.M. A comparison of health status in patients meeting alternative definitions for chronic fatigue syndrome/myalgic encephalomyelitis. *Health Qual. Life Outcomes* **2014**, *12*.
991. Johnston, S.; Brenu, E.W.; Staines, D.R.; Marshall-Gradisnik, S. The adoption of chronic fatigue syndrome/myalgic encephalomyelitis case definitions to assess prevalence: a systematic review. *Ann. Epidemiol.* **2013**, *23*, 371–376.
992. Jones, J.F.; Lin, J.-M.S.; Maloney, E.M.; Boneva, R.S.; Nater, U.M.; Unger, E.R.; Reeves, W.C. An evaluation of exclusionary medical/psychiatric conditions in the definition of chronic fatigue syndrome. *BMC Med.* **2009**, *7*, 57.
993. Jones, J.F.; Streib, J.; Baker, S.; Herberger, M. Chronic fatigue syndrome: I. Epstein-Barr virus immune response and molecular epidemiology. *J. Med. Virol.* **1991**, *33*, 151–158.
994. Jones, J.F.; Maloney, E.M.; Boneva, R.S.; Jones, A.-B.; Reeves, W.C. Complementary and alternative medical therapy utilization by people with chronic fatiguing illnesses in the United States. *BMC Complement. Altern. Med.* **2007**, *7*, 12.
995. Jones, J.F.; Nisenbaum, R.; Solomon, L.; Reyes, M.; Reeves, W.C. Chronic fatigue syndrome and other fatiguing illnesses in adolescents: a population-based study. *J. Adolesc. Health* **2004**, *35*, 34–40.
996. Jones, K.D.; Gelbart, T.; Whisenant, T.C.; Waalen, J.; Mondala, T.S.; Ikle, D.N.; Salomon, D.R.; Bennett, R.M.; Kurian, S.M. Genome-wide expression profiling in the peripheral blood of patients with fibromyalgia. *Clin. Exp. Rheumatol.* **2016**, *34*, S89–S98.
997. Jones, N.S. The prevalence of facial pain and purulent sinusitis. *Curr. Opin. Otolaryngol. Head Neck Surg.* **2009**, *17*, 38–42.
998. Jordan, K.M.; Ayers, P.M.; Jahn, S.C.; Taylor, K.K.; Huang, C.-F.; Richman, J.; Jason, L.A. Prevalence of fatigue and chronic fatigue syndrome-like illness in children and adolescents. *J. Chronic Fatigue Syndr.* **2000**, *6*, 3–21.
999. Jordan, K.M.; Jason, L.A.; Mears, C.J.; Katz, B.Z.; Rademaker, A.; Huang, C.-F.; Richman, J.; McCready, W.; Ayers, P.M.; Taylor, K.K. Prevalence of pediatric chronic fatigue syndrome in a community-based sample. *J. Chronic Fatigue Syndr.* **2006**, *13*, 75–78.
1000. Jordan, K.M.; Landis, D.A.; Downey, M.C.; Osterman, S.L.; Thurm, A.E.; Jason, L.A. Chronic fatigue syndrome in children and adolescents: a review. *J. Adolesc. Health* **1998**, *22*, 4–18.
1001. Jordan, K.M.; Mears, C.J.; Katz, B.; Ayers, P.; Taylor, K. Prevalence of chronic fatigue syndrome-like illness in a community-based sample. *J. Adolesc. Heal.* **1999**, *24*, 153.
1002. Jorgensen, R. Chronic fatigue: An evolutionary concept analysis. *J. Adv. Nurs.* **2008**, *63*, 199–207.
1003. Josev, E.K.; Jackson, M.L.; Bei, B.; Trinder, J.; Harvey, A.; Clarke, C.; Snodgrass, K.; Scheinberg, A.; Knight, S.J. Sleep Quality in Adolescents With Chronic Fatigue Syndrome/Myalgic Encephalomyelitis (CFS/ME). *J. Clin. sleep Med. JCSM Off. Publ. Am. Acad. Sleep Med.* **2017**, *13*, 1057–1066.
1004. Joustra, M.L.; Zijlema, W.L.; Rosmalen, J.G.M.; Janssens, K.A.M. Physical Activity and Sleep in Chronic Fatigue Syndrome and Fibromyalgia Syndrome:

Associations with Symptom Severity in the General Population Cohort LifeLines. *PAIN Res. Manag.* **2018**.
1005. Joyce, J.; Hotopf, M.; Wessely, S. The prognosis of chronic fatigue and chronic fatigue syndrome: a systematic review. *QJM* **1997**, *90*, 223–233.
1006. Kaabia, N.; Letaief, A. Q fever in Tunisia. *Pathol. Biol.* **2009**, *57*, 439–443.
1007. Kaabia, N.; Letaief, A. Q fever in Tunisia [La fièvre Q en Tunisie]. *Pathol. Biol.* **2009**, *57*, 439–443. Work in French
1008. Kabelitz, D.; Kremer, B.; Lode, H.; Meinertz, T.; Sauerbruch, T.; Sterry, W. Fibromyalgia and chronic fatigue syndrome - Similarities and differences. *Dtsch. MEDIZINISCHE WOCHENSCHRIFT* **1996**, *121*, 1165–1168.
1009. Kakumanu, S.S.; Mende, C.N.; Lehman, E.B.; Hughes, K.; Craig, T.J. Effect of topical nasal corticosteroids on patients with chronic fatigue syndrome and rhinitis. *J. Am. Osteopath. Assoc.* **2003**, *103*, 423–427.
1010. Kalkman, J.S.; Schillings, M.L.; Zwarts, M.J.; van Engelen, B.G.M.; Bleijenberg, G. Psychiatric disorders appear equally in patients with myotonic dystrophy, facioscapulohumeral dystrophy, and hereditary motor and sensory neuropathy type I. *ACTA Neurol. Scand.* **2007**, *115*, 265–270.
1011. Kanaan, R.A.A.; Lepine, J.P.; Wessely, S.C. The association or otherwise of the functional somatic syndromes. *Psychosom. Med.* **2007**, *69*, 855–859. Proceedings of the Conference on the Futute of Psychiatric Diagnosis - Refining the Research Agenda in Beijing, China.
1012. Kang, H.K.; Li, B.; Mahan, C.M.; Eisen, S.A.; Engel, C.C. Health of US veterans of 1991 Gulf War: A follow-up survey in 10 years. *J. Occup. Environ. Med.* **2009**, *51*, 401–410.
1013. Kang, H.K.; Natelson, B.H.; Mahan, C.M.; Lee, K.Y.; Murphy, F.M. Post-traumatic stress disorder and chronic fatigue syndrome-like illness among Gulf War veterans: a population-based survey of 30,000 veterans. *Am. J. Epidemiol.* **2003**, *157*, 141–148.
1014. Kano, O.; Iwamoto, K.; Cridebring, D.; Ikeda, K.; Iwasaki, Y. Relationship between vitamin D and depression in multiple sclerosis. *Acta Neurol. Scand.* 2012, *125*, e25; author reply e26-7.
1015. Kant, I.J.; Jansen, N.W.H.; Van Amelsvoort, L.G.P.M.; Huibers, M.J.H. Course, consequences and treatment of prolonged fatigue among workers: An overview of findings from the Maastricht Cohort Study [Beloop, consequenties en behandeling van langdurige vermoeidheid onder werknemers: Overzicht van bevindingen uit de Maastrichtse Cohort Studie]. *Tijdschr. Psychiatr.* **2007**, *49*, 547–554. Work in Dutch
1016. Kant, I.; Jansen, N.W.H.; van Amelsvoort, L.G.P.M.; Huibers, M.J.H.; van Amelsvoort, L.G.P.M.; Kant, I. [Aetiology of prolonged fatigue among workers. An overview of findings from the Maastricht Cohort Study]. *Tijdschr. Psychiatr.* **2007**, *49*, 547–554.
1017. Kapfhammer, H.P.; Rothenhausler, H.B. [Chronic fatigue syndrome]. *MMW Fortschr. Med.* **2004**, *146*, 29,31-33.
1018. Kapfhammer, H.-P.; Rothenhäusler, H.-B. Chronic fatigue syndrome [Das chronische müdigkeitssyndrom]. *MMW-Fortschritte der Medizin* **2004**, *146*, 29–33. Work in German
1019. Kapur, N.; Webb, R. Suicide risk in people with chronic fatigue syndrome. *Lancet (London, England)* **2016**, *387*, 1596–1597.

1020. Kara, I.H.; Demir, D.; Erdem, O.; Sayin, G.T.; Yildiz, N.; Yaman, H. Chronic Fatigue Syndrome among nurses and healthcare workers in a research hospital in Turkey. *Soc. Behav. Pers.* **2008**, *36*, 585–590.
1021. Karlson, R. En evolusjonær forståelse av kronisk utmattelsessyndrom. *Tidsskr. den Nor. Laegeforening* **2012**, *132*, 400–401. Work in Norwegian
1022. Karthikeyan, P.; Ramalingam, K.P. Meningitis: is a major cause of disability amongst Papua New Guinea children? *Disabil. Rehabil.* **2012**, *34*, 1585–1588.
1023. Kasatkin, D.S.; Spirin, N.N. [Possible mechanisms of chronic fatigue syndrome in multiple sclerosis]. *Zhurnal Nevrol. i psikhiatrii Im. S.S. Korsakova* **2006**, *Spec No 3*, 87–91. Work in Russian
1024. Katerndahl, D.A.; Bell, I.R.; Palmer, R.F.; Miller, C.S. Chemical Intolerance in Primary Care Settings: Prevalence, Comorbidity, and Outcomes. *Ann. Fam. Med.* **2012**, *10*, 357–365.
1025. Kato, K.; Sullivan, P.F.; Evengard, B.; Pedersen, N.L. A population-based twin study of functional somatic syndromes. *Psychol. Med.* **2009**, *39*, 497–505.
1026. Kato, K.; Sullivan, P.F.; Evengard, B.; Pedersen, N.L. Premorbid predictors of chronic fatigue. *Arch. Gen. Psychiatry* **2006**, *63*, 1267–1272.
1027. Kato, K.; Sullivan, P.F.; Evengard, B.; Pedersen, N.L. Chronic widespread pain and its comorbidities: a population-based study. *Arch. Intern. Med.* **2006**, *166*, 1649–1654.
1028. Kato, K.; Sullivan, P.F.; Pedersen, N.L. Latent class analysis of functional somatic symptoms in a population-based sample of twins. *J. Psychosom. Res.* **2010**, *68*, 447–453.
1029. Kato, Y.H.; Yamate, M.; Tsujikawa, M.; Nishigaki, H.; Tanaka, Y.; Yunoki, M.; Kuratsune, H.; Watanabe, Y.; Ikuta, K. No apparent difference in the prevalence of parvovirus B19 infection between chronic fatigue syndrome patients and healthy controls in Japan. *J. Clin. Virol.* **2009**, *44*, 246–247.
1030. Katon, W.J.; Buchwald, D.S.; Simon, G.E.; Russo, J.E.; Mease, P.J. Psychiatric illness in patients with chronic fatigue and those with rheumatoid arthritis. *J. Gen. Intern. Med.* **1991**, *6*, 277–285.
1031. Katon, W.; Russo, J. Chronic fatigue syndrome criteria. A critique of the requirement for multiple physical complaints. *Arch. Intern. Med.* **1992**, *152,* 1604–1609.
1032. Katz, B.Z.; Jason, L.A. Chronic fatigue syndrome following infections in adolescents. *Curr. Opin. Pediatr.* **2013**, *25*, 95–102.
1033. Katz, B.Z.; Shiraishi, Y.; Mears, C.J.; Binns, H.J.; Taylor, R. Chronic fatigue syndrome after infectious mononucleosis in adolescents. *Pediatrics* **2009**, *124*, 189–193. Proceedings of the Annual Meeting of the Pediatric-Academic-Societies/Academic-Pediatric-Association/Society-for-Pediatric-Research National Conference in Honolulu, USA
1034. Kawada, T. Chronic fatigue syndrome in adolescents: Definition and epidemiological characteristics. *J. Paediatr. Child Health* **2014**, *50*, 840.
1035. Kawakami, N.; Iwata, N.; Fujihara, S.; Kitamura, T. Prevalence of chronic fatigue syndrome in a community population in Japan. *Tohoku J. Exp. Med.* **1998**, *186*, 33–41.
1036. Kazar, J. Coxiella burnetii infection. In Proceedings of the RICKETTSIOSES: FROM GENOME TO PROTEOME, PATHOBIOLOGY, AND RICKETTSIAE AS AN INTERNATIONAL THREAT; Hechemy, KE and Oteo, JA and Raoult, DA and Silverman, DJ and Blanco, JR, Ed.; 2005; Vol. 1063, pp. 105–114.

1037. Kazmirchuk, V.I.; Tsaryk, V. V; Sydorenko, O.I.; Solon’ko, I.I.; Diuseieva, V. V; Voĭtiuk, T. V FREQUENCY OF IMMUNOGLOBULIN E DEFICIENCY AMONG PATIENTS WITH IMMUNODEPENDENT DISORDERS. *Lik. Sprava* **2014**, 3–9.
1038. Kearney, M.; Maldarelli, F. Current status of xenotropic murine leukemia virus-related retrovirus in chronic fatigue syndrome and prostate cancer: reach for a scorecard, not a prescription pad. *J. Infect. Dis.* 2010, *202*, 1463–1466.
1039. Keightley, P.; Pavli, P.; Platten, J.; Looi, J.C. Gut feelings 1. Mind, mood and gut in irritable bowel syndrome: Approaches to psychiatric care. *Australas. Psychiatry* **2015**, *23*, 403–406.
1040. Keller, J.; Chen, Y.-K.; Lin, H.-C. Association of bladder pain syndrome/interstitial cystitis with urinary calculus: A nationwide population-based study. *Int. Urogynecol. J. Pelvic Floor Dysfunct.* **2013**, *24*, 565–571.
1041. Keller, J.J.; Liu, S.-P.; Lin, H.-C. A Case-Control Study on the Association Between Rheumatoid Arthritis and Bladder Pain Syndrome/Interstitial Cystitis. *Neurourol. Urodyn.* **2013**, *32*, 980–985.
1042. Kellett, S.; Webb, K.; Wilkinson, N.; Bliss, P.; Ayers, T.; Hardy, G. Developing Services for Patients with Depression or Anxiety in the Context of Long-term Physical Health Conditions and Medically Unexplained Symptoms: Evaluation of an IAPT Pathfinder Site. *Behav. Cogn. Psychother.* **2016**, *44*, 553–567.
1043. Kelsall, H.L.; Sim, M.R.; Forbes, A.B.; Glass, D.C.; McKenzie, D.P.; Ikin, J.F.; Abramson, M.J.; Blizzard, L.; Ittak, P. Symptoms and medical conditions in Australian veterans of the 1991 Gulf War: relation to immunisations and other Gulf War exposures. *Occup. Environ. Med.* **2004**, *61*, 1006–1013.
1044. Kelsall, H.; Sim, M.; McKenzie, D.; Forbes, A.; Leder, K.; Glass, D.; Ikin, J.; McFarlane, A. Medically evaluated psychological and physical health of Australian Gulf War veterans with chronic fatigue. *J. Psychosom. Res.* **2006**, *60*, 575–584.
1045. Kempke, S.; Luyten, P.; Claes, S.; Goossens, L.; Bekaert, P.; Van Wambeke, P.; Van Houdenhove, B. Self-critical perfectionism and its relationship to fatigue and pain in the daily flow of life in patients with chronic fatigue syndrome. *Psychol. Med.* **2013**, *43*, 995–1002.
1046. Kempke, S.; Luyten, P.; Claes, S.; Van Wambeke, P.; Bekaert, P.; Goossens, L.; Van Houdenhove, B. The prevalence and impact of early childhood trauma in Chronic Fatigue Syndrome. *J. Psychiatr. Res.* **2013**, *47*, 664–669.
1047. Kempke, S.; Luyten, P.; Van Houdenhove, B.; Goossens, L.; Bekaert, P.; Van Wambeke, P. Self-esteem mediates the relationship between maladaptive perfectionism and depression in chronic fatigue syndrome. *Clin. Rheumatol.* **2011**, *30*, 1543–1548.
1048. Kempke, S.; Van Den Eede, F.; Schotte, C.; Claes, S.; Van Wambeke, P.; Van Houdenhove, B.; Luyten, P. Prevalence of DSM-IV personality disorders in patients with chronic fatigue syndrome: a controlled study. *Int. J. Behav. Med.* **2013**, *20*, 219–228.
1049. Kennedy, G.; Khan, F.; Hill, A.; Underwood, C.; Belch, J.J.F. Biochemical and vascular aspects of pediatric chronic fatigue syndrome. *Arch. Pediatr. Adolesc. Med.* **2010**, *164*, 817–823.
1050. Kenney, D.; Jenkins, S.; Youssef, P.; Kotagal, S. Patient Use of Complementary and Alternative Medicines in an Outpatient Pediatric Neurology Clinic. *Pediatr. Neurol.* **2016**, *58*, 48–52.e7.

1051. Kenny, R.A.; Graham, L.A. Chronic fatigue syndrome symptoms common in patients with vasovagal syncope [4]. *Am. J. Med.* **2001**, *110*, 242–243.
1052. Kenny, R.A.; Graham, L.A. Chronic fatigue syndrome symptoms common in patient with vasovagal syncope. *Am. J. Med.* 2001, *110*, 242–243.
1053. Kenter, E.G.H.; Okkes, I.M. Patients with fatigue in general practice; prevalence and treatment [Prevalentie en behandeling van vermoeide patienten in de huisartspraktijk; gegevens uit het transitieproject]. *Ned. Tijdschr. Geneeskd.* **1999**, *143*, 796–800. Work in Dutch
1054. Kenter, E.G.; Okkes, I.M. [Patients with fatigue in family practice: prevalence and treatment]. *Ned. Tijdschr. Geneeskd.* **1999**, *143*, 796–801. Work in Dutch
1055. Kenyon, J.C.; Lever, A.M.L. XMRV, prostate cancer and chronic fatigue syndrome. *Br. Med. Bull.* **2011**, *98*, 61–74.
1056. Kerr, J.R.; Barrett, A.-M.; Curran, M.D.; Behan, W.M.H.; Middleton, D.; Behan, P.O. Parvovirus B19 and chronic fatigue syndrome. *J. Chronic Fatigue Syndr.* **1997**, *3*, 101–107.
1057. Kerr, J.R.; Bracewell, J.; Laing, I.; Mattey, D.L.; Bernstein, R.M.; Bruce, I.N.; Tyrrell, D.A.J. Chronic fatigue syndrome and arthraliga following parvovirus B19 infection. *J. Rheumatol.* **2002**, *29*, 595–602.
1058. Kerr, J.R.; Cunniffe, V.S. Antibodies to parvovirus B19 non-structural protein are associated with chronic but not acute arthritis following B19 infection. *Rheumatology* **2000**, *39*, 903–908.
1059. Kerr, J.R.; Curran, M.D.; Moore, J.E.; Murphy, P.G. Parvovirus b19 infection - persistence and genetic variation. *Scand. J. Infect. Dis.* **1995**, *27*, 551–557.
1060. Kerr, J.R.; Umene, K. The molecular epidemiology of parvovirus B19. *Rev. Med. Microbiol.* **1997**, *8*, 21–31.
1061. Kerr, J.R.; Bracewell, J.; Laing, I.; Mattey, D.L.; Bernstein, R.M.; Bruce, I.N.; Tyrrell, D.A.J. Chronic fatigue syndrome and arthralgia following parvovirus B19 infection. *J. Rheumatol.* **2002**, *29*, 595–602.
1062. Keshavarz, M.; Niya, M.H.K.; Tameshkel, F.S.; Nejad, A.S.M.; Monavari, S.H.; Keyvani, H. A survey on human T-cell lymphotropic virus type 1 (HTLV-1) and xenotropic murine leukemia virus-related virus (XMRV) coinfection in Tehran, Iran. *J. Pharm. Bioallied Sci.* **2018**, *10*, 166–171.
1063. Kessler, R.; White, L.A.; Birnbaum, H.; Qiu, Y.; Kidolezi, Y.; Mallett, D.; Swindle, R. Comparative and interactive effects of depression relative to other health problems on work performance in the workforce of a large employer. *J. Occup. Environ. Med.* **2008**, *50*, 809–816.
1064. Khoo, T.; Proudman, S.; Limaye, V. Silicone breast implants and depression, fibromyalgia and chronic fatigue syndrome in a rheumatology clinic population. *Clin. Rheumatol.* **2019**, *38*, 1271–1276.
1065. Kiesswetter, E.; Sietmann, B.; Zupanic, M.; van Thriel, C.; Golka, K.; Seeber, A. Neurobehavioral aspects of the prevalence and etiology of ``multiple chemical sensitivity{’’}. *ALLERGOLOGIE* **1999**, *22*, 719–735.
1066. Kim, C.H.; Shin, H.C.; Won, C.W.; Cheol, H.K.; Ho, C.S.; Chang, W.W. Prevalence of chronic fatigue and chronic fatigue syndrome in Korea: Community-based primary care study. *J. Korean Med. Sci.* **2005**, *20*, 529–534.
1067. Kim, H.S.; Zuckerbraun, E.; Daigle, K.; Lee, M.L.; Friedman, T.C. High prevalence of renin-aldosterone axis abnormalities in patients with chronic fatigue syndrome. *J. Investig. Med.* **2004**, *52*, S98.
1068. Kim, J.; Ku, B.; Kim, K.H. Validation of the qi blood yin yang deficiency questionnaire on chronic fatigue. *Chinese Med. (United Kingdom)* **2016**, *11*.

1069. Kim, J.K.S.; Zhu, Z.; Casale, G.; Koutakis, P.; McComb, R.D.; Swanson, S.; Thompson, J.; Miserlis, D.; Johanning, J.M.; Haynatzki, G.; et al. Human Enterovirus in the Gastrocnemius of Patients With Peripheral Arterial Disease. *J. Am. Heart Assoc.* **2013**, *2*.
1070. Kim, S.-H. Prevalence of chronic widespread pain and chronic fatigue syndrome in young korean adults. *J. Musculoskelet. Pain* **2008**, *16*, 149–153.
1071. Kim, S.-H.; Lee, K.; Lim, H.-S. Prevalence of chronic widespread pain and chronic fatigue syndrome in Korean livestock raisers. *J. Occup. Health* **2008**, *50*, 525–528.
1072. Kimmel, S.R.; Burns, I.T.; Wolfe, R.M.; Zimmerman, R.K. Addressing immunization barriers, benefits, and risks. *J. Fam. Pract.* **2007**, *56*, S61–S69.
1073. Kimmel, S.R.; Wolfe, R.M. Communicating the benefits and risks of vaccines. *J. Fam. Pract.* **2005**, *54*, S51–S57.
1074. Kindlon, T. Criteria used to define chronic fatigue syndrome questioned. *Psychosom. Med.* 2010, *72*, 506–509.
1075. Kingma, E.M.; Rosmalen, J.G.M.; White, P.D.; Stansfeld, S.A.; Clark, C. The prospective association between childhood cognitive ability and somatic symptoms and syndromes in adulthood: the 1958 British birth cohort. *J. Epidemiol. Community Health* **2013**, *67*, 1047–1053.
1076. Kipen Neuroscience research agenda options panel. *Toxicol. Ind. Health* **1994**, *10*, 645–652.
1077. Kipen, H.M.; Fiedler, N. A 37-year-old mechanic with multiple chemical sensitivities. *Environ. Health Perspect.* **2000**, *108*, 377–381.
1078. Kipen, H.M.; Fiedler, N. The role of environmental factors in medically unexplained symptoms and related syndromes: conference summary and recommendations. *Environ. Health Perspect.* **2002**, *110 Suppl 4*, 591–595.
1079. Kipen, H.M.; Fiedler, N. Environmental factors in medically unexplained symptoms and related syndromes: the evidence and the challenge. *Environ. Health Perspect.* **2002**, *110 Suppl 4*, 597–599.
1080. Kipen, H.M.; Hallman, W.; Kang, H.; Fiedler, N.; Natelson, B.H. Prevalence of chronic fatigue and chemical sensitivities in Gulf Registry Veterans. *Arch. Environ. Health* **1999**, *54*, 313–318.
1081. Kir'iakov, V.A.; Saarkoppel', L.M.; Krylova, I. V; Sukhova, A. V [Chronic fatigue syndrome in patients with vibration disease]. *Med. Tr. Prom. Ekol.* **2013**, 28–32. Work in Russian
1082. Kitai, E.; Blumberg, G.; Golan-Cohen, A.; Levi, D.; Vinker, S. Seasonality of fatigue among young adults in the primary care setting. *Public Health* **2015**, *129*, 591–593.
1083. Kivimaki, M.; Leino-Arjas, P.; Virtanen, M.; Elovainio, M.; Keltikangas-Jarvine, L.; Puttonen, S.; Vartia, M.; Brunner, E.; Vahtera, J. Work stress and incidence of newly diagnosed fibromyalgia prospective cohort study. *J. Psychosom. Res.* **2004**, *57*, 417–422.
1084. Klein, R.; Berg, P.A. High incidence of antibodies to 5-hydroxytryptamine, gangliosides and phospholipids in patients with chronic fatigue and fibromyalgia syndrome and their relatives: evidence for a clinical entity of both disorders. *Eur. J. Med. Res.* **1995**, *1*, 21–26.
1085. Klineberg, I.; McGregor, N.; Butt, H.; Dunstan, H.; Roberts, T.; Zerbes, M. Chronic orofacial muscle pain: a new approach to diagnosis and management. *Alpha Omegan* **1998**, *91*, 25–28.
1086. Klonoff, D.C. Chronic fatigue syndrome. *Clin. Infect. Dis.* **1992**, *15*, 812–823.

1087. Kluger, B.M.; Krupp, L.B.; Enoka, R.M. Fatigue and fatigability in neurologic illnesses: Proposal for a unified taxonomy. *Neurology* **2013**, *80*, 409–416.
1088. Kminek, A.; Simunek, I.; Sadeh, A.; Carskadon, M.A.; Acebo, C.; Dahl, R.; Van Aerde, J. Chronic fatigue immune dysfunction syndrome: an epidemic? *Pediatrics* 1992, *89*, 804.
1089. Knight, S.; Elders, S.; Rodda, J.; Harvey, A.; Lubitz, L.; Rowe, K.; Reveley, C.; Hennel, S.; Towns, S.; Kozlowska, K.; et al. Epidemiology of paediatric chronic fatigue syndrome in Australia. *Arch. Dis. Child.* **2019**.
1090. Knight, S.J.; Politis, J.; Garnham, C.; Scheinberg, A.; Tollit, M.A. School Functioning in Adolescents With Chronic Fatigue Syndrome. *Front. Pediatr.* **2018**, *6*.
1091. Knight, S.; Harvey, A.; Lubitz, L.; Rowe, K.; Reveley, C.; Veit, F.; Hennel, S.; Scheinberg, A. Paediatric chronic fatigue syndrome: complex presentations and protracted time to diagnosis. *J. Paediatr. Child Health* **2013**, *49*, 919–924.
1092. Knight, S.; Harvey, A.; Towns, S.; Payne, D.; Lubitz, L.; Rowe, K.; Reveley, C.; Hennel, S.; Hiscock, H.; Scheinberg, A. How is paediatric chronic fatigue syndrome/myalgic encephalomyelitis diagnosed and managed by paediatricians? An Australian Paediatric Research Network Study. *J. Paediatr. Child Health* **2014**, *50*, 1000–1007.
1093. Knippenberg, S.; Bol, Y.; Damoiseaux, J.; Hupperts, R.; Smolders, J. Vitamin D status in patients with MS is negatively correlated with depression, but not with fatigue. *Acta Neurol. Scand.* **2011**, *124*, 171–175.
1094. Knudsen, A.K.; Henderson, M.; Harvey, S.B.; Chalder, T. Long-term sickness absence among patients with chronic fatigue syndrome. *Br. J. Psychiatry* **2011**, *199*, 430–431.
1095. Koch, L.; Hennessey, M. Emerging disabilities. *Work* **2008**, *31*, 273–275.
1096. Kodama, M.; Kodama, T.; Murakami, M. The value of the dehydroepiandrosterone-annexed vitamin C infusion treatment in the clinical control of chronic fatigue syndrome (CFS). II. Characterization of CFS patients with special reference to their response to a new vitamin C infusion treatment. *In Vivo* **1996**, *10*, 585–596.
1097. Kodama, M.; Kodama, M. The Autoimmune Disease Complex Interstitial Pneumonia/Dermatomyositis in the Light of Endocrinology and Cancer Epidemiology. *In Vivo (Brooklyn).* **2009**, *23*, 353–356.
1098. Koedel, U.; Fingerle, V.; Pfister, H.-W. Lyme neuroborreliosis-epidemiology, diagnosis and management. *Nat. Rev. Neurol.* **2015**, *11*, 446–456.
1099. Koedel, U.; Pfister, H.-W. Lyme neuroborreliosis. *Curr. Opin. Infect. Dis.* **2017**, *30*, 101–107.
1100. Koelle, D.M.; Barcy, S.; Huang, M.-L.; Ashley, R.L.; Corey, L.; Zeh, J.; Ashton, S.; Buchwald, D. Markers of viral infection in monozygotic twins discordant for chronic fatigue syndrome. *Clin. Infect. Dis.* **2002**, *35*, 518–525.
1101. Koh, J.H.; Kwok, S.-K.; Lee, J.; Park, S.-H. Autonomic dysfunction in primary Sjogren's syndrome: a prospective cohort analysis of 154 Korean patients. *KOREAN J. Intern. Med.* **2017**, *32*, 165+.
1102. Kollmann, J.; Gollwitzer, M.; Spada, M.M.; Fernie, B.A. The association between metacognitions and the impact of Fibromyalgia in a German sample. *J. Psychosom. Res.* **2016**, *83*, 1–9.
1103. Komaroff, A.L.; Delbanco A 56-year-old woman with chronic fatigue syndrome. *JAMA-JOURNAL Am. Med. Assoc.* **1997**, *278*, 1179–1185.

1104. Komaroff, A.L.; Fagioli, L.R.; Doolittle, T.H.; Gandek, B.; Gleit, M.A.; Guerriero, R.T.; Kornish, J.; Ware, N.C.; Ware, J.E.; Bates, D.W. Health status in patients with chronic fatigue syndrome and in general population and disease comparison groups. *Am. J. Med.* **1996**, *101*, 281–290.
1105. Komaroff, A.L.; Klimas, N. Chronic fatigue syndrome: What have we learned and what do we need to know? *Clin. Infect. Dis.* **1994**, *18*, S166–S167.
1106. Komaroff, A.L. Myalgic Encephalomyelitis/Chronic Fatigue Syndrome: A Real Illness. *Ann. Intern. Med.* 2015, *162*, 871–872.
1107. Koo, D. Chronic fatigue syndrome. A critical appraisal of the role of Epstein-Barr virus. *West. J. Med.* **1989**, *150*, 590–596.
1108. Kop, W.J.; Kupper, H.M. *Fatigue and stress*; 2016;
1109. Korszun, A.; Papadopoulos, E.; Demitrack, M.; Engleberg, C.; Crofford, L. The relationship between temporomandibular disorders and stress-associated syndromes. *ORAL Surg. ORAL Med. ORAL Pathol. ORAL Radiol. ENDODONTOLOGY* **1998**, *86*, 416–420.
1110. Koutoubi, S.; Cartmell, J.W.; Kestin, M.; Lecovin, G. Protein nutrition in fibromyalgia. *J. Chronic Fatigue Syndr.* **2008**, *14*, 47–58.
1111. Kovacic, K.; Chelimsky, T.C.; Sood, M.R.; Simpson, P.; Nugent, M.; Chelimsky, G. Joint Hypermobility: A Common Association with Complex Functional Gastrointestinal Disorders. *J. Pediatr.* **2014**, *165*, 973–978.
1112. Kovaleva, A.I.; Pyshnov, G.I. [Problems of chronic fatigue]. *Med. Tr. Prom. Ekol.* **2001**, 1–5. Work in Russian
1113. Kowal, K.; Schacterele, R.S.; Schur, P.H.; Komaroff, A.L.; DuBuske, L.M. Prevalence of allergen-specific IgE among patients with chronic fatigue syndrome. *Allergy asthma Proc.* **2002**, *23*, 35–39.
1114. Kozireva, S. V; Zestkova, J. V; Mikazane, H.J.; Kadisa, A.L.; Kakurina, N.A.; Lejnieks, A.A.; Danilane, I.N.; Murovska, M.F. Incidence and clinical significance of parvovirus B19 infection in patients with rheumatoid arthritis. *J. Rheumatol.* **2008**, *35*, 1265–1270.
1115. Kranzler, H.R.; Manu, P.; Hesselbrock, V.M.; Lane, T.J.; Matthews, D.A. Substance use disorders in patients with chronic fatigue. *Hosp. Community Psychiatry* **1991**, *42*, 924–928.
1116. Kravitz, H.M.; Katz, R.S. Fibrofog and fibromyalgia: a narrative review and implications for clinical practice. *Rheumatol. Int.* **2015**, *35*, 1115–1125.
1117. Kreijne, J.E.; Lie, M.R.K.L.; Vogelaar, L.; van der Woude, C.J. Practical Guideline for Fatigue Management in Inflammatory Bowel Disease. *J. Crohns. Colitis* **2016**, *10*, 105–111.
1118. Krieger, J.N.; Stephens, A.J.; Landis, J.R.; Clemens, J.Q.; Kreder, K.; Lai, H.H.; Afari, N.; Rodriguez, L.; Schaeffer, A.; Mackey, S.; et al. Relationship between Chronic Nonurological Associated Somatic Syndromes and Symptom Severity in Urological Chronic Pelvic Pain Syndromes: Baseline Evaluation of the MAPP Study. *J. Urol.* **2015**, *193*, 1254–1262.
1119. Krilov, L.R.; Fisher, M.; Friedman, S.B.; Reitman, D.; Mandel, F.S. Course and outcome of chronic fatigue in children and adolescents. *Pediatrics* **1998**, *102*, 360–366.
1120. Krueger, G.R.F.; Ablashi, D. V Human herpesvirus-6: A short review of its biological behavior. *Intervirology* **2003**, *46*, 257–269.
1121. Krueger, G.R.F.; Sander, O. What's New in Human Herpesvirus-6? Clinical Immunopathology of the HHV-6 Infection. *Pathol. Res. Pract.* **1989**, *185*, 915–929.

1122. Krueger, G.R.; Klueppelberg, U.; Hoffmann, A.; Ablashi, D. V Clinical correlates of infection with human herpesvirus-6. *In Vivo* **1994**, *8*, 457–485.
1123. Kruesi, M.J.P.; Dale, J.; Straus, S.E. Psychiatric diagnoses in patients who have chronic fatigue syndrome. *J. Clin. Psychiatry* **1989**, *50,* 53–56.
1124. KRUPP, L.B.; MENDELSON, W.B.; FRIEDMAN, R. AN OVERIVEW OF CHRONIC FATIGUE SYNDROME. *J. Clin. Psychiatry* **1991**, *52*, 403–410.
1125. Krupp, L.B.; Mendelson, W.B.; Friedman, R. An overview of chronic fatigue syndrome. *J. Clin. Psychiatry* **1991**, *52*, 403–410.
1126. Krupp, L.B.; Pollina, D. Neuroimmune and neuropsychiatric aspects of chronic fatigue syndrome. *Adv. Neuroimmunol.* **1996**, *6*, 155–167.
1127. KRUPP, L.B.; SLIWINSKI, M.; MASUR, D.M.; FRIEDBERG, F.; COYLE, P.K. COGNITIVE-FUNCTIONING AND DEPRESSION IN PATIENTS WITH CHRONIC FATIGUE SYNDROME AND MULTIPLE-SCLEROSIS. *Arch. Neurol.* **1994**, *51*, 705–710.
1128. Krzeczkowska, A.; Karatzias, T.; Dickson, A. Pain in people with chronic fatigue syndrome/myalgic encephalomyelitis: The role of traumatic stress and coping strategies. *Psychol. Heal. Med.* **2015**, *20*, 210–216.
1129. Kubo, K.; Fujiyoshi, T.; Yokoyama, M.M.; Kamei, K.; Richt, J.A.; Kitze, B.; Herzog, S.; Takigawa, M.; Sonoda, S. Lack of association of Borna disease virus and human T-cell leukemia virus type 1 infections with psychiatric disorders among Japanese patients. *Clin. Diagn. Lab. Immunol.* **1997**, *4*, 189–194.
1130. Kuhnt, S.; Brähler, E. Tumorassociated fatigue [Tumorassoziierte Fatigue]. *PPmP Psychother. Psychosom. Medizinische Psychol.* **2010**, *60*, 402–411. Work in German
1131. Kumar, A.; Clark, S.; Boudreaux, E.D.; Camargo Jr., C.A. A multicenter study of depression among emergency department patients. *Acad. Emerg. Med.* **2004**, *11*, 1284–1289.
1132. Kung, W.W.; Lu, P.-C. How symptom manifestations affect help seeking for mental health problems among Chinese Americans. *J. Nerv. Ment. Dis.* **2008**, *196*, 46–54.
1133. Kuratsune, H.; Watanabe, Y. *Chronic fatigue syndrome*; 2008;
1134. Kuratsune, H.; Yamaguti, K.; Lindh, G.; Evengard, B.; Takahashi, M.; Machii, T.; Matsumura, K.; Takaishi, J.; Kawata, S.; Langstrom, B.; et al. Low levels of serum acylcarnitine in chronic fatigue syndrome and chronic hepatitis type C, but not seen in other diseases. *Int. J. Mol. Med.* **1998**, *2*, 51–56.
1135. Kuratsune, H. [Overview of chronic fatigue syndrome focusing on prevalence and diagnostic criteria]. *Nihon Rinsho.* **2007**, *65*, 983–990. Work in Japanese
1136. Kyrdalen, A.E.; Dahl, A.A.; Hernes, E.; Cvancarova, M.; Fosså, S.D. Fatigue in hormone-naïve prostate cancer patients treated with radical prostatectomy or definitive radiotherapy. *Prostate Cancer Prostatic Dis.* **2010**, *13*, 144–150.
1137. Kyrdalen, A.E.; Dahl, A.A.; Hernes, E.; Hem, E.; Fosså, S.D. Fatigue in prostate cancer survivors treated with definitive radiotherapy and LHRH analogs. *Prostate* **2010**, *70*, 1480–1489.
1138. Łabuz-Roszak, B.; Pierzhała, K.; Kumor, K.; Wyrozumska, K.; Baran, A.; Werner, A.; Bak, E.; Jaźwiecka, M. Evaluation of frequency of fatigue syndrome in patients with multiple sclerosis [Ocena czestości wystepowania zespołu zmeczenia u chorych na stwardnienie rozsiane.]. *Wiad. Lek.* **2009**, *62*, 99–103. Work in Polish
1139. Labuz-Roszak, B.; Pierzhala, K.; Kumor, K.; Wyrozumska, K.; Baran, A.; Werner, A.; Bak, E.; Jazwiecka, M. [Evaluation of frequency of fatigue syndrome in patients with multiple sclerosis]. *Wiad. Lek.* **2009**, *62*, 99–103. Work in Polish

1140. Lacerda, E.M.; McDermott, C.; Kingdon, C.C.; Butterworth, J.; Cliff, J.M.; Nacul, L. Hope, disappointment and perseverance: Reflections of people with Myalgic encephalomyelitis/Chronic Fatigue Syndrome (ME/CFS) and Multiple Sclerosis participating in biomedical research. A qualitative focus group study. *Heal. Expect.* **2019**, *22*, 373–384.
1141. Lacour, M.; Zunder, T.; Dettenkofer, M.; Schonbeck, S.; Ludtke, R.; Scheidt, C. An interdisciplinary therapeutic approach for dealing with patients attributing chronic fatigue and functional memory disorders to environmental poisoning - a pilot study. *Int. J. Hyg. Environ. Health* **2002**, *204*, 339–346.
1142. Lacour, M.; Zunder, T.; Huber, R.; Sander, A.; Daschner, F.; Frank, U. The pathogenetic significance of intestinal Candida colonization - A systematic review from an interdisciplinary and environmental medical point of view. *Int. J. Hyg. Environ. Health* **2002**, *205*, 257–268.
1143. Lacourt, T.E.; Houtveen, J.H.; Smeets, H.M.; Lipovsky, M.M.; van Doornen, L.J.P. Infection Load as a Predisposing Factor for Somatoform Disorders: Evidence From a Dutch General Practice Registry. *Psychosom. Med.* **2013**, *75*, 759–764.
1144. Lago Blanco, E.; Puiguriguer Ferrando, J.; Rodríguez Enríquez, M.; Agüero Gento, L.; Salvà Coll, J.; Pizà Portell, M.R. Multiple chemical sensitivity: Clinical evaluation of the severity and psychopathological profile [Sensibilidad química múltiple: evaluación clínica de la gravedad y perfil psicopatológico]. *Med. Clin. (Barc).* **2016**, *146*, 108–111. Work in Spanish
1145. Lago Blanco, E.; Puiguriguer Ferrando, J.; Rodriguez Enriquez, M.; Aguero Gento, L.; Salva Coll, J.; Piza Portell, M.R. [Multiple chemical sensitivity: Clinical evaluation of the severity and psychopathological profile]. *Med. Clin. (Barc).* **2016**, *146*, 108–111. Work in Spanish
1146. Lai, H.H.; Krieger, J.N.; Pontari, M.A.; Buchwald, D.; Hou, X.; Landis, J.R.; Network, M.R. Painful Bladder Filling and Painful Urgency are Distinct Characteristics in Men and Women with Urological Chronic Pelvic Pain Syndromes: A MAPP Research Network Study. *J. Urol.* **2015**, *194*, 1634–1641.
1147. LaManca, J.J.; Peckerman, A.; Walker, J.; Kesil, W.; Cook, S.; Taylor, A.; Natelson, B.H. Cardiovascular response during head-up tilt in chronic fatigue syndrome. *Clin. Physiol.* **1999**, *19*, 111–120.
1148. Lamarque, D. Why looking for martial deficiency in patients with inflammatory bowel disease? [Pourquoi chercher la carence martiale chez les patients ayant une maladie inflammatoire intestinale ?]. *Hepato-Gastro Oncol. Dig.* **2017**, *24*, 1077–1081. Work in French
1149. Landis, C.A.; Lentz, M.J.; Rothermel, J.; Buchwald, D.; Shaver, J.L.F. Decreased sleep spindles and spindle activity in midlife women with fibromyalgia and pain. *Sleep* **2004**, *27*, 741–750.
1150. Landis, J.R.; Williams, D.A.; Lucia, M.S.; Clauw, D.J.; Naliboff, B.D.; Robinson, N.A.; Van Bokhoven, A.; Sutcliffe, S.; Schaeffer, A.J.; Rodriguez, L. V; et al. The MAPP research network: Design, patient characterization and operations. *BMC Urol.* **2014**, *14*.
1151. Landmark-Høyvik, H.; Reinertsen, K. V; Loge, J.H.; Kristensen, V.N.; Dumeaux, V.; Fosså, S.D.; Børresen-Dale, A.-L.; Edvardsen, H. The Genetics and Epigenetics of Fatigue. *PM R* **2010**, *2*, 456–465.
1152. Landmark-Hoyvik, H.; Reinertsen, K. V; Loge, J.H.; Kristensen, V.N.; Dumeaux, V.; Fossa, S.D.; Borresen-Dale, A.-L.; Edvardsen, H. The Genetics and Epigenetics of Fatigue. *PM&R* **2010**, *2*, 456–465.

1153. Lane, T.J.; Manu, P.; Matthews, D.A. Depression and somatization in the chronic fatigue syndrome. *Am. J. Med.* **1991**, *91*, 335–344.
1154. Langenberg, P.W.; Wallach, E.E.; Clauw, D.J.; Howard, F.M.; Diggs, C.M.; Wesselmann, U.; Greenberg, P.; Warren, J.W. Pelvic pain and surgeries in women before interstitial cystitis/painful bladder syndrome. *Am. J. Obstet. Gynecol.* **2010**, *202*, 286.e1-6.
1155. Langsjoen, P.H.; Folkers, K. Isolated diastolic dysfunction of the myocardium and its response to CoQ10 treatment. *Clin. Investig.* **1993**, *71*, S140–S144.
1156. Larkin, D.; Martin, C.R. The interface between chronic fatigue syndrome and depression: A psychobiological and neurophysiological conundrum [L'interface entre le syndrome de fatigue chronique et la dépression : un enchevêtrement psychobiologique et neurophysiologique]. *Neurophysiol. Clin.* **2017**, *47*, 123–129. Work in French
1157. Lau, C.; Lin, C.-C.; Chen, W.-H.; Wang, H.-C.; Kao, C.-H. Increased risk of chronic fatigue syndrome in patients with migraine: A retrospective cohort study. *J. Psychosom. Res.* **2015**, *79*, 514–518.
1158. Laufer, N.; Zilber, N.; Jecsmien, P.; Maoz, B.; Grupper, D.; Hermesh, H.; Gilad, R.; Weizman, A.; Munitz, H. Mental disorders in primary care in Israel: prevalence and risk factors. *Soc. Psychiatry Psychiatr. Epidemiol.* **2013**, *48*, 1539–1554.
1159. Laugharne, R.; Flynn, A. Personality disorders in consultation-liaison psychiatry. *Curr. Opin. Psychiatry* **2013**, *26*, 84–89.
1160. Laukkanen, T.; Laukkanen, J.A.; Kunutsor, S.K. Sauna Bathing and Risk of Psychotic Disorders: A Prospective Cohort Study. *Med. Princ. Pract.* **2018**, *27*, 562–569.
1161. Lavie, P. On sleepy humans and sleepy rats. *J. Sleep Res.* 2008, *17*, 363–364.
1162. Lawrie, S.M.; Manders, D.N.; Geddes, J.R.; Pelosi, A.J. A population-based incidence study of chronic fatigue. *Psychol. Med.* **1997**, *27*, 343–353.
1163. Lawrie, S.M.; Pelosi, A.J. Chronic fatigue syndrome and myaligic encephalomyelitis. *BMJ* **1994**, *309*, 275.
1164. Lawrie, S.M.; Pelosi, A.J. Chronic fatigue syndrome in the community. Prevalence and associations. *Br. J. Psychiatry* **1995**, *166*, 793–797.
1165. Lawrie, S.M.; Pelosi, A.J. Chronic fatigue syndrome: prevalence and outcome. *BMJ* 1994, *308*, 732–733.
1166. Le Blackadder-Coward, J.C.Q.; Perry, S. Chronic fatigue syndrome in the UK armed forces. *J. R. Nav. Med. Serv.* **2013**, *99*, 121–126.
1167. Le Bon, O. Chronic fatigue syndrome [Le syndrome de fatigue chronique]. *Rev. Med. Brux.* **2006**, *27*, S 115-S 117. Work in French
1168. Le Bon, O. [Chronic fatigue syndrome]. *Rev. Med. Brux.* **2006**, *27*, S115-7. Work in French
1169. Le Bon, O.; Fischler, B.; Hoffmann, G.; Murphy, J.R.; De Meirleir, K.; Cluydts, R.; Pelc, I. How significant are primary sleep disorders and sleepiness in the chronic fatigue syndrome? *Sleep Res. Online* **2000**, *3*, 43–48.
1170. Le Bon, O.; Minner, P.; Van Moorsel, C.; Hoffmann, G.; Gallego, S.; Lambrecht, L.; Pelc, I.; Linkowski, P. First-night effect in the chronic fatigue syndrome. *Psychiatry Res.* **2003**, *120*, 191–199.
1171. Leask, J.; Chapman, S.; Cooper Robbins, S.C. "All manner of ills": The features of serious diseases attributed to vaccination. *Vaccine* **2010**, *28*, 3066–3070.
1172. Lee, A.K.Y.; Miller, W.C.; Townson, A.F.; Anton, H.A. Medication use is associated with fatigue in a sample of community-living individuals who have a spinal cord injury: a chart review. *Spinal Cord* **2010**, *48*, 429–433.

1173. Lee, J.-H.; Kim, S.-K.; Ko, S.-J.; Lee, S.-H.; Lee, J.-H.; Kim, M.-J.; Han, G.; Kim, J.; Chung, S.-Y.; Lee, B.-J.; et al. The Effect of Oriental Medicine Music Therapy on Idiopathic Chronic Fatigue. *J. Altern. Complement. Med.* **2015**, *21*, 422–429.
1174. Lee, R.; Rodin, G.; Devins, G.; Weiss, M.G. Illness experience, meaning and help-seeking among Chinese immigrants in Canada with chronic fatigue and weakness. *Anthropol. Med.* **2001**, *8*, 89–107.
1175. LEE, S.; WONG, K.C. RETHINKING NEURASTHENIA - THE ILLNESS CONCEPTS OF SHENJING SHUAIRUO AMONG CHINESE UNDERGRADUATES IN HONG-KONG. *Cult. Med. PSYCHIATRY* **1995**, *19*, 91–111.
1176. Lee, S.; Yu, H.; Wing, Y.; Chan, C.; Lee, A.M.; Lee, D.T.; Chen, C.; Lin, K.; Weiss, M.G. Psychiatric morbidity and illness experience of primary care patients with chronic fatigue in Hong Kong. *Am. J. Psychiatry* **2000**, *157*, 380–384.
1177. Lee, S.W.; Bae, G.Y.; Rim, H.-D.; Lee, S.J.; Chang, S.M.; Kim, B.-S.; Won, S. Mediating Effect of Resilience on the Association between Emotional Neglect and Depressive Symptoms. *PSYCHIATRY Investig.* **2018**, *15*, 62–69.
1178. Lehman, A.M.; Lehman, D.R.; Hemphill, K.J.; Mandel, D.R.; Cooper, L.M. Illness experience, depression, and anxiety in chronic fatigue syndrome. *J. Psychosom. Res.* **2002**, *52*, 461–465.
1179. Lehman, R. Flora medica. *Br. J. Gen. Pract.* **2005**, *55*, 61.
1180. Leiblum, S.; Seehuus, M.; Goldmeier, D.; Brown, C. Psychological, medical, and pharmacological correlates of persistent genital arousal disorder. *J. Sex. Med.* **2007**, *4*, 1358–1366.
1181. Leiblum, S.; Seehuus, M.; Goldrneier, D.; Brown, C. Psychological, medicalq and pharmacological correlates of persistent genital arousal disorder. *J. Sex. Med.* **2007**, *4*, 1358–1366.
1182. Leiknes, K.A.; Finset, A.; Moum, T.; Sandanger, I. Current somatoform disorders in Norway: Prevalence, risk factors and comorbidity with anxiety, depression and musculoskeletal disorders. *Soc. Psychiatry Psychiatr. Epidemiol.* **2007**, *42*, 698–710.
1183. LEITCH, A.G. NEURASTHENIA, MYALGIC ENCEPHALITIS OR CRYPTOGENIC CHRONIC FATIGUE SYNDROME. *QJM-MONTHLY J. Assoc. PHYSICIANS* **1995**, *88*, 447–450.
1184. Leite, J.C. de C.; Drachler, M. de L.; Killett, A.; Kale, S.; Nacul, L.; McArthur, M.; Hong, C.S.; O'Driscoll, L.; Pheby, D.; Campion, P.; et al. Social support needs for equity in health and social care: a thematic analysis of experiences of people with chronic fatigue syndrome/myalgic encephalomyelitis. *Int. J. Equity Health* **2011**, *10*.
1185. Leone, S.S.; Wessely, S.; Huibers, M.J.H.; Knottnerus, J.A.; Kant, Ij. Two sides of the same coin? On the history and phenomenology of chronic fatigue and burnout. *Psychol. Health* **2011**, *26*, 449–464.
1186. Leppavuori, A. [Chronic fatigue syndrome]. *Duodecim.* **2006**, *122*, 545–553. Work in Finnish
1187. Lerdal, A.; Celius, E.G.; Krupp, L.; Dahl, A.A. A prospective study of patterns of fatigue in multiple sclerosis. *Eur. J. Neurol.* **2007**, *14*, 1338–1343.
1188. Lerner, A.M.; Dworkin, H.J.; Sayyed, T.; Chang, C.H.; Fitzgerald, J.T.; Beqaj, S.; Deeter, R.G.; Goldstein, J.; Gottipolu, P.; O'Neill, W. Prevalence of abnormal cardiac wall motion in the cardiomyopathy associated with incomplete multiplication of Epstein-Barr virus and/or cytomegalovirus in patients with chronic fatigue syndrome. *In Vivo (Brooklyn).* **2004**, *18*, 417–424. Proceedings of

the 40th Interscience Conference on Antimicrobial Agents and Chemotherapy in Toronto, Canada.
1189. Lerner, A.M.; Goldstein, J.; Chang, C.-H.; Zervos, M.; Fitzgerald, J.T.; Dworkin, H.J.; Lawrie-hoppen, C.; Korotkin, S.M.; Brodsky, M.; O'Neill, W. Cardiac involvement in patients with chronic fatigue syndrome as documented with holter and biopsy data in Birmingham, Michigan, 1991-1993. *Infect. Dis. Clin. Pract.* **1997**, *6*, 327–333.
1190. Lerner, A.M.; Lawrie, C.; Dworkin, H.S. Repetitively negative changing T waves at 24-h electrocardiographic monitors in patients with the chronic fatigue syndrome. Left ventricular dysfunction in a cohort. *Chest* **1993**, *104*, 1417–1421.
1191. Levin, A.M. Chronic fatigue syndrome: The yeast concept. *J. Chronic Fatigue Syndr.* **2001**, *8*, 71–76.
1192. Levin, J.; Steele, L. On the epidemiology of "mysterious" phenomena. *Altern. Ther. Health Med.* **2001**, *7*, 64–66.
1193. Levine, P.H. The history and epidemiology of chronic fatigue syndrome (CFS). *EOS Riv. di Immunol. ed Immunofarmacol.* **1995**, *15*, 4–7.
1194. Levine, P.H. Summary and perspective: Epidemiology of chronic fatigue syndrome. *Clin. Infect. Dis.* **1994**, *18*, S57–S60.
1195. Levine, P.H. Chronic fatigue syndrome comes of age. *Am. J. Med.* **1998**, *105*, 2S–6S.
1196. Levine, P.H. Epidemiologic advances in chronic fatigue syndrome. *J. Psychiatr. Res.* **1997**, *31*, 7–18. Proceedings of the 1st Research and Clinical Conference of the American-Association-for-Chronic-Fatigue-Syndrome in FT Lauderdale, USA.
1197. Levine, P.H. Epidemiology of chronic fatigue syndrome. *Clin. Infect. Dis.* **1994**, *18 Suppl 1*, S57-60.
1198. Levine, P.H. Epidemic neuromyasthenia and chronic fatigue syndrome: epidemiological importance of a cluster definition. *Clin. Infect. Dis.* **1994**, *18 Suppl 1*, S16-20.
1199. Levine, P.H.; Atherton, M.; Fears, T.; Hoover, R. An approach to studies of cancer subsequent to clusters of chronic fatigue syndrome: use of data from the Nevada State Cancer Registry. *Clin. Infect. Dis.* **1994**, *18 Suppl 1*, S49-53.
1200. Levine, P.H.; Fears, T.R.; Cummings, P.; Hoover, R.N. Cancer and a fatiguing illness in Northern Nevada--a causal hypothesis. *Ann. Epidemiol.* **1998**, *8*, 245–249.
1201. Levine, P.H.; Jacobson, S.; Pocinki, A.G.; Cheney, P.; Peterson, D.; Connelly, R.R.; Weil, R.; Robinson, S.M.; Ablashi, D. V; Salahuddin, S.Z. Clinical, epidemiologic, and virologic studies in four clusters of the chronic fatigue syndrome. *Arch. Intern. Med.* **1992**, *152*, 1611–1616.
1202. Levine, P.H.; Peterson, D.; McNamee, F.L.; O'Brien, K.; Gridley, G.; Hagerty, M.; Brady, J.; Fears, T.; Atherton, M.; Hoover, R. Does chronic fatigue syndrome predispose to non-Hodgkin's lymphoma? *Cancer Res.* **1992**, *52*, 5516s–5518s; discussion 5518s–5521s.
1203. LEVINE, P.H.; PETERSON, D.; MCNAMEE, F.L.; OBRIEN, K.; GRIDLEY, G.; HAGERTY, M.; BRADY, J.; FEARS, T.; ATHERTON, M.; HOOVER, R. DOES CHRONIC FATIGUE SYNDROME PREDISPOSE TO NON-HODGKINS-LYMPHOMA. *CANCER Res.* **1992**, *52*, S5516–S5518.
1204. Levine, P.H.; Snow, P.G.; Ranum, B.A.; Paul, C.; Holmes, M.J. Epidemic neuromyasthenia and chronic fatigue syndrome in west Otago, New Zealand. A 10-year follow-up. *Arch. Intern. Med.* **1997**, *157*, 750–754.

1205. Levine, P.; Pilkington, D.; Peterson, D.L.; Pilkington, D. Chronic fatigue syndrome and cancer. *J. Chronic Fatigue Syndr.* **2000**, *7*, 29–38.
1206. Levine, S. Prevalence in the cerebrospinal fluid of the following infectious agents in a cohort of 12 CFS subjects: Human herpes virus-6 and 8; Chlamydia species; Mycoplasma species; EBV; CMV; and Coxsackievirus. *J. Chronic Fatigue Syndr.* **2001**, *9*, 41–51.
1207. Levine, S. Borna disease virus proteins in patients with CFS. *J. Chronic Fatigue Syndr.* **1999**, *5*, 199–206.
1208. Levine, S.; Eastman, H.; Ablashi, D. V Prevalence of IgM and IgG antibody to HHV-6 and HHV-8 and results of plasma PCR to HHV-6 and HHV-7 in a group of CFS patients and healthy donors. *J. Chronic Fatigue Syndr.* **2001**, *9*, 31–40.
1209. Lewis, G.; Wessely, S. The epidemiology of fatigue: more questions than answers. *J. Epidemiol. Community Health* **1992**, *46*, 92–97.
1210. Leza, J.C. Fibromyalgia: A challenge for neuroscience [Fibromialgia: Un reto también para la neurociencia]. *Rev. Neurol.* **2003**, *36*, 1165–1175. Work in Spanish
1211. Li, B.; Mahan, C.M.; Kang, H.K.; Eisen, S.A.; Engel, C.C. Longitudinal health study of us 1991 gulf war veterans: Changes in health status at 10-year follow-up. *Am. J. Epidemiol.* **2011**, *174*, 761–768.
1212. Li, H.; Wang, J.; Zhang, W.; Zhao, N.; Hai, X.; Sun, S.; Sun, Q.; Han, Y.; Zhang, R.; Ma, F. Chronic fatigue syndrome treated by the traditional Chinese procedure abdominal tuina: a randomized controlled clinical trial. *J. Tradit. Chinese Med.* **2017**, *37*, 819–826.
1213. Li, Y.J.; Wang, D.X.; Zhang, F.M.; Liu, Z.D.; Yang, A.Y.; Ykuta, K. Detection of antibody against Borna disease virus-p24 in the plasma of Chinese patients with chronic fatigue syndrome by Western-blot analysis. *Zhonghua Shi Yan He Lin Chuang Bing Du Xue Za Zhi* **2003**, *17*, 330–333.
1214. Li, Y.-J.; Wang, D.-X.; Bai, X.-L.; Chen, J.; Liu, Z.-D.; Feng, Z.-J.; Zhao, Y.-M. Clinical characteristics of patients with chronic fatigue syndrome: Analysis of 82 cases. *Natl. Med. J. China* **2005**, *85*, 701–704.
1215. Lian, O.S.; Bondevik, H. Medical constructions of long-term exhaustion, past and present. *Sociol. Health Illn.* **2015**, *37*, 920–935.
1216. Libbus, K.; Baker, J.L.; Osgood, J.M.; Phillips, T.C.; Valentine, D.M. Persistent fatigue in well women. *Women Health* **1995**, *23*, 57–72.
1217. Libman, E.; Creti, L.; Baltzan, M.; Rizzo, D.; Fichten, C.S.; Bailes, S. Sleep apnea and psychological functioning in chronic fatigue syndrome. *J. Health Psychol.* **2009**, *14*, 1251–1267.
1218. Lidbury, B.A.; Kita, B.; Lewis, D.P.; Hayward, S.; Ludlow, H.; Hedger, M.P.; de Kretser, D.M. Activin B is a novel biomarker for chronic fatigue syndrome/myalgic encephalomyelitis (CFS/ME) diagnosis: a cross sectional study. *J. Transl. Med.* **2017**, *15*, 60.
1219. Lieb, K.; Dammann, G.; Berger, M.; Bauer, J. Chronic fatigue syndrome: Definition, clinical diagnosis, and therapy. *NERVENARZT* **1996**, *67*, 711–720.
1220. Lieberman, J.; Bell, D.S. Serum angiotensin-converting enzyme as a marker for the chronic fatigue-immune dysfunction syndrome: A comparison to serum angiotensin-converting enzyme in sarcoidosis. *Am. J. Med.* **1993**, *95*, 407–412.
1221. Liedberg, G.M.; Burckhardt, C.S.; Henriksson, C.M. Young women with fibromyalgia in the United States and Sweden: Perceived difficulties during the first year after diagnosis. *Disabil. Rehabil.* **2006**, *28*, 1177–1184.

1222. Lievesley, K.; Rimes, K.A.; Chalder, T. A review of the predisposing, precipitating and perpetuating factors in Chronic Fatigue Syndrome in children and adolescents. *Clin. Psychol. Rev.* **2014**, *34*, 233–248.
1223. Lightfoot, R.W.J.; Luft, B.J.; Rahn, D.W.; Steere, A.C.; Sigal, L.H.; Zoschke, D.C.; Gardner, P.; Britton, M.C.; Kaufman, R.L. Empiric parenteral antibiotic treatment of patients with fibromyalgia and fatigue and a positive serologic result for Lyme disease. A cost-effectiveness analysis. *Ann. Intern. Med.* **1993**, *119*, 503–509.
1224. Lim, B.; Hortobagyi, G.N. Current challenges of metastatic breast cancer. *Cancer Metastasis Rev.* **2016**, *35*, 495–514.
1225. Lim, B.R.; Tan, S.-Y.; Zheng, Y.-P.; Lin, K.-M.; Park, B.C.; Turk, A.A. Psychosocial factors in chronic fatigue syndrome among Chinese Americans: a longitudinal community-based study. *Transcult. Psychiatry* **2003**, *40*, 429–441.
1226. Lin, C.-C.; Chen, M.-C.; Hsieh, H.-F.; Chang, S.-C. Illness Representations and Coping Processes of Taiwanese Patients With Early-Stage Chronic Kidney Disease. *J. Nurs. Res.* **2013**, *21*, 120–128.
1227. Lin, J.-M.S.; Brimmer, D.J.; Maloney, E.M.; Nyarko, E.; BeLue, R.; Reeves, W.C. Further validation of the Multidimensional Fatigue Inventory in a US adult population sample. *Popul. Health Metr.* **2009**, *7*.
1228. Lin, J.-M.S.; Brimmer, D.J.; Boneva, R.S.; Jones, J.F.; Reeves, W.C. Barriers to healthcare utilization in fatiguing illness: a population-based study in Georgia. *BMC Health Serv. Res.* **2009**, *9*, 13.
1229. Lindal, E.; Stefansson, J.G.; Bergmann, S. Letter to the editor. *Nord. J. Psychiatry* **2006**, *60*, 183.
1230. Lindal, E.; Stefansson, J.G.; Bergmann, S.; Líndal, E.; Stefánsson, J.G.; Bergmann, S. The prevalence of chronic fatigue syndrome in Iceland - A national comparison by gender drawing on four different criteria. *Nord. J. Psychiatry* **2002**, *56*, 273–277.
1231. Lindh, U.; Hudecek, R.; Danersund, A.; Eriksson, S.; Lindvall, A. Removal of dental amalgam and other metal alloys supported by antioxidant therapy alleviates symptoms and improves quality of life in patients with amalgam-associated ill health. *Neuro Endocrinol. Lett.* **2002**, *23*, 459–482.
1232. Lipkin, W.I.; Briese, T.; Hornig, M. Borna disease virus - Fact and fantasy. *Virus Res.* **2011**, *162*, 162–172.
1233. Lipkin, W.I.; Hornig, M.; Briese, T. Borna disease virus and neuropsychiatric disease - a reappraisal. *TRENDS Microbiol.* **2001**, *9*, 295–298.
1234. Litleskare, S.; Rortveit, G.; Eide, G.E.; Hanevik, K.; Langeland, N.; Wensaas, K.-A. Prevalence of Irritable Bowel Syndrome and Chronic Fatigue 10 Years After Giardia Infection. *Clin. Gastroenterol. Hepatol.* **2018**, *16*, 1064–1072.e4.
1235. Lizeth Luengas, L.; Carolina Tiga, D.; Mauricio Herrera, V.; Angel Villar-Centeno, L. Characterization of the health condition of people convalescing from a dengue episode. *BIOMEDICA* **2016**, *36*, 89–97.
1236. Lloyd, A.R. Chronic fatigue and chronic fatigue syndrome: shifting boundaries and attributions. *Am. J. Med.* **1998**, *105*, 7S–10S.
1237. Lloyd, A.R.; Hickie, I.; Boughton, C.R.; Spencer, O.; Wakefield, D. Prevalence of chronic fatigue syndrome in an Australian population. *Med. J. Aust.* **1990**, *153*, 522–528.
1238. Lloyd, A.R.; Pender, H. The economic impact of chronic fatigue syndrome. *Med. J. Aust.* **1992**, *157*, 599–601.

1239. LLOYD, A.; HICKIE, I.; HICKIE, C.; DWYER, J.; WAKEFIELD, D. Cell-mediated immunity in patients with chronic fatigue syndrome, healthy control subjects and patients with major depression. *Clin. Exp. Immunol.* **1992**, *87*, 76–79.
1240. LLOYD, A.; HICKIE, I.; WILSON, A.; WAKEFIELD, D. IMMUNE FUNCTION IN CHRONIC FATIGUE SYNDROME AND DEPRESSION - IMPLICATIONS FOR UNDERSTANDING THESE DISORDERS AND FOR THERAPY. *Clin. Immunother.* **1994**, *2*, 84–88.
1241. Lloyd, A.; Pender, H. Chronic fatigue syndrome: does it need more healthcare resources? *Pharmacoeconomics* 1994, *5*, 460–464.
1242. Lloyd, A.; White, P.; Wessely, S.; Sharpe, M.; Buchwald, D. Comment on "Detection of an infectious retrovirus, XMRV, in blood cells of patients with chronic fatigue syndrome." *Science (80-.).* **2010**, *328*, 825b.
1243. Lo Fo Wong, S.H.; Lagro-Janssen, A.L.M. Intimate partner abuse of women: Identification of victims in medical practice [Mishandeling van vrouwen binnen de partnerrelatie: Signalering in de medische praktijk]. *Ned. Tijdschr. Geneeskd.* **2005**, *149*, 6–9. Work in Dutch
1244. Lo, S.-C.; Pripuzova, N.; Li, B.; Komaroff, A.L.; Hung, G.-C.; Wang, R.; Alter, H.J. RETRACTED: Detection of MLV-related virus gene sequences in blood of patients with chronic fatigue syndrome and healthy blood donors (Retracted article. See vol. 109, pg. 346, 2012). *Proc. Natl. Acad. Sci. U. S. A.* **2010**, *107*, 15874–15879.
1245. Loades, M.E.; Chalder, T. Same, Same But Different? Cognitive Behavioural Treatment Approaches for Paediatric CFS/ME and Depression. *Behav. Cogn. Psychother.* **2017**, *45*, 366–381.
1246. Loades, M.; Brigden, A.; Crawley, E. Current treatment approaches for paediatric CFS/ME. *Paediatr. Child Heal. (United Kingdom)* **2017**, *27*, 432–434.
1247. Loades, M.E.; Rimes, K.A.; Ali, S.; Lievesley, K.; Chalder, T. Does fatigue and distress in a clinical cohort of adolescents with chronic fatigue syndrome correlate with fatigue and distress in their parents? *CHILD CARE Heal. Dev.* **2019**, *45*, 129–137.
1248. Loades, M.E.; Sheils, E.A.; Crawley, E. Treatment for paediatric chronic fatigue syndrome or myalgic encephalomyelitis (CFS/ME) and comorbid depression: a systematic review. *BMJ Open* **2016**, *6*, e012271.
1249. Loades, M.E.; Rimes, K.A.; Ali, S.; Lievesley, K.; Chalder, T. The presence of co-morbid mental health problems in a cohort of adolescents with chronic fatigue syndrome. *Clin. Child Psychol. Psychiatry* **2018**, *23*, 398–408.
1250. Löbel, M.; Mooslechner, A.A.; Bauer, S.; Günther, S.; Letsch, A.; Hanitsch, L.G.; Grabowski, P.; Meisel, C.; Volk, H.-D.; Scheibenbogen, C. Polymorphism in COMT is associated with IgG<inf>3</inf> subclass level and susceptibility to infection in patients with chronic fatigue syndrome. *J. Transl. Med.* **2015**, *13*.
1251. Lobovska, A.; Havlickova, I.; Holub, M. Chronic fatigue syndrome in hospitalized subjects [CHRONICKY UNAVOVY SYNDROM U HOSPITALIZOVANYCH OSOB]. *Prakt. Lek.* **1996**, *76*, 370–372. Work in Czech
1252. Loebel, M.; Eckey, M.; Sotzny, F.; Hahn, E.; Bauer, S.; Grabowski, P.; Zerweck, J.; Holenya, P.; Hanitsch, L.G.; Wittke, K.; et al. Serological profiling of the EBV immune response in Chronic Fatigue Syndrome using a peptide microarray. *PLoS One* **2017**, *12*, e0179124.
1253. Loebel, M.; Mooslechner, A.A.; Bauer, S.; Guenther, S.; Letsch, A.; Hanitsch, L.G.; Grabowski, P.; Meisel, C.; Volk, H.-D.; Scheibenbogen, C. Polymorphism

in COMT is associated with IgG(3) subclass level and susceptibility to infection in patients with chronic fatigue syndrome. *J. Transl. Med.* **2015**, *13*.
1254. Loew, S.; Watson, K. THE PREVALENCE OF SYMPTOMS OF SCOTOPIC SENSITIVITY SYNDROME/MEARES-IRLEN SYNDROME IN SUBJECTS DIAGNOSED WITH CHRONIC FATIGUE SYNDROME. *Int. J. Behav. Med.* **2012**, *19*, S278.
1255. Loew, S.J.; Marsh, N. V; Watson, K. Symptoms of Meares-Irlen/Visual Stress Syndrome in subjects diagnosed with Chronic Fatigue Syndrome. *Int. J. Clin. Heal. Psychol.* **2014**, *14*, 87–92.
1256. Lombardi, V.C.; Ruscetti, F.W.; Das Gupta, J.; Pfost, M.A.; Hagen, K.S.; Peterson, D.L.; Ruscetti, S.K.; Bagni, R.K.; Petrow-Sadowski, C.; Gold, B.; et al. Detection of an infectious retrovirus, XMRV, in blood cells of patients with chronic fatigue syndrome. *Science* **2009**, *326*, 585–589.
1257. Lorduy, K.M.; Liegey-Dougall, A.; Haggard, R.; Sanders, C.N.; Gatchel, R.J. The prevalence of comorbid symptoms of central sensitization syndrome among three different groups of temporomandibular disorder patients. *Pain Pract.* **2013**, *13*, 604–613.
1258. Lorenzo Gómez, M.F.; Castro, S.G. Interstitial cystitis pathophysiology and its relationship with rheumatic, autoimmune, and chronic inflammatory diseases [Relación fisiopatológiea de la cistopatia intersticial eon enfermedades reumátieas, autoinmunes e inflamatorias crónicas.]. *Arch. Esp. Urol.* **2004**, *57*, 25–34. Work in Spanish
1259. Loriol, M. A sociological stance on fatigue and tiredness: Social inequalities, norms and representations [Un regard sociologique sur la fatigue : inégalités sociales, normes et représentations]. *Neurophysiol. Clin.* **2017**, *47*, 87–94. Work in French
1260. Loriol, M. A sociological stance on fatigue and tiredness: Social inequalities, norms and representations. *Neurophysiol. Clin. Neurophysiol.* **2017**, *47*, 87–94.
1261. Lorusso, L.; Mikhaylova, S. V; Capelli, E.; Ferrari, D.; Ngonga, G.K.; Ricevuti, G. Immunological aspects of chronic fatigue syndrome. *Autoimmun. Rev.* **2009**, *8*, 287–291.
1262. Louie, S. *Care of the patient with fatigue*; 2016;
1263. Lowry, T.J.; Pakenham, K.I. Health-related quality of life in chronic fatigue syndrome: predictors of physical functioning and psychological distress. *Psychol. Health Med.* **2008**, *13*, 222–238.
1264. Loy, B.D.; O'Connor, P.J.; Dishman, R.K. Effect of Acute Exercise on Fatigue in People with ME/CFS/SEID: A Meta-analysis. *Med. Sci. Sports Exerc.* **2016**, *48*, 2003–2012.
1265. Lu, C.-C.; Tseng, C.-J.; Tang, H.-S.; Tung, C.-S. Orthostatic intolerance: Potential pathophysiology and therapy. *Chin. J. Physiol.* **2004**, *47*, 101–109.
1266. Lucas, K.E.; Armenian, H.K.; Petersen, G.M.; Rowe, P.C. Familial aggregation of fainting in a case - Control study of neurally mediated hypotension patients who present with unexplained chronic fatigue. *Europace* **2006**, *8*, 846–851.
1267. Lucas, K.E.; Rowe, P.C.; Coresh, J.; Klag, M.J.; Meoni, L.A.; Ford, D.E. Prospective association between hypotension and idiopathic chronic fatigue. *J. Hypertens.* **2004**, *22*, 691–695.
1268. Luczkowiak, J.; Martinez-Prats, L.; Sierra, O.; Fiorante, S.; Rubio, R.; Pulido, F.; Otero, J.R.; Delgado, R. Lack of the Detection of XMRV or Polytropic MLV-Related Sequences in Blood Cells From HIV-1-Infected Patients in Spain. *JAIDS-JOURNAL Acquir. IMMUNE Defic. Syndr.* **2012**, *59*, 101–104.

1269. Ludwig, H.; Bode, L. Borna disease virus: new aspects on infection, disease, diagnosis and epidemiology. *Rev. Sci. Tech. Int. DES Epizoot.* **2000**, *19*, 259–288.
1270. Luft, F.C. The disease that never was. *J. Mol. Med. (Berl).* **2004**, *82*, 723–724.
1271. Lundell, K.; Qazi, S.; Eddy, L.; Uckun, F.M. Clinical activity of folinic acid in patients with chronic fatigue syndrome. *Arzneimittel-Forschung/Drug Res.* **2006**, *56*, 399–404.
1272. Luthra, A.; Wessely, S. Unloading the trunk: neurasthenia, CFS and race. *Soc. Sci. Med.* **2004**, *58*, 2363–2369.
1273. Luty, J. Medically unexplained syndromes: Irritable bowel syndrome, fibromyalgia and chronic fatigue. *BJPsych Adv.* **2018**, *24*, 252–263.
1274. Luyten, P.; Van Houdenhove, B.; Pae, C.-U.; Kempke, S.; Van Wambeke, P. Treatment of chronic fatigue syndrome: Findings, principles and strategies. *Psychiatry Investig.* **2008**, *5*, 209–212.
1275. Lydiard, R.B. Increased prevalence of functional gastrointestinal disorders in panic disorder: Clinical and theoretical implications. *CNS Spectr.* **2005**, *10*, 899–908.
1276. Maaijwee, N.A.M.M.; Schaapsmeerders, P.; Rutten-Jacobs, L.C.A.; Arntz, R.M.; Schoonderwaldt, H.C.; van Dijk, E.J.; Kessels, R.P.C.; de Leeuw, F.-E. Subjective cognitive failures after stroke in young adults: prevalent but not related to cognitive impairment. *J. Neurol.* **2014**, *261*, 1300–1308.
1277. MacDowellCarneiro, A.L.; Pandiri, P.; Foong, S.; Ali, M.; Bellanti, J.; Clauw, D.; Baraniuk, J.N. Allergies are not a cause of chronic fatigue syndrome (CFS): IgE concentrations and prevalence of allergic rhinitis in atopic, CFS, and negative control populations. *J. Allergy Clin. Immunol.* **1996**, *97*, 1022.
1278. MacGregor, E.A.; Rosenberg, J.D.; Kurth, T. Sex-related differences in epidemiological and clinic-based headache studies. *Headache* **2011**, *51*, 843–859.
1279. MacPherson, H.; Scullion, A.; Thomas, K.J.; Walters, S. Patient reports of adverse events associated with acupuncture treatment: A prospective national survey. *Qual. Saf. Heal. Care* **2004**, *13*, 349–355.
1280. Madhavan, G.; Goddard, A.A.; McLeod, K.J. Prevalence and etiology of delayed orthostatic hypotension in adult women. *Arch. Phys. Med. Rehabil.* **2008**, *89*, 1788–1794.
1281. Madigan, J. Beyond hearing loss. *Occup. Health Saf.* **1998**, *67*, 84–89.
1282. Maes, M.; Kubera, M.; Leunis, J.-C.; Berk, M. Increased IgA and IgM responses against gut commensals in chronic depression: Further evidence for increased bacterial translocation or leaky gut. *J. Affect. Disord.* **2012**, *141*, 55–62.
1283. Maes, M.; Libbrecht, I.; Van Hunsel, F.; Lin, A.H.; De Clerck, L.; Stevens, W.; Kenis, G.; de Jongh, R.; Bosmans, E.; Neels, H. The immune-inflammatory pathophysiology of fibromyalgia: increased serum soluble gp130, the common signal transducer protein of various neurotrophic cytokines. *Psychoneuroendocrinology* **1999**, *24*, 371–383.
1284. Maes, M.; Mihaylova, I.; Kubera, M.; Leunis, J.-C. An IgM-mediated immune response directed against nitro-bovine serum albumin (nitro-BSA) in chronic fatigue syndrome (CFS) and major depression: Evidence that nitrosative stress is another factor underpinning the comorbidity between major depression and CFS. *Neuroendocrinol. Lett.* **2008**, *29*, 313–319.
1285. Maes, M.; Mihaylova, I.; Leunis, J.-C. Increased serum IgM antibodies directed against phosphatidyl inositol (Pi) in chronic fatigue syndrome (CFS) and major depression: Evidence that an IgM-mediated immune response against Pi is one factor underpinning the comorbidity between both CFS and depression. *Neuroendocrinol. Lett.* **2007**, *28*, 861–867.

1286. Maes, M.; Mihaylova, I.; Leunis, J.-C. Increased serum IgA and IgM against LPS of enterobacteria in chronic fatigue syndrome (CFS): Indication for the involvement of gram-negative enterobacteria in the etiology of CFS and for the presence of an increased gut-intestinal permeability. *J. Affect. Disord.* **2007**, *99*, 237–240.
1287. Maes, M.; Mihaylova, I.; Leunis, J.-C. Chronic fatigue syndrome is accompanied by an IgM-related immune response directed against neopitopes formed by oxidative or nitrosative damage to lipids and proteins. *Neuroendocrinol. Lett.* **2006**, *27*, 615–621.
1288. Maes, M.; Ringel, K.; Kubera, M.; Anderson, G.; Morris, G.; Galecki, P.; Geffard, M. In myalgic encephalomyelitis/chronic fatigue syndrome, increased autoimmune activity against 5-HT is associated with immuno-inflammatory pathways and bacterial translocation. *J. Affect. Disord.* **2013**, *150*, 223–230.
1289. Maes, M.; Ringel, K.; Kubera, M.; Berk, M.; Rybakowski, J. Increased autoimmune activity against 5-HT: A key component of depression that is associated with inflammation and activation of cell-mediated immunity, and with severity and staging of depression. *J. Affect. Disord.* **2012**, *136*, 386–392.
1290. Maes, M. An intriguing and hitherto unexplained co-occurrence: Depression and chronic fatigue syndrome are manifestations of shared inflammatory, oxidative and nitrosative (IO&NS) pathways. *Prog. Neuropsychopharmacol. Biol. Psychiatry* **2011**, *35*, 784–794.
1291. Maes, M.; Mihaylova, I.; Kubera, M.; Uytterhoeven, M.; Vrydags, N.; Bosmans, E. Lower whole blood glutathione peroxidase (GPX) activity in depression, but not in myalgic encephalomyelitis / chronic fatigue syndrome: another pathway that may be associated with coronary artery disease and neuroprogression in depression. *Neuro Endocrinol. Lett.* **2011**, *32*, 133–140.
1292. Maes, M.; Mihaylova, I.; Kubera, M.; Uytterhoeven, M.; Vrydags, N.; Bosmans, E. Increased plasma peroxides and serum oxidized low density lipoprotein antibodies in major depression: markers that further explain the higher incidence of neurodegeneration and coronary artery disease. *J. Affect. Disord.* **2010**, *125*, 287–294.
1293. Maes, M.; Mihaylova, I.; Kubera, M.; Uytterhoeven, M.; Vrydags, N.; Bosmans, E. Increased 8-hydroxy-deoxyguanosine, a marker of oxidative damage to DNA, in major depression and myalgic encephalomyelitis / chronic fatigue syndrome. *Neuro Endocrinol. Lett.* **2009**, *30*, 715–722.
1294. Maes, M.; Mihaylova, I.; Kubera, M.; Uytterhoeven, M.; Vrydags, N.; Bosmans, E. Coenzyme Q10 deficiency in myalgic encephalomyelitis/chronic fatigue syndrome (ME/CFS) is related to fatigue, autonomic and neurocognitive symptoms and is another risk factor explaining the early mortality in ME/CFS due to cardiovascular disorder. *Neuro Endocrinol. Lett.* **2009**, *30*, 470–476.
1295. Maes, M.; Twisk, F.N.M.; Ringel, K. Inflammatory and cell-mediated immune biomarkers in myalgic encephalomyelitis/chronic fatigue syndrome and depression: inflammatory markers are higher in myalgic encephalomyelitis/chronic fatigue syndrome than in depression. *Psychother. Psychosom.* **2012**, *81*, 286–295.
1296. Magen, E.; Schlesinger, M.; David, M.; Ben-Zion, I.; Vardy, D. Selective IgE deficiency, immune dysregulation, and autoimmunity. *Allergy Asthma Proc.* **2014**, *35*, e27–e33.
1297. Maggi, F.; Bazzichi, L.; Sernissi, F.; Mazzetti, P.; Lanini, L.; Scarpellini, P.; Consensi, A.; Giacomelli, C.; Macera, L.; Vatteroni, M.L.; et al. Absence of

xenotropic murine leukemia virus-related virus in Italian patients affected by chronic fatigue syndrome, fibromyalgia, or rheumatoid arthritis. *Int. J. Immunopathol. Pharmacol.* 2012, *25*, 523–529.
1298. Maggi, F.; Focosi, D.; Lanini, L.; Sbranti, S.; Mazzetti, P.; Macera, L.; Davini, S.; De Donno, M.; Mariotti, M.L.; Antonelli, G.; et al. Xenotropic murine leukaemia virus-related virus is not found in peripheral blood cells from treatment-naive human immunodeficiency virus-positive patients. *Clin. Microbiol. Infect.* **2012**, *18*, 184–188.
1299. Magnus, P.; Brubakk, O.; Nyland, H.; Wold, B.H.; Gjessing, H.K.; Brandt, I.; Eidem, T.; Nokleby, H.; Stene-Larsen, G. Vaccination as teenagers against meningococcal disease and the risk of the chronic fatigue syndrome. *Vaccine* **2009**, *27*, 23–27.
1300. Magnus, P.; Gunnes, N.; Tveito, K.; Bakken, I.J.; Ghaderi, S.; Stoltenberg, C.; Hornig, M.; Lipkin, W.I.; Trogstad, L.; Haberg, S.E. Chronic fatigue syndrome/myalgic encephalomyelitis (CFS/ME) is associated with pandemic influenza infection, but not with an adjuvanted pandemic influenza vaccine. *Vaccine* **2015**, *33*, 6173–6177.
1301. Magora, A.; Vatine, J.J. Fibromyalgia: Review of a common, still controversial, disease. *PAIN Clin.* **1995**, *8*, 269–275.
1302. Mahjoub, F.; Salari, R.; Noras, M.R.; Yousefi, M. Are Traditional Remedies Useful in Management of Fibromyalgia and Chronic Fatigue Syndrome? A Review Study. *J. EVIDENCE-BASED Integr. Med.* **2017**, *22*, 1011–1016.
1303. Mahnke, C.; Kashaiya, P.; Rössler, J.; Bannert, H.; Levin, A.; Blattner, W.A.; Dietrich, M.; Luande, J.; Löchelt, M.; Friedman-Kien, A.E.; et al. Human spumavirus antibodies in sera from African patients. *Arch. Virol.* **1992**, *123*, 243–253.
1304. Majer, M.; Jones, J.F.; Unger, E.R.; Youngblood, L.S.; Decker, M.J.; Gurbaxani, B.; Heim, C.; Reeves, W.C. Perception versus polysomnographic assessment of sleep in CFS and non-fatigued control subjects: Results from a population-based study. *BMC Neurol.* **2007**, *7*.
1305. Mak, A.; Ho, R.C.M.; Lau, C.S. Clinical implications of neuropsychiatric systemic lupus erythematosus. *Adv. Psychiatr. Treat.* **2009**, *15*, 451–458.
1306. Mak, K.Y. The interesting but confusing phenomenon of neurasthenia and chronic fatigue syndrome. *Hong Kong Pract.* **2001**, *23*, 390–396.
1307. Makarova, N.; Zhao, C.; Zhang, Y.; Bhosle, S.; Suppiah, S.; Rhea, J.M.; Kozyr, N.; Arnold, R.S.; Ly, H.; Molinaro, R.J.; et al. Antibody responses against xenotropic murine leukemia virus-related virus envelope in a murine model. *PLoS One* **2011**, *6*.
1308. Malhotra, S.K.; Wig, N.; Nakra, D. Drug abuse among physicians - Specific concerns in anaesthesiologists. *J. Int. Med. Sci. Acad.* **2005**, *18*, 60–62.
1309. Malleson, P.N. Pain syndromes, disability, and chronic disease in childhood. *Curr. Opin. Rheumatol.* **1991**, *3*, 860–866.
1310. Maloney, E.M.; Boneva, R.S.; Lin, J.-M.S.; Reeves, W.C. Chronic fatigue syndrome is associated with metabolic syndrome: results from a case-control study in Georgia. *Metabolism.* **2010**, *59*, 1351–1357.
1311. Maloney, E.M.; Boneva, R.; Nater, U.M.; Reeves, W.C. Chronic fatigue syndrome and high allostatic load: results from a population-based case-control study in Georgia. *Psychosom. Med.* **2009**, *71*, 549–556.

1312. Maloney, E.M.; Gurbaxani, B.M.; Jones, J.F.; de Souza Coelho, L.; Pennachin, C.; Goertzel, B.N. Chronic fatigue syndrome and high allostatic load. *Pharmacogenomics* **2006**, *7*, 467–473.
1313. Malterud, K. Subjective symptoms without objective findings - a challenge to general medical theory and practice. *Ugeskr. Laeger* **2001**, *163*, 6729–6734.
1314. MANIAN, F.A. SIMULTANEOUS MEASUREMENT OF ANTIBODIES TO EPSTEIN-BARR-VIRUS, HUMAN HERPESVIRUS-6, HERPES-SIMPLEX VIRUS TYPE-1 AND TYPE-2, AND 14 ENTEROVIRUSES IN CHRONIC-FATIGUE-SYNDROME - IS THERE EVIDENCE OF ACTIVATION OF A NONSPECIFIC POLYCLONAL IMMUNE-RESPONSE. *Clin. Infect. Dis.* **1994**, *19*, 448–453.
1315. Manian, F.A. Simultaneous measurement of antibodies to epstein-barr virus, human herpesvirus 6 herpes simplex virus types 1 and 2 and 14 enteroviruses in chronic fatigue syndrome: is there evidence of activation of a nonspecific polyclonal immune response? *Clin. Infect. Dis.* **1994**, *19*, 448–453.
1316. Mann, A.H.; Mc Donald, E.; Cope, H.; Pelosi, A.; David, A. [Epidemiologic study of chronic fatigue in primary care (general practice)]. *Encephale.* **1994**, *20 Spec No 3*, 575–579.
1317. Mann, A.H.; McDonald, E.; Cope, H.; Pelosi, A.; David, A. An epidemiological study of chronic fatigue in primary care [ETUDE EPIDEMIOLOGIQUE DE LA FATIGUE CHRONIQUE EN MILIEU DE SOINS PRIMAIRES (MEDECINE GENERALE)]. *Encephale* **1994**, *20*, 575–579. Work in French
1318. Mannarino, M.R.; Di Filippo, F.; Pirro, M. Obstructive sleep apnea syndrome. *Eur. J. Intern. Med.* **2012**, *23*, 586–593.
1319. Manousek, J.; Privarova, L.; Pavkova-Goldbergova, M. *Metal hypersensitivity as the cause of chronic fatigue syndrome: Case report*; 2014;
1320. Manu, P.; Affleck, G.; Tennen, H.; Morse, P.A.; Escobar, J.I. Hypochondriasis influences quality-of-life outcomes in patients with chronic fatigue. *Psychother. Psychosom.* **1996**, *65*, 76–81.
1321. Manu, P.; Lane, T.J.; Matthews, D.A. The pathophysiology of chronic fatigue syndrome: Confirmations, contradictions, and conjectures. *Int. J. Psychiatry Med.* **1992**, *22*, 397–408.
1322. Manu, P.; Lane, T.J.; Matthews, D.A. Chronic fatigue syndromes in clinical practice. *Psychother. Psychosom.* **1992**, *58*, 60–68.
1323. MANU, P.; LANE, T.J.; Matthews, D.A. CHRONIC FATIGUE AND CHRONIC FATIGUE SYNDROME - CLINICAL EPIDEMIOLOGY AND ETIOLOGIC CLASSIFICATION. *CIBA Found. Symp.* **1993**, *173*, 23–42. Proceedings of the Symposium on chronic fatigue syndrome in London, UK.
1324. Manu, P.; Lane, T.J.; Matthews, D.A.; Castriotta, R.J.; Watson, R.K.; Abeles, M. Alpha-delta sleep in patients with a chief complaint of chronic fatigue. *South. Med. J.* **1994**, *87*, 465–470.
1325. Manu, P.; Matthews, D.A.; Lane, T.J. Food intolerance in patients with chronic fatigue. *Int. J. Eat. Disord.* **1993**, *13*, 203–209.
1326. Maoz, D.; Shoenfeld, Y. Editorials - Chronic fatigue syndrome. *Harefuah* **2006**, *145*, 272–275.
1327. Maoz, D.; Shoenfeld, Y. [Chronic fatigue syndrome]. *Harefuah* 2006, *145*, 272–275,318,319. Work in Hebrew
1328. Maquet, D.; Croisier, J.L.; Crielaard, J.M. What happens to the fibromyalgia syndrome? [Le point sur la fibromyalgie]. *Ann. Readapt. Med. Phys.* **2001**, *44*, 316–325. Work in French

1329. Maquet, D.; Croisier, J.L.; Crielaard, J.M. Fibromyalgia in the year 2000 [La fibromyalgie en l'an 2000]. *Rev. Med. Liege* **2000**, *55*, 991–997. Work in French
1330. Maquet, D.; Croisier, J.L.; Crielaard, J.M. [Fibromyalgia in the year 2000]. *Rev. Med. Liege* **2000**, *55*, 991–997. Work in French
1331. Maquet, D.; Demoulin, C.; Crielaard, J.M. A view on the chronic fatigue syndrome [Le point sur le syndrome de fatigue chronique]. *Ann. Readapt. Med. Phys.* **2006**, *49*, 337–347. Work in French
1332. Maquet, D.; Demoulin, C.; Crielaard, J.-M. Chronic fatigue syndrome: a systematic review. *Ann. Readapt. Med. Phys.* **2006**, *49*, 418–427.
1333. Marathe, C.S.; Torpy, D.J. A role for corticosteroid-binding globulin variants in stress-related disorders. *Expert Rev. Endocrinol. Metab.* **2012**, *7*, 301–308.
1334. Marcovitch, H. Managing chronic fatigue syndrome in children - Liaise with family and teachers to keep morale high and minimise disability. *Br. Med. J.* **1997**, *314*, 1635–1636.
1335. Marcusson, J.A. The frequency of mercury intolerance in patients with chronic fatigue syndrome and healthy controls. *Contact Dermatitis* 1999, *41*, 60–61.
1336. Maric, D.; Brkic, S.; Mikic, A.N.; Tomic, S.; Cebovic, T.; Turkulov, V. Multivitamin mineral supplementation in patients with chronic fatigue syndrome. *Med. Sci. Monit.* **2014**, *20*, 47–53.
1337. Mariman, A.; Delesie, L.; Tobback, E.; Hanoulle, I.; Sermijn, E.; Vermeir, P.; Pevernagie, D.; Vogelaers, D. Prevalence of primary sleep disorders in a large sample of patients with presumed chronic fatigue syndrome referred to a tertiary care referral centre. *J. Sleep Res.* **2012**, *21*, 90.
1338. Mariman, A.; Delesie, L.; Tobback, E.; Hanoulle, I.; Sermijn, E.; Vermeir, P.; Pevernagie, D.; Vogelaers, D. Undiagnosed and comorbid disorders in patients with presumed chronic fatigue syndrome. *J. Psychosom. Res.* **2013**, *75*, 491–496.
1339. Mariman, A.; Vogelaers, D.; Hanoulle, I.; Delesie, L.; Pevernagie, D. Subjective sleep quality and daytime sleepiness in a large sample of patients with chronic fatigue syndrome (CFS). *Acta Clin. Belg.* **2012**, *67*, 19–24.
1340. Mariman, A.N.; Vogelaers, D.P.; Tobback, E.; Delesie, L.M.; Hanoulle, I.P.; Pevernagie, D.A. Sleep in the chronic fatigue syndrome. *Sleep Med. Rev.* **2013**, *17*, 193–199.
1341. Marmion, B.P.; Storm, P.A.; Ayres, J.G.; Semendric, L.; Mathews, L.; Winslow, W.; Turra, M.; Harris, R.J. Long-term persistence of Coxiella burnetii after acute primary Q fever. *QJM-AN Int. J. Med.* **2005**, *98*, 7–20.
1342. Maroti, D.; Molander, P.; Bileviciute-Ljungar, I. Differences in alexithymia and emotional awareness in exhaustion syndrome and chronic fatigue syndrome. *Scand. J. Psychol.* **2017**, *58*, 52–61.
1343. Marques, M.; De Gucht, V.; Leal, I.; Maes, S. A cross-cultural perspective on psychological determinants of chronic fatigue syndrome: a comparison between a Portuguese and a Dutch patient sample. *Int. J. Behav. Med.* **2013**, *20*, 229–238.
1344. Marshall, G.S. Report of a workshop on the epidemiology, natural history, and pathogenesis of chronic fatigue syndrome in adolescents. *J. Pediatr.* **1999**, *134*, 395–405.
1345. Marshall, R.; Paul, L.; Wood, L. The search for pain relief in people with chronic fatigue syndrome: a descriptive study. *Physiother. Theory Pract.* **2011**, *27*, 373–383.
1346. Martikainen, K.; Partinen, M.; Hasan, J.; Urponen, H.; Vuori, I.; Laippala, P. Natural evolution of sleepiness. A 5-year follow-up study in a middle-aged population. *Eur. J. Neurol.* **1998**, *5*, 355–363.

1347. Martin-Subero, M.; Anderson, G.; Kanchanatawan, B.; Berk, M.; Maes, M. Comorbidity between depression and inflammatory bowel disease explained by immune-inflammatory, oxidative, and nitrosative stress; tryptophan catabolite; and gut-brain pathways. *CNS Spectr.* **2016**, *21*, 184–198.
1348. Martin, A.; Chalder, T.; Rief, W.; Braehler, E. The relationship between chronic fatigue and somatization syndrome: a general population survey. *J. Psychosom. Res.* **2007**, *63*, 147–156.
1349. Martin, B.L.; Nelson, M.R.; Hershey, J.N.; Engler, R.J.M. Adverse reactions to vaccines. *Clin. Rev. Allergy Immunol.* **2003**, *24*, 263–275.
1350. Martin, F.; Bangham, C.R.M.; Ciminale, V.; Lairmore, M.D.; Murphy, E.L.; Switzer, W.M.; Mahieux, R. Conference highlights of the 15th international conference on human retrovirology: HTLV and related retroviruses, 4-8 june 2011, Leuven, Gembloux, Belgium. *Retrovirology* **2011**, *8*.
1351. Martinez, S.; Guilleminault, C. Periodic leg movements in prepubertal children with sleep disturbance. *Dev. Med. Child Neurol.* **2004**, *46*, 765–770.
1352. Marx, J.J.M. Prevention of organ failure in hereditary haemochromatosis. *Neth. J. Med.* **2002**, *60*, 419–422.
1353. Masri, T.J.; Gonzales, C.G.; Kushida, C.A. Idiopathic hypersomnia. *Sleep Med. Clin.* **2012**, *7*, 283–289.
1354. Masruha, M.R.; Lin, J.; de Souza Vieira, D.S.; Minett, T.S.C.; Cipolla-Neto, J.; Zukerman, E.; Vilanova, L.C.P.; Peres, M.F.P. Urinary 6-sulphatoxymelatonin levels are depressed in chronic migraine and several comorbidities. *Headache* **2010**, *50*, 413–419.
1355. Matheis, A.; Martens, U.; Kruse, J.; Enck, P. Irritable bowel syndrome and chronic pelvic pain: A singular or two different clinical syndrome? *WORLD J. Gastroenterol.* **2007**, *13*, 3446–3455.
1356. Mathew, S.J.; Mao, X.; Keegan, K.A.; Levine, S.M.; Smith, E.L.P.; Heier, L.A.; Otcheretko, V.; Coplan, J.D.; Shungu, D.C. Ventricular cerebrospinal fluid lactate is increased in chronic fatigue syndrome compared with generalized anxiety disorder: an in vivo 3.0 T H-1 MRS imaging study. *NMR Biomed.* **2009**, *22*, 251–258.
1357. Mathieu, N. Somatic comorbidities in irritable bowel syndrome: fibromyalgia, chronic fatigue syndrome, and interstitial cystitis [Comorbidités somatiques dans le Syndrome de l'Intestin Irritable : fibromyalgie, syndrome de fatigue chronique et cystite interstitielle/syndrome de la vessie douloureuse]. *Gastroenterol. Clin. Biol.* **2009**, *33*, S17–S25. Work in French
1358. Mathieu, N. [Somatic comorbidities in irritable bowel syndrome: fibromyalgia, chronic fatigue syndrome, and interstitial cystitis]. *Gastroenterol. Clin. Biol.* **2009**, *33 Suppl 1*, S17-25.
1359. Matsuda, Y.; Matsui, T.; Kataoka, K.; Fukada, R.; Fukuda, S.; Kuratsune, H.; Tajima, S.; Yamaguti, K.; Kato, Y.H.; Kiriike, N. A two-year follow-up study of chronic fatigue syndrome comorbid with psychiatric disorders. *Psychiatry Clin. Neurosci.* **2009**, *63*, 365–373.
1360. Matsumoto, Y.; Ninomiya, S. [Allergy among Japanese patients with chronic fatigue syndrome]. *Arerugi* **1992**, *41*, 1722–1725. Work in Japanese
1361. MATTHEWS, D.A.; DOWELL, E.H.; MANU, P.; LANE, T.J. EFFECT OF CHANGES IN CRITERIA UPON THE PREVALENCE OF CHRONIC FATIGUE SYNDROME AMONG PATIENTS WITH CHRONIC FATIGUE. *Clin. Res.* **1993**, *41*, A521.

1362. Maubec, E.; Pinquier, L.; Viguier, M.; Caux, F.; Amsler, E.; Aractingi, S.; Chafi, H.; Janin, A.; Cayuela, J.-M.; Dubertret, L.; et al. Vaccination-induced cutaneous pseudolymphoma. *J. Am. Acad. Dermatol.* **2005**, *52*, 623–629.
1363. Mawle, A.C. Chronic fatigue syndrome. *Immunol. Invest.* **1997**, *26*, 269–273. Proceedings of the 13th International Convocation on Immunology - Immunological and Molecular Diagnosis of Infectious Disease in Buffalo, USA.
1364. Mawle, A.C.; Nisenbaum, R.; Dobbins, J.G.; Gary, H.E.J.; Stewart, J.A.; Reyes, M.; Steele, L.; Schmid, D.S.; Reeves, W.C. Seroepidemiology of chronic fatigue syndrome: a case-control study. *Clin. Infect. Dis.* **1995**, *21*, 1386–1389.
1365. Maxmen, A. A reboot for chronic fatigue syndrome research. *Nature* **2018**, *553*, 14–17.
1366. Maxmen, A. The invisible disability. *Nature* **2018**, *553*, 14–17.
1367. Mayer, T.G.; Neblett, R.; Cohen, H.; Howard, K.J.; Choi, Y.H.; Williams, M.J.; Perez, Y.; Gatchel, R.J. The Development and Psychometric Validation of the Central Sensitization Inventory. *PAIN Pract.* **2012**, *12*, 276–285.
1368. McBeth, J.; Tomenson, B.; Chew-Graham, C.A.; Macfarlane, G.J.; Jackson, J.; Littlewood, A.; Creed, F.H. Common and unique associated factors for medically unexplained chronic widespread pain and chronic fatigue. *J. Psychosom. Res.* **2015**, *79*, 484–491.
1369. McBeth, J.; Tajar, A.; O'Neill, T.W.; Macfarlane, G.J.; Pye, S.R.; Bartfai, G.; Boonen, S.; Bouillon, R.; Casanueva, F.; Finn, J.D.; et al. Perturbed Insulin-like Growth Factor-1 (IGF-1) and IGF Binding Protein-3 Are Not Associated with Chronic Widespread Pain in Men: Results from the European Male Ageing Study. *J. Rheumatol.* **2009**, *36*, 2523–2530.
1370. McCauley, L.A.; Joos, S.K.; Barkhuizen, A.; Shuell, T.; Tyree, W.A.; Bourdette, D.N. Chronic fatigue in a population-based study of Gulf War veterans. *Arch. Environ. Health* **2002**, *57*, 340–348.
1371. McClure, M.; Kaye, S. Can detection of xenotropic murine leukemia virus-related virus be linked to chronic fatigue syndrome? *Expert Rev. Mol. Diagn.* **2010**, *10*, 537–539.
1372. McCue, P.; Buchanan, T.; Martin, C.R. Screening for psychological distress using internet administration of the Hospital Anxiety and Depression Scale (HADS) in individuals with chronic fatigue syndrome. *Br. J. Clin. Psychol.* **2006**, *45*, 483–498.
1373. McCully, K.K.; Sisto, S.A.; Natelson, B.H. Use of exercise for treatment of chronic fatigue syndrome. *Sport. Med.* **1996**, *21*, 35–48.
1374. McDermott, C.; Al Haddabi, A.; Akagi, H.; Selby, M.; Cox, D.; Lewith, G. What is the current NHS service provision for patients severely affected by chronic fatigue syndrome/myalgic encephalomyelitis? A national scoping exercise. *BMJ Open* **2014**, *4*, e005083.
1375. McDonald, C.; Koshi, S.; Busner, L.; Kavi, L.; Newton, J.L. Postural tachycardia syndrome is associated with significant symptoms and functional impairment predominantly affecting young women: A UK perspective. *BMJ Open* **2014**, *4*.
1376. MCDONALD, E.; DAVID, A.S.; PELOSI, A.J.; MANN, A.H. CHRONIC FATIGUE IN PRIMARY-CARE ATTENDERS. *Psychol. Med.* **1993**, *23*, 987–998.
1377. McFarlane, A.C. Stress-related musculoskeletal pain. *Best Pract. Res. Clin. Rheumatol.* **2007**, *21*, 549–565.

1378. McFetridge-Durdle, J.A.; Routledge, F.S.; Sampalli, T.; Fox, R.; Livingston, H.; Adams, B. Hemodynamic Response to Postural Shift in Women with Multiple Chemical Sensitivities. *Biol. Res. Nurs.* **2009**, *10*, 267–273.
1379. McGrath, S. Omission of data weakens the case for causal mediation in the PACE Trial. *The Lancet Psychiatry* **2015**, *2*, e7–e8.
1380. McGregor, N.R.; Dunstan, R.H.; Butt, H.L.; Roberts, T.K.; Klineberg, I.J.; Zerbes, M. A preliminary assessment of the association of SCL-90-R psychological inventory responses with changes in urinary metabolites in patients with chronic fatigue syndrome. *J. Chronic Fatigue Syndr.* **1997**, *3*, 17–37.
1381. Mcgregor, N.R.; Dunstan, R.H.; Zerbes, M.; Butt, H.L.; Roberts, T.K.; Klineberg, I.J. Preliminary determination of a molecular basis to chronic fatigue syndrome. *Biochem. Mol. Med.* **1996**, *57*, 73–80.
1382. McGregor, N.R.; Dunstan, R.H.; Zerbes, M.; Butt, H.L.; Roberts, T.K.; Klineberg, I.J. Preliminary determination of the association between symptom expression and urinary metabolites in subjects with chronic fatigue syndrome. *Biochem. Mol. Med.* **1996**, *58*, 85–92.
1383. McGregor, N.R.; Zerbes, M.; Niblett, S.H.; Dunstan, R.H.; Roberts, T.K.; Butt, H.L.; Klineberg, I.J. Coagulase-negative staphylococcal membrane-damaging toxins, pain intensity, and metabolic changes in temporomandibular disorder patients with chronic muscle pain. *J. Orofac. Pain* **2003**, *17*, 125–132.
1384. McGregor, N.R.; Zerbes, M.; Niblett, S.H.; Dunstan, R.H.; Roberts, T.K.; Butt, H.L.; Klineberg, I.J. Pain intensity, illness duration, and protein catabolism in temporomandibular disorder patients with chronic muscle pain. *J. Orofac. Pain* **2003**, *17*, 112–124.
1385. McIlwain, H.H. Glucocorticoid-induced osteoporosis: Pathogenesis, diagnosis, and management. *Prev. Med. (Baltim).* **2003**, *36*, 243–249.
1386. McIntyre, R.S.; Konarski, J.Z.; Soczynska, J.K.; Wilkins, K.; Panjwani, G.; Bouffard, B.; Bottas, A.; Kennedy, S.H. Medical comorbidity in bipolar disorder: Implications for functional outcomes and health service utilization. *Psychiatr. Serv.* **2006**, *57*, 1140–1144.
1387. McKenzie, D.P.; Sim, M.R.; Clarke, D.M.; Forbes, A.B.; Ikin, J.F.; Kelsall, H.L. Developing a brief depression screen and identifying associations with comorbid physical and psychological illness in Australian Gulf War veterans. *J. Psychosom. Res.* **2015**, *79*, 566–573.
1388. McKenzie, R.; O'Fallon, A.; Dale, J.; Demitrack, M.; Sharma, G.; Deloria, M.; Garcia-Borreguero, D.; Blackwelder, W.; Straus, S.E. Low-dose hydrocortisone for treatment of chronic fatigue syndrome - A randomized controlled trial. *JAMA-JOURNAL Am. Med. Assoc.* **1998**, *280*, 1061–1066.
1389. Mcloughlin, M.J.; Colbert, L.H.; Stegner, A.J.; Cook, D.B. Are Women with Fibromyalgia Less Physically Active than Healthy Women? *Med. Sci. Sports Exerc.* **2011**, *43*, 905–912.
1390. McManimen, S.L.; Jason, L.A.; Williams, Y.J. Variability in symptoms complicates utility of case definitions. *FATIGUE-BIOMEDICINE Heal. Behav.* **2015**, *3*, 164–172.
1391. McSherry, J. Chronic fatigue syndrome. A fresh look at an old problem. *Can. Fam. Physician* **1993**, *39*, 336–340.
1392. MD, M.P.; P, B.P. [The chronic fatigue and neurasthenia in the student population]. *Actas Esp. Psiquiatr.* **1999**, *27*, 14–21. Work in Spanish

1393. Mears, C.J.; Taylor, R.R.; Jordan, K.M.; Binns, H.J. Sociodemographic and symptom correlates of fatigue in an adolescent primary care sample. *J. Adolesc. Health* **2004**, *35*, 528e.21-6.
1394. MECHANIC, D. CHRONIC FATIGUE SYNDROME AND THE TREATMENT PROCESS. *CIBA Found. Symp.* **1993**, *173*, 318–341.
1395. Medow, M.S. Postural tachycardia syndrome from a pediatrics perspective. *J. Pediatr.* **2011**, *158*, 4–6.
1396. Meeus, M.; Hermans, L.; Ickmans, K.; Struyf, F.; Van Cauwenbergh, D.; Bronckaerts, L.; De Clerck, L.S.; Moorken, G.; Hans, G.; Grosemans, S.; et al. Endogenous pain modulation in response to exercise in patients with rheumatoid arthritis, patients with chronic fatigue syndrome and comorbid fibromyalgia, and healthy controls: a double-blind randomized controlled trial. *Pain Pract.* **2015**, *15*, 98–106.
1397. Meeus, M.; Nijs, J.; Meirleir, K. De Chronic musculoskeletal pain in patients with the chronic fatigue syndrome: a systematic review. *Eur. J. Pain* **2007**, *11*, 377–386.
1398. Meggs, W.J. Gulf War Syndrome, Chronic Fatigue Syndrome, and the Multiple Chemical Sensitivity Syndrome: Stirring the Cauldron of Confusion. *Arch. Environ. Health* **1999**, *54*, 309–311.
1399. Mehta, R.; Gerardin, P.; de Brito, C.A.A.; Soares, C.N.; Brito Ferreira, M.L.; Solomon, T. Reply to Simon et al. on "The neurological complications of chikungunya virus: A systematic review." *Rev. Med. Virol.* **2018**, *28*.
1400. Meisler, J.G. Toward optimal health: The experts discuss fibromyalgia. *J. Women's Heal. Gender-Based Med.* **2000**, *9*, 1055–1060.
1401. Melidis, C.; Denham, S.L.; Hyland, M.E. A test of the adaptive network explanation of functional disorders using a machine learning analysis of symptoms. *Biosystems.* **2018**, *165*, 22–30.
1402. Menéndez-Arias, L. Evidence and controversies on the role of XMRV in prostate cancer and chronic fatigue syndrome. *Rev. Med. Virol.* **2011**, *21*, 3–17.
1403. Menon, P.M.; Nasrallah, H.A.; Reeves, R.R.; Ali, J.A. Hippocampal dysfunction in Gulf War Syndrome. A proton MR spectroscopy study. *BRAIN Res.* **2004**, *1009*, 189–194.
1404. Mensah, F.; Bansal, A.; Berkovitz, S.; Sharma, A.; Reddy, V.; Leandro, M.J.; Cambridge, G. Extended B cell phenotype in patients with myalgic encephalomyelitis/chronic fatigue syndrome: a cross-sectional study. *Clin. Exp. Immunol.* **2016**, *184*, 237–247.
1405. MERIKANGAS, K.; ANGST, J. NEURASTHENIA IN A LONGITUDINAL COHORT STUDY OF YOUNG-ADULTS. *Psychol. Med.* **1994**, *24*, 1013–1024.
1406. Mesters, P.; Clumeck, N.; Delroisse, S.; Gozlan, S.; Le Polain, M.; Massart, A.-C.; Pitchot, W. Professional fatigue syndrome (burnout): Part 2: From therapeutic management [Syndrome de fatigue professionnelle (burnout) 2 ème partie: De la prise en charge thérapeutique]. *Rev. Med. Liege* **2017**, *72*, 301–307. Work in French
1407. Metcalf, L.N.; McGregor, N.R.; Roberts, T.K. Membrane damaging toxins from coagulase-negative Staphylococcus are associated with self-reported temporomandibular disorder (TMD) in patients with chronic fatigue syndrome. *J. Chronic Fatigue Syndr.* **2004**, *12*, 25–43.
1408. Mi, Z.; Lu, Y.; Zhang, S.; An, X.; Wang, X.; Chen, B.; Wang, Q.; Tong, Y. Absence of xenotropic murine leukemia virus-related virus in blood donors in China. *Transfusion* **2012**, *52*, 326–331.

1409. Michel, J.-P. Herpes zoster vaccine: a shot to maintain wellbeing. *Aging Clin. Exp. Res.* **2015**, *27*.
1410. Michiels, V.; Cluydts, R. Neuropsychological functioning in chronic fatigue syndrome: a review. *Acta Psychiatr. Scand.* **2001**, *103*, 84–93.
1411. Michielsen, H.J.; Van Houdenhove, B.; Leirs, I.; Vandenbroeck, A.; Onghena, P. Depression, attribution style and self-esteem in chronic fatigue syndrome and fibromyalgia patients: Is there a link? *Clin. Rheumatol.* **2006**, *25*, 183–188.
1412. Miike, T. [Childhood chronic fatigue syndrome]. *Nihon Rinsho.* **2007**, *65*, 1099–1104. Work in Japanese
1413. Mikovits, J.A.; Lombardi, V.C.; Ruscetti, F.W. Xenotropic murine leukemia virus-related virus: Current research, disease associations and therapeutic opportunities. *Therapy* **2010**, *7*, 377–384.
1414. Mikovits, J.A.; Huang, Y.; Pfost, M.A.; Lombardi, V.C.; Bertolette, D.C.; Hagen, K.S.; Ruscetti, F.W. Distribution of Xenotropic Murine Leukemia Virus-Related Virus (XMRV) Infection in Chronic Fatigue Syndrome and Prostate Cancer. *AIDS Rev.* **2010**, *12*, 149–152.
1415. Miller, G. Molecular approaches to epidemiologic evaluation of viruses as risk factors for patients who have chronic fatigue syndrome. *Rev. Infect. Dis.* **1991**, *13 Suppl 1*, S119-22.
1416. Million, M.; Lepidi, H.; Raoult, D. Q fever: Current diagnosis and treatment options [Fièvre Q : actualités diagnostiques et thérapeutiques]. *Med. Mal. Infect.* **2009**, *39*, 82–94. Work in French
1417. Million, M.; Lepidi, H.; Raoult, D. [Q fever: current diagnosis and treatment options]. *Med. Mal. Infect.* **2009**, *39*, 82–94. Work in French
1418. Milrad, S.F.; Hall, D.L.; Jutagir, D.R.; Lattie, E.G.; Ironson, G.H.; Wohlgemuth, W.; Nunez, M.V.; Garcia, L.; Czaja, S.J.; Perdomo, D.M.; et al. Poor sleep quality is associated with greater circulating pro-inflammatory cytokines and severity and frequency of chronic fatigue syndrome/myalgic encephalomyelitis (CFS/ME) symptoms in women. *J. Neuroimmunol.* **2017**, *303*, 43–50.
1419. Milton, J.D.; Clements, G.B.; Edwards, R.H.T. Immune responsiveness in chronic fatigue syndrome. *Postgrad. Med. J.* **1991**, *67*, 532–537.
1420. Minhas, F.A.; Nizami, A.T. Somatoform disorders: Perspectives from Pakistan. *Int. Rev. PSYCHIATRY* **2006**, *18*, 55–60.
1421. Minoia, C.; Ciavarella, S.; Lerario, G.; Daniele, A.; De Summa, S.; Napolitano, M.; Guarini, A. Improvable Lifestyle Factors in Lymphoma Survivors. *Acta Haematol.* **2018**, *139*, 235–237.
1422. Minowa, M.; Jiamo, M. Descriptive epidemiology of chronic fatigue syndrome based on a nationwide survey in Japan. *J. Epidemiol.* **1996**, *6*, 75–80.
1423. Mirkin, D.; Murphy-Barron, C.; Iwasaki, K. Actuarial analysis of private payer administrative claims data for women with endometriosis. *J. Manag. Care Pharm.* **2007**, *13*, 262–272.
1424. Miro, O.; Font, C.; FernandezSola, J.; Casademont, J.; Pedrol, E.; Grau, J.M.; UrbanoMarquez, A. Chronic fatigue syndrome: Clinical and evolutive study of 28 cases. *Med. Clin. (Barc).* **1997**, *108*, 561–565.
1425. Mistiaen, W.; Roussel, N.A.; Vissers, D.; Daenen, L.; Truijen, S.; Nijs, J. EFFECTS OF AEROBIC ENDURANCE, MUSCLE STRENGTH, AND MOTOR CONTROL EXERCISE ON PHYSICAL FITNESS AND MUSCULOSKELETAL INJURY RATE IN PREPROFESSIONAL DANCERS: AN UNCONTROLLED TRIAL. *J. Manipulative Physiol. Ther.* **2012**, *35*, 381–389.

1426. Mitchell, W.M.; Carter, W.A. The quest for effective Ebola treatment: Ebola VP35 is an evidence-based target for dsRNA drugs. *Emerg. Microbes Infect.* **2014**, *3*, e77.
1427. Miwa, K.; Fujita, M. Is small heart syndrome a "heart" disease or low output syndrome? *Int. J. Cardiol.* **2011**, *146*, 95–96.
1428. Miwa, K.; Fujita, M. Cardiovascular dysfunction with low cardiac output due to a small heart in patients with chronic fatigue syndrome. *Intern. Med.* **2009**, *48*, 1849–1854.
1429. Miwa, K.; Fujita, M. Increased oxidative stress suggested by low serum vitamin E concentrations in patients with chronic fatigue syndrome. *Int. J. Cardiol.* 2009, *136*, 238–239.
1430. Miwa, K.; Fujita, M. Small heart syndrome in patients with chronic fatigue syndrome. *Clin. Cardiol.* **2008**, *31*, 328–333.
1431. Mizuno, K.; Tanaka, M.; Fukuda, S.; Imai-Matsumura, K.; Watanabe, Y. Relationship between cognitive function and prevalence of decrease in intrinsic academic motivation in adolescents. *Behav. BRAIN Funct.* **2011**, *7*.
1432. Mo, F.; Liepold, H.; Bishop, M.; Vardy, L.; Morrison, H. *The burden of chronic fatigue syndrome (CFS) in Canada*; 2010;
1433. Mohammad, A.; Carey, J.J.; Storan, E.; Scarry, M.; Coughlan, R.J.; Lee, J.M. High Prevalence of Fibromyalgia in Patients with HFE-related Hereditary Hemochromatosis. *J. Clin. Gastroenterol.* **2013**, *47*, 559–564.
1434. Mohammed, R.H.A.; Elmakhzangy, H.I.; Gamal, A.; Mekky, F.; El Kassas, M.; Mohammed, N.; Hamid, M.A.; Esmat, G. Prevalence of rheumatologic manifestations of chronic hepatitis C virus infection among Egyptians. *Clin. Rheumatol.* **2010**, *29*, 1373–1380.
1435. Mohanty, A.F.; Muthukutty, A.; Carter, M.E.; Palmer, M.N.; Judd, J.; Helmer, D.; McAndrew, L.M.; Garvin, J.H.; Samore, M.H.; Gundlapalli, A. V Chronic multisymptom illness among female Veterans deployed to Iraq and Afghanistan. *Med. Care* **2015**, *53*, S143-8.
1436. Mojarro Práxedes, M.D.; Benjumea Pino, P. The chronic fatigue and neurasthenia in the student population [Fatiga crónica y neurastenia en población estudiantil]. *Actas Esp. Psiquiatr.* **1999**, *27*, 14–21. Work in Spanish
1437. Moldofsky, H. Role of the sleeping/waking brain in the pathogenesis of fibromyalgia, chronic fatigue syndrome, and related disorders. *Prim. Psychiatry* **2006**, *13*, 52–58.
1438. Molina, K.M.; Chen, C.-N.; Alegria, M.; Li, H. Prevalence of neurasthenia, comorbidity, and association with impairment among a nationally representative sample of US adults. *Soc. Psychiatry Psychiatr. Epidemiol.* **2012**, *47*, 1733–1744.
1439. Mommersteeg, P.M.C.; Heijnen, C.J.; Kavelaars, A.; van Doornen, L.J.P. Immune and endocrine function in burnout syndrome. *Psychosom. Med.* **2006**, *68*, 879–886.
1440. Montoya, J.G.; Anderson, J.N.; Adolphs, D.L.; Bateman, L.; Klimas, N.; Levine, S.M.; Garvert, D.W.; Kaiser, J.D. KPAX002 as a treatment for Myalgic Encephalomyelitis/Chronic Fatigue Syndrome (ME/CFS): a prospective, randomized trial. *Int. J. Clin. Exp. Med.* **2018**, *11*, 2890–2900.
1441. Mørch, K.; Hanevik, K.; Rivenes, A.C.; Bødtker, J.E.; Næss, H.; Stubhaug, B.; Wensaas, K.-A.K.-A.; Rortveit, G.; Eide, G.E.; Hausken, T.; et al. Chronic fatigue syndrome 5 years after giardiasis: Differential diagnoses, characteristics and natural course. *BMC Gastroenterol.* **2013**, *13*, 28.

1442. Morelli, V. Fatigue and Chronic Fatigue in the Elderly: Definitions, Diagnoses, and Treatments. *Clin. Geriatr. Med.* **2011**, *27*, 673–686.
1443. Morris, G.; Anderson, G.; Galecki, P.; Berk, M.; Maes, M. A narrative review on the similarities and dissimilarities between myalgic encephalomyelitis/chronic fatigue syndrome (ME/CFS) and sickness behavior. *BMC Med.* **2013**, *11*, 64.
1444. Morris, G.; Stubbs, B.; Kohler, C.A.; Walder, K.; Slyepchenko, A.; Berk, M.; Carvalho, A.F. The putative role of oxidative stress and inflammation in the pathophysiology of sleep dysfunction across neuropsychiatric disorders: Focus on chronic fatigue syndrome, bipolar disorder and multiple sclerosis. *Sleep Med. Rev.* **2018**, *41*, 255–265.
1445. Morris, J.A.; Broughton, S.J.; Wessels, Q. Microbes, molecular mimicry and molecules of mood and motivation. *Med. Hypotheses* **2016**, *87*, 40–43.
1446. Morrison, M.; Rammage, L. The irritable larynx syndrome as a central sensitivity syndrome. *Can. J. Speech-Language Pathol. Audiol.* **2010**, *34*, 284–289.
1447. Morriss, R.K.; Ahmed, M.; Wearden, A.J.; Mullis, R.; Strickland, P.; Appleby, L.; Campbell, I.T.; Pearson, D. The role of depression in pain, psychophysiological syndromes and medically unexplained symptoms associated with chronic fatigue syndrome. *J. Affect. Disord.* **1999**, *55*, 143–148.
1448. Morriss, R.K.; Wearden, A.J.; Battersby, L. The relation of sleep difficulties to fatigue, mood and disability in chronic fatigue syndrome. *J. Psychosom. Res.* **1997**, *42*, 597–605.
1449. Moss-Morris, R.; Petrie, K.J. Discriminating between chronic fatigue syndrome and depression: a cognitive analysis. *Psychol. Med.* **2001**, *31*, 469–479.
1450. Moss-Morris, R.; Spence, M. To “lump” or to “split” the functional somatic syndromes: Can infectious and emotional risk factors differentiate between the onset of chronic fatigue syndrome and irritable bowel syndrome? *Psychosom. Med.* **2006**, *68*, 463–469.
1451. Moss-Morris, R.; Spence, M.J.; Hou, R. The pathway from glandular fever to chronic fatigue syndrome: can the cognitive behavioural model provide the map? *Psychol. Med.* **2011**, *41*, 1099–1107.
1452. Moss, R.B.; Mercandetti, A.; Vojdani, A. TNF-alpha and chronic fatigue syndrome. *J. Clin. Immunol.* **1999**, *19*, 314–316.
1453. MossMorris, R.; Petrie, K.J.; Weinman, J. Functioning in chronic fatigue syndrome: Do illness perceptions play a regulatory role? *Br. J. Health Psychol.* **1996**, *1*, 15–25.
1454. Mostafalou, S.; Abdollahi, M. Pesticides and human chronic diseases: Evidences, mechanisms, and perspectives. *Toxicol. Appl. Pharmacol.* **2013**, *268*, 157–177.
1455. Mounstephen, A.; Sharpe, M. Chronic fatigue syndrome and occupational health. *Occup. Med. (Lond).* **1997**, *47*, 217–227.
1456. Mueller, T.; Jerrentrup, A.; Bauer, M.J.; Fritsch, H.W.; Schaefer, J.R. Characteristics of patients contacting a center for undiagnosed and rare diseases. *Orphanet J. Rare Dis.* **2016**, *11*.
1457. Mulak, A.; Waszczuk, E.; Paradowski, L. Irritable bowel syndrome as an interdisciplinary clinical problem. *Adv. Clin. Exp. Med.* **2008**, *17*, 667–675.
1458. Müller, B.; Müller, W. Generalized tendomyopathy (fibromyalgia) [Die generalisierte Tendomyopathie (Fibromyalgie).]. *Z. Gesamte Inn. Med.* **1991**, *46*, 361–369. Work in German
1459. Muller, H.E. Atopy and angst. *MEDIZINISCHE WELT* **1999**, *50*, 375–383.
1460. Mullin, G.E.; Lee, L.A. *Irritable bowel syndrome*; 2007;

1461. Mullins, C.; Bavendam, T.; Kirkali, Z.; Kusek, J.W. Novel research approaches for interstitial cystitis/bladder pain syndrome: thinking beyond the bladder. *Transl. Androl. Urol.* **2015**, *4*, 524–533.
1462. Munro, R. Ethnic minorities. Black and blue? ME too. *Nurs. Times* 2000, *96*, 15.
1463. Munzenmaier, D.H.; Wilentz, J.; Cowley A.W., J. Genetic, epigenetic, and mechanistic studies of temporomandibular disorders and overlapping pain conditions. *Mol. Pain* **2014**, *10*.
1464. Murdoch, J.C. The myalgic encephalomyelitis syndrome. *N. Z. Med. J.* **1989**, *102*, 372–373.
1465. Murphy, H.; Susana, A.; Patric, S. Investigation of diagnostic criteria for cancer-related fatigue syndrome in patients with advanced cancer: A feasibility study. *Palliat. Med.* **2006**, *20*, 413–418.
1466. Murray, A.M.; Toussaint, A.; Althaus, A.; Loewe, B. The challenge of diagnosing non-specific, functional, and somatoform disorders: A systematic review of barriers to diagnosis in primary care. *J. Psychosom. Res.* **2016**, *80*, 1–10.
1467. Murray, K.J. Hypermobility disorders in children and adolescents. *Best Pract. Res. Clin. Rheumatol.* **2006**, *20*, 329–351.
1468. Murthy, R.S.; Lakshminarayana, R. Is it possible to carry out high-quality epidemiological research in psychiatry with limited resources? *Curr. Opin. Psychiatry* **2005**, *18*, 565–571.
1469. Nacu, A.; Benamouzig, D. Fibromyalgia: from public issue to the patient experience. *Sante Publique (Paris).* **2010**, *22*, 551–562.
1470. Nacul, L.C.; Lacerda, E.M.; Campion, P.; Pheby, D.; Drachler, M. de L.; Leite, J.C.; Poland, F.; Howe, A.; Fayyaz, S.; Molokhia, M. The functional status and well being of people with myalgic encephalomyelitis/chronic fatigue syndrome and their carers. *BMC Public Health* **2011**, *11*, 402.
1471. Nacul, L.C.; Lacerda, E.M.; Pheby, D.; Campion, P.; Molokhia, M.; Fayyaz, S.; Leite, J.C.D.C.; Poland, F.; Howe, A.; Drachler, M.L. Prevalence of myalgic encephalomyelitis/chronic fatigue syndrome (ME/CFS) in three regions of England: a repeated cross-sectional study in primary care. *BMC Med.* **2011**, *9*, 91.
1472. Nacul, L.C.; Mudie, K.; Kingdon, C.C.; Clark, T.G.; Lacerda, E.M. Hand Grip Strength as a Clinical Biomarker for ME/CFS and Disease Severity. *Front. Neurol.* **2018**, *9*.
1473. Nadarajah, M.; Mazlan, M.; Abdul-Latif, L.; Goh, H.-T. Test-retest reliability, internal consistency and concurrent validity of Fatigue Severity Scale in measuring post-stroke fatigue. *Eur. J. Phys. Rehabil. Med.* **2017**, *53*, 703–709.
1474. Naess, H.; Nyland, M.; Hausken, T.; Follestad, I.; Nyland, H.I. Chronic fatigue syndrome after Giardia enteritis: clinical characteristics, disability and long-term sickness absence. *BMC Gastroenterol.* **2012**, *12*, 13.
1475. Naess, H.; Sundal, E.; Myhr, K.-M.; Nyland, H.I. Postinfectious and chronic fatigue syndromes: clinical experience from a tertiary-referral centre in Norway. *In Vivo* **2010**, *24*, 185–188.
1476. Nag, A.; Nag, P.K. Do the work stress factors of women telephone operators change with the shift schedules? *Int. J. Ind. Ergon.* **2004**, *33*, 449–461.
1477. Nagy-Szakal, D.; Williams, B.L.; Mishra, N.; Che, X.; Lee, B.; Bateman, L.; Klimas, N.G.; Komaroff, A.L.; Levine, S.; Montoya, J.G.; et al. Fecal metagenomic profiles in subgroups of patients with myalgic encephalomyelitis/chronic fatigue syndrome. *Microbiome* **2017**, *5*, 44.
1478. Nagy-Szakal, D.; Barupal, D.K.; Lee, B.; Che, X.; Williams, B.L.; Kahn, E.J.R.; Ukaigwe, J.E.; Bateman, L.; Klimas, N.G.; Komaroff, A.L.; et al. Insights into

myalgic encephalomyelitis/chronic fatigue syndrome phenotypes through comprehensive metabolomics. *Sci. Rep.* **2018**, *8*.
1479. Nakagawa, S.; Sugiura, M.; Sekiguchi, A.; Kotozaki, Y.; Araki, T.; Hanawa, S.; Makoto Miyauchi, C.; Sakuma, A.; Kawashima, R. Fatigue and relating to others 3 months after the 2011 Great East Japan Earthquake. *Psychiatry Res.* **2014**, *218*, 324–328.
1480. Nakao, M. [Etiology of functional somatic syndromes]. *Nihon Rinsho.* **2009**, *67*, 1661–1668. Work in Japanese
1481. Nakaya, T.; Kuratsune, H.; Kitani, T.; Ikuta, K. Demonstration on Borna disease virus in patients with chronic fatigue syndrome. *Nippon rinsho. Japanese J. Clin. Med.* **1997**, *55*, 3064–3071.
1482. Nakaya, T.; Takahashi, H.; Nakamura, Y.; Asahi, S.; Tobiume, M.; Kuratsune, H.; Kitani, T.; Yamanishi, K.; Ikuta, K. Demonstration of Borna disease virus RNA in peripheral blood mononuclear cells derived from Japanese patients with chronic fatigue syndrome. *FEBS Lett.* **1996**, *378*, 145–149.
1483. Nakaya, T.; Takahashi, H.; Nakamura, Y.; Kuratsune, H.; Kitani, T.; Machii, T.; Yamanishi, K.; Ikuta, K. Borna disease virus infection in two family clusters of patients with chronic fatigue syndrome. *Microbiol. Immunol.* **1999**, *43*, 679–689.
1484. Naring, G.W.B.; van Lankveld, W.; Geenen, R. Somatoform dissociation and traumatic experiences in patients with rheumatoid arthritis and fibromyalgia. *Clin. Exp. Rheumatol.* **2007**, *25*, 872–877.
1485. Narusyte, J.; Ropponen, A.; Alexanderson, K.; Svedberg, P. Genetic and Environmental Influences on Disability Pension Due To Mental Diagnoses: Limited Importance of Major Depression, Generalized Anxiety, and Chronic Fatigue. *Twin Res. Hum. Genet.* **2016**, *19*, 10–16.
1486. Naschitz, J.E.; Sabo, E.; Naschitz, S.; Rosner, I.; Rozenbaum, M.; Madelain, F.; Hillel, I.; Priselac, R.M.; Gaitini, L.; Eldar, S.; et al. Hemodynamics instability score in chronic fatigue syndrome and in non-chronic fatigue syndrome. *Semin. Arthritis Rheum.* **2002**, *32*, 141–148.
1487. Naschitz, J.E.; Rozenbaum, M.; Shaviv, N.; Fields, M.C.; Enis, S.; Babich, J.P.; Manor, H.; Yeshurun, D.; Sabo, E.; Rosner, I. The feeling of fatigue--fatigue severity by unidimensional versus composite questionnaires. *Behav. Med.* **2004**, *29*, 167–172.
1488. Nasralla, M.Y.; Haier, J.; Nicolson, N.L.; Nicolson, G.L. Examination of mycoplasmas in blood of 565 chronic illness patients by polymerase chain reaction. *Int. J. Med. Biol. Environ.* **2000**, *28*, 15–23.
1489. Natelson, B.H. Chronic fatigue syndrome. *J. Am. Med. Assoc.* **2001**, *285*, 2557–2559.
1490. Natelson, B.H.; Johnson, S.K.; De Luca, J.; Sisto, S.; Ellis, S.P.; Hill, N.; Bergen, M.T. Reducing heterogeneity in chronic fatigue syndrome: A comparison with depression and multiple sclerosis. *Clin. Infect. Dis.* **1995**, *21*, 1204–1210.
1491. Natelson, B.H. Chronic fatigue syndrome and fibromyalgia: a status report in 2010. *MD Advis.* **2010**, *3*, 18–25.
1492. Natelson, B.H.; Mao, X.; Stegner, A.J.; Lange, G.; Vu, D.; Blate, M.; Kang, G.; Soto, E.; Kapusuz, T.; Shungu, D.C. Multimodal and simultaneous assessments of brain and spinal fluid abnormalities in chronic fatigue syndrome and the effects of psychiatric comorbidity. *J. Neurol. Sci.* **2017**, *375*, 411–416.
1493. Natelson, B.H.; Weaver, S.A.; Tseng, C.-L.; Ottenweller, J.E. Spinal fluid abnormalities in patients with chronic fatigue syndrome. *Clin. Diagn. Lab. Immunol.* **2005**, *12*, 52–55.

1494. Nater, U.M.; Jones, J.F.; Lin, J.-M.S.; Maloney, E.; Reeves, W.C.; Heim, C. Personality features and personality disorders in chronic fatigue Syndrome: A population-based study. *Psychother. Psychosom.* **2010**, *79*, 312–318.
1495. Nater, U.M.; Lin, J.-M.; Maloney, E.M.; Jones, J.F.; Tian, H.; Heim, C.; Raison, C.L.; Boneva, R.S.; Reeves, W.C. The authors reply. *Psychosom. Med.* **2010**, *72*, 507–509.
1496. Nater, U.M.; Heim, C.M.; Raison, C. Chronic fatigue syndrome. *Handb. Clin. Neurol.* **2012**, *106*, 573–587.
1497. Nater, U.M.; Lin, J.-M.S.; Maloney, E.M.; Jones, J.F.; Tian, H.; Boneva, R.S.; Raison, C.L.; Reeves, W.C.; Heim, C. Psychiatric comorbidity in persons with chronic fatigue syndrome identified from the Georgia population. *Psychosom. Med.* **2009**, *71*, 557–565.
1498. Nater, U.M.; Maloney, E.; Heim, C.; Reeves, W.C. Cumulative life stress in chronic fatigue syndrome. *Psychiatry Res.* **2011**, *189*, 318–320.
1499. Nater, U.M.; Maloney, E.; Lin, J.-M.S.; Heim, C.; Reeves, W.C. Coping styles in chronic fatigue syndrome: findings from a population-based study. *Psychother. Psychosom.* 2012, *81*, 127–129.
1500. Nater, U.M.; Wagner, D.; Solomon, L.; Jones, J.F.; Unger, E.R.; Papanicolaou, D.A.; Reeves, W.C.; Heim, C. Coping styles in people with chronic fatigue syndrome identified from the general population of Wichita, KS. *J. Psychosom. Res.* **2006**, *60*, 567–573.
1501. Nawab, S.S.; Miller, C.S.; Dale, J.K.; Greenberg, B.D.; Friedman, T.C.; Chrousos, G.P.; Straus, S.E.; Rosenthal, N.E. Self-reported sensitivity to chemical exposures in five clinical populations and healthy controls. *PSYCHIATRY Res.* **2000**, *95*, 67–74.
1502. Nazir, F.S.; Lees, K.R.; Bone, I. Clinical features associated with medically unexplained stroke-like symptoms presenting to an acute stroke unit. *Eur. J. Neurol.* **2005**, *12*, 81–85.
1503. Neblett, R.; Cohen, H.; Choi, Y.; Hartzell, M.M.; Williams, M.; Mayer, T.G.; Gatchel, R.J. The Central Sensitization Inventory (CSI): Establishing Clinically Significant Values for Identifying Central Sensitivity Syndromes in an Outpatient Chronic Pain Sample. *J. PAIN* **2013**, *14*, 438–445.
1504. Neblett, R.; Hartzell, M.M.; Mayer, T.G.; Cohen, H.; Gatchel, R.J. Establishing Clinically Relevant Severity Levels for the Central Sensitization Inventory. *PAIN Pract.* **2017**, *17*, 166–175.
1505. Nederhof, E.; Lemmink, K.A.P.M.; Visscher, C.; Meeusen, R.; Mulder, T. Psychomotor speed: possibly a new marker for overtraining syndrome. *Sports Med.* **2006**, *36*, 817–828.
1506. Nelsen Jr., D.A. Gluten-sensitive enteropathy (celiac disease): More common than you think. *Am. Fam. Physician* **2002**, *66*, 2259–2266+2269.
1507. Nelsen, D.A.; Craig, T. Differential diagnosis for chronic fatigue syndrome [1] (multiple letters). *Am. Fam. Physician* **2003**, *67*, 252.
1508. Nelson, M.; Ogden, J. An exploration of food intolerance in the primary care setting: The general practitioner's experience. *Soc. Sci. Med.* **2008**, *67*, 1038–1045.
1509. Neu, D.; Hoffmann, G.; Moutrier, R.; Verbanck, P.; Linkowski, P.; Le Bon, O. Are patients with chronic fatigue syndrome just "tired" or also "sleepy"? *J. Sleep Res.* **2008**, *17*, 427–431.
1510. Neu, D.; Mairesse, O.; Hoffmann, G.; Dris, A.; Lambrecht, L.J.; Linkowski, P.; Verbanck, P.; Le Bon, O. Sleep quality perception in the chronic fatigue syndrome:

correlations with sleep efficiency, affective symptoms and intensity of fatigue. *Neuropsychobiology* **2007**, *56*, 40–46.
1511. Neu, D.; Mairesse, O.; Montana, X.; Gilson, M.; Corazza, F.; Lefevre, N.; Linkowski, P.; Le Bon, O.; Verbanck, P. Dimensions of pure chronic fatigue: psychophysical, cognitive and biological correlates in the chronic fatigue syndrome. *Eur. J. Appl. Physiol.* **2014**, *114*, 1841–1851.
1512. Neu, D.; Mairesse, O.; Verbanck, P.; Linkowski, P.; Le Bon, O. Non-REM sleep EEG power distribution in fatigue and sleepiness. *J. Psychosom. Res.* **2014**, *76*, 286–291.
1513. Neutra, R.R. Some preliminary thoughts on the potential contribution of epidemiology to the question of multiple chemical sensitivity. *Public Health Rev.* **1994**, *22*, 271–278.
1514. Newland, P.; Starkweather, A.; Sorenson, M. Central fatigue in multiple sclerosis: a review of the literature. *J. Spinal Cord Med.* **2016**, *39*, 386–399.
1515. Newton, J.L.; Okonkwo, O.; Sutcliffe, K.; Seth, A.; Shin, J.; Jones, D.E.J. Symptoms of autonomic dysfunction in chronic fatigue syndrome. *QJM* **2007**, *100*, 519–526.
1516. Newton, J.L.; Pairman, J.; Hallsworth, K.; Moore, S.; Plötz, T.; Trenell, M.I. Physical activity intensity but not sedentary activity is reduced in chronic fatigue syndrome and is associated with autonomic regulation. *QJM* **2011**, *104*, 681–687.
1517. Newton, J.L.; Frith, J.; Powell, D.; Hackett, K.; Wilton, K.; Bowman, S.; Price, E.; Pease, C.; Andrews, J.; Emery, P.; et al. Autonomic symptoms are common and are associated with overall symptom burden and disease activity in primary Sjogren's syndrome. *Ann. Rheum. Dis.* **2012**, *71*, 1973–1979.
1518. Newton, J.L.; Sheth, A.; Shin, J.; Pairman, J.; Wilton, K.; Burt, J.A.; Jones, D.E.J. Lower ambulatory blood pressure in chronic fatigue syndrome. *Psychosom. Med.* **2009**, *71*, 361–365.
1519. Ng, B.-Y.; Lim, C.C.T.; Yeoh, A.; Lee, W.L. Neuropsychiatric sequelae of Nipah virus encephalitis. *J. Neuropsychiatry Clin. Neurosci.* **2004**, *16*, 500–504.
1520. Ng, S.Y. Hair calcium and magnesium levels in patients with fibromyalgia: A case center study. *J. Manipulative Physiol. Ther.* **1999**, *22*, 586–593.
1521. Nguyen, C.B.; Alsoe, L.; Lindvall, J.M.; Sulheim, D.; Fagermoen, E.; Winger, A.; Kaarbo, M.; Nilsen, H.; Wyller, V.B. Whole blood gene expression in adolescent chronic fatigue syndrome: an exploratory cross-sectional study suggesting altered B cell differentiation and survival. *J. Transl. Med.* **2017**, *15*, 102.
1522. Nguyen, R.H.N.; Ecklund, A.M.; Maclehose, R.F.; Veasley, C.; Harlow, B.L. Co-morbid pain conditions and feelings of invalidation and isolation among women with vulvodynia. *Psychol. Health Med.* **2012**, *17*, 589–598.
1523. Nguyen, R.H.N.; Veasley, C.; Smolenski, D. Latent class analysis of comorbidity patterns among women with generalized and localized vulvodynia: preliminary findings. *J. Pain Res.* **2013**, *6*, 303–309.
1524. Niblett, S.H.; King, K.E.; Dunstan, R.H.; Clifton-Bligh, P.; Hoskin, L.A.; Roberts, T.K.; Fulcher, G.R.; McGregor, N.R.; Dunsmore, J.C.; Butt, H.L.; et al. Hematologic and urinary excretion anomalies in patients with chronic fatigue syndrome. *Exp. Biol. Med. (Maywood).* **2007**, *232*, 1041–1049.
1525. Nickel, J.C.; Tripp, D.A.; Pontari, M.; Moldwin, R.; Mayer, R.; Carr, L.K.; Doggweiler, R.; Yang, C.C.; Mishra, N.; Nordling, J. Interstitial cystitis/painful bladder syndrome and associated medical conditions with an emphasis on irritable bowel syndrome, fibromyalgia and chronic fatigue syndrome. *J. Urol.* **2010**, *184*, 1358–1363.

1526. Nicolas, M.E.O.; Krause, P.K.; Gibson, L.E.; Murray, J.A. Dermatitis herpetiformis. *Int. J. Dermatol.* **2003**, *42*, 588–600.
1527. Nicolson, G.L.; Gan, R.; Haier, J. Evidence for Brucella spp. and Mycoplasma spp. co-infections in blood of chronic fatigue syndrome patients. *J. Chronic Fatigue Syndr.* **2004**, *12*, 5–17.
1528. Nicolson, G.L.; Gan, R.; Haier, J. Multiple co-infections (Mycoplasma, Chlamydia, human herpes virus-6) in blood of chronic fatigue syndrome patients: Association with signs and symptoms. *APMIS* **2003**, *111*, 557–566.
1529. Nicolson, G.L.; Nasralla, M.Y.; De Meirleir, K.; Gan, R.; Haier, J. Evidence for bacterial (Mycoplasma, Chlamydia) and viral (HHV-6) co-infections in Chronic Fatigue Syndrome patients. *J. Chronic Fatigue Syndr.* **2003**, *11*, 7–19.
1530. Nicolson, G.L.; Nasralla, M.Y.; Nicolson, N.L.; Haier, J. High prevalence of mycoplasma infections in symptomatic (Chronic Fatigue Syndrome) family members of mycoplasma-positive Gulf War illness patients. *J. Chronic Fatigue Syndr.* **2003**, *11*, 21–36.
1531. Nicolson, G.L.; Nicolson, N.L.; Haier, J. Chronic fatigue syndrome patients subsequently diagnosed with lyme disease borrelia burgdorferi: Evidence for mycoplasma species coinfections. *J. Chronic Fatigue Syndr.* **2008**, *14*, 5–17.
1532. Nicolson, G.L.; Gan, R.; Nicolson, N.L.; Haier, J. Evidence for mycoplasma ssp., Chlamydia pneumoniae, and human herpes virus-6 coinfections in the blood of patients with autistic spectrum disorders. *J. Neurosci. Res.* **2007**, *85*, 1143–1148.
1533. Nierenberg, A.A.; Sonino, N. From clinical observations to clinimetrics: A tribute to. *Psychother. Psychosom.* **2004**, *73*, 131–133.
1534. Nijhof, L.N.; Nijhof, S.L.; Bleijenberg, G.; Stellato, R.K.; Kimpen, J.L.L.; Hulshoff Pol, H.E.; van de Putte, E.M. The impact of chronic fatigue syndrome on cognitive functioning in adolescents. *Eur. J. Pediatr.* **2016**, *175*, 245–252.
1535. Nijhof, S.L.; Bleijenberg, G.; Uiterwaal, C.S.P.M.; Kimpen, J.L.L.; van de Putte, E.M. Effectiveness of internet-based cognitive behavioural treatment for adolescents with chronic fatigue syndrome (FITNET): a randomised controlled trial. *Lancet* **2012**, *379*, 1412–1418.
1536. Nijhof, S.L.; Maijer, K.; Bleijenberg, G.; Uiterwaal, C.S.P.M.; Kimpen, J.L.L.; van de Putte, E.M. Adolescent chronic fatigue syndrome: prevalence, incidence, and morbidity. *Pediatrics* **2011**, *127*, e1169-75.
1537. Nijhof, S.L.; Priesterbach, L.P.; Uiterwaal, C.S.P.M.; Bleijenberg, G.; Kimpen, J.L.L.; van de Putte, E.M. Internet-Based Therapy for Adolescents With Chronic Fatigue Syndrome: Long-term Follow-up. *Pediatrics* **2013**, *131*, E1788–E1795.
1538. Nijs, J. Generalized joint hypermobility: An issue in fibromyalgia and chronic fatigue syndrome? *J. Bodyw. Mov. Ther.* **2005**, *9*, 310–317.
1539. Nijs, J.; Adriaens, J.; Schuermans, D.; Buyl, R.; Vincken, W. Breathing retraining in patients with chronic fatigue syndrome: A pilot study. *Physiother. Theory Pract.* **2008**, *24*, 83–94.
1540. Nijs, J.; De Meirleir, K.; Coomans, D.; De Becker, P.; Nicolson, G.L. Deregulation of the 2,5A synthetase RNase L antiviral pathway by Mycoplasma spp. in subsets of Chronic Fatigue Syndrome. *J. Chronic Fatigue Syndr.* **2003**, *11*, 37–50.
1541. Nijs, J.; De Meirleir, K.; Truyen, S. Hypermobility in patients with chronic fatigue syndrome: Preliminary observations. *J. Musculoskelet. Pain* **2004**, *12*, 9–17.
1542. Nijs, J.; Nees, A.; Paul, L.; De Kooning, M.; Ickmans, K.; Meeus, M.; Van Oosterwijck, J. Altered immune response to exercise in patients with chronic fatigue syndrome/myalgic encephalomyelitis: A systematic literature review. *Exerc. Immunol. Rev.* **2014**, *20*, 94–116.

1543. Nijs, J.; Aerts, A.; De Meirleir, K. Generalized joint hypermobility is more common in chronic fatigue syndrome than in healthy control subjects. *J. Manipulative Physiol. Ther.* **2006**, *29*, 32–39. Proceedings of the International Conference on Chiropractic Research in Sydney, Australia.
1544. Nijs, J.; Meeus, M.; McGregor, N.R.; Meeusen, R.; de Schutter, G.; van Hoof, E.; de Meirleir, K. Chronic fatigue syndrome: exercise performance related to immune dysfunction. *Med. Sci. Sports Exerc.* **2005**, *37*, 1647–1654.
1545. Nijs, J.; Nicolson, G.L.; De Becker, P.; Coomans, D.; De Meirleir, K. High prevalence of Mycoplasma infections among European chronic fatigue syndrome patients. Examination of four Mycoplasma species in blood of chronic fatigue syndrome patients. *FEMS Immunol. Med. Microbiol.* **2002**, *34*, 209–214.
1546. Nijs, J.; Roussel, N.; Van Oosterwijck, J.; De Kooning, M.; Ickmans, K.; Struyf, F.; Meeus, M.; Lundberg, M. Fear of movement and avoidance behaviour toward physical activity in chronic-fatigue syndrome and fibromyalgia: state of the art and implications for clinical practice. *Clin. Rheumatol.* **2013**, *32*, 1121–1129.
1547. Nijs, J.; Van de Putte, K.; Louckx, F.; De Meirleir, K. Employment status in chronic fatigue syndrome. A cross-sectional study examining the value of exercise testing and self-reported measures for the assessment of employment status. *Clin. Rehabil.* **2005**, *19*, 895–899.
1548. Nijs, J.; Van de Putte, K.; Louckx, F.; Truijen, S.; De Meirleir, K. Exercise performance and chronic pain in chronic fatigue syndrome: the role of pain catastrophizing. *Pain Med.* **2008**, *9*, 1164–1172.
1549. Nijs, J.; Vanherberghen, K.; Duquet, W.; De Meirleir, K. Chronic fatigue syndrome: lack of association between pain-related fear of movement and exercise capacity and disability. *Phys. Ther.* **2004**, *84*, 696–705.
1550. Nimnuan, C.; Rabe-Hesketh, S.; Wessely, S.; Hotopf, M. How many functional somatic syndromes? *J. Psychosom. Res.* **2001**, *51*, 549–557.
1551. Nisenbaum, R.; Reyes, M.; Mawle, A.C.; Reeves, W.C. Factor analysis of unexplained severe fatigue and interrelated symptoms: overlap with criteria for chronic fatigue syndrome. *Am. J. Epidemiol.* **1998**, *148*, 72–77.
1552. Nishikai, M. Chronic Fatigue Syndrome : Current Perspectives. *IRYO - Japanese J. Natl. Med. Serv.* **1996**, *50*, 747–752.
1553. Nishikai, M. [Chronic fatigue syndrome--study of 51 cases treated at the Second Tokyo National Hospital]. *Nihon Rinsho.* **1992**, *50*, 2641–2647.
1554. Nishikai, M.; Kosaka, S.; Tan, E.M.; vonMikecz, A.; Konstantinov, K.; Gerace, L.; Buchwald, D.S. Incidence of antinuclear antibodies in Japanese patients with chronic fatigue syndrome - Reply. *ARTHRITIS Rheum.* **1997**, *40*, 2096–2097.
1555. Nishikai, M. [Antinuclear antibodies in patients with chronic fatigue syndrome]. *Nihon Rinsho.* **2007**, *65*, 1067–1070. Work in Japanese
1556. Nix, W.A. The impact of multiple chemical sensitivities and of other environmental associated disorders - fatigued and hypersensitive. *ALLERGOLOGIE* **1999**, *22*, 736–743.
1557. Nix, W.A.; Egle, U.T. The chronic fatigue syndrome. *AKTUELLE Neurol.* **1998**, *25*, 6–12.
1558. Njoku, M.G.C.; Jason, L.A.; Torres-Harding, S.R. The relationships among coping styles and fatigue in an ethnically diverse sample. *Ethn. Health* **2005**, *10*, 263–278.
1559. Njoku, M.G.C.; Jason, L.A.; Torres-Harding, S.R. The prevalence of chronic fatigue syndrome in Nigeria. *J. Health Psychol.* **2007**, *12*, 461–474.
1560. Nogue Xarau, S.; Alarcon Romay, M.; Martinez Martinez, J.-M.; Delclos Clanchet, J.; Rovira Prat, E.; Fernandez Sola, J. [Multiple chemical sensitivity:

epidemiological, clinical and prognostic differences between occupational and non-occupational cases]. *Med. Clin. (Barc).* **2010**, *135*, 52–58. Work in Spanish
1561. Nogue Xarau, S.; Duenas Laita, A.; Ferrer Dufol, A.; Fernandez Sola, J.; M, G.T.S.Q. Multiple chemical sensitivity. *Med. Clin. (Barc).* **2011**, *136*, 683–687.
1562. Nordin, M.; Nordin, S. Sleep and sleepiness in environmental intolerances: a population-based study. *Sleep Med.* **2016**, *24*, 1–9.
1563. Norris, T.; Hawton, K.; Hamilton-Shield, J.; Crawley, E. Obesity in adolescents with chronic fatigue syndrome: an observational study. *Arch. Dis. Child.* **2017**, *102*, 35–39.
1564. Norris, T.; Collin, S.M.; Tilling, K.; Nuevo, R.; Stansfeld, S.A.; Sterne, J.A.; Heron, J.; Crawley, E. Natural course of chronic fatigue syndrome/myalgic encephalomyelitis in adolescents. *Arch. Dis. Child.* **2017**, *102*, 522–528.
1565. Norris, T.; Deere, K.; Tobias, J.H.; Crawley, E. Chronic Fatigue Syndrome and Chronic Widespread Pain in Adolescence: Population Birth Cohort Study. *J. Pain* **2017**, *18*, 285–294.
1566. Nowicki, M.J.; Balistreri, W.F. The hepatitis C virus: Identification, epidemiology, and clinical controversies. *J. Pediatr. Gastroenterol. Nutr.* **1995**, *20*, 248–274.
1567. Nyland, M.; Naess, H.; Birkeland, J.S.; Nyland, H. Longitudinal follow-up of employment status in patients with chronic fatigue syndrome after mononucleosis. *BMJ Open* **2014**, *4*, e005798.
1568. O'Malley, P.G.; Jackson, J.L.; Kroenke, K.; Yoon, I.K.; Hornstein, E.; Dennis, G.J. The value of screening for psychiatric disorders in rheumatology referrals. *Arch. Intern. Med.* **1998**, *158*, 2357–2362.
1569. Oberg, K. State of the art and future prospects in the management of neuroendocrine tumors. *Q. J. Nucl. Med.* **2000**, *44*, 3–12.
1570. Ohayon, M.M. Prevalence and correlates of nonrestorative sleep complaints. *Arch. Intern. Med.* **2005**, *165*, 35–41.
1571. Okamoto, L.E.; RAJ, S.R.; Peltier, A.; Gamboa, A.; Shibao, C.; Diedrich, A.; Black, B.K.; Robertson, D.; Biaggioni, I. Neurohumoral and haemodynamic profile in postural tachycardia and chronic fatigue syndromes. *Clin. Sci.* **2012**, *122*, 183–192.
1572. Oken, B.S.; Flegal, K.; Zajdel, D.; Kishiyama, S.S.; Lovera, J.; Bagert, B.; Bourdette, D.N. Cognition and fatigue in multiple sclerosis: Potential effects of medications with central nervous system activity. *J. Rehabil. Res. Dev.* **2006**, *43*, 83–90.
1573. Oken, M.M.; Kyle, R.A.; Greipp, P.R.; Kay, N.E.; Tsiatis, A.; Gregory, S.A.; Spiegel, R.J.; O'connell, M.J. Complete remission induction with combined VBMCP chemotherapy and interferon (rIFNα2b) in patients with multiple myeloma. *Leuk. Lymphoma* **1996**, *20*, 447–452.
1574. Okuyama, T.; Tanaka, K.; Akechi, T.; Kugaya, A.; Okamura, H.; Nishiwaki, Y.; Hosaka, T.; Uchitomi, Y. Fatigue in ambulatory patients with advanced lung cancer: prevalence, correlated factors, and screening. *J. Pain Symptom Manage.* **2001**, *22*, 554–564.
1575. Olson, K.; Zimka, O.; Stein, E. The Nature of Fatigue in Chronic Fatigue Syndrome. *Qual. Health Res.* **2015**, *25*, 1410–1422.
1576. Oltra, E.; Garcia-Escudero, M.; Vicente Mena-Duran, A.; Monsalve, V.; Cerda-Olmedo, G. Lack of evidence for retroviral infections formerly related to chronic fatigue in Spanish Fibromyalgia patients. *Virol. J.* **2013**, *10*.
1577. Oosterwijck, J. Van; Marusic, U.; De Wandele, I.; Paul, L.; Meeus, M.; Moorkens, G.; Lambrecht, L.; Danneels, L.; Nijs, J. The Role of Autonomic Function in

Exercise-induced Endogenous Analgesia: A Case-control Study in Myalgic Encephalomyelitis/Chronic Fatigue Syndrome and Healthy People. *Pain Physician* **2017**, *20*, E389–E399.
1578. Ortega-Hernandez, O.-D.; Cuccia, M.; Bozzini, S.; Bassi, N.; Moscavitch, S.; Diaz-Gallo, L.-M.; Blank, M.; Agmon-Levin, N.; Shoenfeld, Y. Autoantibodies, polymorphisms in the serotonin pathway, and human leukocyte antigen class II alleles in chronic fatigue syndrome: are they associated with age at onset and specific symptoms? *Ann. N. Y. Acad. Sci.* **2009**, *1173*, 589–599.
1579. Ortolani, C.; Bruijnzeel-Koomen, C.; Bengtsson, U.; Bindslev-Jensen, C.; Björkstén, B.; Høst, A.; Ispano, M.; Jarish, R.; Madsen, C.; Nekam, K.; et al. Controversial aspects of adverse reactions to food. *Allergy Eur. J. Allergy Clin. Immunol.* **1999**, *54*, 27–45.
1580. Osoba, T.; Pheby, D.; Gray, S.; Nacul, L. The development of an epidemiological definition for myalgic encephalomyelitis/chronic fatigue syndrome. *J. Chronic Fatigue Syndr.* **2008**, *14*, 61–84.
1581. Overman, C.L.; Kool, M.B.; Da Silva, J.A.P.; Geenen, R. The prevalence of severe fatigue in rheumatic diseases: an international study. *Clin. Rheumatol.* **2016**, *35*, 409–415.
1582. Öztürk, M.A.; Tezer, M.S.; Özbalkan, Z.; Ünal, A.; Güçlü Güven, D.; Karaarslan, Y. Fibromyalgia syndrome in patients with chronic sinusitis. *Turkish J. Med. Sci.* **2009**, *39*, 377–380.
1583. Pae, C.-U.; Luyten, P.; Marks, D.M.; Han, C.; Park, S.-H.; Patkar, A.A.; Masand, P.S.; Van Houdenhove, B. The relationship between fibromyalgia and major depressive disorder: a comprehensive review. *Curr. Med. Res. Opin.* **2008**, *24*, 2359–2371.
1584. Pagani, M.; Lucini, D. Chronic fatigue syndrome: A hypothesis focusing on the autonomic nervous system. *Clin. Sci.* **1999**, *96*, 117–125.
1585. Page, L.A.; Petrie, K.J.; Wessely, S.C. Psychosocial responses to environmental incidents: A review and a proposed typology. *J. Psychosom. Res.* **2006**, *60*, 413–422. Proceedings of the Conference on Somatoform Disorders in London, UK.
1586. Pailhez, G.; Bulbena, A.; Fullana, M.A.; Castaño, J. Anxiety disorders and joint hypermobility syndrome: the role of collagen tissue. *Gen. Hosp. Psychiatry* **2009**, *31*, 299.
1587. Palacios, N.; Fitzgerald, K.C.; Komaroff, A.L.; Ascherio, A. Incidence of myalgic encephalomyelitis/chronic fatigue syndrome in a large prospective cohort of U.S. nurses. *FATIGUE-BIOMEDICINE Heal. Behav.* **2017**, *5*, 159–166.
1588. Palafox, N.A.; Buenconsejo-Lum, L.E. Ciguatera fish poisoning: Review of clinical manifestations. *J. Toxicol. Rev.* **2001**, *20*, 141–160.
1589. Pall, M.L.; Bedient, S.A. The NO/ONOO- cycle as the etiological mechanism of tinnitus. *Int. Tinnitus J.* **2007**, *13*, 99–104.
1590. Pan, J.C.; Bressler, D.N. Fatigue in Rheumatologic Diseases. *Phys. Med. Rehabil. Clin. N. Am.* **2009**, *20*, 373–387.
1591. Pantry, S.N.; Medveczky, M.M.; Arbuckle, J.H.; Luka, J.; Montoya, J.G.; Hu, J.; Renne, R.; Peterson, D.; Pritchett, J.C.; Ablashi, D. V; et al. Persistent human herpesvirus-6 infection in patients with an inherited form of the virus. *J. Med. Virol.* **2013**, *85*, 1940–1946.
1592. Papadopoulos, A.S.; Cleare, A.J. Hypothalamic-pituitary-adrenal axis dysfunction in chronic fatigue syndrome. *Nat. Rev. Endocrinol.* **2011**, *8*, 22–32.
1593. Papadopoulos, A.; Ebrecht, M.; Roberts, A.D.L.; Poon, L.; Rohleder, N.; Cleare, A.J. Glucocorticoid receptor mediated negative feedback in chronic fatigue

syndrome using the low dose (0.5 mg) dexamethasone suppression test. *J. Affect. Disord.* **2009**, *112*, 289–294.
1594. Papanicolaou, D.A.; Amsterdam, J.D.; Levine, S.; McCann, S.M.; Moore, R.C.; Newbrand, C.H.; Allen, G.; Nisenbaum, R.; Pfaff, D.W.; Tsokos, G.C.; et al. Neuroendocrine Aspects of Chronic Fatigue Syndrome. *Neuroimmunomodulation* **2004**, *11*, 65–74.
1595. Paralikar, V.; Agashe, M.; Sarmukaddam, S.; Deshpande, S.; Goyal, V.; Weiss, M.G. Cultural epidemiology of neurasthenia spectrum disorders in four general hospital outpatient clinics of urban Pune, India. *Transcult. Psychiatry* **2011**, *48*, 257–283.
1596. Paralikar, V.; Agashe, M.; Oke, M.; Dabholkar, H.; Abouihia, A.; Weiss, M.G. Prevalence of clinically significant functional fatigue or weakness in specialty outpatient clinics of Pune, India. *J. Indian Med. Assoc.* **2007**, *105*, 424–426,428,430.
1597. Paralikar, V.; Sarmukaddam, S.; Agashe, M.; Weiss, M.G. Diagnostic concordance of neurasthenia spectrum disorders in Pune, India. *Soc. Psychiatry Psychiatr. Epidemiol.* **2007**, *42*, 561–572.
1598. Park, H.Y.; Jeon, H.J.; Bang, Y.R.; Yoon, I.-Y. Multidimensional Comparison of Cancer-Related Fatigue and Chronic Fatigue Syndrome: The Role of Psychophysiological Markers. *PSYCHIATRY Investig.* **2019**, *16*, 71–79.
1599. Park, J.; Gilmour, H. Medically unexplained physical symptoms (MUPS) among adults in Canada: Comorbidity, health care use and employment. *Heal. Reports* **2017**, *28*, 3–8.
1600. Park, J.; Knudson, S. Medically unexplained physical symptoms. *Heal. reports* **2007**, *18*, 43–47.
1601. Park, S.; Hong, J.P.; Bae, J.N.; Cho, S.-J.; Lee, D.-W.; Lee, J.-Y.; Chang, S.M.; Jeon, H.J.; Hahm, B.-J.; Lee, Y.M.; et al. Impact of childhood exposure to psychological trauma on the risk of psychiatric disorders and somatic discomfort: Single vs. multiple types of psychological trauma. *PSYCHIATRY Res.* **2014**, *219*, 443–449.
1602. Parslow, R.M.; Harris, S.; Broughton, J.; Alattas, A.; Crawley, E.; Haywood, K.; Shaw, A. Children's experiences of chronic fatigue syndrome/myalgic encephalomyelitis (CFS/ME): a systematic review and meta-ethnography of qualitative studies. *BMJ Open* **2017**, *7*.
1603. Pastores, G.M.; Giraldo, P.; Chérin, P.; Mehta, A. Goal-oriented therapy with miglustat in Gaucher disease. *Curr. Med. Res. Opin.* **2009**, *25*, 23–37.
1604. Patarca-Montero, R. Multidisciplinary innovations in research, theory and clinical practice: Editorial. *J. Chronic Fatigue Syndr.* **2000**, *7*, 1.
1605. Patarca-Montero, R.; Fletcher, M.A. The Paul-Bunnell heterophile antibody determinant in Epstein-Barr virus-associated disease. *J. Chronic Fatigue Syndr.* **2002**, *10*, 51–86.
1606. Patel, M.X.; Smith, D.G.; Chalder, T.; Wessely, S. Chronic fatigue syndrome in children: a cross sectional survey. *Arch. Dis. Child.* **2003**, *88*, 894–898.
1607. Patel, V.; Kirkwood, B.; Weiss, H.; Pednekar, S.; Fernandes, J.; Pereira, B.; Upadhye, M.; Mabey, D. Chronic fatigue in developing countries: Population based survey of women in India. *Br. Med. J.* **2005**, *330*, 1190–1193.
1608. Patnaik, M.; Komaroff, A.L.; Conley, E.; Ojo-Amaize, E.A.; Peter, J.B. Prevalence of igm antibodies to human herpesvirus 6 early antigen (p41/38) in patients with chronic fatigue syndrome. *J. Infect. Dis.* **1995**, *172*, 1643.

1609. PATNAIK, M.; OIOAMAIZE, E.; LIN, H.C.; KOMAROFF, A. INCREASED PREVALENCE OF IGM ANTIBODIES TO HHV-6 EARLY ANTIGEN IN CHRONIC FATIGUE SYNDROME. *ARTHRITIS Rheum.* **1993**, *36*, S203.
1610. Patrick, D.M.; Miller, R.R.; Gardy, J.L.; Parker, S.M.; Morshed, M.G.; Steiner, T.S.; Singer, J.; Shojania, K.; Tang, P. Lyme Disease Diagnosed by Alternative Methods: A Phenotype Similar to That of Chronic Fatigue Syndrome. *Clin. Infect. Dis.* **2015**, *61*, 1084–1091.
1611. Patten, S.B.; Beck, C.A.; Kassam, A.; Williams, J.V.A.; Barbui, C.; Metz, L.M. Long-term medical conditions and major depression: strength of association for specific conditions in the general population. *Can. J. Psychiatry.* **2005**, *50*, 195–202.
1612. Patterson, R.E.; Frank, L.L.; Kristal, A.R.; White, E. A comprehensive examination of health conditions associated with obesity in older adults. *Am. J. Prev. Med.* **2004**, *27*, 385–390.
1613. Paulin, J.; Andersson, L.; Nordin, S. Characteristics of hyperacusis in the general population. *Noise Heal.* **2016**, *18*, 178–184.
1614. Paulley, J.W. Hyperventilation. *Recenti Prog. Med.* **1990**, *81*, 594–600.
1615. Pawlikowska, T.; Chalder, T.; Hirsch, S.R.; Wallace, P.; Wright, D.J.; Wessely, S.C. Population based study of fatigue and psychological distress. *BMJ* **1994**, *308*, 763–766.
1616. Payne, B.A.I.; Hateley, C.L.; Ong, E.L.C.; Premchand, N.; Schmid, M.L.; Schwab, U.; Newton, J.L.; Price, D.A. HIV-associated fatigue in the era of highly active antiretroviral therapy: novel biological mechanisms? *HIV Med.* **2013**, *14*, 247–251.
1617. Pazderka-Robinson, H.; Morrison, J.W.; Flor-Henry, P. Electrodermal dissociation of chronic fatigue and depression: Evidence for distinct physiological mechanisms. *Int. J. Psychophysiol.* **2004**, *53*, 171–182.
1618. Peachey, E. Myalgic encephalomyelitis. Myth, mystery or misunderstood? *Pract. Midwife* **2001**, *4*, 29–31.
1619. Pearce, J.M.S. The enigma of chronic fatigue. *Eur. Neurol.* **2006**, *56*, 31–36.
1620. Pearson, N.A.; Packham, J.C.; Parsons, H.; Haywood, K.L. Quality and acceptability of patient-reported outcome measures used to assess fatigue in axial spondyloarthritis (axSpA): a systematic review (protocol). *Syst. Rev.* **2018**, *7*.
1621. Pedersen, M.; Ekstedt, M.; Smastuen, M.C.; Wyller, V.B.; Sulheim, D.; Fagermoen, E.; Winger, A.; Pedersen, E.; Hrubos-Strom, H. Sleep-wake rhythm disturbances and perceived sleep in adolescent chronic fatigue syndrome. *J. Sleep Res.* **2017**, *26*, 595–601.
1622. Pedersen, N.L.; Lichtenstein, P.; Svedberg, P. The Swedish Twin Registry in the third millennium. *Twin Res.* **2002**, *5*, 427–432.
1623. Pejovic, S.; Natelson, B.H.; Basta, M.; Fernandez-Mendoza, J.; Mahr, F.; Vgontzas, A.N. Chronic fatigue syndrome and fibromyalgia in diagnosed sleep disorders: a further test of the "unitary" hypothesis. *BMC Neurol.* **2015**, *15*, 53.
1624. Pelletier, J.; Benoit, N.; Montreuil, M.; Habib, M. Cognitive and emotional disorders in multiple sclerosis. Can a suitable management strategy be envisaged? *Pathol. Biol.* **2000**, *48*, 121–131.
1625. Penfold, S.; Denis, E.S.; Mazhar, M.N. The association between borderline personality disorder, fibromyalgia and chronic fatigue syndrome: Systematic review. *BJPsych Open* **2016**, *2*, 275–279.
1626. Peres, M.F.P. Fibromyalgia, fatigue, and headache disorders. *Curr. Neurol. Neurosci. Rep.* **2003**, *3*, 97–103.

1627. Peres, M.F.P.; Zukerman, E.; Young, W.B.; Silberstein, S.D. Fatigue in chronic migraine patients. *Cephalalgia* **2002**, *22*, 720–724.
1628. Perez, A.O. Multiple chemical sensitivity, a disease commonly missed. *Med. Clin. (Barc).* **2005**, *125*, 257–262.
1629. Perrin, R.N.; Edwards, J.; Hartley, P. An evaluation of the effectiveness of osteopathic treatment on symptoms associated with myalgic encephalomyelitis. A preliminary report. *J. Med. Eng. Technol.* **1998**, *22*, 1–13.
1630. Perruccio, A. V; Power, J.D.; Badley, E.M. The relative impact of 13 chronic conditions across three different outcomes. *J. Epidemiol. Community Health* **2007**, *61*, 1056–1061.
1631. Perski, A.; Grossi, G.; Evengard, B.; Blomkvist, V.; Yilbar, B.; Orth-Gomer, K. [Emotional exhaustion common among women in the public sector]. *Lakartidningen* **2002**, *99*, 2047–2052. Work in Swedish
1632. Persson, R.; Wensaas, K.-A.; Hanevik, K.; Eide, G.E.; Langeland, N.; Rortveit, G. The relationship between irritable bowel syndrome, functional dyspepsia, chronic fatigue and overactive bladder syndrome: A controlled study 6years after acute gastrointestinal infection. *BMC Gastroenterol.* **2015**, *15*.
1633. Persson, R.; Wensaas, K.-A.; Hanevik, K.; Eide, G.E.; Langeland, N.; Rortveit, G. The relationship between irritable bowel syndrome, functional dyspepsia, chronic fatigue and overactive bladder syndrome: a controlled study 6 years after acute gastrointestinal infection. *BMC Gastroenterol.* **2015**, *15*, 66.
1634. Peters, K.M. Reply to Letter-to-the-Editor: Prevalence of Pelvic Floor Dysfunction in Patients With Interstitial Cystitis. Urology 71: 1231, 2008. *Urology* **2008**, *71*, 1232.
1635. Petersen, H.H.; Jianmin, W.; Katakam, K.K.; Mejer, H.; Thamsborg, S.M.; Dalsgaard, A.; Olsen, A.; Enemark, H.L. Cryptosporidium and Giardia in Danish organic pig farms: Seasonal and age-related variation in prevalence, infection intensity and species/genotypes. *Vet. Parasitol.* **2015**, *214*, 29–39.
1636. Petersen, I.; Thomas, J.M.; Hamilton, W.T.; White, P.D. Risk and predictors of fatigue after infectious mononucleosis in a large primary-care cohort. *QJM* **2006**, *99*, 49–55.
1637. Petersen, M.W.; Schroder, A.; Jorgensen, T.; Ornbol, E.; Dantoft, T.M.; Eliasen, M.; Fink, P. RIFD - A brief clinical research interview for functional somatic disorders and health anxiety. *J. Psychosom. Res.* **2019**, *122*, 104–111.
1638. Peterson, C.; Millen, N.; Woodward, R. Chronic fatigue syndrome: A problem of legitimation. *Aust. J. Prim. Heal. - Interchang.* **1999**, *5*, 65–79.
1639. Peterson, P.K.; Pheley, A.; Schroeppel, J.; Schenck, C.; Marshall, P.; Kind, A.; Haugland, J.M.; Lambrecht, L.J.; Swan, S.; Goldsmith, S.; et al. A preliminary placebo-controlled crossover trial of fludrocortisone for chronic fatigue syndrome. *Arch. Intern. Med.* **1998**, *158*, 908–914.
1640. Peterson, P.K.; Schenck, C.H.; Sherman, R. Chronic fatigue syndrome in Minnesota. *Minn. Med.* **1991**, *74*, 21–26.
1641. Petrie, K.J.; Sivertsen, B.; Hysing, M.; Broadbent, E.; Moss-Morris, R.; Eriksen, H.R.; Ursin, H. Thoroughly modern worries - The relationship of worries about modernity to reported symptoms, health and medical care utilization. *J. Psychosom. Res.* **2001**, *51*, 395–401.
1642. Pheby, D.; Saffron, L. Risk factors for severe ME/CFS. *Biol. Med.* **2009**, *1*, 50–74.
1643. Phillips, N. The clinical and scientific basis of chronic fatigue syndrome: From myth towards management. *Australas. Psychiatry* **1998**, *6*, 186–187.

1644. Picariello, F.; Ali, S.; Moss-Morris, R.; Chalder, T. The most popular terms for medically unexplained symptoms: the views of CFS patients. *J. Psychosom. Res.* **2015**, *78*, 420–426.
1645. Pietrangelo, T.; Fulle, S.; Coscia, F.; Gigliotti, P. V; Fanò-Illic, G. Old muscle in young body: An aphorism describing the Chronic Fatigue Syndrome. *Eur. J. Transl. Myol.* **2018**, *28*, 239–247.
1646. Pigatto, P.D.; Minoia, C.; Ronchi, A.; Brambilla, L.; Ferrucci, S.M.; Spadari, F.; Passoni, M.; Somalvico, F.; Bombeccari, G.P.; Guzzi, G. Allergological and Toxicological Aspects in a Multiple Chemical Sensitivity Cohort. *Oxid. Med. Cell. Longev.* **2013**.
1647. Piko, B.; Barabas, K.; Boda, K. [Epidemiology of psychosomatic symptoms and its effect on the self-evaluation of general health in university students]. *Orv. Hetil.* **1995**, *136*, 1667–1671. Work in Hungarian
1648. Pikó, B.; Barabás, K.; Boda, K. Epidemiology of psychosomatic symptoms and its effect on the self-evaluation of general health in university students [Pszichoszomatikus tünetek epidemiológiája és hatása az egészségi állapot önértékelésére egyetemi hallgatók körében.]. *Orv. Hetil.* **1995**, *136*, 1667–1671. Work in Hungarian
1649. Pinardi, G.; Scarlato, G. [The chronic fatigue syndrome. A multifactorial approach and the treatment possibilities]. *Recenti Prog. Med.* **1990**, *81*, 773–777. Work in Italian
1650. Pinquart, M.; Shen, Y. Anxiety in children and adolescents with chronic physical illnesses: a meta-analysis. *ACTA Paediatr.* **2011**, *100*, 1069–1076.
1651. Pizzutelli, S. Systemic nickel hypersensitivity and diet: Myth or reality? *Eur. Ann. Allergy Clin. Immunol.* **2011**, *43*, 5–18.
1652. Plioplys, A. V; Plioplys, S.; Davis IV, J.S. Meeting the frustrations of chronic fatigue syndrome. *Hosp. Pract.* **1997**, *32*, 147–150+153–156+160–161+166.
1653. PLIOPLYS, S.; PLIOPLYS, A. V CHRONIC FATIGUE SYNDROME (MYALGIC ENCEPHALOPATHY). *South. Med. J.* **1995**, *88*, 993–1000.
1654. Plummer, W.P. Chronic fatigue syndrome. *Br. J. Gen. Pract.* 1991, *41*, 480.
1655. Poddighe, D.; Castelli, L.; Marseglia, G.L.; Bruni, P. A sudden onset of a pseudo-neurological syndrome after HPV-16/18 AS04-adjuvated vaccine: might it be an autoimmune/inflammatory syndrome induced by adjuvants (ASIA) presenting as a somatoform disorder? *Immunol. Res.* **2014**, *60*, 236–246.
1656. Podolecki, T.; Podolecki, A.; Hrycek, A. Fibromyalgia: pathogenetic, diagnostic and therapeutic concerns. *Pol. Arch. Med. Wewn.* **2009**, *119*, 157–161.
1657. Pohar, S.L.; Jones, C.A.; Warren, S.; Turpin, K.V.L.; Warren, K. Health status and health care utilization of multiple sclerosis in Canada. *Can. J. Neurol. Sci.* **2007**, *34*, 167–174.
1658. Poitras, P.; Gougeon, A.; Binn, M.; Bouin, M. Extra digestive manifestations of irritable bowel syndrome: Intolerance to drugs? *Dig. Dis. Sci.* **2008**, *53*, 2168–2176.
1659. Ponka, D.; Kirlew, M. Top 10 differential diagnoses in family medicine: Fatigue. *Can. Fam. Physician* **2007**, *53*, 892.
1660. Ponnusamy, V.; Owens, A.P.; Purkayastha, S.; Iodice, V.; Mathias, C.J. Orthostatic intolerance and autonomic dysfunction following bariatric surgery: A retrospective study and review of the literature. *Auton. Neurosci. Basic Clin.* **2016**, *198*, 1–7.
1661. POOL, J.H.; WALTON, J.N.; BREWIS, E.G.; Uldall, P.R.; Wright, A.E.; Gardner, P.S.; Hope Pool, J.; WALTON, J.N.; BREWIS, E.G.; Uldall, P.R.; et al. BENIGN

MYALGIC ENCEPHALOMYELITIS IN NEWCASTLE UPON TYNE. *Lancet* **1961**, *277*, 733–737.
1662. POPE, H.G.; HUDSON, J.I.; Pope Jr., H.G.; HUDSON, J.I. A SUPPLEMENTAL INTERVIEW FOR FORMS OF AFFECTIVE SPECTRUM DISORDER. *Int. J. Psychiatry Med.* **1991**, *21*, 205–232.
1663. Poppe, C.; Crombez, G.; Hanoulle, I.; Vogelaers, D.; Petrovic, M. Mental quality of life in chronic fatigue is associated with an accommodative coping style and neuroticism: a path analysis. *Qual. Life Res.* **2012**, *21*, 1337–1345.
1664. POSKANZER, D.C.; HENDERSON, D.A.; KUNKLE, E.C.; KALTER, S.S. Epidemic protracted debility at Punta Gorda, Florida: an illness resembling Iceland disease. *Trans. Am. Neurol. Assoc. 82nd Meeting*, 121–125.
1665. Poteliakhoff, A. Adrenal insufficiency. *Lancet (London, England)* 2003, *362*, 580.
1666. Pouchain, D. [Fatigued or depressed?]. *Encephale.* **2008**, *34 Spec No 2*, S17-20. Work in French
1667. Prang, N.S.; Von Baehr, V.; Bieger, W.P. Increased genetic susceptibility to xenobiotics in patients suffering from chronic fatigue syndrome after being exposed to harmful substances [Erhöhte genetische suszeptibilität gegenüber umweltgiften bei schadstoffbelasteten patienten mit chronischem erschöpfungssyndrom]. *Zeitschrift fur Umweltmedizin* **2001**, *9*, 38–45. Work in German
1668. Praxedes, M.D.; Pino, P.B. The chronic fatigue and neurasthemia in the student population. *ACTAS Esp. Psiquiatr.* **1999**, *27*, 14–21.
1669. Price, E.J.; Venables, P.J.W. Dry eyes and mouth syndrome - A subgroup of patients presenting with sicca symptoms. *Rheumatology* **2002**, *41*, 416–422.
1670. Price, R.K.; North, C.S.; Wessely, S.; Fraser, V.J. Estimating the prevalence of chronic fatigue syndrome and associated symptoms in the community. *Public Health Rep.* **1992**, *107*, 514–522.
1671. PRICE, R.K.; NORTH, C.S.; WESSELY, S.; FRASER, V.J.; Robin, R.; Lipkin, D.M.; Hume, G.W. TAKING EXCEPTION TO CHRONIC FATIGUE SYNDROME PREVALENCE FINDINGS BY PRICE, ET-AL - REPLY. *Public Health Rep.* **1993**, *108*, 136–137.
1672. Prins, J.; Bleijenberg, G.; Rouweler, E.K.; Van Der Meer, J. Effect of psychiatric disorders on outcome of cognitive-behavioural therapy for chronic fatigue syndrome. *Br. J. Psychiatry* **2005**, *187*, 184–185.
1673. Prins, J.B.; van der Meer, J.W.M.; Bleijenberg, G. Chronic fatigue syndrome. *Lancet (London, England)* **2006**, *367*, 346–355.
1674. Proal, A.D.; Albert, P.J.; Marshall, T.G.; Blaney, G.P.; Lindseth, I.A. Immunostimulation in the treatment for chronic fatigue syndrome/myalgic encephalomyelitis. *Immunol. Res.* **2013**, *56*, 398–412.
1675. Prochalska, C.; Gressier, F.; Corruble, E. Chronic Fatigue Syndrome: An independent disease? [Syndrome de Fatigue Chronique: Une entité à part entière?]. *Ann. Med. Psychol. (Paris).* **2014**, *172*, 474–478. Work in French
1676. Prochalska, C.; Gressier, F.; Corruble, E. Chronic Fatigue Syndrome: An independent disease? *Ann. Med. Psychol. (Paris).* **2014**, *172*, 474–478.
1677. Proctor, S.P.; Heaton, K.J.; White, R.F.; Wolfe, J. Chemical sensitivity and chronic fatigue in Gulf War veterans: a brief report. *J. Occup. Environ. Med.* **2001**, *43*, 259–264.
1678. Puckett, C.L. On the safety of silicone gel breast implants. *Cancer Invest.* **2000**, *18*, 278–280.

1679. Qanneta, R. Obstructive sleep apnea syndrome manifested as a subset of chronic fatigue syndrome: a comorbidity or an exclusion criterion? *Rheumatol. Int.* 2014, *34*, 441–442.
1680. Qiu, X.; Swanson, P.; Luk, K.-C.; Tu, B.; Villinger, F.; Das Gupta, J.; Silverman, R.H.; Klein, E.A.; Devare, S.; Schochetman, G.; et al. Characterization of antibodies elicited by XMRV infection and development of immunoassays useful for epidemiologic studies. *Retrovirology* **2010**, *7*.
1681. Qiu, X.; Swanson, P.; Tang, N.; Leckie, G.W.; Devare, S.G.; Schochetman, G.; Hackett, J.J. Seroprevalence of xenotropic murine leukemia virus-related virus in normal and retrovirus-infected blood donors. *Transfusion* **2012**, *52*, 307–316.
1682. Queiroz, L.P. Worldwide Epidemiology of Fibromyalgia. *Curr. Pain Headache Rep.* **2013**, *17*.
1683. Rabhi, M.; Ennibi, K.; Chaari, J.; Toloune, F. Functional somatic syndromes. *Rev. Med. INTERNE* **2010**, *31*, 17–22.
1684. Rabhi, M.; Ennibi, K.; Chaari, J.; Toloune, F. Functional somatic syndromes [Les syndromes somatiques fonctionnels]. *Rev. Med. Interne* **2010**, *31*, 17–22. Work in French
1685. Racciatti, D.; Gorgoretti, V.; Sepede, G.; Gambi, F.; Pizzigallo, E. An Italian study on health-related quality of life and fatigue in patients with chronic fatigue syndrome and patients with chronic HCV virus infection: similarities and differences. *Int. J. Immunopathol. Pharmacol.* **2011**, *24*, 673–681.
1686. Rajeevan, M.S.; Dimulescu, I.; Murray, J.; Falkenberg, V.R.; Unger, E.R. Pathway-focused genetic evaluation of immune and inflammation related genes with chronic fatigue syndrome. *Hum. Immunol.* **2015**, *76*, 553–560.
1687. Rajeevan, M.S.; Murray, J.; Oakley, L.; Lin, J.-M.S.; Unger, E.R. Association of chronic fatigue syndrome with premature telomere attrition. *J. Transl. Med.* **2018**, *16*.
1688. Rangel, L.; Garralda, E.; Levin, M.; Roberts, H. Personality in adolescents with chronic fatigue syndrome. *Eur. Child Adolesc. Psychiatry* **2000**, *9*, 39–45.
1689. Rangel, L.; Garralda, M.E.; Hall, A.; Woodham, S. Psychiatric adjustment in chronic fatigue syndrome of childhood and in juvenile idiopathic arthritis. *Psychol. Med.* **2003**, *33*, 289–297.
1690. Ranjith, G. Epidemiology of chronic fatigue syndrome. *Occup. Med. (Lond).* **2005**, *55*, 13–19.
1691. Ranque-Garnier, S.; Eldin, C.; Sault, C.; Raoult, D.; Donnet, A. Management of patients presenting with generalized musculoskeletal pain and a suspicion of Lyme disease [Conduite à tenir devant un tableau polyalgique chez des patients adressés pour suspicion de maladie de Lyme]. *Med. Mal. Infect.* **2019**, *49*, 157–166. Work in French
1692. Rantzen, E. No cause, no cure. *Nurs. Stand.* **2000**, *14*, 23.
1693. Rao, A.; Sibbritt, D.; Phillips, J.L.; Hickman, L.D. Prayer or spiritual healing as adjuncts to conventional care: a cross sectional analysis of prevalence and characteristics of use among women. *BMJ Open* **2015**, *5*.
1694. Rasa, S.; Nora-Krukle, Z.; Henning, N.; Eliassen, E.; Shikova, E.; Harrer, T.; Scheibenbogen, C.; Murovska, M.; Prusty, B.K. Chronic viral infections in myalgic encephalomyelitis/chronic fatigue syndrome (ME/CFS). *J. Transl. Med.* **2018**, *16*.
1695. Rasa, S.; Nora-Krukle, Z.; Chapenko, S.; Krumina, A.; Roga, S.; Murovska, M. No evidence of XMRV provirus sequences in patients with myalgic

encephalomyelitis/chronic fatigue syndrome and individuals with unspecified encephalopathy. *New Microbiol.* **2014**, *37*, 17–24.
1696. Rask, C.U. Functional somatic symptoms in 5-7-year-old children. Assessment, prevalence and co-occurrence. *Dan. Med. J.* **2012**, *59*.
1697. Raskov, H.; Burcharth, J.; Pommergaard, H.-C.; Rosenberg, J. Irritable bowel syndrome, the microbiota and the gut-brain axis. *Gut Microbes* **2016**, *7*, 365–383.
1698. Rasmussen, A.K.; Nielsen, H.; Andersen, V.; Barington, T.; Bendtzen, K.; Hansen, M.B.; Nielsen, L.; Pedersen, B.K.; Wiik, A. [Chronic fatigue syndrome--a controlled cross-sectional study]. *Ugeskr. Laeger* **1994**, *156*, 6836–6840. Work in Danish
1699. Rasmussen, A.K.; Nielsen, H.; Andersen, V.; Barington, T.; Bendtzen, K.; Hansen, M.B.; Nielsen, L.; Pedersen, B.K.; Wiik, A. Chronic fatigue syndrome--a controlled cross sectional study. *J. Rheumatol.* **1994**, *21*, 1527–1531.
1700. Rasouli, O.; Fors, E.A.; Vasseljen, O.; Stensdotter, A.-K. A Concurrent Cognitive Task Does Not Perturb Quiet Standing in Fibromyalgia and Chronic Fatigue Syndrome. *Pain Res. Manag.* **2018**, *2018*, 9014232.
1701. Rathore, M.H. Human herpesvirus 6. *South. Med. J.* **1993**, *86*, 1197–1201.
1702. Rathwell, I.; Simonoff, E. Editorial Perspective: Key issues in children with intellectual disability for practitioners. *Child Adolesc. Ment. Health* **2019**, *24*, 194–198.
1703. Ravindran, M.; Baraniuk, J.N. The sinus headache explained. *Curr. Allergy Asthma Rep.* **2010**, *10*, 202–209.
1704. Ravindran, M.K.; Zheng, Y.; Timbol, C.; Merck, S.J.; Baraniuk, J.N. Migraine headaches in chronic fatigue syndrome (CFS): comparison of two prospective cross-sectional studies. *BMC Neurol.* **2011**, *11*, 30.
1705. Ravindran, M.; Adewuyi, O.; Zheng, Y.; Rayhan, R.U.; Le, U.; Timbol, C.; Merck, S.; Esteitie, R.; Read, C.; Cooney, M.; et al. Dyspnea in Chronic Fatigue Syndrome (CFS): comparison of two prospective cross-sectional studies. *Glob. J. Health Sci.* **2012**, *5*, 94–110.
1706. Ray, C.; Jefferies, S.; Weir, W.R. Coping and other predictors of outcome in chronic fatigue syndrome: a 1-year follow-up. *J. Psychosom. Res.* **1997**, *43*, 405–415.
1707. Ray, C.; Jefferies, S.; Weir, W.R. Life-events and the course of chronic fatigue syndrome. *Br. J. Med. Psychol.* **1995**, *68 (Pt 4)*, 323–331.
1708. Ray, C.; Jefferies, S.; Weir, W.R. Coping with chronic fatigue syndrome: illness responses and their relationship with fatigue, functional impairment and emotional status. *Psychol. Med.* **1995**, *25*, 937–945.
1709. Rayhan, R.U.; Ravindran, R.K.; Baraniuk, J.N. Migraine in gulf war illness and chronic fatigue syndrome: Prevalence, potential mechanisms, and evaluation. *Front. Physiol.* **2013**, *4 JUL*.
1710. Reale, M.; Conti, L.; Velluto, D. Immune and Inflammatory-Mediated Disorders: From Bench to Bedside. *J. Immunol. Res.* **2018**, *2018*.
1711. Redmond, C.K. Analysis of clinical, epidemiologic, and laboratory data on chronic fatigue syndrome. *Rev. Infect. Dis.* **1991**, *13 Suppl 1*, S90-3.
1712. Reed, B.D.; Harlow, S.D.; Sen, A.; Edwards, R.M.; Chen, D.; Haefner, H.K. Relationship Between Vulvodynia and Chronic Comorbid Pain Conditions. *Obstet. Gynecol.* **2012**, *120*, 145–151.
1713. Rees, R.J.; Bellon, M.L. Post concussion syndrome ebb and flow: Longitudinal effects and management. *NeuroRehabilitation* **2007**, *22*, 229–242.

1714. Reeves, W.C.; Jones, J.F.; Maloney, E.; Heim, C.; Hoaglin, D.C.; Boneva, R.S.; Morrissey, M.; Devlin, R. Prevalence of chronic fatigue syndrome in metropolitan, urban, and rural Georgia. *Popul. Health Metr.* **2007**, *5*.
1715. Reeves, W.C.; Lloyd, A.; Vernon, S.D.; Klimas, N.; Jason, L.A.; Bleijenberg, G.; Evengard, B.; White, P.D.; Nisenbaum, R.; Unger, E.R.; et al. Identification of ambiguities in the 1994 chronic fatigue syndrome research case definition and recommendations for resolution. *BMC Health Serv. Res.* **2003**, *3*.
1716. Reeves, W.C.; Stamey, F.R.; Black, J.B.; Mawle, A.C.; Stewart, J.A.; Pellett, P.E. Human herpesviruses 6 and 7 in chronic fatigue syndrome: A case-control study. *Clin. Infect. Dis.* **2000**, *31*, 48–52.
1717. Reeves, W.C.; Heim, C.; Maloney, E.M.; Youngblood, L.S.; Unger, E.R.; Decker, M.J.; Jones, J.F.; Rye, D.B. Sleep characteristics of persons with chronic fatigue syndrome and non-fatigued controls: results from a population-based study. *BMC Neurol.* **2006**, *6*, 41.
1718. Reeves, W.C.; Lin, J.-M.S.; Nater, U.M. Mental illness in metropolitan, urban and rural Georgia populations. *BMC Public Health* **2013**, *13*, 414.
1719. Regenauer, A. Contentious diseases--a medico-social phenomenon from an insurance medicine perspective [Schwer objektivierbare Erkrankungen aus der Sicht der Versicherungsmedizin.]. *Versicherungsmedizin* **2008**, *60*, 3–7. Work in German
1720. Reid, S. Multiple chemical sensitivity - Is the environment really to blame? *J. R. Soc. Med.* **1999**, *92*, 616–619.
1721. Reid, S.; Chalder, T.; Cleare, A.; Hotopf, M.; Wessely, S. Extracts from ``clinical evidence{''} - Chronic fatigue syndrome. *BMJ-BRITISH Med. J.* **2000**, *320*, 292–296.
1722. Reid, S.; Chalder, T.; Cleare, A.; Hotopf, M.; Wessely, S. Chronic fatigue syndrome. *Br. Med. J.* **2000**, *320*, 292–296.
1723. Reid, S.; Hotopf, M.; Hull, L.; Ismail, K.; Unwin, C.; Wessely, S. Multiple chemical sensitivity and chronic fatigue syndrome in British Gulf War veterans. *Am. J. Epidemiol.* **2001**, *153*, 604–609.
1724. Reid, S.F.; Chalder, T.; Cleare, A.; Hotopf, M.; Wessely, S. Chronic fatigue syndrome. *BMJ Clin. Evid.* **2008**, *2008*.
1725. Reinertsen, K.V.; Cvancarova, M.; Loge, J.H.; Edvardsen, H.; Wist, E.; Fossa, S.D. Predictors and course of chronic fatigue in long-term breast cancer survivors. *J. Cancer Surviv.* **2010**, *4*, 405–414.
1726. Rest, K.M. Advancing the understanding of multiple chemical sensitivity (MCS): overview and recommendations from an AOEC workshop. *Toxicol. Ind. Health* 1992, *8*, 1–13.
1727. Reuter, K.; Harter, M. The concepts of fatigue and depression in cancer. *Eur. J. Cancer Care (Engl).* **2004**, *13*, 127–134.
1728. Revelas, A.; Baltaretsou, E. Chronic fatigue syndrome: Diagnosis and treatment. *South African Fam. Pract.* **2013**, *55*, 53–55.
1729. Reyes, M.; Dobbins, J.G.; Mawle, A.C.; Steele, L.; Gary Jr., H.E.; Malani, H.; Schmid, S.; Fukuda, K.; Stewart, J.; Nisenbaum, R.; et al. Risk factors for chronic fatigue syndrome: A case-control study. *J. Chronic Fatigue Syndr.* **1996**, *2*, 17–33.
1730. Reyes, M.; Gary, H.E.J.; Dobbins, J.G.; Randall, B.; Steele, L.; Fukuda, K.; Holmes, G.P.; Connell, D.G.; Mawle, A.C.; Schmid, D.S.; et al. Surveillance for chronic fatigue syndrome--four U.S. cities, September 1989 through August 1993.

MMWR. CDC Surveill. Summ. Morb. Mortal. Wkly. report. CDC Surveill. Summ. **1997**, *46*, 1–13.
1731. Reyes, M.; Nisenbaum, R.; Hoaglin, D.C.; Unger, E.R.; Emmons, C.; Randall, B.; Stewart, J.A.; Abbey, S.; Jones, J.F.; Gantz, N.; et al. Prevalence and incidence of chronic fatigue syndrome in Wichita, Kansas. *Arch. Intern. Med.* **2003**, *163*, 1530–1536.
1732. Reynaga-Estrada, P.; Robles, D.A.M.; Valadez Jiménez, A.; Rodríguez Villalobos, A.; González Hernández, J. Burnout syndrome in Mexican university sportmen [Síndrome de burnout en deportistas universitarios Mexicanos]. *Rev. Psicol. del Deport.* **2017**, *26*, 209–214. Work in Spanish
1733. Reynaga-Estrada, P.; Mena Robles, D.A.; Valadez Jimenez, A.; Rodriguez Villalobos, A.; Gonzalez Hernandez, J. BURNOUT SYNDROME IN MEXICAN UNIVERSITY SPORTMEN. *Rev. Psicol. DEL Deport.* **2017**, *26*, 209–214.
1734. Reynolds, G.K.; Lewis, D.P.; Richardson, A.M.; Lidbury, B.A. Comorbidity of postural orthostatic tachycardia syndrome and chronic fatigue syndrome in an Australian cohort. *J. Intern. Med.* **2014**, *275*, 409–417.
1735. Richards, J. Chronic fatigue syndrome in children and adolescents: A review article. *Clin. Child Psychol. Psychiatry* **2000**, *5*, 31–51.
1736. Richards, R.S.; McGregor, N.R.; Roberts, T.K. Association between oxidative damage markers and self-reported temporomandibular dysfunction symptoms in patients with chronic fatigue syndrome. *J. Chronic Fatigue Syndr.* **2004**, *12*, 45–61.
1737. Richards, S.C.M.; Scott, D.L. Prescribed exercise in people with fibromyalgia: parallel group randomised controlled trial. *BMJ-BRITISH Med. J.* **2002**, *325*, 185–187.
1738. Richardson, A.M.; Lewis, D.P.; Kita, B.; Ludlow, H.; Groome, N.P.; Hedger, M.P.; de Kretser, D.M.; Lidbury, B.A. Weighting of orthostatic intolerance time measurements with standing difficulty score stratifies ME/CFS symptom severity and analyte detection. *J. Transl. Med.* **2018**, *16*.
1739. Richardson, A. The symptoms and management of myalgic encephalomyelitis. *Nurs. Times* **2002**, *98*, 32–35.
1740. Richardson, J. Myalgic enchephalomyelitis: Guidelines for doctors. *J. Chronic Fatigue Syndr.* **2002**, *10*, 65–80.
1741. Richardson, J.C. Establishing the (extra) ordinary in chronic widespread pain. *Health (Irvine. Calif).* **2005**, *9*, 31–48. Proceedings of the 8th Annual Qualitative Health Research Conference in Banff, Canada.
1742. Richardson, R.D.; Engel, C.C.J. Evaluation and management of medically unexplained physical symptoms. *Neurologist* **2004**, *10*, 18–30.
1743. Richardson, S.J.; Morgan, N.G.; Foulis, A.K. Pancreatic Pathology in Type 1 Diabetes Mellitus. *Endocr. Pathol.* **2014**, *25*, 80–92.
1744. Richman, J.A.; Flaherty, J.A.; Rospenda, K.M. Chronic fatigue syndrome: have flawed assumptions been derived from treatment-based studies? *Am. J. Public Health* **1994**, *84*, 282–284.
1745. Richman, J.A.; Jason, L.A.; Taylor, R.R.; Jahn, S.C. Feminist perspectives on the social construction of chronic fatigue syndrome. *Health Care Women Int.* **2000**, *21*, 173–185.
1746. Richt, J.A.; Rott, R. Borna disease virus: a mystery as an emerging zoonotic pathogen. *Vet. J.* **2001**, *161*, 24–40.
1747. Rico-Villademoros, F.; Calandre, E.P. Fibromyalgia: Comorbidity indicative of vulnerability? *Med. Clin. (Barc).* **2014**, *142*, 538–539.

1748. Riedl, A.; Schmidtmann, M.; Stengel, A.; Goebel, M.; Wisser, A.-S.; Klapp, B.F.; Monnikes, H. Somatic comorbidities of irritable bowel syndrome: a systematic analysis. *J. Psychosom. Res.* **2008**, *64*, 573–582.
1749. Rief, W.; Avorn, J.; Barsky, A.J. Medication-attributed adverse effects in placebo groups - Implications for assessment of adverse effects. *Arch. Intern. Med.* **2006**, *166*, 155–160.
1750. Rief, W.; Isaac, M. The future of somatoform disorders: Somatic symptom disorder, bodily distress disorder or functional syndromes? *Curr. Opin. Psychiatry* **2014**, *27*, 315–319.
1751. Rief, W.; Pilger, F.; Ihle, D.; Verkerk, R.; Scharpe, S.; Maes, M. Psychobiological aspects of somatoform disorders: Contributions of monoaminergic transmitter systems. *Neuropsychobiology* **2004**, *49*, 24–29.
1752. Rigney, T. Allostatic Load and Delirium in the Hospitalized Older Adult. *Nurs. Res.* **2010**, *59*, 322–330.
1753. Rimbaut, S.; Van Gutte, C.; Van Brabander, L.; Vanden Bossche, L. Chronic fatigue syndrome – an update. *Acta Clin. Belgica Int. J. Clin. Lab. Med.* **2016**, *71*, 273–280.
1754. Rimes, K.A.; Ashcroft, J.; Bryan, L.; Chalder, T. Emotional suppression in chronic fatigue syndrome: Experimental study. *Health Psychol.* **2016**, *35*, 979–986.
1755. Rimes, K.A.; Goodman, R.; Hotopf, M.; Wessely, S.; Meltzer, H.; Chalder, T. Incidence, prognosis, and risk factors for fatigue and chronic fatigue syndrome in adolescents: a prospective community study. *Pediatrics* **2007**, *119*, e603-9.
1756. Robbins, L. Refractory headache definition. *Headache* **2011**, *51*, 310–311.
1757. Roberts, E.; Wessely, S.; Chalder, T.; Chang, C.-K.; Hotopf, M. Mortality of people with chronic fatigue syndrome: a retrospective cohort study in England and Wales from the South London and Maudsley NHS Foundation Trust Biomedical Research Centre (SLaM BRC) Clinical Record Interactive Search (CRIS) Register. *Lancet (London, England)* **2016**, *387*, 1638–1643.
1758. Robertson-Ritchie, H. Toward a new definition of chronic fatigue syndrome. *West. J. Med.* 2001, *174*, 241.
1759. Robinson, L.J.; Durham, J.; MacLachlan, L.L.; Newton, J.L. Autonomic function in chronic fatigue syndrome with and without painful temporomandibular disorder. *FATIGUE-BIOMEDICINE Heal. Behav.* **2015**, *3*, 205–219.
1760. Robinson, M.J.; Erlwein, O.W.; Kaye, S.; Weber, J.; Cingoz, O.; Patel, A.; Walker, M.M.; Kim, W.-J.; Uiprasertkul, M.; Coffin, J.M.; et al. Mouse DNA contamination in human tissue tested for XMRV. *Retrovirology* **2010**, *7*.
1761. Robinson, M.J.; Tuke, P.W.; Erlwein, O.; Tettmar, K.I.; Kaye, S.; Naresh, K.N.; Patel, A.; Walker, M.M.; Kimura, T.; Gopalakrishnan, G.; et al. No evidence of XMRV or MuLV sequences in prostate cancer, diffuse large B-cell lymphoma, or the UK blood donor population. *Adv. Virol.* **2011**, *2011*.
1762. Robinson, P.; MacDonell, M. Priorities for mixtures health effects research. *Environ. Toxicol. Pharmacol.* **2004**, *18*, 201–213. Proceedings of the International Conference on Chemical Mixtures (ICCM) in Atlanta, USA.
1763. Rocha, E.A. Neurally mediated syndromes [Síndromes neuralmente mediadas]. *Arq. Bras. Cardiol.* **2006**, *87*, e32–e41. Work in Spanish
1764. Rodevand, L. How is it to be an adolescent living with chronic fatigue syndrome/myalgic encephalomyelitis? A narrative review [Hvordan er det å være ungdom og leve med kronisk utmattelsessyndrom/myalgisk encefalopati? En narrativ oversikt]. *Scand. Psychol.* **2017**, *4*. Work in Norwegian

1765. Rodriguez, A.S.; Marono, C.G.; Ledesma, M.S. Chronic fatigue syndrome: A syndrome in search of definition. *Rev. Clin. Esp.* **2005**, *205*, 70–74.
1766. Roehr, B. Researchers find no link between XMRV and chronic fatigue syndrome. *BMJ* 2012, *345*, e6331.
1767. Roerink, M.E.; Lenders, J.W.M.; Schmits, I.C.; Pistorius, A.M.A.; Smit, J.W.; Knoop, H.; van der Meer, J.W.M. Postural orthostatic tachycardia is not a useful diagnostic marker for chronic fatigue syndrome. *J. Intern. Med.* **2017**, *281*, 179–188.
1768. Rogers, D.C.; Dittner, A.J.; Rimes, K.A.; Chalder, T. Fatigue in an adult attention deficit hyperactivity disorder population: A trans-diagnostic approach. *Br. J. Clin. Psychol.* **2017**, *56*, 33–52.
1769. Rollnik, J.D. Chronic Fatigue Syndrome: A Critical Review [Das chronische Müdigkeitssyndrom - ein kritischer Diskurs]. *Fortschritte der Neurol. Psychiatr.* **2017**, *85*, 79–85. Work in German
1770. Rollnik, J.D. Chronic Fatigue Syndrome: A Critical Review. *FORTSCHRITTE DER Neurol. Psychiatr.* **2017**, *85*, 79–85.
1771. Roma, M.; Marden, C.L.; De Wandele, I.; Francomano, C.A.; Rowe, P.C. Postural tachycardia syndrome and other forms of orthostatic intolerance in Ehlers-Danlos syndrome. *Auton. Neurosci. Basic Clin.* **2018**, *215*, 89–96.
1772. Roman, P.; Estevez, A.F.; Sanchez-Labraca, N.; Canadas, F.; Miras, A.; Cardona, D. Probiotics for fibromyalgia: study design for a pilot double-blind, randomized controlled trial. *Nutr. Hosp.* **2017**, *34*, 250–255.
1773. Romans, S.; Cohen, M. Unexplained and underpowered: The relationship between psychosomatic disorders and interpersonal abuse - A critical review. *Harv. Rev. Psychiatry* **2008**, *16*, 35–54.
1774. Romans, S.; Belaise, C.; Martin, J.; Morris, E.; Raffi, A. Childhood abuse and later medical disorders in women. An epidemiological study. *Psychother. Psychosom.* **2002**, *71*, 141–150.
1775. Rome, J.D.; Sletten, C.D.; Bruce, B.K. A rehabilitation approach to chronic pain in rheumatologic practice. *Curr. Opin. Rheumatol.* **1996**, *8*, 163–168.
1776. Romero, H.R.; Navarro, P.; Gómez, L. V; De La Parte, M.A.; Ramírez, E. V Infectious mononucleosis as an international health problem [Mononucleosis Infecciosa como Problema Internacional de Salud]. *Inf. Med.* **2014**, *16*, 134–141.
1777. Romero, H.R.; Navarro, P.; Gómez, L. V; De La Parte, M.A.; Ramírez, E. V Infectious mononucleosis, an int [La mononucleosis infecciosa, un problema internacional de salud]. *Inf. Med.* **2013**, *15*, 27–35. Work in Spanish
1778. Rosen, S.D.; King, J.C.; Wilkinson, J.B.; Nixon, P.G. Is chronic fatigue syndrome synonymous with effort syndrome? *J. R. Soc. Med.* **1990**, *83*, 761–764.
1779. Roser Galard, C.; Juncadella Garcia, E.; Hernandez Hernandez, A.; Maymo Pijuan, N. [Chronic fatigue syndrome: is it ignored in primary care?]. *Aten. primaria* 1995, *15*, 587–588. Work in Spanish
1780. Ross, R.L.; Jones, K.D.; Ward, R.L.; Wood, L.J.; Bennett, R.M. Atypical depression is more common than melancholic in fibromyalgia: an observational cohort study. *BMC Musculoskelet. Disord.* **2010**, *11*.
1781. Rosti-Otajarvi, E.; Hamalainen, P. Behavioural symptoms and impairments in multiple sclerosis: a systematic review and meta-analysis. *Mult. Scler. J.* **2013**, *19*, 31–45.
1782. Rouillon, F.; Delhommeau, L.; Vinceneux, P. [Chronic fatigue syndrome]. *Presse Med.* **1996**, *25*, 2031–2036. Work in French

1783. Roussou, E.; Ciurtin, C. Clinical overlap between fibromyalgia tender points and enthesitis sites in patients with spondyloarthritis who present with inflammatory back pain. *Clin. Exp. Rheumatol.* **2012**, *30,* S24–S30.
1784. Rowat, S.C. Paraoxonase/MCS [4]. *Environ. Health Perspect.* **1999**, *107,* A395.
1785. Rowe, K.S. Long Term Follow up of Young People With Chronic Fatigue Syndrome Attending a Pediatric Outpatient Service. *Front. Pediatr.* **2019**, *7*.
1786. Rowe, P.C.; Calkins, H. Neurally mediated hypotension and chronic fatigue syndrome. *Am. J. Med.* **1998**, *105*, 15S–21S.
1787. Rowe, P.C.; Marden, C.L.; Flaherty, M.A.K.; Jasion, S.E.; Cranston, E.M.; Johns, A.S.; Fan, J.; Fontaine, K.R.; Violand, R.L. Impaired range of motion of limbs and spine in chronic fatigue syndrome. *J. Pediatr.* **2014**, *165*, 360–366.
1788. Rowe, P.C.; Underhill, R.A.; Friedman, K.J.; Gurwitt, A.; Medow, M.S.; Schwartz, M.S.; Speight, N.; Stewart, J.M.; Vallings, R.; Rowe, K.S. Myalgic encephalomyelitis/chronic fatigue syndrome diagnosis and management in young people: A primer. *Front. Pediatr.* **2017**, *5*.
1789. Rowe, P.C.; Lucas, K.E. Orthostatic intolerance in chronic fatigue syndrome. *Am. J. Med.* 2007, *120*, e13.
1790. Rowe, P.C.; Marden, C.L.; Jasion, S.E.; Cranston, E.M.; Flaherty, M.A.K.; Kelly, K.J. Cow's milk protein intolerance in adolescents and young adults with chronic fatigue syndrome. *Acta Paediatr.* **2016**, *105*, e412-8.
1791. Rowland, T. Announcing the 2005 Roy Acuff ACSM abstract awards. *Pediatr. Exerc. Sci.* **2005**, *17*, 323–328.
1792. Roy-Byrne, P.; Smith, W.R.; Goldberg, J.; Afari, N.; Buchwald, D. Post-traumatic stress disorder among patients with chronic pain and chronic fatigue. *Psychol. Med.* **2004**, *34*, 363–368.
1793. Royes, B.; Alvarez, C.; Lalinde, S.; Vidal, L.; Martín, A. [Chronic fatigue syndrome: more than fatigue]. [Síndrome de fatiga crónica. Mucho más que fatiga.]. *Rev. Enferm.* **2010**, *33,* 16–19. Work in Spanish
1794. Rubal, E.; Iwanenko, W. Chronic fatigue syndrome: Is there a role for occupational therapy? *Occup. Ther. Heal. Care* **2004**, *18*, 33–45.
1795. Rubin, G.J.; Hardy, R.; Hotopf, M. A systematic review and meta-analysis of the incidence and severity of postoperative fatigue. *J. Psychosom. Res.* **2004**, *57,* 317–326.
1796. Rubin, J.J. Psychosomatic pain: New insights and management strategies. *South. Med. J.* **2005**, *98,* 1099–1112.
1797. Rubio-Tapia, A.; Hill, I.D.; Kelly, C.P.; Calderwood, A.H.; Murray, J.A. ACG clinical guidelines: Diagnosis and management of celiac disease. *Am. J. Gastroenterol.* **2013**, *108,* 656–676.
1798. Rucińska, M.; Tokajuk, P.; Wojtukiewicz, M.Z. Chronic fatigue syndrome in cancer patients treated with radiotherapy [Zespół przewlekłego zmęzenia u chorych na nowotwory leczonych energią promienistą]. *Nowotwory* **2004**, *54,* 143–147. Work in Polish
1799. Rudolph, T.; Larsen, J.P.; Farbu, E. The long-term functional status in patients with Guillain-Barre syndrome. *Eur. J. Neurol.* **2008**, *15*, 1332–1337.
1800. Rueffer, J.U.; Adamietz, I.A. Fatigue - cancer-related exhaustion. An unavoidable destiny? *ONKOLOGE* **2013**, *19*, 279+.
1801. Ruiz Castro, M.; Nadador, V.; Fernández-Alcantud, J.; Hernández-Salván, J.; Riquelme, I.; Benito, G. Muscular pain: Myofascial pain syndrome and fibromyalgia [Dolor de origen muscular: Dolor miofascial y fibromialgia]. *Rev. la Soc. Esp. del Dolor* **2007**, *14*, 36–44. Work in Spanish

1802. Ruiz-Nunez, B.; Tarasse, R.; Vogelaar, E.F.; Dijck-Brouwer, D.A.J.; Muskiet, F.A.J. Higher Prevalence of ``Low T3 Syndrome{''} in Patients With Chronic Fatigue Syndrome: A Case-Control Study. *Front. Endocrinol. (Lausanne).* **2018**, *9*.
1803. Ruiz-Núñez, B.; Tarasse, R.; Vogelaar, E.F.; Dijck-Brouwer, D.A.J.; Muskiet, F.A.J. Higher prevalence of "Low T3 Syndrome" in patients with chronic fatigue syndrome: A case-control study. *Front. Endocrinol. (Lausanne).* **2018**, *9*.
1804. Ruiz, E.; Alegre, J.; Garcia Quintana, A.M.; Aliste, L.; Blazquez, A.; Fernandez de Sevilla, T. [Chronic fatigue syndrome: study of a consecutive series of 824 cases assessed in two specialized units]. *Rev. Clin. Esp.* **2011**, *211*, 385–390. Work in Spanish
1805. Ruiz, E.; Alegre, J.; García Quintana, A.M.; Aliste, L.; Blázquez, A.; Fernández De Sevilla, T. Chronic fatigue syndrome: Study of a consecutive series of 824 cases assessed in two specialized units [Síndrome de fatiga crónica: estudio de una serie consecutiva de 824 casos evaluados en dos unidades especializadas]. *Rev. Clin. Esp.* **2011**, *211*, 385–390. Work in Spanish
1806. Rusmevichientong, A.; Chow, S.A. Biology and pathophysiology of the new human retrovirus XMRV and its association with human disease. *Immunol. Res.* **2010**, *48*, 27–39.
1807. Russell, I.J.; Michalek, J.E.; Kang, Y.K.; Richards, A.B. Reduction of morning stiffness and improvement in physical function in fibromyalgia syndrome patients treated sublingually with low doses of human interferon-alpha. *J. Interf. CYTOKINE Res.* **1999**, *19*, 961–968.
1808. Russell, I.J.; Vipraio, G.A.; Michalek, J.E.; Craig, F.E.; Kang, Y.K.; Richards, A.B. Lymphocyte markers and natural killer cell activity in fibromyalgia syndrome: Effects of low-dose, sublingual use of human interferon-alpha. *J. Interf. CYTOKINE Res.* **1999**, *19*, 969–978.
1809. Russo, J.; Katon, W.; Clark, M.; Kith, P.; Sintay, M.; Buchwald, D. Longitudinal changes associated with improvement in chronic fatigue patients. *J. Psychosom. Res.* **1998**, *45*, 67–76.
1810. Rusu, C.; Gee, M.E.; Lagace, C.; Parlor, M. Chronic fatigue syndrome and fibromyalgia in Canada: prevalence and associations with six health status indicators. *Heal. Promot. chronic Dis. Prev. Canada Res. policy Pract.* **2015**, *35*, 3–11.
1811. Sa, M.J. Psychological aspects of multiple sclerosis. *Clin. Neurol. Neurosurg.* **2008**, *110*, 868–877.
1812. Sack, M.; Henningsen, P. Neurasthenia and chronic fatigue syndrome - an overview of the empirical literature. *Z. Psychosom. Med. Psychoanal.* **1998**, *44*, 319–337.
1813. Sack, M.; Henningsen, P. Neurasthenia and chronic fatigue syndrome - An overview of the empirical literature [Neurasthenie und chronic fatigue syndrome - Eine übersicht zur empirischen literatur]. *Z. Psychosom. Med. Psychother.* **1998**, *44*, 319–337. Work in German
1814. Saez Francas, N.; Alegre, J.; Calvo Pinero, N.; Ramos Quiroga, J.A.; Ruiz, E.; Olivares, B.; Garcia Gimenez, E.; Casas, M. PREVALENCE OF ADHD IN CHRONIC FATIGUE SYNDROME. *Eur. PSYCHIATRY* **2011**, *26*.
1815. Saez-Francas, N.; Calvo, N.; Alegre, J.; Castro-Marrero, J.; Ramirez, N.; Hernandez-Vara, J.; Casas, M. Childhood trauma in Chronic Fatigue Syndrome: focus on personality disorders and psychopathology. *Compr. Psychiatry* **2015**, *62*, 13–19.

1816. Saidi, G.; Haines, L. The management of children with chronic fatigue syndrome-like illness in primary care: a cross-sectional study. *Br. J. Gen. Pract.* **2006**, *56*, 43–47.
1817. Sakakibara, S.; Sakakibara, K.; Tosato, G. NF-kappa B Activation Stimulates Transcription and Replication of Retrovirus XMRV in Human B-Lineage and Prostate Carcinoma Cells. *J. Virol.* **2011**, *85*, 3179–3186.
1818. Sakuma, T.; Hué, S.; Squillace, K.A.; Tonne, J.M.; Blackburn, P.R.; Ohmine, S.; Thatava, T.; Towers, G.J.; Ikeda, Y. No evidence of XMRV in prostate cancer cohorts in the Midwestern United States. *Retrovirology* **2011**, *8*.
1819. Sakuma, T.; Tonne, J.M.; Malcolm, J.A.; Thatava, T.; Ohmine, S.; Peng, K.-W.; Ikeda, Y. Long-term infection and vertical transmission of a gammaretrovirus in a foreign host species. *PLoS One* **2012**, *7*.
1820. Salehpour, S.; Tavakkoli, S. Cyclic pamidronate therapy in children with osteogenesis imperfecta. *J. Pediatr. Endocrinol. Metab.* **2010**, *23*, 73–80.
1821. Saletu, B.; Anderer, P.; Saletu-Zyhlarz, G.M. Nonorganic hypersomnia - Epidemiology, diagnosis and treatment [Nichtorganische hypersomnie - Epidemiologie, diagnose und therapie]. *Wien. Klin. Wochenschr.* **2001**, *113*, 266–277. Work in German
1822. Salkovskis, P.M.; Gregory, J.D.; Sedgwick-Taylor, A.; White, J.; Opher, S.; Ólafsdóttir, S. Extending cognitive-behavioural theory and therapy to medically unexplained symptoms and long-term physical conditions: A hybrid transdiagnostic/problem specific approach. *Behav. Chang.* **2016**, *33*, 172–192.
1823. Saltzstein, B.J.; Wyshak, G.; Hubbuch, J.T.; Perry, J.C. A naturalistic study of the chronic fatigue syndrome among women in primary care. *Gen. Hosp. Psychiatry* **1998**, *20*, 307–316.
1824. Samuel, B.; Axelband, J.; Mckim, K.; Leh, D. Borrelia burgdorferi: A clinical chameleon. *Consultant* **2015**, *55*, 530–535.
1825. Sanchez Rodriguez, A.; Gonzalez Marono, C.; Sanchez Ledesma, M. [Chronic fatigue syndrome: a syndrome in search of definition]. *Rev. Clin. Esp.* **2005**, *205*, 70–74. Work in Spanish
1826. Sánchez Rodríguez, A.; González Maroño, C.; Sánchez Ledesma, M. Chronic fatigue syndrome: A syndrome in search of definition [Síndrome de fatiga crónica: Un síndrome en busca de definición]. *Rev. Clin. Esp.* **2005**, *205*, 70–74. Spanish
1827. Sanchez, A.I.; Valenza, M.C.; Martinez, M.P.; Miro, E.; Diener, F.N.; Lami, J.; Caliz, R. Gender Differences in Pain Experience and Physical Activity of Fibromyalgia Syndrome Patients. *J. Musculoskelet. Pain* **2013**, *21*, 147–155.
1828. Sankey, A.; Hill, C.M.; Brown, J.; Quinn, L.; Fletcher, A. A follow-up study of chronic fatigue syndrome in children and adolescents: symptom persistence and school absenteeism. *Clin. Child Psychol. Psychiatry* **2006**, *11*, 126–138.
1829. Sansone, R.A.; Whitecar, P.; Wiederman, M.W. Psychophysiological disorders among buprenorphine patients. *Int. J. Psychiatry Clin. Pract.* **2009**, *13*, 338–340.
1830. Santamarina-Perez, P.; Eiroa-Orosa, F.J.; Freniche, V.; Moreno-Mayos, A.; Alegre, J.; Saez, N.; Jacas, C. Length of illness does not predict cognitive dysfunction in chronic fatigue syndrome. *Appl. Neuropsychol.* **2011**, *18*, 216–222.
1831. Santamarina-Perez, P.; Eiroa-Orosa, F.J.; Rodriguez-Urrutia, A.; Qureshi, A.; Alegre, J. Neuropsychological impairment in female patients with chronic fatigue syndrome: a preliminary study. *Appl. Neuropsychol. Adult* **2014**, *21*, 120–127.
1832. Sarzi-Puttini, P.; Atzeni, F.; Di Franco, M.; Buskila, D.; Alciati, A.; Giacomelli, C.; Rossi, A.; Bazzichi, L. Dysfunctional syndromes and fibromyalgia: a 2012 critical digest. *Clin. Exp. Rheumatol.* **2012**, *30*, 143–151.

1833. Sathiyamoorthy, S. Ehlers danlos syndrome - A review. *J. Pharm. Sci. Res.* **2016**, *8*, 750–751.
1834. Satterfield, B.C.; Garcia, R.A.; Jia, H.; Tang, S.; Zheng, H.; Switzer, W.M. Serologic and PCR testing of persons with chronic fatigue syndrome in the United States shows no association with xenotropic or polytropic murine leukemia virus-related viruses. *Retrovirology* **2011**, *8*, 12.
1835. Schafer, C.; Evans, M.; Jason, L.A.; So, S.; Brown, A. Measuring substantial reductions in activity. *J. Prev. Interv. Community* **2015**, *43*, 5–19.
1836. Schatzberg, A.F. New indications for antidepressants. *J. Clin. Psychiatry* **2000**, *61 Suppl 11*, 9–17.
1837. Scheeres, K.; Wensing, M.; Severens, H.; Adang, E.; Bleijenberg, G. Determinants of health care use in chronic fatigue syndrome patients: a cross-sectional study. *J. Psychosom. Res.* **2008**, *65*, 39–46.
1838. Scherber, R.M.; Kosiorek, H.E.; Senyak, Z.; Dueck, A.C.; Clark, M.M.; Boxer, M.A.; Geyer, H.L.; McCallister, A.; Cotter, M.; Van Husen, B.; et al. Comprehensively understanding fatigue in patients with myeloproliferative neoplasms. *Cancer* **2016**, *122*, 477–485.
1839. SCHIMATSCHEK, H.F.; CLASSEN, H.G. EPIDEMIOLOGIC STUDIES ON THE FREQUENCY OF HYPOMAGNESEMIA AND HYPOCALCEMIA IN CHILDREN WITH FUNCTIONAL-DISORDERS AND NEURASTHENIA. *MAGNESIUM-BULLETIN* **1993**, *15*, 85–104.
1840. Schmaling, K.B.; Jones, J.F. MMPI profiles of patients with chronic fatigue syndrome. *J. Psychosom. Res.* **1996**, *40*, 67–74.
1841. Schmaling, K.B.; Fiedelak, J.I.; Katon, W.J.; Bader, J.O.; Buchwald, D.S. Prospective study of the prognosis of unexplained chronic fatigue in a clinic-based cohort. *Psychosom. Med.* **2003**, *65*, 1047–1054.
1842. Schmitt, L.; Faure, K. [Discussion]. *Encephale.* **2008**, *34 Spec No 2*, S21-3. Work in French
1843. Schnieders, J.; Willemsen, D.; De Boer, H. Factors contributing to chronic fatigue after traumatic brain injury. *J. Head Trauma Rehabil.* **2012**, *27*, 404–412.
1844. Schochat, T.; Raspe, H. Elements of fibromyalgia in an open population. *Rheumatology* **2003**, *42*, 829–835.
1845. Schondorf, R.; Benoit, J.; Wein, T.; Phaneuf, D. Orthostatic intolerance in the chronic fatigue syndrome. *J. Auton. Nerv. Syst.* **1999**, *75*, 192–201.
1846. Schulte-van Maaren, Y.W.M.; Giltay, E.J.; van Hemert, A.M.; Zitman, F.G.; de Waal, M.W.M.; Van Rood, Y.R.; Carlier, I.V.E. Reference values for the Body Image Concern Inventory (BICI), the Whitely Index (WI), and the Checklist Individual Strength (CIS-20R): The Leiden Routine Outcome Monitoring Study. *J. Affect. Disord.* **2014**, *164*, 82–89.
1847. Schumann, R.; Adamaszek, M.; Sommer, N.; Kirkby, K.C. Stress, depression and antidepressant treatment options in patients suffering from multiple sclerosis. *Curr. Pharm. Des.* **2012**, *18*, 5837–5845.
1848. Schur, E.A.; Noonan, C.; Smith, W.R.; Goldberg, J.; Buchwald, D. Body mass index and fatigue severity in chronic fatigue syndrome. *J. Chronic Fatigue Syndr.* **2007**, *14*, 69–77.
1849. Schur, E.A.; Afari, N.; Furberg, H.; Olarte, M.; Goldberg, J.; Sullivan, P.F.; Buchwald, D. Feeling bad in more ways than one: comorbidity patterns of medically unexplained and psychiatric conditions. *J. Gen. Intern. Med.* **2007**, *22*, 818–821.

1850. Schur, E.; Afari, N.; Goldberg, J.; Buchwald, D.; Sullivan, P.F. Twin analyses of fatigue. *Twin Res. Hum. Genet.* **2007**, *10*, 729–733.
1851. Schurink-van't Klooster, T.M.; Kemmeren, J.M.; van der Maas, N.A.T.; van de Putte, E.M.; ter Wolbeek, M.; Nijhof, S.L.; Vanrolleghem, A.M.; van Vliet, J.A.; Sturkenboom, M.; de Melker, H.E. No evidence found for an increased risk of long-term fatigue following human papillomavirus vaccination of adolescent girls. *Vaccine* **2018**, *36*, 6796–6802.
1852. Schuster, V.; Kreth, H.W. Epstein-Barr virus infection and associated diseases in children. I. Pathogenesis, epidemiology and clinical aspects. *Eur. J. Pediatr.* **1992**, *151*, 718–725.
1853. Schwartz, D.A. Self-reported illness and health status among Gulf War veterans: A population-based study. *J. Am. Med. Assoc.* **1997**, *277*, 238–245.
1854. SCHWARTZ, R.B.; GARADA, B.M.; KOMAROFF, A.L.; TICE, H.M.; GLEIT, M.; JOLESZ, F.A.; HOLMAN, B.L. DETECTION OF INTRACRANIAL ABNORMALITIES IN PATIENTS WITH CHRONIC FATIGUE SYNDROME - COMPARISON OF MR-IMAGING AND SPECT. *Am. J. Roentgenol.* **1994**, *162*, 935–941.
1855. SCHWEITZER, R.; ROBERTSON, D.L.; KELLY, B.; WHITING, J. ILLNESS BEHAVIOR OF PATIENTS WITH CHRONIC FATIGUE SYNDROME. *J. Psychosom. Res.* **1994**, *38*, 41–49.
1856. Scott, L. V; Dinan, T.G. Urinary free cortisol excretion in chronic fatigue syndrome, major depression and in healthy volunteers. *J. Affect. Disord.* **1998**, *47*, 49–54.
1857. Sebastian, A.; Sebastian, M.; Misterska-Skora, M.; Woytala, P.; Jakuszko, K.; Wiland, P. How to Distinguish Patients with pSS among Individuals with Dryness without Invasive Diagnostic Studies. *J. Immunol. Res.* **2018**, *2018*, 1060421.
1858. Secchiero, P.; Berneman, Z.N.; Gallo, R.C.; Lusso, P. Biological and Molecular Characteristics of Human Herpesvirus 7: In Vitro Growth Optimization and Development of a Syncytia Inhibition Test. *Virology* **1994**, *202*, 506–512.
1859. Seet, R.C.S.; Quek, A.M.L.; Lim, E.C.H. Post-infectious fatigue syndrome in dengue infection. *J. Clin. Virol.* **2007**, *38*, 1–6.
1860. Seishima, M.; Mizutani, Y.; Shibuya, Y.; Arakawa, C. Chronic fatigue syndrome after human parvovirus B19 infection without persistent viremia. *Dermatology* **2008**, *216*, 341–346.
1861. Selden, S.M.; Cameron, A.S. Changing epidemiology of Ross River virus disease in South Australia. *Med. J. Aust.* **1996**, *165*, 313–317.
1862. Sène, D.; Saadoun, D.; Limal, N.; Piette, J.-C.; Cacoub, P. Update in Hepatitis C virus associated extrahepatic manifestations [Actualités des manifestations extrahépatiques associées au virus de l'hépatite C]. *Rev. Med. Interne* **2007**, *28*, 388–393. Work in French
1863. Sepede, G.; Racciatti, D.; Gorgoretti, V.; Nacci, M.; Pizzigallo, E.; Onofrj, M.; Di Giannantonio, M.; Niolu, C.; Salerno, R.M.; Gambi, F. Psychophysical distress and alexithymic traits in chronic fatigue syndrome with and without comorbid depression. *Int. J. Immunopathol. Pharmacol.* **2011**, *24*, 1017–1025.
1864. Servaes, P.; van der Werf, S.; Prins, J.; Verhagen, S.; Bleijenberg, G. Fatigue in disease-free cancer patients compared with fatigue in patients with chronic fatigue syndrome. *Support. care cancer Off. J. Multinatl. Assoc. Support. Care Cancer* **2001**, *9*, 11–17.

1865. Servaes, P.; Verhagen, S.; Bleijenberg, G. Determinants of chronic fatigue in disease-free breast cancer patients: A cross-sectional study. *Ann. Oncol.* **2002**, *13*, 589–598.
1866. Sesiuk, A.; Rzepiela, L. Selected psychiatric disorders in patients with cancer [Wybrane zaburzenia psychiczne w przebiegu chorób nowotworowych]. *Psychiatr. i Psychol. Klin.* **2016**, *16*, 21–26. Work in Polish
1867. Sevel, L.S.; Boissoneault, J.; Letzen, J.E.; Robinson, M.E.; Staud, R. Structural brain changes versus self-report: machine-learning classification of chronic fatigue syndrome patients. *Exp. BRAIN Res.* **2018**, *236*, 2245–2253.
1868. Shadili, G.; Goumard, D.; Provoost, J.-P.; Le Pallec, G. The "burn-out" or chronic fatigue syndrome of physicians and other hospital practitioners [Burn out des médecins et autres praticiens hospitaliers]. *Inf. Psychiatr.* **2018**, *94*, 13–18. Work in French
1869. Shafran, S.D. The chronic fatigue syndrome. *Am. J. Med.* **1991**, *90*, 730–739.
1870. Shahar, E.; Lederer, J. Asthenic symptoms in a rural family practice: Epidemiologic characteristics and a proposed classification. *J. Fam. Pract.* **1990**, *31*, 257–262.
1871. Shanks, M.F.; Ho-Yen, D.O. A clinical study of chronic fatigue syndrome. *Br. J. Psychiatry* **1995**, *166*, 798–801.
1872. Shannon, M.; Clovis, L.L. Health care workers, predominant gender females at high risk: Turning the spotlight on the endocrine system. *J. Chronic Fatigue Syndr.* **2000**, *7*, 75–91.
1873. Shapiro, J.S. Does varicella-zoster virus infection of the peripheral ganglia cause Chronic Fatigue Syndrome? *Med. Hypotheses* **2009**, *73*, 728–734.
1874. Sharma, O.P. Fatigue and sarcoidosis. *Eur. Respir. J.* **1999**, *13*, 713–714.
1875. Sharpe, M. Chronic fatigue syndrome. *Medicine (Baltimore).* **2008**, *36*, 452–454.
1876. Sharpe, M. The symptom of generalised fatigue. *Pract. Neurol.* **2006**, *6*, 72–77.
1877. Sharpe, M.; Wilks, D. Fatigue. *BMJ* **2002**, *325*, 480.
1878. Shaw, C.A.; Li, Y.; Tomljenovic, L. Administration of aluminium to neonatal mice in vaccine-relevant amounts is associated with adverse long term neurological outcomes. *J. Inorg. Biochem.* **2013**, *128*, 237–244.
1879. Shefer, A.; Dobbins, J.G.; Fukuda, K.; Steele, L.; Koo, D.; Nisenbaum, R.; Rutherford, G.W. Fatiguing illness among employees in three large state office buildings, California, 1993: Was there an outbreak? *J. Psychiatr. Res.* **1997**, *31*, 31–43. Proceedings of the 1st Research and Clinical Conference of the American-Association-for-Chronic-Fatigue-Syndrome in FT Lauderdale, USA.
1880. Shephard, R.J. Chronic fatigue syndrome: an update. *Sports Med.* **2001**, *31*, 167–194.
1881. Shepherd, C. Disagreements still exist over the chronic fatigue syndrome [6]. *Br. Med. J.* **1997**, *314*, 146.
1882. Shepherd, C.; Lees, H. ME: is it a genuine disease? *Health Visit.* **1992**, *65*, 165–167.
1883. Shepherd, C. The debate: myalgic encephalomyelitis and chronic fatigue syndrome. *Br. J. Nurs.* **2006**, *15*, 662–669.
1884. Shi, J.; Shen, J.; Xie, J.; Zhi, J.; Xu, Y. Chronic fatigue syndrome in Chinese middle-school students. *Medicine (Baltimore).* **2018**, *97*, e9716.
1885. Shih, Y.-Y.; Lee, S.-D.; Kuo, S.-D. An investigation to somatic symptoms, psychiatric symptoms and personality of chronic fatigue patients. *Taiwan J. Public Heal.* **2003**, *22*, 362–367.

1886. Shin, C.H.; Bateman, L.; Schlaberg, R.; Bunker, A.M.; Leonard, C.J.; Hughen, R.W.; Light, A.R.; Light, K.C.; Singh, I.R. Absence of XMRV Retrovirus and Other Murine Leukemia Virus-Related Viruses in Patients with Chronic Fatigue Syndrome. *J. Virol.* **2011**, *85*, 7195–7202.
1887. Shinchuk, L.; Holick, M.F. Vitamin D and rehabilitation: Improving functional outcomes. *Nutr. Clin. Pract.* **2007**, *22*, 297–304.
1888. Shipton, E.A. Controversies, new risk factors and the neuroendocrine approach to fibromyalgia: Can therapeutic progress be made at last? *N. Z. Med. J.* **2003**, *116*.
1889. Shirom, A. Reflections on the study of burnout. *Work Stress* **2005**, *19*, 263–270.
1890. Shuttleworth, A. Understanding chronic fatigue. *Nurs. Times* **2006**, *102*, 20–21.
1891. Siddiqui, M.Z. Gulf War syndrome. *Cleve. Clin. J. Med.* 2000, *67*, 510.
1892. Siegel, Z.A.; Brown, A.; Devendorf, A.; Collier, J.; Jason, L.A. A content analysis of chronic fatigue syndrome and myalgic encephalomyelitis in the news from 1987 to 2013. *Chronic Illn.* **2018**, *14*, 3–12.
1893. Siegert, R.J.; Abernethy, D.A. Depression in multiple sclerosis: a review. *J. Neurol. Neurosurg. PSYCHIATRY* **2005**, *76*, 469–475.
1894. Simoens, S.; D'Hooghe, T. *Economic aspects of diagnosis and treatment of endometriosis*; 2010;
1895. Şimşek, I.; Simsek, I. Irritable bowel syndrome and other functional gastrointestinal disorders. *J. Clin. Gastroenterol.* **2011**, *45*, S86–S88. Proceedings of the Conferencia: Pan-European Conference on Irritable Bowel Syndrome in Vienna, Austria
1896. Sinaii, N.; Cleary, S.D.; Ballweg, M.L.; Nieman, L.K.; Stratton, P. High rates of autoimmune and endocrine disorders, fibromyalgia, chronic fatigue syndrome and atopic diseases among women with endometriosis: a survey analysis. *Hum. Reprod.* **2002**, *17*, 2715–2724.
1897. Singh, B.B.; Wu, W.S.; Hwang, S.H.; Khorsan, R.; Der-Martirosian, C.; Vinjamury, S.P.; Wang, C.N.; Lin, S.Y. Effectiveness of acupuncture in the treatment of fibromyalgia. *Altern. Ther. Health Med.* **2006**, *12*, 34–41.
1898. Singh, B.B.; Khorsan, R.; Vinjamury, S.P. Influence of comorbidities on improvement of fibromyalgia symptoms when treated with acupuncture: a short report. *Altern. Ther. Health Med.* **2008**, *14*, 24–25.
1899. Singh, I.R.; Gorzynski, J.E.; Drobysheva, D.; Bassit, L.; Schinazi, R.F. Raltegravir Is a Potent Inhibitor of XMRV, a Virus Implicated in Prostate Cancer and Chronic Fatigue Syndrome. *PLoS One* **2010**, *5*.
1900. Siniscalchi, M.; Iovino, P.; Tortora, R.; Forestiero, S.; Somma, A.; Capuano, L.; Franzese, M.D.; Sabbatini, F.; Ciacci, C. Fatigue in adult coeliac disease. *Aliment. Pharmacol. Ther.* **2005**, *22*, 489–494.
1901. Sirois, D.A.; Natelson, B. Clinicopathological findings consistent with primary Sjögren's syndrome in a subset of patients diagnosed with chronic fatigue syndrome: Preliminary observations. *J. Rheumatol.* **2001**, *28*, 126–131.
1902. Sirois, F.M.; Molnar, D.S. Perfectionism and maladaptive coping styles in patients with chronic fatigue syndrome, irritable bowel syndrome and fibromyalgia/arthritis and in healthy controls. *Psychother. Psychosom.* 2014, *83*, 384–385.
1903. Siu, Y.-F.; Chan, S.; Wong, K.-M.; Wong, W.-S. The Comorbidity of Chronic Pain and Sleep Disturbances in a Community Adolescent Sample: Prevalence and Association with Sociodemographic and Psychosocial Factors. *PAIN Med.* **2012**, *13*, 1292–1303.

1904. Skapinakis, P.; Lewis, G.; Mavreas, V. Cross-cultural differences in the epidemiology of unexplained fatigue syndromes in primary care. *Br. J. Psychiatry* **2003**, *182*, 205–209.
1905. Skapinakis, P.; Lewis, G.; Meltzer, H. Clarifying the relationship between unexplained chronic fatigue and psychiatric morbidity: results from a community survey in Great Britain. *Am. J. Psychiatry* **2003**, *15*, 57–64.
1906. Skapinakis, P.; Lewis, G.; Mavreas, V. Temporal relations between unexplained fatigue and depression: longitudinal data from an international study in primary care. *Psychosom. Med.* **2004**, *66*, 330–335.
1907. Skapinakis, P.; Lewis, G.; Mavreas, V. Unexplained fatigue syndromes in a multinational primary care sample: specificity of definition and prevalence and distinctiveness from depression and generalized anxiety. *Am. J. Psychiatry* **2003**, *160*, 785–787.
1908. Skovbjerg, S.; Brorson, S.; Rasmussen, A.; Johansen, J.D.; Elberling, J. Impact of self-reported multiple chemical sensitivity on everyday life: A qualitative study. *Scand. J. Public Health* **2009**, *37*, 621–626.
1909. Skowera, A.; Peakman, M.; Cleare, A.; Davies, E.; Deale, A.; Wessely, S. High prevalence of serum markers of coeliac disease in patients with chronic fatigue syndrome [2]. *J. Clin. Pathol.* **2001**, *54*, 335–336.
1910. Skowera, A.; Stewart, E.; Davis, E.T.; Cleare, A.J.; Unwin, C.; Hull, L.; Ismail, K.; Hossain, G.; Wessely, S.C.; Peakman, M. Antinuclear autoantibodies (ANA) in Gulf War-related illness and chronic fatigue syndrome (CFS) patients. *Clin. Exp. Immunol.* **2002**, *129*, 354–358.
1911. Skufca, J.; Ollgren, J.; Ruokokoski, E.; Lyytikäinen, O.; Nohynek, H. Incidence rates of Guillain Barré (GBS), chronic fatigue/systemic exertion intolerance disease (CFS/SEID) and postural orthostatic tachycardia syndrome (POTS) prior to introduction of human papilloma virus (HPV) vaccination among adolescent girls in Finland, 2002–2012. *Papillomavirus Res.* **2017**, *3*, 91–96.
1912. Slomko, J.; Newton, J.L.; Kujawski, S.; Tafil-Klawe, M.; Klawe, J.; Staines, D.; Marshall-Gradisnik, S.; Zalewski, P. Prevalence and characteristics of chronic fatigue syndrome/myalgic encephalomyelitis (CFS/ME) in Poland: A cross-sectional study. *BMJ Open* **2019**, *9*.
1913. Slade, G.D.; Sanders, A.E.; By, K. Role of Allostatic Load in Sociodemographic Patterns of Pain Prevalence in the US Population. *J. PAIN* **2012**, *13*, 666–675.
1914. Slavkin, H.C. Distinguishing Mars from Venus: emergence of gender biology differences in oral health and systemic disease. *Compend. Contin. Educ. Dent.* **2002**, *23*, 29–31.
1915. Slyepchenko, A.; Maes, M.; Machado-Vieira, R.; Anderson, G.; Solmik, M.; Sanz, Y.; Berk, M.; Köhlerp, C.A.; Carvalhop, A.F. Intestinal dysbiosis, gut hyperpermeability and bacterial translocation: Missing links between depression, obesity and type 2 diabetes? *Curr. Pharm. Des.* **2016**, *22*, 6087–6106.
1916. Smith, A.P.; Thomas, M.A. Chronic fatigue syndrome and increased susceptibility to upper respiratory tract infections and illnesses. *FATIGUE-BIOMEDICINE Heal. Behav.* **2015**, *3*, 156–163.
1917. Smith, B.H.; Macfarlane, G.J.; Torrance, N. Epidemiology of chronic pain, from the laboratory to the bus stop: time to add understanding of biological mechanisms to the study of risk factors in population-based research? *Pain* **2007**, *127*, 5–10.
1918. Smith, B.N.; Wang, J.M.; Vogt, D.; Vickers, K.; King, D.W.; King, L.A. Gulf War Illness Symptomatology Among Veterans 10 Years After Deployment. *J. Occup. Environ. Med.* **2013**, *55*, 116–122.

1919. Smith, B.W.; Papp, Z.Z.; Tooley, E.M.; Montague, E.Q.; Robinson, A.E.; Cosper, C.J. Traumatic Events, Perceived Stress and Health in Women with Fibromyalgia and Healthy Controls. *Stress Heal.* **2010**, *26*, 83–93.
1920. Smith, M.S. Adolescent chronic fatigue syndrome. *Arch. Pediatr. Adolesc. Med.* **2004**, *158*, 207–208.
1921. Smith, M.S.; Buchwald, D.S.; Bogart, A.; Goldberg, J.; Smith, W.R.; Afari, N. Adolescent offspring of mothers with chronic fatigue syndrome. *J. Adolesc. Health* **2010**, *46*, 284–291.
1922. Smith, M.S.; Martin-Herz, S.P.; Womack, W.M.; Marsigan, J.L. Comparative study of anxiety, depression, somatization, functional disability, and illness attribution in adolescents with chronic fatigue or migraine. *Pediatrics* **2003**, *111*, e376-81.
1923. Smith, R.A. Contamination of clinical specimens with MLV-encoding nucleic acids: Implications for XMRV and other candidate human retroviruses. *Retrovirology* **2010**, *7*.
1924. Smith, R.D.; Scott, A. The economic impact of chronic fatigue syndrome. *Med. J. Aust.* 1993, *158*, 286–287.
1925. Smith, W.R.; Noonan, C.; Buchwald, D. Mortality in a cohort of chronically fatigued patients. *Psychol. Med.* **2006**, *36*, 1301–1306.
1926. Smorgick, N.; Marsh, C.A.; As-Sanie, S.; Smith, Y.R.; Quint, E.H. Prevalence of pain syndromes, mood conditions, and asthma in adolescents and young women with endometriosis. *J. Pediatr. Adolesc. Gynecol.* **2013**, *26*, 171–175.
1927. Snekkevik, H.; Eriksen, H.R.; Tangen, T.; Chalder, T.; Reme, S.E. Fatigue and Depression in Sick-Listed Chronic Low Back Pain Patients. *Pain Med. (United States)* **2014**, *15*, 1163–1170.
1928. Snodgrass, K.; Harvey, A.; Scheinberg, A.; Knight, S. Sleep Disturbances in Pediatric Chronic Fatigue Syndrome: A Review of Current Research. *J. Clin. sleep Med. JCSM Off. Publ. Am. Acad. Sleep Med.* **2015**, *11*, 757–764.
1929. Soderholm, A.; Ohman, A.; Stenberg, B.; Nordin, S. Experience of living with nonspecific building-related symptoms. *Scand. J. Psychol.* **2016**, *57*, 406–412.
1930. Sokołowski, Ł.; Ukleja-Sokołowska, N.; Zalewski, P.; Bartuzi, Z. Is allergy a risk factor of chronic fatigue syndrome? [Czy alergia stanowi czynnik ryzyka zespołu przewlekłego zmęczenia?]. *Alerg. Astma Immunol.* **2016**, *21*, 96–101. Work in Polish
1931. Sokolowski, L.; Ukleja-Sokolowska, N.; Zalewski, P.; Bartuzi, Z. Is allergy a risk factor of chronic fatigue syndrome? *Alerg. ASTMA Immunol.* **2016**, *21*, 96–101.
1932. Solomon, L.; Reeves, W.C. Factors influencing the diagnosis of chronic fatigue syndrome. *Arch. Intern. Med.* **2004**, *164*, 2241–2245.
1933. Sordet, C. Chronic lyme disease: Fact or fiction? *Jt. Bone Spine* **2014**, *81*, 110–111.
1934. Soriano, V. [XMRV, a new human retrovirus for disease]. *Med. Clin. (Barc).* 2011, *136*, 669–670. Work in Spanish
1935. Soriano, V.; Trevino, A. [New human retrovirus]. *Med. Clin. (Barc).* 2010, *135*, 65–66. Work in Spanish
1936. Spaeth, M. Fibromyalgia review. *J. Musculoskelet. Pain* **2007**, *15*, 75–81.
1937. Spain, L.A.; Tubridy, N.; Kilpatrick, T.J.; Adams, S.J.; Holmes, A.C.N. Illness perception and health-related quality of life in multiple sclerosis. *Acta Neurol. Scand.* **2007**, *116*, 293–299.
1938. Speer, L.M.; Mushkbar, S.; Erbele, T. Chronic Pelvic Pain in Women. *Am. Fam. Physician* **2016**, *93*, 380–387.

1939. Spence, M.; Moss-Morris, R.; Chalder, T. The Behavioural Responses to Illness Questionnaire (BRIQ): a new predictive measure of medically unexplained symptoms following acute infection. *Psychol. Med.* **2005**, *35*, 583–593.
1940. Spindler, J.; Hackett, J.; Qiu, X.; Wiegand, A.; Boltz, V.F.; Swanson, P.; Bream, J.H.; Jacobson, L.P.; Li, X.; Rinaldo, C.R.; et al. Prevalence of XMRV nucleic acid and antibody in HIV-1-infected men and in men at risk for HIV-1 infection. *Adv. Virol.* **2011**, *2011*.
1941. Spitzer, A.R.; Broadman, M. A Retrospective Review of the Sleep Characteristics in Patients with Chronic Fatigue Syndrome and Fibromyalgia. *PAIN Pract.* **2010**, *10*, 294–311.
1942. Stadje, R.; Dornieden, K.; Baum, E.; Becker, A.; Biroga, T.; Bösner, S.; Haasenritter, J.; Keunecke, C.; Viniol, A.; Donner-Banzhoff, N. The differential diagnosis of tiredness: A systematic review. *BMC Fam. Pract.* **2016**, *17*.
1943. Staeheli, P.; Sauder, C.; Hausmann, J.; Ehrensperger, F.; Schwemmle, M. Epidemiology of Borna disease virus. *J. Gen. Virol.* **2000**, *81*, 2123–2135.
1944. Stanaway, J.D.; Shepard, D.S.; Undurraga, E.A.; Halasa, Y.A.; Coffeng, L.E.; Brady, O.J.; Hay, S.I.; Bedi, N.; Bensenor, I.M.; Castañeda-Orjuela, C.A.; et al. The global burden of dengue: an analysis from the Global Burden of Disease Study 2013. *Lancet Infect. Dis.* **2016**, *16*, 712–723.
1945. Stanley, I.; Salmon, P.; Peters, S. Doctors and social epidemics: the problem of persistent unexplained physical symptoms, including chronic fatigue. *Br. J. Gen. Pract.* 2002, *52*, 355–356.
1946. Starcevic, V. Neurasthenia: Cross-cultural and conceptual issues in relation to chronic fatigue syndrome. *Gen. Hosp. Psychiatry* **1999**, *21*, 249–255.
1947. Steele, L. Prevalence and patterns of Gulf War illness in Kansas veterans: Association of symptoms with characteristics of person, place, and time of military service. *Am. J. Epidemiol.* **2000**, *152*, 992–1002.
1948. Steele, L.; Dobbins, J.G.; Fukuda, K.; Reyes, M.; Randall, B.; Koppelman, M.; Reeves, W.C. The epidemiology of chronic fatigue in San Francisco. *Am. J. Med.* **1998**, *105*, 83S–90S.
1949. Steere, A.C. A 58-year-old man with a diagnosis of chronic lyme disease. *JAMA* **2002**, *288*, 1002–1010.
1950. Stefansson, C.-G. Chapter 5.5: Major public health problems - Mental ill-health. *Scand. J. Public Health* **2006**, *34*, 87–103.
1951. Stein, E.; MacQuarrie, M. Myalgic encephalomyelitis/chronic fatigue syndrome (ME/CFS) program and interdisciplinary research symposium on disabling fatigue in chronic illness. *Chronic Dis. Can.* 2009, *29*, 136–138.
1952. Steinau, M.; Unger, E.R.; Vernon, S.D.; Jones, J.F.; Rajeevan, M.S. Differential-display PCR of peripheral blood for biomarker discovery in chronic fatigue syndrome. *J. Mol. Med. (Berl).* **2004**, *82*, 750–755.
1953. Steinberg, P.; McNutt, B.E.; Marshall, P.; Schenck, C.; Lurie, N.; Pheley, A.; Peterson, P.K. Double-blind placebo-controlled study of the efficacy of oral terfenadine in the treatment of chronic fatigue syndrome. *J. Allergy Clin. Immunol.* **1996**, *97*, 119–126.
1954. Stenager, E.; Svendsen, M.A.; Stenager, E. Disability retirement pension for patients with syndrome diagnoses. A register study on the basis of data from the social appeal board [Førtidspension til patienter med syndromsygdomme: En registerundersogelse på basis af oplysninger fra Den Sociale Ankestyrelse]. *Ugeskr. Laeger* **2003**, *165*, 469–474. Work in Danish

1955. Stenlund, T.; Ahlgren, C.; Lindahl, B.; Burell, G.; Knutsson, A.; Stegmayr, B.; Birgander, L.S. Patients with burnout in relation to gender and a general population. *Scand. J. Public Health* **2007**, *35*, 516–523.
1956. Sterzl, I.; Fucíková, T.; Hrdá, P.; Matucha, P.; Zamrazil, V. The fatigue syndrome in autoimmune thyroiditis with polyglandular activation of autoimmunity [Unavový syndrom u autoimunitní tyroiditity s polyglandulární aktivací autoimunity.]. *Vnitr. lékarství* **1998**, *44*, 456–460. Work in Czech
1957. Sterzl, I.; Hrdá, P.; Procházková, J.; Bártová, J.; Matucha, P. Reactions to metals in patients with chronic fatigue and autoimmune endocrinopathy [Reakce na kovy u pacientů s chronickou únavou a autoimunitními endokrinopatiemi.]. *Vnitr. lékarství* **1999**, *45*, 527–531. Work in Czech
1958. Stewart, D.; Abbey, S.; Meana, M.; Boydell, K.M. What makes women tired? A community sample. *J. WOMENS Heal.* **1998**, *7*, 69–76.
1959. Stewart, J.M.; Gewitz, M.H.; Weldon, A.; Munoz, J. Patterns of orthostatic intolerance: the orthostatic tachycardia syndrome and adolescent chronic fatigue. *J. Pediatr.* **1999**, *135*, 218–225.
1960. Stieler, K.; Schindler, S.; Schlomm, T.; Hohn, O.; Bannert, N.; Simon, R.; Minner, S.; Schindler, M.; Fischer, N. No Detection of XMRV in Blood Samples and Tissue Sections from Prostate Cancer Patients in Northern Europe. *PLoS One* **2011**, *6*.
1961. Stoll, S.V.E.; Crawley, E.; Richards, V.; Lal, N.; Brigden, A.; Loades, M.E. What treatments work for anxiety in children with chronic fatigue syndrome/myalgic encephalomyelitis (CFS/ME)? Systematic review. *BMJ Open* **2017**, *7*.
1962. Stone, C.A.; Kenny, R.A.; Nolan, B.; Lawlor, P.G. Autonomic dysfunction in patients with advanced cancer; prevalence, clinical correlates and challenges in assessment. *BMC Palliat. Care* **2012**, *11*.
1963. Stone, K.C.; Taylor, D.J.; McCrae, C.S.; Kalsekar, A.; Lichstein, K.L. Nonrestorative sleep. *Sleep Med. Rev.* **2008**, *12*, 275–288.
1964. Stormorken, E.; Jason, L.A.; Kirkevold, M. Factors impacting the illness trajectory of post-infectious fatigue syndrome: a qualitative study of adults' experiences. *BMC Public Health* **2017**, *17*, 952.
1965. Stormorken, E.; Jason, L.A.; Kirkevold, M. From good health to illness with post-infectious fatigue syndrome: a qualitative study of adults' experiences of the illness trajectory. *BMC Fam. Pract.* **2017**, *18*, 49.
1966. Stoye, J.P.; Silverman, R.H.; Boucher, C.A.; Le Grice, S.F.J. The xenotropic murine leukemia virus-related retrovirus debate continues at first international workshop. *Retrovirology* **2010**, *7*.
1967. Stranden, M.; Solvin, H.; Fors, E.A.; Getz, L.; Helvik, A.-S. Are persons with fibromyalgia or other musculoskeletal pain more likely to report hearing loss? A HUNT study. *BMC Musculoskelet. Disord.* **2016**, *17*.
1968. Strassheim, V.; Welford, J.; Ballantine, R.; Newton, J.L. Managing fatigue in postural tachycardia syndrome (PoTS): The Newcastle approach. *Auton. Neurosci. Basic Clin.* **2018**, *215*, 56–61.
1969. Strassheim, V.J.; Sunnquist, M.; Jason, L.A.; Newton, J.L. Defining the prevalence and symptom burden of those with self-reported severe chronic fatigue syndrome/myalgic encephalomyelitis (CFS/ME): a two-phase community pilot study in the North East of England. *BMJ Open* **2018**, *8*.
1970. Straus, S.E. Caring for patients with chronic fatigue syndrome: Conclusions in CMO's report are shaped by anecdote not evidence. *Br. Med. J.* **2002**, *324*, 124–125.

1971. Straus, S.E. Chronic fatigue syndrome. *Br. Med. J.* **1996**, *313*, 831–832.
1972. Straus, S.E. Studies of herpesvirus infection in chronic fatigue syndrome. *Ciba Found. Symp.* **1993**, *173*, 132–145.
1973. Straus, S.E.; Fritz, S.; Dale, J.K.; Gould, B.; Strober, W. Lymphocyte phenotype and function in the chronic fatigue syndrome. *J. Clin. Immunol.* **1993**, *13*, 30–40.
1974. Strauss, B.; Loschau, M.; Seidel, T.; Stallmach, A.; Thomas, A. Are fatigue symptoms and chronic fatigue syndrome following Q fever infection related to psychosocial variables? *J. Psychosom. Res.* **2012**, *72*, 300–304.
1975. Streeten, D.H.P.; Bell, D.S. Circulating blood volume in chronic fatigue syndrome. *J. Chronic Fatigue Syndr.* **1998**, *4*, 3–11.
1976. Streeten, D.H.; Anderson, G.H.J. The role of delayed orthostatic hypotension in the pathogenesis of chronic fatigue. *Clin. Auton. Res.* **1998**, *8*, 119–124.
1977. Stricker, R.B.; Lautin, A.; Burrascano, J.J. Lyme disease: Point/counterpoint. *Expert Rev. Anti. Infect. Ther.* **2005**, *3*, 155–165.
1978. Struyf, F.; Meeus, M. Current evidence on physical therapy in patients with adhesive capsulitis: what are we missing? *Clin. Rheumatol.* **2014**, *33*, 593–600.
1979. Sudlow, C.; Macleod, M.; Al-Shahi Salman, R.; Stone, J. Comment on "Detection of an infectious retrovirus, XMRV, in blood cells of patients with chronic fatigue syndrome". *Science* **2010**, *328*, 825; author reply 825.
1980. Suhadolnik, R.J.; Peterson, D.L.; Reichenbach, N.L.; Roen, G.; Metzger, M.; McCahan, J.; O'Brien, K.; Welsch, S.; Gabriel, J.; Gaughan, J.P.; et al. Clinical and biochemical characteristics differentiating chronic fatigue syndrome from major depression and healthy control populations: Relation to dysfunction in the RNase L pathway. *J. Chronic Fatigue Syndr.* **2004**, *12*, 5–35.
1981. Sulheim, D.; Fagermoen, E.; Sivertsen, O.S.; Winger, A.; Wyller, V.B.; Oie, M.G. Cognitive dysfunction in adolescents with chronic fatigue: a cross-sectional study. *Arch. Dis. Child.* **2015**, *100*, 838–844.
1982. Sulheim, D.; Fagermoen, E.; Winger, A.; Andersen, A.M.; Godang, K.; Muller, F.; Rowe, P.C.; Saul, J.P.; Skovlund, E.; Oie, M.G.; et al. Disease mechanisms and clonidine treatment in adolescent chronic fatigue syndrome: a combined cross-sectional and randomized clinical trial. *JAMA Pediatr.* **2014**, *168*, 351–360.
1983. Sullivan, P.F.; Kovalenko, P.; York, T.P.; Prescott, C.A.; Kendler, K.S. Fatigue in a community sample of twins. *Psychol. Med.* **2003**, *33*, 263–281.
1984. Sullivan, P.F.; Allander, T.; Lysholm, F.; Goh, S.; Persson, B.; Jacks, A.; Evengard, B.; Pedersen, N.L.; Andersson, B. An unbiased metagenomic search for infectious agents using monozygotic twins discordant for chronic fatigue. *BMC Microbiol.* **2011**, *11*, 2.
1985. Sullivan, P.F.; Evengard, B.; Jacks, A.; Pedersen, N.L. Twin analyses of chronic fatigue in a Swedish national sample. *Psychol. Med.* **2005**, *35*, 1327–1336.
1986. Sullivan, P.F.; Pedersen, N.L.; Jacks, A.; Evengard, B. Chronic fatigue in a population sample: definitions and heterogeneity. *Psychol. Med.* **2005**, *35*, 1337–1348.
1987. SUMAYA, C. V SEROLOGIC AND VIROLOGICAL EPIDEMIOLOGY OF EPSTEIN-BARR-VIRUS - RELEVANCE TO CHRONIC FATIGUE SYNDROME. *Rev. Infect. Dis.* **1991**, *13*, S19–S25.
1988. Sumaya, C. V Serologic and virologic epidemiology of Epstein-Barr virus: relevance to chronic fatigue syndrome. *Rev. Infect. Dis.* **1991**, *13 Suppl 1*, S19-25.
1989. Sundelin, G. Exercise as therapy in chronic diseases. *Adv. Physiother.* **2006**, *8*, 49.

1990. Surrey, E.S.; Soliman, A.M.; Johnson, S.J.; Davis, M.; Castelli-Haley, J.; Snabes, M.C. Risk of Developing Comorbidities Among Women with Endometriosis: A Retrospective Matched Cohort Study. *J. Women's Heal.* **2018**, *27*, 1114–1123.
1991. Suskind, A.M.; Berry, S.H.; Suttorp, M.J.; Elliott, M.N.; Hays, R.D.; Ewing, B.A.; Clemens, J.Q. Health-related quality of life in patients with interstitial cystitis/bladder pain syndrome and frequently associated comorbidities. *Qual. Life Res.* **2013**, *22*, 1537–1541.
1992. Sutton, G.C. "Too tired to go to the support group": a health needs assessment of myalgic encephalomyelitis. *J. Public Health Med.* **1996**, *18*, 343–349.
1993. Suzuki, S. Exhausting Physicians Employed in Hospitals in Japan Assessed by a Health Questionnaire. *Sangyo Eiseigaku Zasshi* **2017**, *59*, 107–118.
1994. Swanink, C.M.A.; Stolk-Engelaar, V.M.M.; Van Der Meer, J.W.M.; Vercoulen, J.H.M.M.; Bleijenberg, G.; Fennis, J.F.M.; Galama, J.M.D.; Hoogkamp-Korstanje, J.A.A. Yersinia enterocolitica and the chronic fatigue syndrome. *J. Infect.* **1998**, *36*, 269–272.
1995. Sweetman, B.J. Chronic fatigue syndrome in mother and child. *J. R. Soc. Med.* **2000**, *93*, 337–338.
1996. Swenson, T.S. Chronic fatigue syndrome. *J. Rehabil.* **2000**, *66*, 37–42.
1997. Swinkels, D.W.; Aalbers, N.; Elving, L.D.; Bleijenberg, G.; Swanink, C.M.A.; van der Meer, J.W.M. Primary haemochromatosis: a missed cause of chronic fatigue syndrome? *Neth. J. Med.* **2002**, *60*, 429–433.
1998. Switzer, W.M.; Jia, H.; Hohn, O.; Zheng, H.; Tang, S.; Shankar, A.; Bannert, N.; Simmons, G.; Hendry, R.M.; Falkenberg, V.R.; et al. Absence of evidence of Xenotropic Murine Leukemia Virus-related virus infection in persons with Chronic Fatigue Syndrome and healthy controls in the United States. *Retrovirology* **2010**, *7*.
1999. Switzer, W.M.; Jia, H.; Zheng, H.; Tang, S.; Heneine, W. No Association of Xenotropic Murine Leukemia Virus-Related Viruses with Prostate Cancer. *PLoS One* **2011**, *6*.
2000. Swoboda, D.A. The social construction of contested illness legitimacy: A grounded theory analysis. *Qual. Res. Psychol.* **2006**, *3*, 233–251.
2001. Syx, D.K.; De Wandele, I.; Rombaut, L.; Malfait, F. Hypermobility, the Ehlers-Danlos syndromes and chronic pain. *Clin. Exp. Rheumatol.* **2017**, *35*, S116–S122.
2002. Szarek, M.J.; Bell, I.R.; Schwartz, G.E. Validation of a brief screening measure of environmental chemical sensitivity: The chemical odor intolerance index. *J. Environ. Psychol.* **1997**, *17*, 345–351.
2003. Szer, I.S. Psychogenic rheumatism in adolescents. *Drug Benefit Trends* **2002**, *14*, 32–36.
2004. Tahmaz, N.; Soutar, A.; Cherrie, J.W. Chronic fatigue and organophosphate pesticides in sheep farming: A retrospective study amongst people reporting to a UK pharmacovigilance scheme. *Ann. Occup. Hyg.* **2003**, *47*, 261–267.
2005. Taillefer, S.S.; Kirmayer, L.J.; Robbins, J.M.; Lasry, J.C. Correlates of illness worry in chronic fatigue syndrome. *J. Psychosom. Res.* **2003**, *54*, 331–337.
2006. Takada, M.; Nishida, K.; Kataoka-Kato, A.; Gondo, Y.; Ishikawa, H.; Suda, K.; Kawai, M.; Hoshi, R.; Watanabe, O.; Igarashi, T.; et al. Probiotic Lactobacillus casei strain Shirota relieves stress-associated symptoms by modulating the gut-brain interaction in human and animal models. *Neurogastroenterol. Motil.* **2016**, *28*, 1027–1036.

2007. Tanaka, H.; Matsushima, R.; Tamai, H.; Kajimoto, Y. Impaired postural cerebral hemodynamics in young patients with chronic fatigue with and without orthostatic intolerance. *J. Pediatr.* **2002**, *140*, 412–417.
2008. Tanaka, M.; Fukuda, S.; Mizuno, K.; Imai-Matsumura, K.; Jodoi, T.; Kawatani, J.; Takano, M.; Miike, T.; Tomoda, A.; Watanabe, Y. Reliability and validity of the Japanese version of the Chalder Fatigue Scale among youth in Japan. *Psychol. Rep.* **2008**, *103*, 682–690.
2009. Tanaka, S.; Kuratsune, H.; Hidaka, Y.; Hakariya, Y.; Tatsumi, K.I.; Takano, T.; Kanakura, Y.; Amino, N. Autoantibodies against muscarinic cholinergic receptor in chronic fatigue syndrome. *Int. J. Mol. Med.* **2003**, *12*, 225–230.
2010. Tang, S.; Zhao, J.; Viswanath, R.; Nyambi, P.N.; Redd, A.D.; Dastyar, A.; Spacek, L.A.; Quinn, T.C.; Wang, X.; Wood, O.; et al. Absence of detectable xenotropic murine leukemia virus-related virus in plasma or peripheral blood mononuclear cells of human immunodeficiency virus Type 1-infected blood donors or individuals in Africa. *Transfusion* **2011**, *51*, 463–468.
2011. Tang, S.; Zhao, J.; Setty, M.K.H.G.; Devadas, K.; Gaddam, D.; Viswanath, R.; Wood, O.; Zhang, P.; Hewlett, I.K. Absence of Detectable XMRV and Other MLV-Related Viruses in Healthy Blood Donors in the United States. *PLoS One* **2011**, *6*.
2012. Tarello, W. Immunological anomalies and thrombocytopenia in 117 dogs and cats diagnosed with chronic fatigue syndrome (CFS). *Acta Vet. Hung.* **2003**, *51*, 61–72.
2013. Tattam, A. CFS--an occupational hazard for nurses? *Aust. Nurs. J.* **1994**, *2*, 21–22.
2014. Tavel, M.E. Somatic Symptom Disorders Without Known Physical Causes: One Disease with Many Names? *Am. J. Med.* **2015**, *128*, 1054–1058.
2015. Taylor, A.K.; Loades, M.; Brigden, A.L.C.; Collin, S.M.; Crawley, E. 'It's personal to me': A qualitative study of depression in young people with CFS/ME. *Clin. Child Psychol. Psychiatry* **2017**, *22*, 326–340.
2016. Taylor, J.; Goodkin, H.P. Dizziness and Vertigo in the Adolescent. *Otolaryngol. Clin. NORTH Am.* **2011**, *44*, 309+.
2017. Taylor, R.R.; Jason, L.A. Sexual abuse, physical abuse, chronic fatigue, and chronic fatigue syndrome: a community-based study. *J. Nerv. Ment. Dis.* **2001**, *189*, 709–715.
2018. Taylor, R.R.; Jason, L.A.; Jahn, S.C. Chronic fatigue and sociodemographic characteristics as predictors of psychiatric disorders in a community-based sample. *Psychosom. Med.* **2003**, *65*, 896–901.
2019. Taylor, R.R.; Jason, L.A. Chronic fatigue, abuse-related traumatization, and psychiatric disorders in a community-based sample. *Soc. Sci. Med.* **2002**, *55*, 247–256.
2020. Taylor, R.R.; Jason, L.A.; Curie, C.J. Prognosis of chronic fatigue in a community-based sample. *Psychosom. Med.* **2002**, *64*, 319–327.
2021. Taylor, S.; Garralda, E. The management of somatoform disorder in childhood. *Curr. Opin. Psychiatry* **2003**, *16*, 227–231.
2022. Teitelbaum, J. Effective treatment of chronic fatigue syndrome. *Integr. Med.* **2005**, *4*, 24–29.
2023. Téllez-Zenteno, J.F.; Matijevic, S.; Wiebe, S. Somatic comorbidity of epilepsy in the general population in Canada. *Epilepsia* **2005**, *46*, 1955–1962.
2024. Ter Wolbeek, M.; Van Doornen, L.J.P.; Kavelaars, A.; Heijnen, C.J. Severe fatigue in adolescents: A common phenomenon? *Pediatrics* **2006**, *117*, e1078–e1086.

2025. ter Wolbeek, M.; Van Doornen, L.J.P.; Kavelaars, A.; Heijnen, C.J.; Wolbeek, M.; Van Doornen, L.J.P.; Kavelaars, A.; Heijnen, C.J. Predictors of persistent and new-onset fatigue in adolescent girls. *Pediatrics* **2008**, *121*, e449–e457.
2026. ter Wolbeek, M.; van Doornen, L.J.P.; Kavelaars, A.; Tersteeg-Kamperman, M.D.J.; Heijnen, C.J. Fatigue, depressive symptoms, and anxiety from adolescence up to young adulthood: a longitudinal study. *Brain. Behav. Immun.* **2011**, *25*, 1249–1255.
2027. Terlou, A.; Ruble, K.; Stapert, A.F.; Chang, H.-C.; Rowe, P.C.; Schwartz, C.L. Orthostatic intolerance in survivors of childhood cancer. *Eur. J. Cancer* **2007**, *43*, 2685–2690.
2028. Terrone, G.; Parente, I.; Romano, A.; Auricchio, R.; Greco, L.; Del Giudice, E. The Pediatric Symptom Checklist as screening tool for neurological and psychosocial problems in a paediatric cohort of patients with coeliac disease. *Acta Paediatr. Int. J. Paediatr.* **2013**, *102*, e325–e328.
2029. Terzi, R.; Altin, F. The prevalence of low back pain in hospital staff and its relationship with chronic fatigue syndrome and occupational factors [Hastane çalişanlarinda bel ağrisi sikliği, bel ağrisinin kronik yorgunluk sendromu ve mesleki faktörler ile ilişkisi]. *Agri* **2015**, *27*, 149–154. Work in Turkish
2030. Terzi, R.; Altin, F. [The prevalence of low back pain in hospital staff and its relationship with chronic fatigue syndrome and occupational factors]. *Agri* **2015**, *27*, 149–154. Work in Turkish
2031. Thase, M.E. Managing medical comorbidities in patients with depression to improve prognosis. *J. Clin. Psychiatry* **2016**, *77*, 22–27.
2032. Theorell, T.; Blomkvist, V.; Lindh, G.; Evengard, B. Critical life events, infections, and symptoms during the year preceding chronic fatigue syndrome (CFS): an examination of CFS patients and subjects with a nonspecific life crisis. *Psychosom. Med.* **1999**, *61*, 304–310.
2033. Thieben, M.J.; Sandroni, P.; Sletten, D.M.; Benrud-Larson, L.M.; Fealey, R.D.; Vernino, S.; Lennon, V.A.; Shen, W.-K.; Low, P.A. Postural orthostatic tachycardia syndrome: The Mayo Clinic experience. *MAYO Clin. Proc.* **2007**, *82*, 308–313.
2034. Thomas, A.M.; Tenholder, M.F. Pulmonary-related diseases of women: A perspective. *Clin. Pulm. Med.* **1999**, *6*, 73–79.
2035. Thomas, H. V; Stimpson, N.J.; Weightman, A.L.; Dunstan, F.; Lewis, G. Systematic review of multi-symptom conditions in Gulf War veterans. *Psychol. Med.* **2006**, *36*, 735–747.
2036. Thomas, M.; Christopher, G. Fatigue in developmental coordination disorder: an exploratory study in adults. *FATIGUE-BIOMEDICINE Heal. Behav.* **2018**, *6*, 41–51.
2037. Thomm, A.M.; Schotthoefer, A.M.; Dupuis II, A.P.; Kramer, L.D.; Frost, H.M.; Fritsche, T.R.; Harrington, Y.A.; Knox, K.K.; Kehl, S.C. Development and Validation of a Serologic Test Panel for Detection of Powassan Virus Infection in US Patients Residing in Regions Where Lyme Disease Is Endemic. *MSPHERE* **2018**, *3*.
2038. Thompson, D.P.; Antcliff, D.; Woby, S.R. Symptoms of chronic fatigue syndrome/myalgic encephalopathy are not determined by activity pacing when measured by the chronic pain coping inventory. *Physiotherapy* **2018**, *104*, 129–135.

2039. Thompson, D.; Hylan, T.R.; McMullen, W.; Romeis, M.E.; Buesching, D.; Oster, G. Predictors of a medical-offset effect among patients receiving antidepressant therapy. *Am. J. Psychiatry* **1998**, *155*, 824–827.
2040. Thompson, E.A.; Mathie, R.T.; Baitson, E.S.; Barron, S.J.; Berkovitz, S.R.; Brands, M.; Fisher, P.; Kirby, T.M.; Leckridge, R.W.; Mercer, S.W.; et al. Towards standard setting for patient-reported outcomes in the NHS homeopathic hospitals. *Homeopathy* **2008**, *97*, 114–121.
2041. Thorn, A. Methodologic aspects of the study of modern-age diseases: The example of sick-building syndrome. *Int. J. Occup. Environ. Health* **2002**, *8*, 363–370.
2042. Thörn, Å. Methodologic aspects of the study of modern-age diseases: The example of sick-building syndrome. *Int. J. Occup. Environ. Health* **2002**, *8*, 363–370.
2043. Thorpe, T.; McManimen, S.; Gleason, K.; Stoothoff, J.; Newton, J.L.; Strand, E.B.; Jason, L.A. Assessing current functioning as a measure of significant reduction in activity level. *FATIGUE-BIOMEDICINE Heal. Behav.* **2016**, *4*, 175–188.
2044. Tiersky, L.A.; Matheis, R.J.; Deluca, J.; Lange, G.; Natelson, B.H. Functional status, neuropsychological functioning, and mood in chronic fatigue syndrome (CFS): relationship to psychiatric disorder. *J. Nerv. Ment. Dis.* **2003**, *191*, 324–331.
2045. Tietjen, G.E.; Bushnell, C.D.; Herial, N.A.; Utley, C.; White, L.; Hafeez, F. Endometriosis is associated with prevalence of comorbid conditions in migraine: CME. *Headache* **2007**, *47*, 1069–1078.
2046. Tietjen, G.E.; Brandes, J.L.; Peterlin, B.L.; Eloff, A.; Dafer, R.M.; Stein, M.R.; Drexler, E.; Martin, V.T.; Hutchinson, S.; Aurora, S.K.; et al. Childhood maltreatment and migraine (part III). Association with comorbid pain conditions. *Headache* **2010**, *50*, 42–51.
2047. Tietjen, G.E.; Brandes, J.L.; Peterlin, B.L.; Eloff, A.; Dafer, R.M.; Stein, M.R.; Drexler, E.; Martin, V.T.; Hutchinson, S.; Aurora, S.K.; et al. Allodynia in migraine: association with comorbid pain conditions. *Headache* **2009**, *49*, 1333–1344.
2048. Tietjen, G.E.; Herial, N.A.; Hardgrove, J.; Utley, C.; White, L. Migraine comorbidity constellations. *Headache* **2007**, *47*, 857–865.
2049. Till, C.; Udler, E.; Ghassemi, R.; Narayanan, S.; Arnold, D.L.; Banwell, B.L. Factors associated with emotional and behavioral outcomes in adolescents with multiple sclerosis. *Mult. Scler. J.* **2012**, *18*, 1170–1180.
2050. Timmers, H.J.L.M.; Wieling, W.; Soetekouw, P.M.M.B.; Bleijenberg, G.; der Meer, J.W.M.; Lenders, J.W.M. Hemodynamic and neurohumoral responses to head-up tilt in patients with chronic fatigue syndrome. *Clin. Auton. Res.* **2002**, *12*, 273–280.
2051. Ting, J.Y.S.; Brown, A.F.T. Ciguatera poisoning: A global issue with common management problems. *Eur. J. Emerg. Med.* **2001**, *8*, 295–300.
2052. Tîrdei, G.; Ruţa, S.M.; Popescu, A.E. Human herpesvirus 6. General overview [Le virus herpétique humain du type 6. Présentation générale.]. *Rev. Roum. Virol.* **1994**, *45*, 83–95. Work in French
2053. Tirelli, U.; Lleshi, A.; Berretta, M.; Spina, M.; Talamini, R.; Giacalone, A. Treatment of 741 italian patients with chronic fatigue syndrome. *Eur. Rev. Med. Pharmacol. Sci.* **2013**, *17*, 2847–2852.
2054. Tjørve, E.; Tjørve, K.M.C.; Olsen, J.O.; Senum, R.; Oftebro, H. On commonness and rarity of thyroid hormone resistance: A discussion based on mechanisms of reduced sensitivity in peripheral tissues. *Med. Hypotheses* **2007**, *69*, 913–921.

2055. Tófoli, L.F.; Andrade, L.H.; Fortes, S. Somatization in Latin America: A review of the classification of somatoform disorders, functional syndromes and medically unexplained symptoms [Somatização na América Latina: Uma revisão sobre a classificação de transtornos somatoformes, syndromes funcionais e sintomas sem explicação médica]. *Rev. Bras. Psiquiatr.* **2011**, *33*, 559–569. Work in Portuguese
2056. Tófoli, L.F.; Andrade, L.H.; Fortes, S. Somatization in Latin America: a review of the classification of somatoform disorders, functional syndromes and medically unexplained symptoms. *Rev. Bras. Psiquiatr.* **2011**, *33 Suppl 1*, S59-80.
2057. Tofoli, L.F.; Andrade, L.H.; Fortes, S. Somatization in Latin America: a review on the classification of somatoform disorders, functional syndromes, and medically unexplained symptoms. *Rev. Bras. Psiquiatr.* **2011**, *33*, S59–S80.
2058. Togo, F.; Natelson, B.H. Heart rate variability during sleep and subsequent sleepiness in patients with chronic fatigue syndrome. *Auton. Neurosci.* **2013**, *176*, 85–90.
2059. Tollit, M.; Politis, J.; Knight, S. Measuring School Functioning in Students With Chronic Fatigue Syndrome: A Systematic Review. *J. Sch. Health* **2018**, *88*, 74–89.
2060. Tomljenovic, D.; Baudoin, T.; Megla, Z.B.; Geber, G.; Scadding, G.; Kalogjera, L. Females have stronger neurogenic response than males after non-specific nasal challenge in patients with seasonal allergic rhinitis. *Med. Hypotheses* **2018**, *116*, 114–118.
2061. Tomljenovic, L.; Shaw, C.A. Human papillomavirus (HPV) vaccine policy and evidence-based medicine: Are they at odds? *Ann. Med.* **2013**, *45*, 182–193.
2062. Tomljenovic, L.; Shaw, C.A. Do aluminum vaccine adjuvants contribute to the rising prevalence of autism? *J. Inorg. Biochem.* **2011**, *105*, 1489–1499.
2063. Tonkin, A. Low blood pressure and low energy: (How) are they related? *J. Hypertens.* **2004**, *22*, 671–673.
2064. Torpy, D.J.; Bachmann, A.W.; Grice, J.E.; Fitzgerald, S.P.; Phillips, P.J.; Whitworth, J.A.; Jackson, R. V Familial corticosteroid-binding globulin deficiency due to a novel null mutation: Association with fatigue and relative hypotension. *J. Clin. Endocrinol. Metab.* **2001**, *86*, 3692–3700.
2065. Torres-Harding, S.R.; Jason, L.A.; Cane, V.; Carrico, A.; Taylor, R.R. Physicians' diagnoses of psychiatric disorders for people with chronic fatigue syndrome. *Int. J. Psychiatry Med.* **2002**, *32*, 109–124.
2066. Torres-Harding, S.R.; Mason-Shutter, J.; Jason, L.A. Fatigue among Spanish- and English-speaking Latinos. *Soc. Work Public Health* **2008**, *23*, 55–72.
2067. Toulkidis, V.; Loblay, R.; Stewart, G.; Bertouch, J.; Cistulli, P.; Darveniza, P.; Ellis, C.; Gatenby, P.; Gillis, D.; Hickie, I.; et al. Chronic fatigue syndrome: Clinical practice guidelines - 2002. *Med. J. Aust.* **2002**, *176*, S17–S55.
2068. Tournier, J.N.; Drouet, E.; Jouan, A. [The Gulf war syndrome]. *Presse Med.* **2002**, *31*, 3–9. Work in French
2069. Trabal, J.; Leyes, P.; Fernandez-Sola, J.; Forga, M.; Fernandez-Huerta, J. Patterns of food avoidance in chronic fatigue syndrome: is there a case for dietary recommendations? *Nutr. Hosp.* **2012**, *27*, 659–662.
2070. Trevino, A.; Soriano, V.; VIH-HTLV, G.E.P.E. Infection by HIV-2, HTLV and new human retroviruses in Spain. *Med. Clin. (Barc).* **2012**, *138*, 541–544.
2071. Trigwell, P.; Hatcher, S.; Johnson, M.; Stanley, P.; House, A. "Abnormal" illness behaviour in chronic fatigue syndrome and multiple sclerosis. *BMJ* **1995**, *311*, 15–18.
2072. Trinidad, E.E.; Ramirez-Ronda, C. [Chronic fatigue syndrome]. *Bol. Asoc. Med. P. R.* **1994**, *86*, 56–61. Work in Spanish

2073. Trinidad, E.E.; Ramírez-Ronda, C. Chronic fatigue syndrome [Síndrome de fatiga crónica.]. *Bol. Asoc. Med. P. R.* **1994**, *86*, 56–61. Work in
2074. Tritt, K.; Nickel, M.; Mitterlehner, F.; Nickel, C.; Forthuber, P.; Leiberich, P.; Rother, W.; Loew, T. Chronic fatigue and indicators of long-term employment disability in psychosomatic inpatients. *Wien. Klin. Wochenschr.* **2004**, *116*, 182–189.
2075. Trivedi, M.H.; Katon, W.J.; Daly, E.; Clayton, A.H.; Frank, E. Recent advances in the treatment of depression in the presence of physical symptoms. *J. Clin. Psychiatry* **2006**, *67*, 310–321.
2076. Trivedi, M.S.; Oltra, E.; Sarria, L.; Rose, N.; Beljanski, V.; Fletcher, M.A.; Klimas, N.G.; Nathanson, L. Identification of Myalgic Encephalomyelitis/Chronic Fatigue Syndrome-associated DNA methylation patterns. *PLoS One* **2018**, *13*.
2077. Trojani, F.T. [The chronic fatigue syndrome]. *Clin. Ter.* 1994, *144*, 373–376. Work in Italian
2078. Trojani, F.T. [Chronic fatigue syndrome]. *Clin. Ter.* **1994**, *144*, 163–166. Work in Italian
2079. Tsai, S.-Y.; Yang, T.-Y.; Chen, H.-J.; Chen, C.-S.; Lin, W.-M.; Shen, W.-C.; Kuo, C.-N.; Kao, C.-H. Increased risk of chronic fatigue syndrome following herpes zoster: a population-based study. *Eur. J. Clin. Microbiol. Infect. Dis.* **2014**, *33*, 1653–1659.
2080. Tsai, S.-Y.; Chen, H.-J.; Chen, C.; Lio, C.-F.; Kuo, C.-F.; Leong, K.-H.; Wang, Y.-T.T.; Yang, T.-Y.; You, C.-H.; Wang, W.-S. Increased risk of chronic fatigue syndrome following psoriasis: A nationwide population-based cohort study. *J. Transl. Med.* **2019**, *17*.
2081. Tsai, S.-Y.; Chen, H.-J.; Lio, C.-F.; Kuo, C.-F.; Kao, A.-C.; Wang, W.-S.; Yao, W.-C.; Chen, C.; Yang, T.-Y. Increased risk of chronic fatigue syndrome in patients with inflammatory bowel disease: A population-based retrospective cohort study. *J. Transl. Med.* **2019**, *17*.
2082. Tsai, S.-Y.; Lin, C.-L.; Shih, S.-C.; Hsu, C.-W.; Leong, K.-H.; Kuo, C.-F.; Lio, C.-F.; Chen, Y.-T.; Hung, Y.-J.; Shi, L. Increased risk of chronic fatigue syndrome following burn injuries 11 Medical and Health Sciences 1117 Public Health and Health Services 11 Medical and Health Sciences 1103 Clinical Sciences. *J. Transl. Med.* **2018**, *16*.
2083. Tsai, S.-Y.; Lin, C.-L.; Shih, S.-C.; Hsu, C.-W.; Leong, K.-H.; Kuo, C.-F.; Lio, C.-F.; Chen, Y.-T.; Hung, Y.-J.; Shi, L. Increased risk of chronic fatigue syndrome following burn injuries. *J. Transl. Med.* **2018**, *16*, 342.
2084. Tschudi-Madsen, H.; Kjeldsberg, M.; Natvig, B.; Ihlebaek, C.; Straand, J.; Bruusgaard, D. Medically unexplained conditions considered by patients in general practice. *Fam. Pract.* **2014**, *31*, 156–163.
2085. Tseng, C.L.; Natelson, B.H. Few gender differences exist between women and men with chronic fatigue syndrome. *J. Clin. Psychol. Med. Settings* **2004**, *11*, 55–62.
2086. Tseng, Y.-J.; DeMaria Jr., A.; Goldmann, D.A.; Mandl, K.D. Claims-Based Diagnostic Patterns of Patients Evaluated for Lyme Disease and Given Extended Antibiotic Therapy. *VECTOR-BORNE ZOONOTIC Dis.* **2017**, *17*, 116–122.
2087. Tsujimura, K.; Mizutani, T.; Kariwa, H.; Yoshimatsu, K.; Ogino, M.; Morii, Y.; Inagaki, H.; Arikawa, J.; Takashima, I. A serosurvey of Borna disease virus infection in wild rats by a capture ELISA. *J. Vet. Med. Sci.* **1999**, *61*, 113–117.
2088. Tsukinoki, R.; Murakami, Y. Non-communicable disease epidemic: epidemiology in action (EuroEpi 2013 and NordicEpi 2013): Aarhus, Denmark from 11 August to 14 August 2013. *Eur. J. Epidemiol.* **2013**, *28*, 1–270.

2089. Tuke, P.W.; Tettmar, K.I.; Tamuri, A.; Stoye, J.P.; Tedder, R.S. PCR Master Mixes Harbour Murine DNA Sequences. Caveat Emptor! *PLoS One* **2011**, *6*.
2090. Tuncay, T.; Musabak, I.; Gok, D.E.; Kutlu, M. The relationship between anxiety, coping strategies and characteristics of patients with diabetes. *Health Qual. Life Outcomes* **2008**, *6*.
2091. Tung, A.; Hepp, Z.; Bansal, A.; Devine, E.B. Characterizing health care utilization, direct costs, and comorbidities associated with interstitial cystitis: A retrospective claims analysis. *J. Manag. Care Spec. Pharm.* **2017**, *23*, 474–482.
2092. TURGEON, S.A. CHRONIC FATIGUE SYNDROME, REVIEW OF THE LITERATURE. *Can. Fam. PHYSICIAN* **1991**, *37*, 1640–1646.
2093. Tveit, B. Snags in statistics and questionnaires. *Scand. J. Prim. Health Care* 1996, *14*, 64.
2094. Twisk, F.N.M. Underperformance of myalgic encephalomyelitis (ME)/chronic fatigue syndrome (CFS) patients at neurocognitive tests should be assessed objectively without an a priori judgment about the etiology. *J. Psychosom. Res.* **2014**, *76*, 339.
2095. Twisk, F.N.M. The status of and future research into Myalgic Encephalomyelitis and Chronic Fatigue Syndrome: The need of accurate diagnosis, objective assessment, and acknowledging biological and clinical subgroups. *Front. Physiol.* **2014**, *5 MAR*.
2096. Twisk, F.N.M.; Arnoldus, R.J.W.; Maes, M. Letter to the Editor: Plausible explanations for neurocognitive deficits in ME/CFS, aggravation of neurocognitive impairment induced by exertion. *Psychol. Med.* 2010, *40*, 1230–1231.
2097. Ulas, U.H.; Chelimsky, T.C.; Chelimsky, G.; Mandawat, A.; McNeeley, K.; Alshekhlee, A. Comorbid health conditions in women with syncope. *Clin. Auton. Res.* **2010**, *20*, 223–227.
2098. Ullrich, N.J. Introduction. *Semin. Pediatr. Neurol.* **2012**, *19*, 1–2.
2099. Ulus, Y.; Akyol, Y.; Tander, B.; Durmus, D.; Bilgici, A.; Kuru, O. Sleep quality in fibromyalgia and rheumatoid arthritis: associations with pain, fatigue, depression, and disease activity. *Clin. Exp. Rheumatol.* **2011**, *29*, S92-6.
2100. Underhill, R.A. Myalgic encephalomyelitis, chronic fatigue syndrome: An infectious disease. *Med. Hypotheses* **2015**, *85*, 765–773.
2101. Underhill, R.A.; O'Gorman, R. Prevalence of chronic fatigue syndrome and chronic fatigue within families of CFS patients. *J. Chronic Fatigue Syndr.* **2006**, *13*, 3–13.
2102. Underwood, M.; Eldridge, S.; Ridsdale, L.; Godfrey, E.; Seed, P. Chronic fatigue in general practice: (Multiple letters). *Br. J. Gen. Pract.* **2001**, *51*, 317–318.
2103. Unger, E.R.; Lin, J.-M.S.; Tian, H.; Gurbaxani, B.M.; Boneva, R.S.; Jones, J.F. Methods of applying the 1994 case definition of chronic fatigue syndrome - impact on classification and observed illness characteristics. *Popul. Health Metr.* **2016**, *14*.
2104. Unger, E.R.; Lin, J.-M.S.; Tian, H.; Natelson, B.H.; Lange, G.; Vu, D.; Blate, M.; Klimas, N.G.; Balbin, E.G.; Bateman, L.; et al. Multi-Site Clinical Assessment of Myalgic Encephalomyelitis/Chronic Fatigue Syndrome (MCAM): Design and Implementation of a Prospective/Retrospective Rolling Cohort Study. *Am. J. Epidemiol.* **2017**, *185*, 617–626.
2105. Unger, E.R.; Nisenbaum, R.; Moldofsky, H.; Cesta, A.; Sammut, C.; Reyes, M.; Reeves, W.C. Sleep assessment in a population-based study of chronic fatigue syndrome. *BMC Neurol.* **2004**, *4*, 6.

2106. Usón, J.R. V; Alecha, M.Á.I. Diagnostic and treatment challenges of chronic fatigue syndrome: Role of immediate-release methylphenidate. *Expert Rev. Neurother.* **2008**, *8*, 917–927.
2107. Uter, W.; Marcusson, J.A.; Lindh, G.; Evengard, B. Chronic fatigue syndrome and nickel allergy (multiple letters). *Contact Dermatitis* **2000**, *42*, 56–57.
2108. Valdez, A.R.; Hancock, E.E.; Adebayo, S.; Kiernicki, D.J.; Proskauer, D.; Attewell, J.R.; Bateman, L.; DeMaria A., J.; Lapp, C.W.; Rowe, P.C.; et al. Estimating prevalence, demographics, and costs of ME/CFS using large scale medical claims data and machine learning. *Front. Pediatr.* **2019**, *6*.
2109. Valenca, M.M.; Medeiros, F.L.; Martins, H.A.; Massaud, R.M.; Peres, M.F.P. Neuroendocrine dysfunction in fibromyalgia and migraine. *Curr. Pain Headache Rep.* **2009**, *13*, 358–364.
2110. Vallings, R. AACFS 7th International Conference. *J. Chronic Fatigue Syndr.* **2004**, *12*, 61–79.
2111. Valls-Llobet, C. Differential diagnosis between chronic pain and chronic fatigue: A pending matter [Dolor crónico y fatiga crónica: Una asignatura pendiente]. *Med. Clin. (Barc).* **2003**, *121*, 412–413. Work in Spanish
2112. van Campen, C.L.M.C.; Rowe, P.C.; Visser, F.C. Low sensitivity of abbreviated tilt table testing for diagnosing postural tachycardia syndrome in adults with ME/CFS. *Front. Pediatr.* **2018**, *6*.
2113. Van Campen, E.; Van Den Eede, F.; Moorkens, G.; Schotte, C.; Schacht, R.; Sabbe, B.G.C.; Cosyns, P.; Claes, S.J. Use of the Temperament and Character Inventory (TCI) for assessment of personality in chronic fatigue syndrome. *Psychosomatics* **2009**, *50*, 147–154.
2114. Van Cauwenbergh, D.; Nijs, J.; Kos, D.; Van Weijnen, L.; Struyf, F.; Meeus, M. Malfunctioning of the autonomic nervous system in patients with chronic fatigue syndrome: A systematic literature review. *Eur. J. Clin. Invest.* **2014**, *44*, 516–526.
2115. van de Putte, E.M.; Engelbert, R.H.H.; Kuis, W.; Kimpen, J.L.L.; Uiterwaal, C.S.P.M. How fatigue is related to other somatic symptoms. *Arch. Dis. Child.* **2006**, *91*, 824–827.
2116. van de Putte, E.M.; Engelbert, R.H.H.; Kuis, W.; Sinnema, G.; Kimpen, J.L.L.; Uiterwaal, C.S.P.M. Chronic fatigue syndrome and health control in adolescents and parents. *Arch. Dis. Child.* **2005**, *90*, 1020–1024.
2117. van de Putte, E.M.; Uiterwaal, C.S.P.M.; Bots, M.L.; Kuis, W.; Kimpen, J.L.L.; Engelbert, R.H.H. Is chronic fatigue syndrome a connective tissue disorder? A cross-sectional study in adolescents. *Pediatrics* **2005**, *115*, e415-22.
2118. van de Putte, E.M.; Engelbert, R.H.H.; Kuis, W.; Kimpen, J.L.L.; Uiterwaal, C.S.P.M.; de Putte, E.M. van; Engelbert, R.H.H.; Kuis, W.; Kimpen, J.L.L.; Uiterwaal, C.S.P.M. Alexithymia in adolescents with chronic fatigue syndrome. *J. Psychosom. Res.* **2007**, *63*, 377–380.
2119. van de Putte, E.M.; van Doornen, L.J.P.; Engelbert, R.H.H.; Kuis, W.; Kimpen, J.L.L.; Uiterwaal, C.S.P.M. Mirrored symptoms in mother and child with chronic fatigue syndrome. *Pediatrics* **2006**, *117*, 2074–2079.
2120. Van den Bergh, M.; Bauer, F.A.; Posteraro, A.F.; Thumma, S.; Dasanu, C.A. An unusual presentation of Kikuchi-Fujimoto disease. *Conn. Med.* **2014**, *78*, 225–228.
2121. Van Den Eede, F.; Haccuria, T.; De Venter, M.; Moorkens, G. Childhood sexual abuse and chronic fatigue syndrome. *Br. J. Psychiatry* 2012, *200*, 164–165.
2122. van der Hiele, K.; Spliethoff-Kamminga, N.G.; Ruimschotel, R.P.; Middelkoop, H.A.; Visser, L.H. Daily hassles reported by Dutch multiple sclerosis patients. *J. Neurol. Sci.* **2012**, *320*, 85–90.

2123. van der Linden, G.; Chalder, T.; Hickie, I.; Koschera, A.; Sham, P.; Wessely, S. Fatigue and psychiatric disorder: different or the same? *Psychol. Med.* **1999**, *29*, 863–868.
2124. van der Meer, J.W.M.; Netea, M.G.; Galama, J.M.D.; van Kuppeveld, F.J.M. Comment on “Detection of an infectious retrovirus, XMRV, in blood cells of patients with chronic fatigue syndrome”. *Science* **2010**, *328*, 825; author reply 825.
2125. van der Windt, D.A.W.M.; Dunn, K.M.; Spies-Dorgelo, M.N.; Mallen, C.D.; Blankenstein, A.H.; Stalman, W.A.B. Impact of physical symptoms on perceived health in the community. *J. Psychosom. Res.* **2008**, *64*, 265–274.
2126. van Geelen, S.M.; Bakker, R.J.; Kuis, W.; van de Putte, E.M. Adolescent chronic fatigue syndrome: a follow-up study. *Arch. Pediatr. Adolesc. Med.* **2010**, *164*, 810–814.
2127. van Geelen, S.M.; Fuchs, C.E.; van Geel, R.; Luyten, P.; van de Putte, E.M. The Self beyond Somatic Symptoms: A Narrative Approach to Self-Experience in Adolescent Chronic Fatigue Syndrome. *Psychopathology* **2015**, *48*, 278–286.
2128. van Geelen, S.M.; Sinnema, G.; Hermans, H.J.M.; Kuis, W. Personality and chronic fatigue syndrome: methodological and conceptual issues. *Clin. Psychol. Rev.* **2007**, *27*, 885–903.
2129. van Gils, A.; Burton, C.; Bos, E.H.; Janssens, K.A.M.; Schoevers, R.A.; Rosmalen, J.G.M. Individual variation in temporal relationships between stress and functional somatic symptoms. *J. Psychosom. Res.* **2014**, *77*, 34–39.
2130. van Heukelom, R.O.; Prins, J.B.; Smits, M.G.; Bleijenberg, G. Influence of melatonin on fatigue severity in patients with chronic fatigue syndrome and late melatonin secretion. *Eur. J. Neurol.* **2006**, *13*, 55–60.
2131. Van Hoof, E.; De Meirleir, K.; McGregor, N. Journal of Chronic Fatigue Syndrome: Editorial. *J. Chronic Fatigue Syndr.* **2006**, *13*, 1–2.
2132. Van Hoof, R. Gulf syndrome...Balkans syndrome...chronic fatigue syndrome [Syndrome du Golfe ... syndrome des Balkans ... syndrome de fatigue chronique.]. *Bull. Mem. Acad. R. Med. Belg.* **2000**, *155*, 281–294. Work in French
2133. Van Hoof, R. [Gulf syndrome... Balkans syndrome...chronic fatigue syndrome]. *Bull. Mem. Acad. R. Med. Belg.* 2000, *155*, 281–284. Work in French
2134. van Hoogmoed, D.; Fransen, J.; Bleijenberg, G.; van Riel, P. Physical and psychosocial correlates of severe fatigue in rheumatoid arthritis. *Rheumatology* **2010**, *49*, 1294–1302.
2135. Van Houdenhove, B. Letter to the editor. *Semin. Arthritis Rheum.* **2003**, *33*, 134–135.
2136. Van Houdenhove, B. Listening to CFS: Why we should pay more attention to the story of the patient. *J. Psychosom. Res.* **2002**, *52*, 495–499.
2137. Van Houdenhove, B. Psychiatric comorbidity and chronic fatigue syndrome. *Br. J. Psychiatry* 2006, *188*, 395; author reply 396.
2138. Van Houdenhove, B.; Luyten, P. Fibromyalgia and related syndromes characterised by stress intolerance and pain hypersensitivity: Do we need a new nosology? *Curr. Rheumatol. Rev.* **2007**, *3*, 304–308.
2139. Van Houdenhove, B.; Neerinckx, E.R. Victimization in fibromyalgia and chronic fatigue syndrome in tertiary care: A controlled study on prevalence and characteristics. *Psychosom. Med.* **2000**, *62*, 148.
2140. Van Houdenhove, B.; Neerinckx, E.; Lysens, R.; Vertommen, H.; Van Houdenhove, L.; Onghena, P.; Westhovens, R.; D’Hooghe, M.B. Victimization in chronic fatigue syndrome and fibromyalgia in tertiary care: a controlled study on prevalence and characteristics. *Psychosomatics* **2001**, *42*, 21–28.

2141. Van Houdenhove, B. What is the aim of cognitive behaviour therapy in patients with chronic fatigue syndrome? *Psychother. Psychosom.* 2006, *75*, 396–397.
2142. Van Houdenhove, B. Chronic fatigue syndrome, fibromyalgia, and complex regional pain syndrome type I. *Psychosomatics* 2003, *44*, 173–174.
2143. Van Houdenhove, B.; Kempke, S.; Luyten, P. Psychiatric aspects of chronic fatigue syndrome and fibromyalgia. *Curr. Psychiatry Rep.* **2010**, *12*, 208–214.
2144. Van Houdenhove, B.; Neerinckx, E.; Onghena, P.; Vingerhoets, A.; Lysens, R.; Vertommen, H. Daily hassles reported by chronic fatigue syndrome and fibromyalgia patients in tertiary care: a controlled quantitative and qualitative study. *Psychother. Psychosom.* **2002**, *71*, 207–213.
2145. Van Ittersum, M.W.; van Wilgen, C.P.; Hilberdink, W.K.H.A.; Groothoff, J.W.; van der Schans, C.P. Illness perceptions in patients with fibromyalgia. *PATIENT Educ. Couns.* **2009**, *74*, 53–60.
2146. Van Kuppeveld, F.J.M.; De Jong, A.S.; Lanke, K.H.; Verhaegh, G.W.; Melchers, W.J.G.; Swanink, C.M.A.; Bleijenberg, G.; Netea, M.G.; Galama, J.M.D.; Van Der Meer, J.W.M. Prevalence of xenotropic murine leukaemia virus-related virus in patients with chronic fatigue syndrome in the Netherlands: Retrospective analysis of samples from an established cohort. *BMJ* **2010**, *340*, 520.
2147. Van Oudenhove, L.; Vandenberghe, J.; Vos, R.; Holvoet, L.; Tack, J. Factors associated with co-morbid irritable bowel syndrome and chronic fatigue-like symptoms in functional dyspepsia. *Neurogastroenterol. Motil.* **2011**, *23*, 524-e202.
2148. van Tilburg, M.A.L.; Zaki, E.A.; Venkatesan, T.; Boles, R.G. Irritable Bowel Syndrome May Be Associated with Maternal Inheritance and Mitochondrial DNA Control Region Sequence Variants. *Dig. Dis. Sci.* **2014**, *59*, 1392–1397.
2149. van't Leven, M.; Zielhuis, G.A.; van der Meer, J.W.; Verbeek, A.L.; Bleijenberg, G. Fatigue and chronic fatigue syndrome-like complaints in the general population. *Eur. J. Public Health* **2010**, *20*, 251–257.
2150. Vandenbergen, J.; Vanheule, S.; Rosseel, Y.; Desmet, M.; Verhaeghe, P. Unexplained chronic fatigue and core conflictual relationship themes: A study in a chronically fatigued population. *Psychol. Psychother.* **2009**, *82*, 31–40.
2151. Vanheule, S.; Vandenbergen, J.; Desmet, M.; Rosseel, Y.; Insleghers, R. Alexithymia and core conflictual relationship themes: a study in a chronically fatigued primary care population. *Int. J. Psychiatry Med.* **2007**, *37*, 87–98.
2152. Vardy, J.; Tannock, I. Cognitive function after chemotherapy in adults with solid tumours. *Crit. Rev. Oncol. Hematol.* **2007**, *63*, 183–202.
2153. Vavakova, M.; Durackova, Z.; Trebaticka, J. Markers of Oxidative Stress and Neuroprogression in Depression Disorder. *Oxid. Med. Cell. Longev.* **2015**.
2154. Vecchiet, L. Muscle pain and aging. *J. Musculoskelet. Pain* **2002**, *10*, 5–22.
2155. Vella-Baldacchino, M.D.; Schembri, M.; Vella-Baldacchino, M. Myalgic encephalomyelitis/chronic fatigue syndrome (ME/CFS). *Malta Med. J.* **2014**, *26*, 17–22.
2156. Venables, P.J. Management of patients presenting with Sjogren's syndrome. *BEST Pract. Res. Clin. Rheumatol.* **2006**, *20*, 791–807.
2157. Vercoulen, J.H.; Hommes, O.R.; Swanink, C.M.; Jongen, P.J.; Fennis, J.F.; Galama, J.M.; van der Meer, J.W.; Bleijenberg, G. The measurement of fatigue in patients with multiple sclerosis. A multidimensional comparison with patients with chronic fatigue syndrome and healthy subjects. *Arch. Neurol.* **1996**, *53*, 642–649.
2158. Vercoulen, J.H.M.M.; Bazelmans, E.; Swanink, C.M.A.; Galama, J.M.D.; Fennis, J.F.M.; Van Der Meer, J.W.M.; Bleijenberg, G. Evaluating neuropsychological

impairment in chronic fatigue syndrome. *J. Clin. Exp. Neuropsychol.* **1998**, *20*, 144–156.
2159. Verhulst, S.L.; Schrauwen, N.N.; De Backer, W.; Boudewyns, A.; Desager, K. Screening for cannabis use in a pediatric sleep clinic. *J. Paediatr. Child Health* **2008**, *44*, 529–530.
2160. Vermeulen, R.C.W.; Scholte, H.R. Rupture of silicone gel breast implants and symptoms of pain and fatigue. *J. Rheumatol.* **2003**, *30*, 2263–2267.
2161. Vermeulen, R.C.W.; Scholte, H.R. Chronic fatigue syndrome and sexual dysfunction. *J. Psychosom. Res.* **2004**, *56*, 199–201.
2162. Verner, O.M.; Murashko, N.K. [Epidemiology, diagnostics, and treatment of complications after neuroinfections: chronic fatigue syndrome]. *Likars'ka Sprav.* **2012**, 78–81. Work in Ukrainian
2163. Vernon, S.D.; Whistler, T.; Aslakson, E.; Rajeevan, M.; Reeves, W.C. Challenges for molecular profiling of chronic fatigue syndrome. *Pharmacogenomics* **2006**, *7*, 211–218.
2164. Vernon, S.D.; Reeves, W.C. The challenge of integrating disparate high-content data: epidemiological, clinical and laboratory data collected during an in-hospital study of chronic fatigue syndrome. *Pharmacogenomics* **2006**, *7*, 345–354.
2165. Versluis, R.G.; de Waal, M.W.; Opmeer, C.; Petri, H.; Springer, M.P. [Prevalence of chronic fatigue syndrome in 4 family practices in Leiden]. *Ned. Tijdschr. Geneeskd.* **1997**, *141*, 1523–1526.
2166. Versluis, R.G.J.A.; De Waal, M.W.M.; Opmeer, C.; Petri, H.; Springer, M.P. Prevalence of chronic fatigue syndrome in four general practices in Leyden region [Prevalentie van chronische-vermoeidheidsyndroom in 4 huisartspraktijken in de regio Leiden]. *Ned. Tijdschr. Geneeskd.* **1997**, *141*, 1523–1526. Work in Dutch
2167. Viaene, M.; Vermeir, G.; Godderis, L. Sleep disturbances and occupational exposure to solvents. *Sleep Med. Rev.* **2009**, *13*, 235–243.
2168. Vij, B.; Whipple, M.O.; Tepper, S.J.; Mohabbat, A.B.; Stillman, M.; Vincent, A. Frequency of migraine headaches in patients with fibromyalgia. *Headache* **2015**, *55*, 860–865.
2169. Vincent, A.; Brimmer, D.J.; Whipple, M.O.; Jones, J.F.; Boneva, R.; Lahr, B.D.; Maloney, E.; St Sauver, J.L.; Reeves, W.C. Prevalence, incidence, and classification of chronic fatigue syndrome in Olmsted County, Minnesota, as estimated using the Rochester Epidemiology Project. *Mayo Clin. Proc.* **2012**, *87*, 1145–1152.
2170. Viner, R.M.; Clark, C.; Taylor, S.J.C.; Bhui, K.; Klineberg, E.; Head, J.; Booy, R.; Stansfeld, S.A. Longitudinal risk factors for persistent fatigue in adolescents. *Arch. Pediatr. Adolesc. Med.* **2008**, *162*, 469–475.
2171. Viner, R.; Hotopf, M. Childhood predictors of self reported chronic fatigue syndrome/myalgic encephalomyelitis in adults: national birth cohort study. *BMJ* **2004**, *329*, 941.
2172. Vingerhoets, A.; Van Huijgevoort, M.; Van Heck, G.L. Leisure sickness: A pilot study on its prevalence, phenomenology, and background. *Psychother. Psychosom.* **2002**, *71*, 311–317.
2173. Vistad, I.; Fosså, S.D.; Kristensen, G.B.; Dahl, A.A. Chronic fatigue and its correlates in long-term survivors of cervical cancer treated with radiotherapy. *BJOG An Int. J. Obstet. Gynaecol.* **2007**, *114*, 1150–1158.
2174. Vlieger, A.M.; Van De Putte, E.M.; Hoeksma, H. The use of complementary and alternative medicine in children at a general paediatric clinic and parental reasons for use [Het gebruik van complementaire en alternatieve geneeswijzen door

kinderen op een polikliniek voor kindergeneeskunde en de redenen van ouders daarvoor]. *Ned. Tijdschr. Geneeskd.* **2006**, *150*, 625–630. Work in Dutch
2175. Voermans, N.C.; Knoop, H.; Bleijenberg, G.; van Engelen, B.G. Pain in Ehlers-Danlos Syndrome Is Common, Severe, and Associated with Functional Impairment. *J. Pain Symptom Manage.* **2010**, *40*, 370–378.
2176. Vojdani, A.; Choppa, P.C.; Tagle, C.; Andrin, R.; Samimi, B.; Lapp, C.W. Detection of Mycoplasma genus and Mycoplasma fermentans by PCR in patients with chronic fatigue syndrome. *FEMS Immunol. Med. Microbiol.* **1998**, *22*, 355–365.
2177. Vojdani, A.; Lambert, J. *Crossing barriers: Gut-to-brain lessons from interdisciplinary collaboration*; 2016;
2178. Vollmer-Conna, U. Chronic fatigue syndrome in adolescence: Where to from here? *Arch. Pediatr. Adolesc. Med.* **2010**, *164*, 880–881.
2179. Von Baehr, R.; Von Baehr, V. Nickel sensitization as a possible cofactor in chronic fatigue syndrome [Nickelsensibilisierung als möglicher kofaktor bei chronischer müdigkeit]. *Zeitschrift fur Umweltmedizin* **2003**, *11*, 87–91. Work in German
2180. Von Heuft, G.; Schneider, G.; Klaiberg, A.; Brahler, E. [Bombed out--psychic and psychosomatic long term consequences of World War II for the cohort born until 1945 in the year 2004]. *Z. Psychosom. Med. Psychother.* **2007**, *53*, 228–243. Work in German
2181. Vordermark, D.; Schwab, M.; Flentje, M.; Sailer, M.; Kölbl, O. Chronic fatigue after radiotherapy for carcinoma of the prostate: Correlation with anorectal and genitourinary function. *Radiother. Oncol.* **2002**, *62*, 293–297.
2182. Vorsters, A.; Arbyn, M.; Baay, M.; Bosch, X.; de Sanjosé, S.; Hanley, S.; Karafillakis, E.; Lopalco, P.L.; Pollock, K.G.; Yarwood, J.; et al. Overcoming barriers in HPV vaccination and screening programs. *Papillomavirus Res.* **2017**, *4*, 45–53.
2183. Wada, K.; Sakata, Y.; Theriault, G.; Aratake, Y.; Shimizu, M.; Tsutsumi, A.; Tanaka, K.; Aizawa, Y. Effort-reward imbalance and social support are associated with chronic fatigue among medical residents in Japan. *Int. Arch. Occup. Environ. Health* **2008**, *81*, 331–336.
2184. Wagner, L.I.; Jason, L.A. Outcomes of occupational stressors on nurses: Chronic fatigue syndrome-related symptoms. *Nurs. Connect.* **1997**, *10*, 41–49.
2185. Wainberg, M.A.; Jeang, K.-T. XMRV as a human pathogen? *Cell Host Microbe* **2011**, *9*, 260–262.
2186. Wakefield, D.; Lloyd, A.; Brockman, A. Immunoglobulin subclass abnormalities in patients with chronic fatigue syndrome. *Pediatr. Infect. Dis. J.* **1990**, *9*, S50-3.
2187. Walford, G.A.; Nelson, W.M.; McCluskey, D.R. Fatigue, depression, and social adjustment in chronic fatigue syndrome. *Arch. Dis. Child.* **1993**, *68*, 384–388.
2188. Walitt, B.; Ceko, M.; Gracely, J.L.; Gracely, R.H. Neuroimaging of Central Sensitivity Syndromes: Key Insights from the Scientific Literature. *Curr. Rheumatol. Rev.* **2016**, *12*, 55–87.
2189. Walker, E.A.; Katon, W.J.; Jemelka, R.P. Psychiatric disorders and medical care utilization among people in the general population who report fatigue. *J. Gen. Intern. Med.* **1993**, *8*, 436–440.
2190. Walker, T.L. Chronic fatigue syndrome. Do you know what it means? *Am. J. Nurs.* **1999**, *99*, 70–74,76.
2191. Wallace, D.J. The fibromyalgia syndrome. *Ann. Med.* **1997**, *29*, 9–21.
2192. Wallace, H.L.; Natelson, B.; Gause, W.; Hay, J. Human herpesviruses in chronic fatigue syndrome. *Clin. Diagn. Lab. Immunol.* **1999**, *6*, 216–223.

2193. Wallace, P.G. Epidemiology: A critical review. *Br. Med. Bull.* **1991**, *47*, 942–951.
2194. Wallace, P.G. Post-viral fatigue syndrome. Epidemiology: a critical review. *Br. Med. Bull.* **1991**, *47*, 942–951.
2195. Wallis, A.; Ball, M.; Butt, H.; Lewis, D.P.; McKechnie, S.; Paull, P.; Jaa-Kwee, A.; Bruck, D. Open-label pilot for treatment targeting gut dysbiosis in myalgic encephalomyelitis/chronic fatigue syndrome: neuropsychological symptoms and sex comparisons. *J. Transl. Med.* **2018**, *16*.
2196. Wallman, D.; Weinberg, J.; Hohler, A.D. Ehlers-Danlos Syndrome and Postural Tachycardia Syndrome: A relationship study. *J. Neurol. Sci.* **2014**, *340*, 99–102.
2197. Walsh, C.M.; Zainal, N.Z.; Middleton, S.J.; Paykel, E.S. A family history study of chronic fatigue syndrome. *Psychiatr. Genet.* **2001**, *11*, 123–128.
2198. Walters, S.J. Therapist effects in randomised controlled trials: What to do about them. *J. Clin. Nurs.* **2010**, *19*, 1102–1112.
2199. Waltman, P.; Pearlman, A.; Mishra, B. Interpreter of maladies: redescription mining applied to biomedical data analysis. *Pharmacogenomics* **2006**, *7*, 503–509.
2200. Wang, W.; Russell, A.; Yan, Y. Traditional Chinese medicine and new concepts of predictive, preventive and personalized medicine in diagnosis and treatment of suboptimal health. *EPMA J.* **2014**, *5*.
2201. Wang, Y.; Liu, X.; Qiu, J.; Wang, H.; Liu, D.; Zhao, Z.; Song, M.; Song, Q.; Wang, X.; Zhou, Y.; et al. Association between Ideal Cardiovascular Health Metrics and Suboptimal Health Status in Chinese Population. *Sci. Rep.* **2017**, *7*.
2202. Wang, Z.; Hoy, W.; McDonald, S. Body mass index in aboriginal Australians in remote communities. *Aust. N. Z. J. Public Health* **2000**, *24*, 570–575.
2203. Wanman, A. Temporomandibular disorders among smokers and nonsmokers: A longitudinal cohort study. *J. Orofac. Pain* **2005**, *19*, 209–217.
2204. Ward, D.; Thorsen, N.M.; Frisch, M.; Valentiner-Branth, P.; Mølbak, K.; Hviid, A. A cluster analysis of serious adverse event reports after human papillomavirus (HPV) vaccination in Danish girls and young women, September 2009 to August 2017. *Euro Surveill.* **2019**, *24*.
2205. Ward, M.H.; DeLisle, H.; Shores, J.H.; Slocum, P.C.; Foresman, B.H. Chronic fatigue complaints in primary care: Incidence and diagnostic patterns. *J. Am. Osteopath. Assoc.* **1996**, *96*, 34–47.
2206. Ware, M.A.; Adams, H.; Guy, G.W. The medicinal use of cannabis in the UK: Results of a nationwide survey. *Int. J. Clin. Pract.* **2005**, *59*, 291–295.
2207. WARE, N.C. SUFFERING AND THE SOCIAL CONSTRUCTION OF ILLNESS - THE DELEGITIMATION OF ILLNESS EXPERIENCE IN CHRONIC FATIGUE SYNDROME. *Med. Anthropol. Q.* **1992**, *6*, 347–361.
2208. Ware, N.C. Toward a model of social course in chronic illness: The example of chronic fatigue syndrome. *Cult. Med. PSYCHIATRY* **1999**, *23*, 303–331.
2209. Ware, N.C. Sociosomatics and illness course in chronic fatigue syndrome. *Psychosom. Med.* **1998**, *60*, 394–401.
2210. Warren, J.W.; Howard, F.M.; Cross, R.K.; Good, J.L.; Weissman, M.M.; Wesselmann, U.; Langenberg, P.; Greenberg, P.; Clauw, D.J. Antecedent Nonbladder Syndromes in Case-Control Study of Interstitial Cystitis/Painful Bladder Syndrome. *Urology* **2009**, *73*, 52–57.
2211. Warren, J.W.; Langenberg, P.; Clauw, D.J. The number of existing functional somatic syndromes (FSSs) is an important risk factor for new, different FSSs. *J. Psychosom. Res.* **2013**, *74*, 12–17.

2212. Warren, J.W.; Wesselmann, U.; Morozov, V.; Langenberg, P.W. Numbers and types of nonbladder syndromes as risk factors for interstitial cystitis/painful bladder syndrome. *J. Urol.* **2011**, *77*, 313–319.
2213. Warren, J.W.; Clauw, D.J. Functional somatic syndromes: sensitivities and specificities of self-reports of physician diagnosis. *Psychosom. Med.* **2012**, *74*, 891–895.
2214. Watad, A.; Quaresma, M.; Bragazzi, N.L.; Cervera, R.; Tervaert, J.W.C.; Amital, H.; Shoenfeld, Y. The autoimmune/inflammatory syndrome induced by adjuvants (ASIA)/Shoenfeld's syndrome: descriptive analysis of 300 patients from the international ASIA syndrome registry. *Clin. Rheumatol.* **2018**, *37*, 483–493.
2215. Watad, A.; Rosenberg, V.; Tiosano, S.; Tervaert, J.W.C.; Yavne, Y.; Shoenfeld, Y.; Shalev, V.; Chodick, G.; Amital, H. Silicone breast implants and the risk of autoimmune/rheumatic disorders: A real-world analysis. *Int. J. Epidemiol.* **2018**, *47*, 1846–1854.
2216. Watanabe, A. Various clinical types of Q-fever disease. *Intern. Med.* 2004, *43*, 1–2.
2217. Watanabe, N.; Stewart, R.; Jenkins, R.; Bhugra, D.K.; Furukawa, T.A. The epidemiology of chronic fatigue, physical illness, and symptoms of common mental disorders: a cross-sectional survey from the second British National Survey of Psychiatric Morbidity. *J. Psychosom. Res.* **2008**, *64*, 357–362.
2218. Watson, N.F.; Buchwald, D.; Goldberg, J.; Maravilla, K.R.; Noonan, C.; Guan, Q.; Ellenbogen, R.G. Is Chiari I Malformation Associated With Fibromyalgia? *Neurosurgery* **2011**, *68*, 443–448.
2219. Watson, N.F.; Jacobsen, C.; Goldberg, J.; Kapur, V.; Buchwald, D. Subjective and objective sleepiness in monozygotic twins discordant for chronic fatigue syndrome. *Sleep* **2004**, *27*, 973–977.
2220. Watson, N.F.; Kapur, V.; Arguelles, L.M.; Goldberg, J.; Schmidt, D.F.; Armitage, R.; Buchwald, D. Comparison of subjective and objective measures of insomnia in monozygotic twins discordant for chronic fatigue syndrome. *Sleep* **2003**, *26*, 324–328.
2221. Waugh, E.M.; Jarrett, R.F.; Shield, L.; Montgomery, D.; Dean, R.T.G.; Mitchell, A.; Greaves, M.F.; Gallagher, A. The retrovirus XMRV is not directly involved in the pathogenesis of common types of lymphoid malignancy. *Cancer Epidemiol. Biomarkers Prev.* **2011**, *20*, 2232–2236.
2222. Wawrzyniak, N.R.; Joseph, A.-M.; Levin, D.G.; Gundermann, D.M.; Leeuwenburgh, C.; Sandesara, B.; Manini, T.M.; Adhihetty, P.J. Idiopathic chronic fatigue in older adults is linked to impaired mitochondrial content and biogenesis signaling in skeletal muscle. *Oncotarget* **2016**, *7*, 52695–52709.
2223. Wearden, A.; Appleby, L. Cognitive performance and complaints of cognitive impairment in chronic fatigue syndrome (CFS). *Psychol. Med.* **1997**, *27*, 81–90.
2224. Weaver, S.A.; Janal, M.N.; Aktan, N.; Ottenweller, J.E.; Natelson, B.H. Sex differences in plasma prolactin response to tryptophan in chronic fatigue syndrome patients with and without comorbid fibromyalgia. *J. Women's Heal.* **2010**, *19*, 951–958.
2225. Wei, P.M.; Pu, Y.P.; Zhao, J.W. Prevalence survey on condom use and infection of urogenital mycoplasmas in female sex workers in China. *Contraception* **2005**, *72*, 217–220.
2226. Weidenhammer, W.; Wessel, A.; Hutter, A.; Melchart, D.; Schroder, A. [Chronic fatigue in complementary rehabilitative medicine--predictors of the outcomes]. *Rehabilitation (Stuttg).* **2006**, *45*, 299–308. Work in German

2227. Weinbacher, B.S. [Persistent fatigue despite sufficient sleep]. *Praxis (Bern. 1994).* **2003**, *92*, 356–359. Work in German
2228. Weinstock, L.B.; Rezaie, A.; Afrin, L.B. The Significance of Mast Cell Activation in the Era of Precision Medicine. *Am. J. Gastroenterol.* **2018**, *113*, 1725–1726.
2229. Weir, P.T.; Harlan, G.A.; Nkoy, F.L.; Jones, S.S.; Hegmann, K.T.; Gren, L.H.; Lyon, J.L. The incidence of fibromyalgia and its associated comorbidities: A population-based retrospective cohort study based on international classification of diseases, 9th revision codes. *J. Clin. Rheumatol.* **2006**, *12*, 124–128.
2230. Wenz, H.; Wenz, R.; Groden, C.; Schmieder, K.; Fontana, J. The pre-interventional psychiatric history - An underestimated confounder in benign intracranial lesions studies. *Clin. Neurol. Neurosurg.* **2015**, *137*, 116–120.
2231. Werker, C.L.; Nijhof, S.L.; van de Putte, E.M. Clinical Practice: Chronic fatigue syndrome. *Eur. J. Pediatr.* **2013**, *172*, 1293–1298.
2232. Wernham, W.; Pheby, D.; Saffron, L. Risk factors for the development of severe ME/CFS - A pilot study. *J. Chronic Fatigue Syndr.* **2004**, *12*, 47–50.
2233. Wessely, S. Chronic fatigue syndrome and children. *Psychiatr. Bull.* **1998**, *22*, 193–194.
2234. Wessely, S. Neurasthenia and Chronic Fatigue: Theory and Practice in Britain and America. *Transcult. Psychiatry* **1994**, *31*, 173–209.
2235. Wessely, S. Chronic fatigue syndrome: a 20th century illness? *Scand. J. Work. Environ. Health* **1997**, *23 Suppl 3*, 17–34.
2236. Wessely, S. Chronic fatigue syndrome. Summary of a report of a joint committee of the Royal Colleges of Physicians, Psychiatrists and General Practitioners. *J. R. Coll. Physicians Lond.* **1996**, *30*, 497–504.
2237. Wessely, S. The epidemiology of chronic fatigue syndrome. *Epidemiol. Rev.* **1995**, *17*, 139–151.
2238. Wessely, S.; Chalder, T.; Hirsch, S.; Pawlikowska, T.; Wallace, P.; Wright, D.J. Postinfectious fatigue: prospective cohort study in primary care. *Lancet (London, England)* **1995**, *345*, 1333–1338.
2239. Wessely, S.; Chalder, T.; Hirsch, S.; Wallace, P.; Wright, D. The prevalence and morbidity of chronic fatigue and chronic fatigue syndrome: a prospective primary care study. *Am. J. Public Health* **1997**, *87*, 1449–1455.
2240. Wessely, S.; Chalder, T.; Hirsch, S.; Wallace, P.; Wright, D. Psychological symptoms, somatic symptoms, and psychiatric disorder in chronic fatigue and chronic fatigue syndrome: a prospective study in the primary care setting. *Am. J. Psychiatry* **1996**, *153*, 1050–1059.
2241. Wessely, S.; Hotopf, M. Is fibromyalgia a distinct clinical entity? Historical and epidemiological evidence. *Baillieres. Best Pract. Res. Clin. Rheumatol.* **1999**, *13*, 427–436.
2242. Wessely, S.; Sharpe, M. Letter to the Editor. *FATIGUE-BIOMEDICINE Heal. Behav.* **2018**, *6*, 61–62.
2243. White, K.P.; Speechley, M.; Harth, M.; Ostbye, T. Co-existence of chronic fatigue syndrome with fibromyalgia syndrome in the general population. A controlled study. *Scand. J. Rheumatol.* **2000**, *29*, 44–51.
2244. White, L.A.; Birnbaum, H.G.; Kaltenboeck, A.; Tang, J.; Mallett, D.; Robinson, R.L. Employees with fibromyalgia: Medical comorbidity, healthcare costs, and work loss. *J. Occup. Environ. Med.* **2008**, *50*, 13–24.
2245. White, M.T.; Lemkau, J.P.; Clasen, M.E. Fibromyalgia: A feminist biopsychosocial perspective. *WOMEN Ther.* **2001**, *23*, 45–58.

2246. White, P.D. How common is chronic fatigue syndrome; how long is a piece of string? *Popul. Health Metr.* **2007**, *5*.
2247. White, P.D. *Depression and chronic fatigue*; 2006;
2248. White, P.D. The relationship between infection and fatigue. *J. Psychosom. Res.* 1997, *43*, 345–350.
2249. White, P.D.; Thomas, J.M.; Amess, J.; Crawford, D.H.; Grover, S.A.; Kangro, H.O.; Clare, A.W. Incidence, risk and prognosis of acute and chronic fatigue syndromes and psychiatric disorders after glandular fever. *Br. J. Psychiatry* **1998**, *173*, 475–481.
2250. White, P.D. Chronic fatigue syndrome: Is it one discrete syndrome or many? Implications for the ``one vs. many{''} functional somatic syndromes debate. *J. Psychosom. Res.* **2010**, *68*, 455–459.
2251. White, R.F.; Steele, L.; O'Callaghan, J.P.; Sullivan, K.; Binns, J.H.; Golomb, B.A.; Bloom, F.E.; Bunker, J.A.; Crawford, F.; Graves, J.C.; et al. Recent research on Gulf War illness and other health problems in veterans of the 1991 Gulf War: Effects of toxicant exposures during deployment. *Cortex* **2016**, *74*, 449–475.
2252. Whitehead, L.; Campion, P. Can general practitioners manage Chronic Fatigue Syndrome? A controlled trial. *J. Chronic Fatigue Syndr.* **2002**, *10*, 55–64.
2253. Wiborg, J.F.; van der Werf, S.; Prins, J.B.; Bleijenberg, G. Being homebound with chronic fatigue syndrome: A multidimensional comparison with outpatients. *Psychiatry Res.* **2010**, *177*, 246–249.
2254. Wick, J.Y.; LaFleur, J. Fatigue: Implications for the elderly. *Consult. Pharm.* **2007**, *22*, 566–578.
2255. Wiedemann, B. Should patients with chronic fatigue syndrome be tested for hepatitis C? [Patient ist standig mude - Auf hepatitis C testen?]. *MMW-Fortschritte der Medizin* **2000**, *142*, 16–18. Work in German
2256. Wierwille, L. Fibromyalgia: Diagnosing and managing a complex syndrome. *J. Am. Acad. NURSE Pract.* **2012**, *24*, 184–192.
2257. Wijma, A.J.; Speksnijder, C.M.; Crom-Ottens, A.F.; Knulst-Verlaan, J.M.C.; Keizer, D.; Nijs, J.; van Wilgen, C.P. What is important in transdisciplinary pain neuroscience education? A qualitative study. *Disabil. Rehabil.* **2018**, *40*, 2181–2191.
2258. Wilbur, J.; Shaver, J.; Kogan, J.; Buntin, M.; Wang, E. Menopausal transition symptoms in midlife women living with fibromyalgia and chronic fatigue. *Health Care Women Int.* **2006**, *27*, 600–614.
2259. Wildman, M.J.; Ayres, J.G.; Raoult, D. Q fever: Still a mysterious disease. *QJM - Mon. J. Assoc. Physicians* **2002**, *95*, 491–492.
2260. Wilke, W.S. Can fibromyalgia and chronic fatigue syndrome be cured by surgery? *Cleve. Clin. J. Med.* **2001**, *68*, 277–279.
2261. Wilke, W.S.; Fouad-Tarazi, F.M.; Cash, J.M.; Calabrese, L.H. The connection between chronic fatigue syndrome and neurally mediated hypotension. *Cleve. Clin. J. Med.* **1998**, *65*, 261–266.
2262. Wilkinson, K.; Shapiro, C. Nonrestorative sleep: Symptom or unique diagnostic entity? *SLEEP Med.* **2012**, *13*, 561–569.
2263. Williams, D.K.; Galvin, T.A.; Ma, H.; Khan, A.S. Investigation of xenotropic murine leukemia virus-related virus (XMRV) in human and other cell lines. *BIOLOGICALS* **2011**, *39*, 378–383.
2264. Williams, G.S. Irlen syndrome: Expensive lenses for this ill defined syndrome exploit patients. *BMJ* **2014**, *349*.

2265. Williams, M. V; Cox, B.; Ariza, M.E. Herpesviruses dUTPases: A new family of pathogen-associated molecular pattern (PAMP) proteins with implications for human disease. *Pathogens* **2017**, *6*.
2266. Williams, T.E.; Chalder, T.; Sharpe, M.; White, P.D. Heterogeneity in chronic fatigue syndrome - empirically defined subgroups from the PACE trial. *Psychol. Med.* **2017**, *47*, 1454–1465.
2267. Wilson, A.; Hickie, I.; Hadzi-Pavlovic, D.; Wakefield, D.; Parker, G.; Straus, S.E.; Dale, J.; McCluskey, D.; Hinds, G.; Brickman, A.; et al. What is chronic fatigue syndrome? Heterogeneity within an international multicentre study. *Aust. N. Z. J. Psychiatry* **2001**, *35*, 520–527.
2268. Wilson, A.; Hickie, I.; Lloyd, A.; Hadzi-Pavlovic, D.; Boughton, C.; Dwyer, J.; Wakefield, D. Longitudinal study of outcome of chronic fatigue syndrome. *BMJ* **1994**, *308*, 756–759.
2269. Winceslaus, S.J.; Pinching, A.J.; Harris, A.; Ankrett, V.; Mark HIV diagnosis: Why and how do we miss important clues? *Sex. Transm. Infect.* **2008**, *84*, 101–102.
2270. Wing, Y.K.; Leung, C.M. Mental health impact of severe acute respiratory syndrome: a prospective study. *Hong Kong Med. J. = Xianggang yi xue za zhi* **2012**, *18 Suppl 3*, 24–27.
2271. Winger, A.; Kvarstein, G.; Wyller, V.B.; Ekstedt, M.; Sulheim, D.; Fagermoen, E.; Smastuen, M.C.; Helseth, S. Health related quality of life in adolescents with chronic fatigue syndrome: a cross-sectional study. *Health Qual. Life Outcomes* **2015**, *13*, 96.
2272. Winger, A.; Kvarstein, G.; Wyller, V.B.; Sulheim, D.; Fagermoen, E.; Smastuen, M.C.; Helseth, S. Pain and pressure pain thresholds in adolescents with chronic fatigue syndrome and healthy controls: a cross-sectional study. *BMJ Open* **2014**, *4*, e005920.
2273. Winker, R.; Rüdiger, H.W. Orthostatic intolerance - Importance in occupational medicine [Orthostatische intoleranz - Bedeutung in der arbeitsmedizin]. *Arbeitsmedizin Sozialmedizin Umweltmedizin* **2001**, *36*, 325–331. Work in German
2274. Winkler, L.; Thoreux, P.; Lhuissier, F.J. Influence of the QFES fatigue score on the incidence of injuries in middle school athletes [Lien entre le score de fatigue de l'enfant sportif et la présence de blessures en section sportive scolaire]. *Sci. Sport.* **2016**, *31*, 135–144. Work in French
2275. Winters, E.G.; Quinet, R.J. Chronic fatigue syndrome. *J. La. State Med. Soc.* **1992**, *144*, 260–270.
2276. Wirnsberger, R.M.; de Vries, J.; Wouters, E.F.M.; Drent, M. Clinical presentation of sarcoidosis in the Netherlands - An epidemiological study. *Neth. J. Med.* **1998**, *53*, 53–60.
2277. Witham, M.; Kennedy, G.; Belch, J.; Hill, A.; Khan, F. Association between vitamin D status and markers of vascular health in patients with chronic fatigue syndrome/myalgic encephalomyelitis (CFS/ME). *Int. J. Cardiol.* 2014, *174*, 139–140.
2278. Witthoft, M.; Hiller, W.; Loch, N.; Jasper, F. The latent structure of medically unexplained symptoms and its relation to functional somatic syndromes. *Int. J. Behav. Med.* **2013**, *20*, 172–183.
2279. Wittrup, I.H.; Jensen, B.; Bliddal, H.; Danneskiold-Samsoe, B.; Wiik, A. Comparison of viral antibodies in 2 groups of patients with fibromyalgia. *J. Rheumatol.* **2001**, *28*, 601–603.

2280. Woldehiwet, Z. Q fever (coxiellosis): Epidemiology and pathogenesis. *Res. Vet. Sci.* **2004**, *77*, 93–100.
2281. Wong, W.S.; Fielding, R. Prevalence of chronic fatigue among Chinese adults in Hong Kong: a population-based study. *J. Affect. Disord.* **2010**, *127*, 248–256.
2282. Wood, N.J. Infection: Giardia lamblia is associated with an increased risk of both IBS and chronic fatigue that persists for at least 3 years. *Nat. Rev. Gastroenterol. Hepatol.* **2011**, *8*, 597.
2283. Woolley, J.; Allen, R.; Wessely, S. Alcohol use in chronic fatigue syndrome. *J. Psychosom. Res.* **2004**, *56*, 203–206.
2284. Worm-Smeitink, M.; Nikolaus, S.; Goldsmith, K.; Wiborg, J.; Ali, S.; Knoop, H.; Chalder, T. Cognitive behaviour therapy for chronic fatigue syndrome: Differences in treatment outcome between a tertiary treatment centre in the United Kingdom and the Netherlands. *J. Psychosom. Res.* **2016**, *87*, 43–49.
2285. Wormser, G.P.; Dattwyler, R.J.; Shapiro, E.D.; Halperin, J.J.; Steere, A.C.; Klempner, M.S.; Krause, P.J.; Bakken, J.S.; Strle, F.; Stanek, G.; et al. The clinical assessment, treatment, and prevention of Lyme disease, human granulocytic anaplasmosis, and babesiosis: Clinical practice guidelines by the infectious diseases society of America. *Clin. Infect. Dis.* **2006**, *43*, 1089–1134.
2286. Wormser, G.P.; Shapiro, E.D. Implications of gender in chronic Lyme disease. *J. Womens. Health (Larchmt).* **2009**, *18*, 831–834.
2287. Wormser, G.P.; Shapiro, E.D.; Stricker, R.B.; Johnson, L. Gender Bias in Chronic Lyme Disease Reply. *J. WOMENS Heal.* **2009**, *18*, 1719–1720.
2288. Wu, C.-C.; Chung, S.-D.; Lin, H.-C. Endometriosis increased the risk of bladder pain syndrome/interstitial cystitis: A population-based study. *Neurourol. Urodyn.* **2018**, *37*, 1413–1418.
2289. Wu, H.-S.; Mengel, M.B. Unexplained prolonged fatigue in primary care. *J. Chronic Fatigue Syndr.* **2006**, *13*, 15–34.
2290. Wuttke, T. V; Lerche, H. Novel anticonvulsant drugs targeting voltage-dependent ion channels. *Expert Opin. Investig. Drugs* **2006**, *15*, 1167–1177.
2291. Wyller, V.B. The chronic fatigue syndrome - An update. *Acta Neurol. Scand.* **2007**, *115*, 7–14. Proceedings of the Annual Meeting of the Norwegian-Neurological-Association (Nevrodagene 2006) in Oslo, Norway.
2292. Wyller, V.B.; Vitelli, V.; Sulheim, D.; Fagermoen, E.; Winger, A.; Godang, K.; Bollerslev, J. Altered neuroendocrine control and association to clinical symptoms in adolescent chronic fatigue syndrome: a cross-sectional study. *J. Transl. Med.* **2016**, *14*, 121.
2293. Xiang, Y.T.; Yu, X.; Ungvari, G.S.; Correll, C.U.; Chiu, H.F.K. Outcomes of SARS survivors in China: Not only physical and psychiatric co-morbidities. *East Asian Arch. Psychiatry* **2014**, *24*, 37–38.
2294. Xiang, Y.; Lu, L.; Chen, X.; Wen, Z. Does Tai Chi relieve fatigue? A systematic review and meta-analysis of randomized controlled trials. *PLoS One* **2017**, *12*.
2295. Xie, Y.; Liu, B.; Piao, H. [Exploration on the common characters of sub-healthy people based on clinical epidemiology]. *Zhongguo Zhong xi yi jie he za zhi Zhongguo Zhongxiyi jiehe zazhi = Chinese J. Integr. Tradit. West. Med.* **2006**, *26*, 612–616. Work in Chinese
2296. Yalcin, S.; Kuratsune, H.; Yamaguchi, K.; Kitani, T.; Yamanishi, K. Prevalence of Human Herpesvirus 6 Variants A and B in Patients with Chronic Fatigue Syndrome. *Microbiol. Immunol.* **1994**, *38*, 587–590.
2297. Yamaguchi, K.; Sawada, T.; Naraki, T.; Igata-Yi, R.; Shiraki, H.; Horii, Y.; Ishii, T.; Ikeda, K.; Asou, N.; Okabe, H.; et al. Detection of Borna disease virus-reactive

antibodies from patients with psychiatric disorders and from horses by electrochemiluminescence immunoassay. *Clin. Diagn. Lab. Immunol.* **1999**, *6*, 696–700.
2298. Yamanishi, K. Human herpesvirus 6 and human herpesvirus 7 infections: The viruses and their disease manifestations. *Bailliere's Clin. Infect. Dis.* **1996**, *3*, 507–518.
2299. Yang, C.C.; Miller, J.L.; Omidpanah, A.; Krieger, J.N. Physical Examination for Men and Women With Urologic Chronic Pelvic Pain Syndrome: A MAPP (Multidisciplinary Approach to the Study of Chronic Pelvic Pain) Network Study. *Urology* **2018**, *116*, 23–29.
2300. Yang, J.; Battacharya, P.; Singhal, R.; Kandel, E.S. Xenotropic murine leukemia virus-related virus (XMRV) in prostate cancer cells likely represents a laboratory artifact. *Oncotarget* **2011**, *2*, 358–362.
2301. Yang, L.; Adams, J.; Sibbritt, D. Prevalence and factors associated with the use of acupuncture and Chinese medicine: Results of a nationally representative survey of 17161 Australian women. *Acupunct. Med.* **2017**, *35*, 189–199.
2302. Yang, T.-Y.; Kuo, H.-T.; Chen, H.-J.; Chen, C.-S.; Lin, W.-M.; Tsai, S.-Y.; Kuo, C.-N.; Kao, C.-H. Increased risk of chronic fatigue syndrome following atopy. *Med. (United States)* **2015**, *94*.
2303. Yang, T.-Y.; Chen, C.-S.; Lin, C.-L.; Lin, W.-M.; Kuo, C.-N.; Kao, C.-H. Risk for Irritable Bowel Syndrome in Fibromyalgia Patients A National Database Study. *Medicine (Baltimore).* **2015**, *94*.
2304. Yang, T.-Y.; Kuo, H.-T.; Chen, H.-J.; Chen, C.-S.; Lin, W.-M.; Tsai, S.-Y.; Kuo, C.-N.; Kao, C.-H. Increased Risk of Chronic Fatigue Syndrome Following Atopy: A Population-Based Study. *Medicine (Baltimore).* **2015**, *94*, e1211.
2305. Yao, K.; Crawford, J.R.; Komaroff, A.L.; Ablashi, D. V; Jacobson, S. Review part 2: Human herpesvirus-6 in central nervous system diseases. *J. Med. Virol.* **2010**, *82*, 1669–1678.
2306. Yip, H.K.; Cutress, T. Dental amalgam and human health. *Int. Dent. J.* **2003**, *53*, 464–468.
2307. Yiu, Y.-M.; Qiu, M.-Y. [A preliminary epidemiological study and discussion on traditional Chinese medicine pathogenesis of chronic fatigue syndrome in Hong Kong]. *Zhong Xi Yi Jie He Xue Bao* **2005**, *3*, 359–362. Work in Chinese
2308. Yoo, E.H.; Choi, E.S.; Cho, S.H.; Do, J.H.; Lee, S.J.; Kim, J.-H. Comparison of fatigue severity and quality of life between unexplained fatigue patients and explained fatigue patients. *Korean J. Fam. Med.* **2018**, *39*, 180–184.
2309. Yoshiuchi, K. [Psychological symptoms in chronic fatigue syndrome]. *Nihon Rinsho.* **2007**, *65*, 1023–1027. Work in Japanese
2310. Young, H.A.; Simmens, S.J.; Kang, H.K.; Mahan, C.M.; Levine, P.H. Factor analysis of fatiguing syndrome in Gulf War era veterans: implications for etiology and pathogenesis. *J. Occup. Environ. Med.* **2003**, *45*, 1268–1273.
2311. Young, J.L. Chronic fatigue syndrome: 3 cases and a discussion of the natural history of attention-deficit/hyperactivity disorder. *Postgrad. Med.* **2013**, *125*, 162–168.
2312. Youssefi, M.; Linkowski, P. Chronic fatigue syndrome : Psychiatric perspectives [Le syndrome de fatigue chronique : Aspects psychiatriques]. *Rev. Med. Brux.* **2002**, *23*, A299–A304. Work in French
2313. Yunus, M.B. The prevalence of fibromyalgia in other chronic pain conditions. *Pain Res. Treat.* **2012**, *2012*.

2314. Yunus, M.B. Gender differences in fibromyalgia and other related syndromes. *J. Gender-Specific Med.* **2002**, *5*, 42–47.
2315. Zachrisson, O.; Colque-Navarro, P.; Gottfries, C.G.; Regland, B.; Mollby, R. Immune modulation with a staphylococcal preparation in fibromyalgia/chronic fatigue syndrome: Relation between antibody levels and clinical improvement. *Eur. J. Clin. Microbiol. Infect. Dis.* **2004**, *23*, 98–105.
2316. Zachrisson, O.; Regland, B.; Jahreskog, M.; Jonsson, M.; Kron, M.; Gottfries, C.G. Treatment with staphylococcus toxoid in fibromyalgia/chronic fatigue syndrome - a randomised controlled trial. *Eur. J. PAIN-LONDON* **2002**, *6*, 455–466.
2317. Zachrisson, O.; Regland, B.; Jahreskog, M.; Kron, M.; Gottfries, C.G. A rating scale for fibromyalgia and chronic fatigue syndrome (the FibroFatigue scale). *J. Psychosom. Res.* **2002**, *52*, 501–509.
2318. Zadourian, A.; Doherty, T.A.; Swiatkiewicz, I.; Taub, P.R. Postural Orthostatic Tachycardia Syndrome: Prevalence, Pathophysiology, and Management. *Drugs* **2018**, *78*, 983–994.
2319. Zakrzewska, J.M. Chronic/Persistent Idiopathic Facial Pain. *Neurosurg. Clin. N. Am.* **2016**, *27*, 345–351.
2320. Zákutná, Ľ.; Dorko, E.; Mattová, E.; Rimárová, K. Sero-epidemiological study of Lyme disease among high-risk population groups in eastern Slovakia. *Ann. Agric. Environ. Med.* **2015**, *22*, 632–636.
2321. Zakzanis, K.K. Distinct neurocognitive profiles in multiple sclerosis subtypes. *Arch. Clin. Neuropsychol.* **2000**, *15*, 115–136.
2322. Zann, G.R. Removing the Stigma of chronic fatigue syndrome. *Pharm. Times* **2012**, *78*.
2323. Zanni, G.R. Diagnosing and treating fibromyalgia. *Consult. Pharm.* **2009**, *24*, 572–589.
2324. Zannolli, R.; Morgese, G. New pathogens, and diseases old and new. I) Afipia felis and Rochalimaea. II) Parvovirus B 19. III) herpesvirus 6. *Panminerva Med.* **1995**, *37*, 238–247.
2325. Zeliger, H.I. *Human Toxicology of Chemical Mixtures*; 2008;
2326. Zeller, B.; Loge, J.H.; Kanellopoulos, A.; Hamre, H.; Wyller, V.B.; Ruud, E. Chronic fatigue in long-term survivors of childhood lymphomas and leukemia: persistence and associated clinical factors. *J. Pediatr. Hematol. Oncol.* **2014**, *36*, 438–444.
2327. Zeller, B.; Ruud, E.; Loge, J.H.; Kanellopoulos, A.; Hamre, H.; Godang, K.; Wyller, V.B. Chronic Fatigue in Adult Survivors of Childhood Cancer: Associated Symptoms, Neuroendocrine Markers, and Autonomic Cardiovascular Responses. *Psychosomatics* **2014**, *55*, 621–629.
2328. Zevestoski, S.; Brown, P.; McCormick, S.; Mayer, B.; D'Ottavi, M.; Lucove, J.C. Patient activism and the struggle for diagnosis: Gulf War illnesses and other medically unexplained physical symptoms in the US. *Soc. Sci. Med.* **2004**, *58*, 161–175.
2329. Zhang, L.; Xu, M.-M.; Zeng, L.; Liu, S.; Liu, X.; Wang, X.; Li, D.; Huang, R.-Z.; Zhao, L.-B.; Zhan, Q.-L.; et al. Evidence for Borna disease virus infection in neuropsychiatric patients in three western China provinces. *Eur. J. Clin. Microbiol. Infect. Dis.* **2014**, *33*, 621–627.
2330. Zhang, Q.W.; Natelson, B.H.; Ottenweller, J.E.; Servatius, R.J.; Nelson, J.J.; De Luca, J.; Tiersky, L.; Lange, G. Chronic fatigue syndrome beginning suddenly occurs seasonally over the year. *Chronobiol. Int.* **2000**, *17*, 95–99.

2331. Zhang, W.; McLeod, C.; Koehoorn, M. The relationship between chronic conditions and absenteeism and associated costs in Canada. *Scand. J. Work. Environ. Heal.* **2016**, *42*, 413–422.
2332. Zhang, Y.; Zhang, J.; Zhu, S.; Du, C.; Zhang, W. Prevalence and Predictors of Somatic Symptoms among Child and Adolescents with Probable Posttraumatic Stress Disorder: A Cross-Sectional Study Conducted in 21 Primary and Secondary Schools after an Earthquake. *PLoS One* **2015**, *10*.
2333. Zhang, Z.-Z.; Guo, B.-F.; Feng, Z.; Zhang, L.; Zhao, X.-J. Is XMRV a causal virus for prostate cancer? *ASIAN J. Androl.* **2011**, *13*, 698–701.
2334. Zhou, Y.; Steffen, I.; Montalvo, L.; Lee, T.-H.; Zemel, R.; Switzer, W.M.; Tang, S.; Jia, H.; Heneine, W.; Winkelman, V.; et al. Development and application of a high-throughput microneutralization assay: Lack of xenotropic murine leukemia virus-related virus and/or murine leukemia virus detection in blood donors. *Transfusion* **2012**, *52*, 332–342.
2335. Ziem, G.; Donnay, A. Chronic Fatigue, Fibromyalgia, and Chemical Sensitivity: Overlapping Disorders. *Arch. Intern. Med.* **1995**, *155*, 1913.
2336. Zsuzsa, A. Chronic fatigue syndrome [Krónikus fáradtság szindróma]. *Orvoskepzes* **2003**, *78*, 60–64. Work in Hungarian
2337. Zuckerman, J.N. Protective efficacy, immunotherapeutic potential, and safety of hepatitis B vaccines. *J. Med. Virol.* **2006**, *78*, 169–177.
2338. Zverova, M. Frequency of some psychosomatic symptoms in informal caregivers of Alzheimer's disease individuals. Prague's experience. *Neuro Endocrinol. Lett.* **2012**, *33*, 565–567.
2339. Zwarts, M.J.; Bleijenberg, G.; van Engelen, B.G.M. Clinical neurophysiology of fatigue. *Clin. Neurophysiol.* **2008**, *119*, 2–10.
2340. Erratum: Multi-site clinical assessment of myalgic encephalomyelitis/chronic fatigue syndrome (MCAM): Design and implementation of a prospective/retrospective rolling cohort study (American Journal of Epidemiology (2017) 185:8 (617-626) DOI: 10.1093/aje/kwx029). *Am. J. Epidemiol.* **2017**, *186*, 129.
2341. Adverse effects of human papillomavirus vaccines: Data available in 2011. *Prescrire Int.* **2012**, *21*, 156–157.
2342. Pain management programmes for chronic pain? *Drug Ther. Bull.* **2011**, *49*, 33–36.
2343. Chronic fatigue syndrome and pregnancy. *Med. Today* **2004**, *5*, 10.
2344. Robin Goodfellow. *Rheumatology* **2002**, *41*, 478.
2345. Summaries for patients. The health of Gulf War veterans. *Ann. Intern. Med.* 2005, *142*, I22.
2346. Alleged link between hepatitis B vaccine and chronic fatigue syndrome. *Can. Dis. Wkly. Rep.* **1991**, *17*, 215–216.
2347. Multiple chemical sensitivity: a 1999 consensus. *Arch. Environ. Health* **1999**, *54*, 147–149.
2348. Frustrating survey of chronic fatigue. *Lancet (London, England)* 1996, *348*, 971.
