# Supplementary material for: Systematic Review of the Epidemiological Burden of Myalgic Encephalomyelitis/Chronic Fatigue Syndrome Across Europe: Current Evidence and EUROMENE Research Recommendations for Epidemiology
Source: J Clin Med. 2020 May 21;9(5):1557. doi: 10.3390/jcm9051557 (PMC7290765; doi:10.3390/jcm9051557)
Supplement: Supplementary file 1 [file jcm-09-01557-s001.zip › supplementary-files/jcm-784233-supplementary-file3.pdf]

List of references excluding after screening the full-text (n=40)

1. Aggarwal, V.R.; McBeth, J.; Zakrzewska, J.M.; Lunt, M.; Macfarlane, G.J. The epidemiology of chronic syndromes that are frequently unexplained: do they have common associated factors? *Int. J. Epidemiol.* **2006**, *35*, 468–476. Rationale for exclusion: inappropriate case definition for the purposes of the present review.
2. Avellaneda Fernandez, A.; Perez Martin, A.; Izquierdo Martinez, M.; Arruti Bustillo, M.; Barbado Hernandez, F.J.; de la Cruz Labrado, J.; Diaz-Delgado Penas, R.; Gutierrez Rivas, E.; Palacin Delgado, C.; Rivera Redondo, J.; et al. Chronic fatigue syndrome: aetiology, diagnosis and treatment. *BMC Psychiatry* **2009**, *9*. Rationale for exclusion: no primary data (literature review).
3. Bakken, I.J.; Tveito, K.; Gunnes, N.; Ghaderi, S.; Stoltenberg, C.; Trogstad, L.; Haberg, S.E.; Magnus, P. Two age peaks in the incidence of chronic fatigue syndrome/myalgic encephalomyelitis: a population-based registry study from Norway 2008-2012. *BMC Med.* **2014**, *12*, 167. Rationale for exclusion: inappropriate case definition for the purposes of the present review.
4. Bhui, K.S.; Dinos, S.; Ashby, D.; Nazroo, J.; Wessely, S.; White, P.D. Chronic fatigue syndrome in an ethnically diverse population: the influence of psychosocial adversity and physical inactivity. *BMC Med.* **2011**, *9*, 26. Rationale for exclusion: the review includes another study from the same country and with a larger sample size.
5. Campistol, J. Chronic fatigue syndrome in adolescents [Sindrome de fatiga cronica a l'adolescencia]. *Pediatr. Catalana* **1997**, *57*, 294–302. Rationale for exclusion: no primary data (literature review).
6. Castro-Marrero, J.; Faro, M.; Aliste, L.; Saez-Francas, N.; Calvo, N.; Martinez-Martinez, A.; de Sevilla, T.F.; Alegre, J. Comorbidity in Chronic Fatigue Syndrome/Myalgic Encephalomyelitis: A Nationwide Population-Based Cohort Study. *Psychosomatics* **2017**, *58*, 533–543. Rationale for exclusion: no prevalence/incidence data.
7. Cho, H.J.; Menezes, P.R.; Hotopf, M.; Bhugra, D.; Wessely, S. Comparative epidemiology of chronic fatigue syndrome in Brazilian and British primary care: prevalence and recognition. *Br. J. Psychiatry* **2009**, *194*, 117–122. Rationale for exclusion: the review includes another study from the same country and with a larger sample size.
8. Chu, L.; Valencia, I.J.; Garvert, D.W.; Montoya, J.G. Onset patterns and course of myalgic encephalomyelitis/chronic fatigue syndrome. *Front. Pediatr.* **2019**, *7*. Rationale for exclusion: no prevalence/incidence data.
9. Clark, C.; Goodwin, L.; Stansfeld, S.A.; Hotopf, M.; White, P.D. Premorbid risk markers for chronic fatigue syndrome in the 1958 British birth cohort. *Br. J. Psychiatry* **2011**, *199*, 323–329. Rationale for exclusion: inappropriate case definition for the purposes of the present review.
10. Colby, J. Special problems of children with myalgic encephalomyelitis/chronic fatigue syndrome and the enteroviral link. *J. Clin. Pathol.* **2007**, *60*, 125–128. Rationale for exclusion: no primary data (literature review).
11. Dantoft, T.M.; Ebstrup, J.F.; Linneberg, A.; Skovbjerg, S.; Madsen, A.L.; Mehlsen, J.; Brinth, L.; Eplov, L.F.; Carstensen, T.W.; Schroder, A.; et al. Cohort description: The Danish study of functional disorders. *Clin. Epidemiol.* **2017**, *9*, 127–139. Rationale for exclusion: inappropriate case definition for the purposes of the present review.
12. Dowsett, E.G.; Colby, J. Long-term sickness absence due to ME/CFS in UK schools: An epidemiological study with medical and educational implications. *J. Chronic*

Fatigue Syndr. **1997**, *3*, 29–42. Rationale for exclusion: inappropriate case definition for the purposes of the present review.
13. Farmer, A.; Fowler, T.; Scourfield, J.; Thapar, A. Prevalence of chronic disabling fatigue in children and adolescents. *Br. J. Psychiatry* **2004**, *184*, 477–481. Rationale for exclusion: selected (biased) sample as only twins participated.
14. Evengård, B.; Jacks, A.; Pedersen, N.L.; Sullivan, P.F.; Evengard, B.; Jacks, A.; Pedersen, N.L.; Sullivan, P.F. The epidemiology of chronic fatigue in the Swedish Twin Registry. *Psychol. Med.* **2005**, *35*, 1317–1326. Rationale for exclusion: inappropriate case definition for the purposes of the present review.
15. Gallagher, A.M.; Thomas, J.M.; Hamilton, W.T.; White, P.D. Incidence of fatigue symptoms and diagnoses presenting in UK primary care from 1990 to 2001. *J. R. Soc. Med.* **2004**, *97*, 571–575. Rationale for exclusion: inappropriate case definition for the purposes of the present review.
16. Harvey, S.B.; Wadsworth, M.; Wessely, S.; Hotopf, M. The relationship between prior psychiatric disorder and chronic fatigue: evidence from a national birth cohort study. *Psychol. Med.* **2008**, *38*, 933–940. Rationale for exclusion: inappropriate case definition for the purposes of the present review.
17. Harvey, S.B.; Wadsworth, M.; Wessely, S.; Hotopf, M. Etiology of chronic fatigue syndrome: testing popular hypotheses using a national birth cohort study. *Psychosom. Med.* **2008**, *70*, 488–495. Rationale for exclusion: inappropriate case definition for the purposes of the present review.
18. Huibers, M.J.H.; Kant, I.J.; Swaen, G.M.H.; Kasl, S. V Prevalence of chronic fatigue syndrome-like caseness in the working population: results from the Maastricht cohort study. *Occup. Environ. Med.* **2004**, *61*, 464–466. Rationale for exclusion: inappropriate case definition for the purposes of the present review.
19. Janssens, K.A.M.; Zijlema, W.L.; Joustra, M.L.; Rosmalen, J.G.M. Mood and Anxiety Disorders in Chronic Fatigue Syndrome, Fibromyalgia, and Irritable Bowel Syndrome: Results From the LifeLines Cohort Study. *Psychosom. Med.* **2015**, *77*, 449–457. Rationale for exclusion: inappropriate case definition for the purposes of the present review.
20. Jason, L.A.; Richman, J.A.; Rademaker, A.W.; Jordan, K.M.; Plioplys, A. V; Taylor, R.R.; McCready, W.; Huang, C.F.; Plioplys, S. A community-based study of chronic fatigue syndrome. *Arch. Intern. Med.* **1999**, *159*, 2129–2137. Rationale for exclusion: non-European sample.
21. Jason, L.A.; Taylor, R.; Wagner, L.; Holden, J.; Ferrari, J.R.; Plioplys, A. V; Plioplys, S.; Lipkin, D.; Papernik, M. Estimating rates of chronic fatigue syndrome from a community-based sample: a pilot study. *Am. J. Community Psychol.* **1995**, *23*, 557–568. Rationale for exclusion: non-European sample.
22. Jason, L.A.; Porter, N.; Hunnell, J.; Rademaker, A.; Richman, J.A. CFS prevalence and risk factors over time. *J. Health Psychol.* **2011**, *16*, 445–456. Rationale for exclusion: non-European sample.
23. Jones, J.F.; Nisenbaum, R.; Solomon, L.; Reyes, M.; Reeves, W.C. Chronic fatigue syndrome and other fatiguing illnesses in adolescents: a population-based study. *J. Adolesc. Health* **2004**, *35*, 34–40. Rationale for exclusion: non-European sample.
24. Jordan, K.M.; Jason, L.A.; Mears, C.J.; Katz, B.Z.; Rademaker, A.; Huang, C.-F.; Richman, J.; McCready, W.; Ayers, P.M.; Taylor, K.K. Prevalence of pediatric chronic fatigue syndrome in a community-based sample. *J. Chronic Fatigue Syndr.* **2006**, *13*, 75–78. Rationale for exclusion: Non-European sample.

25. Lawrie, S.M.; Pelosi, A.J. Chronic fatigue syndrome in the community. Prevalence and associations. *Br. J. Psychiatry* **1995**, *166*, 793–797. Rationale for exclusion: inappropriate case definition for the purposes of the present review.
26. Martin, A.; Chalder, T.; Rief, W.; Braehler, E. The relationship between chronic fatigue and somatization syndrome: a general population survey. *J. Psychosom. Res.* **2007**, *63*, 147–156. Rationale for exclusion: inappropriate case definition for the purposes of the present review.
27. Mears, C.J.; Taylor, R.R.; Jordan, K.M.; Binns, H.J. Sociodemographic and symptom correlates of fatigue in an adolescent primary care sample. *J. Adolesc. Health* **2004**, *35*, 528e.21-6. Rationale for exclusion: inappropriate case definition for the purposes of the present review.
28. Morelli, V. Fatigue and Chronic Fatigue in the Elderly: Definitions, Diagnoses, and Treatments. *Clin. Geriatr. Med.* **2011**, *27*, 673–686. Rationale for exclusion: no primary data (literature review).
29. Nijhof, S.L.; Maijer, K.; Bleijenberg, G.; Uiterwaal, C.S.P.M.; Kimpen, J.L.L.; van de Putte, E.M. Adolescent chronic fatigue syndrome: prevalence, incidence, and morbidity. *Pediatrics* **2011**, *127*, e1169-75. Rationale for exclusion: inappropriate case definition for the purposes of the present review.
30. Norris, T.; Deere, K.; Tobias, J.H.; Crawley, E. Chronic Fatigue Syndrome and Chronic Widespread Pain in Adolescence: Population Birth Cohort Study. *J. Pain* **2017**, *18*, 285–294. Rationale for exclusion: inappropriate case definition for the purposes of the present review.
31. Pawlikowska, T.; Chalder, T.; Hirsch, S.R.; Wallace, P.; Wright, D.J.; Wessely, S.C. Population based study of fatigue and psychological distress. *BMJ* **1994**, *308*, 763–766. Rationale for exclusion: inappropriate case definition for the purposes of the present review.
32. Price, R.K.; North, C.S.; Wessely, S.; Fraser, V.J. Estimating the prevalence of chronic fatigue syndrome and associated symptoms in the community. *Public Health Rep.* **1992**, *107*, 514–522. Rationale for exclusion: non-European sample.
33. Ranjith, G. Epidemiology of chronic fatigue syndrome. *Occup. Med. (Lond).* **2005**, *55*, 13–19. Rationale for exclusion: no primary data (literature review).
34. Rask, C.U. Functional somatic symptoms in 5-7-year-old children. Assessment, prevalence and co-occurrence. *Dan. Med. J.* **2012**, *59*. Rationale for exclusion: no prevalence/incidence data.
35. Reid, S.F.; Chalder, T.; Cleare, A.; Hotopf, M.; Wessely, S. Chronic fatigue syndrome. *BMJ Clin. Evid.* **2008**, *2008*. Rationale for exclusion: no primary data (literature review).
36. Slomko, J.; Newton, J.L.; Kujawski, S.; Tafil-Klawe, M.; Klawe, J.; Staines, D.; Marshall-Gradisnik, S.; Zalewski, P. Prevalence and characteristics of chronic fatigue syndrome/myalgic encephalomyelitis (CFS/ME) in Poland: A cross-sectional study. *BMJ Open* **2019**, *9*. Rationale for exclusion: selected (biased) sample. Only participants who experienced symptoms of fatigue were recruited.
37. van't Leven, M.; Zielhuis, G.A.; van der Meer, J.W.; Verbeek, A.L.; Bleijenberg, G. Fatigue and chronic fatigue syndrome-like complaints in the general population. *Eur. J. Public Health* **2010**, *20*, 251–257. Rationale for exclusion: Inappropriate case definition for the purposes of the present review.
38. Watanabe, N.; Stewart, R.; Jenkins, R.; Bhugra, D.K.; Furukawa, T.A. The epidemiology of chronic fatigue, physical illness, and symptoms of common mental disorders: a cross-sectional survey from the second British National Survey of

Psychiatric Morbidity. *J. Psychosom. Res.* **2008**, *64*, 357–362. Rationale for exclusion: Inappropriate case definition for the purposes of the present review.
39. Wessely, S.; Chalder, T.; Hirsch, S.; Wallace, P.; Wright, D. The prevalence and morbidity of chronic fatigue and chronic fatigue syndrome: a prospective primary care study. *Am. J. Public Health* **1997**, *87*, 1449–1455. Rationale for exclusion: inappropriate case definition for the purposes of the present review.
40. Wu, H.-S.; Mengel, M.B. Unexplained prolonged fatigue in primary care. *J. Chronic Fatigue Syndr.* **2006**, *13*, 15–34. Rationale for exclusion: no primary data (literature review).
